# Supplementary material for: Normal Ageing Impacts the Extent and Diversity of Neural Plasticity Induced in the Mouse Brain With Repetitive Transcranial Magnetic Stimulation
Source: Aging Cell. 2025 Sep 1;24(10):e70206. doi: 10.1111/acel.70206 (PMC12507392; doi:10.1111/acel.70206)
Supplement: Supplementary file 1 — Data S1: acel70206‐sup‐0001‐DataS1‐S9.pdf. [file ACEL-24-e70206-s003.pdf]

Data S1. Summary of spatial transcriptomics samples.

Sample ID	ET22A01	ET22A02	ET22A03	ET22A04	ET22A05	ET22A06	ET22A07	ET22A09	ET22A11	ET22A12	ET22A13	ET22A14
Stimulation condition	iTBS	cTBS	sham	sham	cTBS	iTBS	sham	cTBS	iTBS	sham	iTBS	cTBS
Visium slide number	1	1	4	4	3	2	2	4	4	3	3	3
Visium area number	A1	B1	A1	C1	A1	A1	B1	B1	D1	B1	C1	D1
Number of Reads	213720183	203973068	218242715	211621221	231707408	200887856	231879150	208106723	203712276	219756357	197475094	205859379

Data S2. Differentially expressed genes (DEGs) identified in sub-cortical regions following iTBS, and cTBS. DEGs were identified using a cutoff of p.adj ≤ 0.05.

Brain region	Stimulation	Gene Symbol	Gene Name	EntrezID	Avg log2FoldChange	% expressed in stimulated samples	% expressed in sham samples	p.adjust value (Bonferroni correction)	p.value
White Matter Tracts	iTBS	Bc1	brain cytoplasmic RNA 1	100568459	-0.433049208	0.816	0.885	0.017471008	1.18E-06
White Matter Tracts	iTBS	Snx3	sorting nexin 3	54198	-0.256664141	0.791	0.877	0.002128645	1.44E-07
White Matter Tracts	iTBS	Dync1h1	dynein cytoplasmic 1 heavy chain 1	13424	-0.235728648	0.827	0.913	0.002055333	1.39E-07
White Matter Tracts	iTBS	Ikbkb	inhibitor of kappaB kinase beta	16150	-0.232327107	0.049	0.127	0.031666974	2.14E-06
White Matter Tracts	iTBS	Atp6v0d1	ATPase, H+ transporting, lysosomal V0 subunit D1	11972	-0.221361557	0.773	0.868	0.020105921	1.36E-06
White Matter Tracts	iTBS	Gprasp1	G protein-coupled receptor associated sorting protein 1	67298	-0.218646244	0.731	0.865	0.000181459	1.23E-08
White Matter Tracts	iTBS	Cyfip2	cytoplasmic FMR1 interacting protein 2	76884	-0.206299765	0.747	0.862	0.000484227	3.28E-08
White Matter Tracts	iTBS	Atp6v0a1	ATPase, H+ transporting, lysosomal V0 subunit A1	11975	-0.200278917	0.717	0.813	0.011909707	8.06E-07
White Matter Tracts	iTBS	Itpr1	inositol 1,4,5-trisphosphate receptor 1	16438	-0.178168816	0.686	0.822	0.00638697	4.32E-07
White Matter Tracts	iTBS	Syn2	synapsin II	20965	-0.177677157	0.724	0.843	0.01599833	1.08E-06
White Matter Tracts	iTBS	Sptbn2	spectrin beta, non-erythrocytic 2	20743	-0.174942322	0.686	0.818	0.003636036	2.46E-07
White Matter Tracts	iTBS	Sv2a	synaptic vesicle glycoprotein 2 a	64051	-0.172060746	0.749	0.833	0.026762547	1.81E-06
White Matter Tracts	iTBS	Kifap3	kinesin-associated protein 3	16579	-0.171420558	0.718	0.847	0.002705414	1.83E-07
White Matter Tracts	iTBS	Fabp3	fatty acid binding protein 3, muscle and heart	14077	-0.171345547	0.659	0.768	0.019439221	1.32E-06
White Matter Tracts	iTBS	Map2	microtubule-associated protein 2	17756	-0.167642608	0.755	0.872	0.000599496	4.06E-08
White Matter Tracts	iTBS	Epn1	epsin 1	13854	-0.167487046	0.771	0.88	0.027127363	1.84E-06
White Matter Tracts	iTBS	Map1a	microtubule-associated protein 1 A	17754	-0.165190308	0.727	0.843	0.012618462	8.54E-07
White Matter Tracts	iTBS	Atp6v1d	ATPase, H+ transporting, lysosomal V1 subunit D	73834	-0.164873352	0.76	0.858	0.03834351	2.59E-06
White Matter Tracts	iTBS	Pdxp	pyridoxal (pyridoxine, vitamin B6) phosphatase	57028	-0.164469269	0.711	0.828	0.041748572	2.83E-06
White Matter Tracts	iTBS	Nat8l	N-acetyltransferase 8-like	269642	-0.158046699	0.623	0.742	0.013943774	9.44E-07
White Matter Tracts	iTBS	Enc1	ectodermal-neural cortex 1	13803	-0.156182649	0.652	0.765	0.043706695	2.96E-06
White Matter Tracts	iTBS	Gnao1	guanine nucleotide binding protein, alpha O	14681	-0.154016163	0.821	0.913	0.025468443	1.72E-06
White Matter Tracts	iTBS	Atxn10	ataxin 10	54138	-0.142676998	0.764	0.88	0.045877523	3.10E-06
White Matter Tracts	iTBS	Pde2a	phosphodiesterase 2A, cGMP-stimulated	207728	-0.135384779	0.662	0.79	0.011522815	7.80E-07
White Matter Tracts	iTBS	Dync1i1	dynein cytoplasmic 1 intermediate chain 1	13426	0.043993426	0.231	0.088	2.18E-05	1.48E-09
White Matter Tracts	iTBS	Praf2	PRA1 domain family 2	54637	0.068533939	0.236	0.113	0.012763434	8.64E-07
White Matter Tracts	iTBS	Prmt1	protein arginine N-methyltransferase 1	15469	0.071608496	0.439	0.275	0.025418944	1.72E-06
White Matter Tracts	iTBS	C1d	C1D nuclear receptor co-repressor	57316	0.071951824	0.291	0.128	3.46E-05	2.34E-09
White Matter Tracts	iTBS	Cacybp	calcyclin binding protein	12301	0.073311383	0.273	0.133	0.003565862	2.41E-07
White Matter Tracts	iTBS	Actr3	ARP3 actin-related protein 3	74117	0.074342733	0.498	0.338	0.037685894	2.55E-06
White Matter Tracts	iTBS	Hspd1	heat shock protein 1 (chaperonin)	15510	0.074386776	0.417	0.255	0.020258934	1.37E-06
White Matter Tracts	iTBS	Mmd2	monocyte to macrophage differentiation-associated 2	75104	0.074645476	0.547	0.35	0.01576143	1.07E-06
White Matter Tracts	iTBS	Aars	alanyl-tRNA synthetase	234734	0.075200505	0.332	0.173	0.003284767	2.22E-07
White Matter Tracts	iTBS	Ehmt2	euchromatic histone lysine N-methyltransferase 2	110147	0.083212026	0.397	0.243	0.013814079	9.35E-07
White Matter Tracts	iTBS	Atxn7l3	ataxin 7-like 3	217218	0.083408609	0.363	0.205	0.002629173	1.78E-07
White Matter Tracts	iTBS	Cct8	chaperonin containing Tcp1, subunit 8 (theta)	12469	0.084224245	0.487	0.31	0.001561987	1.06E-07
White Matter Tracts	iTBS	Rnf126	ring finger protein 126	70294	0.091064402	0.236	0.1	0.00016056	1.09E-08
White Matter Tracts	iTBS	Ik	IK cytokine	24010	0.091410334	0.321	0.152	5.97E-05	4.04E-09
White Matter Tracts	iTBS	Commd4	COMM domain containing 4	66199	0.092188233	0.531	0.357	0.017663457	1.20E-06
White Matter Tracts	iTBS	Brinp1	bone morphogenic protein/retinoic acid inducible neural specific 1	56710	0.093189454	0.392	0.243	0.014316736	9.69E-07
White Matter Tracts	iTBS	Pdxk	pyridoxal (pyridoxine, vitamin B6) kinase	216134	0.093963537	0.41	0.257	0.036084628	2.44E-06
White Matter Tracts	iTBS	Gng2	guanine nucleotide binding protein (G protein), gamma 2	14702	0.09478512	0.211	0.093	0.002334959	1.58E-07
White Matter Tracts	iTBS	Cct2	chaperonin containing Tcp1, subunit 2 (beta)	12461	0.095425437	0.455	0.288	0.01317107	8.91E-07
White Matter Tracts	iTBS	Mgll	monoglyceride lipase	23945	0.09561621	0.341	0.187	0.003867957	2.62E-07
White Matter Tracts	iTBS	Use1	unconventional SNARE in the ER 1 homolog (S. cerevisiae)	67023	0.097433564	0.448	0.26	0.001912664	1.29E-07
White Matter Tracts	iTBS	Chd3	chromodomain helicase DNA binding protein 3	216848	0.098713078	0.357	0.183	7.58E-05	5.13E-09
White Matter Tracts	iTBS	Pip5k1c	phosphatidylinositol-4-phosphate 5-kinase, type 1 gamma	18717	0.10321869	0.278	0.127	5.35E-05	3.62E-09
White Matter Tracts	iTBS	Mrpl11	mitochondrial ribosomal protein L11	66419	0.104494362	0.412	0.225	5.54E-05	3.75E-09
White Matter Tracts	iTBS	Mrpl28	mitochondrial ribosomal protein L28	68611	0.104999452	0.439	0.257	0.000673266	4.56E-08
White Matter Tracts	iTBS	Calb1	calbindin 1	12307	0.105974317	0.394	0.252	0.044200825	2.99E-06
White Matter Tracts	iTBS	Nol7	nucleolar protein 7	70078	0.106189258	0.551	0.36	0.003437455	2.33E-07
White Matter Tracts	iTBS	Smpd1	sphingomyelin phosphodiesterase 1, acid lysosomal	20597	0.106524662	0.404	0.237	0.00226689	1.53E-07
White Matter Tracts	iTBS	Hnrnpa0	heterogeneous nuclear ribonucleoprotein A0	77134	0.106585064	0.397	0.232	0.005579856	3.78E-07
White Matter Tracts	iTBS	Gm17018	NA	NA	0.106742041	0.431	0.263	0.010717744	7.25E-07
White Matter Tracts	iTBS	Dgcr6	DiGeorge syndrome critical region gene 6	13353	0.10762072	0.26	0.137	0.049144235	3.33E-06
White Matter Tracts	iTBS	Cs	citrate synthase	12974	0.109865098	0.379	0.203	0.000336764	2.28E-08
White Matter Tracts	iTBS	Celsr2	cadherin, EGF LAG seven-pass G-type receptor 2	53883	0.111046647	0.227	0.092	3.90E-05	2.64E-09
White Matter Tracts	iTBS	Nudt19	nudix (nucleoside diphosphate linked moiety X)-type motif 19	110959	0.111249745	0.282	0.127	3.01E-05	2.04E-09
White Matter Tracts	iTBS	Smap2	small ArfGAP 2	69780	0.111591752	0.403	0.245	0.0109304	7.40E-07
White Matter Tracts	iTBS	Iqsec1	IQ motif and Sec7 domain 1	232227	0.111709673	0.422	0.257	0.009541888	6.46E-07
White Matter Tracts	iTBS	Grb2	growth factor receptor bound protein 2	14784	0.111977286	0.518	0.352	0.030964313	2.10E-06
White Matter Tracts	iTBS	Ube2v2	ubiquitin-conjugating enzyme E2 variant 2	70620	0.113223351	0.31	0.157	0.000789442	5.34E-08
White Matter Tracts	iTBS	Dkk3	dickkopf WNT signaling pathway inhibitor 3	50781	0.115076524	0.412	0.242	0.000396191	2.68E-08
White Matter Tracts	iTBS	Rufy3	RUN and FYVE domain containing 3	52822	0.115076524	0.504	0.333	0.010396851	7.04E-07
White Matter Tracts	iTBS	Kcnk1	potassium channel, subfamily K, member 1	16525	0.115076524	0.403	0.24	0.001541284	1.04E-07
White Matter Tracts	iTBS	Acly	ATP citrate lyase	104112	0.116683984	0.493	0.305	0.000394228	2.67E-08
White Matter Tracts	iTBS	Epdr1	ependymin related protein 1 (zebrafish)	105298	0.116726262	0.468	0.282	0.000311743	2.11E-08
White Matter Tracts	iTBS	Bod1	biorientation of chromosomes in cell division 1	69556	0.116735748	0.469	0.303	0.009964813	6.74E-07
White Matter Tracts	iTBS	Prkce	protein kinase C, epsilon	18754	0.116817858	0.437	0.273	0.003240253	2.19E-07
White Matter Tracts	iTBS	Dmwd	dystrophia myotonica-containing WD repeat motif	13401	0.118340542	0.43	0.248	0.000629985	4.26E-08
White Matter Tracts	iTBS	Tspan5	tetraspanin 5	56224	0.118475101	0.431	0.25	0.00028039	1.90E-08
White Matter Tracts	iTBS	Rnf10	ring finger protein 10	50849	0.118532083	0.394	0.22	0.000285255	1.93E-08
White Matter Tracts	iTBS	Klhdc3	kelch domain containing 3	71765	0.118578213	0.419	0.25	0.000771326	5.22E-08
White Matter Tracts	iTBS	Gprasp2	G protein-coupled receptor associated sorting protein 2	245607	0.118878095	0.323	0.147	1.75E-06	1.19E-10
White Matter Tracts	iTBS	Hmgb1	high mobility group box 1	15289	0.119146189	0.592	0.393	0.003647968	2.47E-07
White Matter Tracts	iTBS	Sar1a	secretion associated Ras related GTPase 1A	20224	0.120201557	0.399	0.222	0.000404872	2.74E-08
White Matter Tracts	iTBS	Klhl2	kelch-like 2, Mayven	77113	0.120935193	0.551	0.35	0.002103163	1.42E-07
White Matter Tracts	iTBS	Fam171b	family with sequence similarity 171, member B	241520	0.121404144	0.466	0.285	0.001457007	9.86E-08
White Matter Tracts	iTBS	Clpp	caseinolytic mitochondrial matrix peptidase proteolytic subunit	53895	0.123269023	0.444	0.265	0.002141941	1.45E-07
White Matter Tracts	iTBS	H13	histocompatibility 13	14950	0.123689655	0.135	0.053	0.048384163	3.27E-06
White Matter Tracts	iTBS	Reps2	RALBP1 associated Eps domain containing protein 2	194590	0.124110336	0.394	0.228	0.00073276	4.96E-08
White Matter Tracts	iTBS	Mbnl2	muscleblind like splicing factor 2	105559	0.124671681	0.619	0.443	0.027265637	1.85E-06
White Matter Tracts	iTBS	Arnt2	aryl hydrocarbon receptor nuclear translocator 2	11864	0.125320556	0.363	0.177	1.17E-05	7.94E-10
White Matter Tracts	iTBS	Rprml	reprimo-like	104582	0.125829581	0.406	0.25	0.004303787	2.91E-07
White Matter Tracts	iTBS	Hmox2	heme oxygenase 2	15369	0.126366538	0.361	0.192	0.001109806	7.51E-08
White Matter Tracts	iTBS	Ahsa1	AHA1, activator of heat shock protein ATPase 1	217737	0.126421498	0.3	0.14	8.79E-05	5.95E-09
White Matter Tracts	iTBS	Gsk3b	glycogen synthase kinase 3 beta	56637	0.127645198	0.388	0.217	0.000151507	1.03E-08
White Matter Tracts	iTBS	Asphd2	aspartate beta-hydroxylase domain containing 2	72898	0.128932046	0.455	0.29	0.001743015	1.18E-07
White Matter Tracts	iTBS	Rbfox3	RNA binding protein, fox-1 homolog (C. elegans) 3	52897	0.129073676	0.276	0.132	0.000128826	8.72E-09
White Matter Tracts	iTBS	Denr	density-regulated protein	68184	0.12915171	0.381	0.193	1.98E-05	1.34E-09
White Matter Tracts	iTBS	Atp6v1h	ATPase, H+ transporting, lysosomal V1 subunit H	108664	0.129431817	0.209	0.087	0.000369757	2.50E-08
White Matter Tracts	iTBS	Slc39a10	solute carrier family 39 (zinc transporter), member 10	227059	0.12961785	0.231	0.073	1.85E-08	1.25E-12
White Matter Tracts	iTBS	Rab18	RAB18, member RAS oncogene family	19330	0.129704736	0.291	0.147	0.00348324	2.36E-07
White Matter Tracts	iTBS	Evl	Ena-vasodilator stimulated phosphoprotein	14026	0.130104734	0.319	0.168	0.000501413	3.39E-08
White Matter Tracts	iTBS	Pianp	PILR alpha associated neural protein	319352	0.131635616	0.227	0.075	7.43E-08	5.03E-12
White Matter Tracts	iTBS	Ahi1	Abelson helper integration site 1	52906	0.131793919	0.455	0.298	0.015147866	1.03E-06
White Matter Tracts	iTBS	Ndufa9	NADH:ubiquinone oxidoreductase subunit A9	66108	0.132016387	0.303	0.127	1.12E-06	7.56E-11
White Matter Tracts	iTBS	Sesn3	sestrin 3	75747	0.132105254	0.327	0.153	9.80E-06	6.63E-10
White Matter Tracts	iTBS	Fbxo44	F-box protein 44	230903	0.132909818	0.394	0.2	3.17E-06	2.14E-10
White Matter Tracts	iTBS	Gng10	guanine nucleotide binding protein (G protein), gamma 10	14700	0.132998432	0.282	0.142	0.001857206	1.26E-07
White Matter Tracts	iTBS	Mapre2	microtubule-associated protein, RP/EB family, member 2	212307	0.133020724	0.285	0.15	0.018430322	1.25E-06
White Matter Tracts	iTBS	Dnm3	dynamin 3	103967	0.133223871	0.377	0.19	3.71E-06	2.51E-10
White Matter Tracts	iTBS	Gda	guanine deaminase	14544	0.133533429	0.251	0.08	1.60E-09	1.08E-13
White Matter Tracts	iTBS	Rgs10	regulator of G-protein signalling 10	67865	0.133540587	0.401	0.215	3.09E-05	2.09E-09
White Matter Tracts	iTBS	Ybx1	Y box protein 1	22608	0.134024011	0.619	0.413	0.000465951	3.15E-08
White Matter Tracts	iTBS	Hectd4	HECT domain E3 ubiquitin protein ligase 4	269700	0.134584683	0.431	0.253	7.95E-05	5.38E-09
White Matter Tracts	iTBS	Eif3i	eukaryotic translation initiation factor 3, subunit I	54709	0.134772326	0.48	0.275	2.24E-05	1.52E-09
White Matter Tracts	iTBS	Herc1	HECT and RLD domain containing E3 ubiquitin protein ligase family meml	235439	0.134778439	0.361	0.202	0.000996738	6.74E-08
White Matter Tracts	iTBS	Strbp	spermatid perinuclear RNA binding protein	20744	0.135228783	0.338	0.177	0.000298761	2.02E-08
White Matter Tracts	iTBS	Tspyl4	TSPY-like 4	72480	0.135305849	0.339	0.17	4.72E-05	3.19E-09
White Matter Tracts	iTBS	Ssr4	signal sequence receptor, delta	20832	0.135585383	0.496	0.3	0.000272262	1.84E-08
White Matter Tracts	iTBS	Astn1	astrotactin 1	11899	0.136112548	0.435	0.277	0.006271907	4.24E-07
White Matter Tracts	iTBS	Disp2	dispatched RND tramsporter family member 2	214240	0.136507905	0.3	0.137	6.01E-06	4.07E-10
White Matter Tracts	iTBS	Ppp2r2a	protein phosphatase 2, regulatory subunit B, alpha	71978	0.137054894	0.525	0.363	0.008980293	6.08E-07
White Matter Tracts	iTBS	mt-Cytb	NA	NA	0.137413366	1	1	0.013798673	9.34E-07
White Matter Tracts	iTBS	Pet100	PET100 homolog	100503890	0.137630841	0.543	0.358	0.005545063	3.75E-07
White Matter Tracts	iTBS	Adprh	ADP-ribosylarginine hydrolase	11544	0.137907233	0.381	0.215	0.001627521	1.10E-07
White Matter Tracts	iTBS	Setd3	SET domain containing 3	52690	0.1382296	0.574	0.397	0.007795129	5.27E-07
White Matter Tracts	iTBS	Lpgat1	lysophosphatidylglycerol acyltransferase 1	226856	0.138535497	0.339	0.148	3.02E-07	2.05E-11
White Matter Tracts	iTBS	Cope	coatomer protein complex, subunit epsilon	59042	0.138535497	0.529	0.32	3.26E-05	2.21E-09
White Matter Tracts	iTBS	Polr2l	polymerase (RNA) II (DNA directed) polypeptide L	66491	0.139242836	0.513	0.35	0.005944743	4.02E-07
White Matter Tracts	iTBS	Myh10	myosin, heavy polypeptide 10, non-muscle	77579	0.13935808	0.273	0.122	1.65E-05	1.12E-09
White Matter Tracts	iTBS	Dst	dystonin	13518	0.140479372	0.471	0.253	8.78E-06	5.94E-10
White Matter Tracts	iTBS	Cuta	cutA divalent cation tolerance homolog	67675	0.141855856	0.404	0.225	0.000576658	3.90E-08
White Matter Tracts	iTBS	Phyhip	phytanoyl-CoA hydroxylase interacting protein	105653	0.142043572	0.401	0.237	0.000504855	3.42E-08
White Matter Tracts	iTBS	Nrcam	neuronal cell adhesion molecule	319504	0.142963482	0.484	0.328	0.01958101	1.33E-06
White Matter Tracts	iTBS	Eif5	eukaryotic translation initiation factor 5	217869	0.143381894	0.439	0.237	2.96E-06	2.00E-10
White Matter Tracts	iTBS	Ola1	Obg-like ATPase 1	67059	0.144747391	0.372	0.182	2.93E-07	1.98E-11
White Matter Tracts	iTBS	Tafa5	TAFA chemokine like family member 5	106014	0.144862254	0.319	0.132	6.07E-08	4.11E-12
White Matter Tracts	iTBS	Ablim2	actin-binding LIM protein 2	231148	0.144988343	0.226	0.095	0.000154746	1.05E-08
White Matter Tracts	iTBS	Rpl10	ribosomal protein L10	110954	0.14536322	0.973	0.982	0.03599266	2.44E-06
White Matter Tracts	iTBS	Hagh	hydroxyacyl glutathione hydrolase	14651	0.148473734	0.449	0.262	8.43E-05	5.70E-09
White Matter Tracts	iTBS	Trappc6b	trafficking protein particle complex 6B	78232	0.148803399	0.332	0.167	5.32E-05	3.60E-09
White Matter Tracts	iTBS	1700037H04Rik	RIKEN cDNA 1700037H04 gene	67326	0.149023856	0.253	0.092	9.18E-08	6.21E-12
White Matter Tracts	iTBS	Lynx1	Ly6/neurotoxin 1	23936	0.149202308	0.354	0.188	4.84E-05	3.28E-09
White Matter Tracts	iTBS	Fam131a	family with sequence similarity 131, member A	78408	0.149535995	0.421	0.248	0.000115909	7.84E-09
White Matter Tracts	iTBS	Gars	glycyl-tRNA synthetase	353172	0.149891295	0.231	0.083	8.64E-07	5.85E-11
White Matter Tracts	iTBS	Hnrnpul2	heterogeneous nuclear ribonucleoprotein U-like 2	68693	0.150288556	0.318	0.16	0.000264259	1.79E-08
White Matter Tracts	iTBS	Rit2	Ras-like without CAAX 2	19762	0.150946481	0.236	0.088	3.43E-06	2.32E-10
White Matter Tracts	iTBS	Cct4	chaperonin containing Tcp1, subunit 4 (delta)	12464	0.151383528	0.394	0.2	2.47E-06	1.67E-10
White Matter Tracts	iTBS	Chmp5	charged multivesicular body protein 5	76959	0.1516024	0.401	0.22	0.000836798	5.66E-08
White Matter Tracts	iTBS	Cdkn2d	cyclin dependent kinase inhibitor 2D	12581	0.151835074	0.339	0.165	9.07E-06	6.14E-10
White Matter Tracts	iTBS	Arrb1	arrestin, beta 1	109689	0.152275142	0.388	0.228	0.001133648	7.67E-08
White Matter Tracts	iTBS	Trim37	tripartite motif-containing 37	68729	0.154506669	0.433	0.242	1.50E-06	1.01E-10
White Matter Tracts	iTBS	Mycbp2	MYC binding protein 2, E3 ubiquitin protein ligase	105689	0.155614684	0.347	0.162	8.60E-07	5.82E-11
White Matter Tracts	iTBS	Spryd3	SPRY domain containing 3	223918	0.155992968	0.282	0.118	1.40E-06	9.47E-11
White Matter Tracts	iTBS	Ptpra	protein tyrosine phosphatase, receptor type, A	19262	0.156089907	0.551	0.362	0.002039282	1.38E-07
White Matter Tracts	iTBS	Ube2e1	ubiquitin-conjugating enzyme E2E 1	22194	0.156751723	0.478	0.317	0.004999805	3.38E-07
White Matter Tracts	iTBS	Polr2g	polymerase (RNA) II (DNA directed) polypeptide G	67710	0.156949477	0.33	0.162	8.73E-05	5.91E-09
White Matter Tracts	iTBS	Smarcd3	SWI/SNF related, matrix associated, actin dependent regulator of chroma	66993	0.157245979	0.273	0.11	3.39E-07	2.29E-11
White Matter Tracts	iTBS	Madd	MAP-kinase activating death domain	228355	0.157773452	0.426	0.248	2.53E-05	1.71E-09
White Matter Tracts	iTBS	Raly	hnRNP-associated with lethal yellow	19383	0.1577882	0.352	0.183	0.000616583	4.17E-08
White Matter Tracts	iTBS	Ppib	peptidylprolyl isomerase B	19035	0.158244384	0.366	0.182	1.88E-05	1.28E-09
White Matter Tracts	iTBS	Aatk	apoptosis-associated tyrosine kinase	11302	0.159054222	0.704	0.492	0.004730756	3.20E-07
White Matter Tracts	iTBS	Stip1	stress-induced phosphoprotein 1	20867	0.159169109	0.482	0.297	0.000258512	1.75E-08
White Matter Tracts	iTBS	Lin7b	lin-7 homolog B, crumbs cell polarity complex component	22342	0.159416063	0.422	0.277	0.016234577	1.10E-06
White Matter Tracts	iTBS	Sik3	SIK family kinase 3	70661	0.159568444	0.408	0.23	0.000619079	4.19E-08
White Matter Tracts	iTBS	Necap1	NECAP endocytosis associated 1	67602	0.160824347	0.433	0.25	5.80E-05	3.92E-09
White Matter Tracts	iTBS	Baalc	brain and acute leukemia, cytoplasmic	118452	0.160880214	0.44	0.245	2.47E-06	1.67E-10
White Matter Tracts	iTBS	Ddx3x	DEAD box helicase 3, X-linked	13205	0.161042378	0.437	0.222	1.15E-07	7.79E-12
White Matter Tracts	iTBS	Kcnab1	potassium voltage-gated channel, shaker-related subfamily, beta membe	16497	0.161123892	0.325	0.148	3.88E-07	2.63E-11
White Matter Tracts	iTBS	Ctnnd2	catenin (cadherin associated protein), delta 2	18163	0.161262773	0.245	0.097	3.22E-06	2.18E-10
White Matter Tracts	iTBS	Mrpl54	mitochondrial ribosomal protein L54	66047	0.161425265	0.399	0.213	2.21E-05	1.49E-09
White Matter Tracts	iTBS	Rbbp7	retinoblastoma binding protein 7, chromatin remodeling factor	245688	0.161997572	0.271	0.107	2.79E-07	1.89E-11
White Matter Tracts	iTBS	Prkcg	protein kinase C, gamma	18752	0.16218912	0.301	0.142	4.89E-06	3.31E-10
White Matter Tracts	iTBS	Sorl1	sortilin-related receptor, LDLR class A repeats-containing	20660	0.16232265	0.386	0.203	5.82E-06	3.94E-10
White Matter Tracts	iTBS	Sf1	splicing factor 1	22668	0.163022803	0.334	0.138	4.51E-08	3.05E-12
White Matter Tracts	iTBS	Mat2b	methionine adenosyltransferase II, beta	108645	0.163260058	0.421	0.23	2.09E-06	1.41E-10
White Matter Tracts	iTBS	Ier5	immediate early response 5	15939	0.163633345	0.455	0.298	0.002318663	1.57E-07
White Matter Tracts	iTBS	Rpl17	ribosomal protein L17	319195	0.16388795	0.998	0.997	0.001706124	1.15E-07
White Matter Tracts	iTBS	Igfbp6	insulin-like growth factor binding protein 6	16012	0.163986125	0.276	0.118	8.02E-07	5.43E-11
White Matter Tracts	iTBS	Rps14	ribosomal protein S14	20044	0.164294196	1	0.998	0.030014551	2.03E-06
White Matter Tracts	iTBS	Emc6	ER membrane protein complex subunit 6	66048	0.164320043	0.433	0.252	0.000109819	7.43E-09
White Matter Tracts	iTBS	Nptx1	neuronal pentraxin 1	18164	0.164474618	0.236	0.078	2.87E-08	1.94E-12
White Matter Tracts	iTBS	Pnmal1	NA	NA	0.165140362	0.28	0.108	3.74E-08	2.53E-12
White Matter Tracts	iTBS	Igip	IgA inducing protein	109169	0.165702598	0.274	0.107	2.40E-07	1.62E-11
White Matter Tracts	iTBS	Kif5b	kinesin family member 5B	16573	0.16622801	0.5	0.293	0.002233998	1.51E-07
White Matter Tracts	iTBS	Nat14	N-acetyltransferase 14	269854	0.166395302	0.294	0.132	1.72E-06	1.16E-10
White Matter Tracts	iTBS	Ppme1	protein phosphatase methylesterase 1	72590	0.166748812	0.264	0.097	8.58E-08	5.81E-12
White Matter Tracts	iTBS	Cirbp	cold inducible RNA binding protein	12696	0.166859067	0.495	0.313	0.000620011	4.20E-08
White Matter Tracts	iTBS	Cwc15	CWC15 spliceosome-associated protein	66070	0.16828389	0.439	0.218	1.63E-06	1.10E-10
White Matter Tracts	iTBS	Ypel5	yippee like 5	383295	0.168547733	0.448	0.278	0.000729973	4.94E-08
White Matter Tracts	iTBS	AI593442	expressed sequence AI593442	330941	0.168773648	0.285	0.137	4.60E-05	3.11E-09
White Matter Tracts	iTBS	Magee1	MAGE family member E1	107528	0.168981738	0.406	0.228	4.19E-05	2.83E-09
White Matter Tracts	iTBS	Ap1b1	adaptor protein complex AP-1, beta 1 subunit	11764	0.169064171	0.455	0.287	0.000498821	3.38E-08
White Matter Tracts	iTBS	Stx1a	syntaxin 1A (brain)	20907	0.169242312	0.381	0.208	1.06E-05	7.15E-10
White Matter Tracts	iTBS	Mrps12	mitochondrial ribosomal protein S12	24030	0.169283599	0.511	0.35	0.00551951	3.73E-07
White Matter Tracts	iTBS	Dnajc15	DnaJ heat shock protein family (Hsp40) member C15	66148	0.169524308	0.487	0.262	7.55E-08	5.11E-12
White Matter Tracts	iTBS	Celf5	CUGBP, Elav-like family member 5	319586	0.169597805	0.318	0.155	8.96E-06	6.06E-10
White Matter Tracts	iTBS	Taf10	TATA-box binding protein associated factor 10	24075	0.1696751	0.662	0.525	0.041780746	2.83E-06
White Matter Tracts	iTBS	Reep2	receptor accessory protein 2	225362	0.169790783	0.437	0.282	0.001265554	8.56E-08
White Matter Tracts	iTBS	Laptm4b	lysosomal-associated protein transmembrane 4B	114128	0.171407524	0.431	0.232	1.09E-05	7.37E-10
White Matter Tracts	iTBS	Ranbp1	RAN binding protein 1	19385	0.17234073	0.397	0.193	1.28E-07	8.65E-12
White Matter Tracts	iTBS	Pgp	phosphoglycolate phosphatase	67078	0.172636414	0.486	0.297	0.000663347	4.49E-08
White Matter Tracts	iTBS	Spin1	spindlin 1	20729	0.17292818	0.491	0.332	0.000728306	4.93E-08
White Matter Tracts	iTBS	Pfkl	phosphofructokinase, liver, B-type	18641	0.173414461	0.282	0.115	4.02E-07	2.72E-11
White Matter Tracts	iTBS	mt-Atp6	NA	NA	0.173670596	1	1	0.000276388	1.87E-08
White Matter Tracts	iTBS	Nefm	neurofilament, medium polypeptide	18040	0.173970214	0.384	0.213	2.64E-05	1.79E-09
White Matter Tracts	iTBS	Mrpl53	mitochondrial ribosomal protein L53	68499	0.175101296	0.542	0.367	0.001050317	7.11E-08
White Matter Tracts	iTBS	Necab2	N-terminal EF-hand calcium binding protein 2	117148	0.175233335	0.457	0.29	0.000306333	2.07E-08
White Matter Tracts	iTBS	Snrpe	small nuclear ribonucleoprotein E	20643	0.177286188	0.394	0.198	1.13E-06	7.62E-11
White Matter Tracts	iTBS	Cmas	cytidine monophospho-N-acetylneuraminic acid synthetase	12764	0.177334448	0.359	0.18	6.98E-06	4.73E-10
White Matter Tracts	iTBS	Tmem223	transmembrane protein 223	66836	0.177429837	0.426	0.22	1.06E-05	7.18E-10
White Matter Tracts	iTBS	Mrpl48	mitochondrial ribosomal protein L48	52443	0.177580221	0.471	0.287	0.000748181	5.06E-08
White Matter Tracts	iTBS	Camk2g	calcium/calmodulin-dependent protein kinase II gamma	12325	0.17781228	0.291	0.133	9.34E-06	6.32E-10
White Matter Tracts	iTBS	Rusc1	RUN and SH3 domain containing 1	72296	0.18011041	0.245	0.083	1.58E-08	1.07E-12
White Matter Tracts	iTBS	Cdk17	cyclin-dependent kinase 17	237459	0.180402772	0.309	0.132	1.31E-07	8.84E-12
White Matter Tracts	iTBS	Srsf5	serine and arginine-rich splicing factor 5	20384	0.182399549	0.572	0.383	0.000199255	1.35E-08
White Matter Tracts	iTBS	Vps41	VPS41 HOPS complex subunit	218035	0.182444873	0.375	0.187	6.82E-07	4.62E-11
White Matter Tracts	iTBS	Rpl13	ribosomal protein L13	270106	0.182529273	1	1	0.041555533	2.81E-06
White Matter Tracts	iTBS	Csnk1a1	casein kinase 1, alpha 1	93687	0.18272649	0.619	0.44	0.001904821	1.29E-07
White Matter Tracts	iTBS	Lmbrd1	LMBR1 domain containing 1	68421	0.182776765	0.509	0.288	1.24E-07	8.41E-12
White Matter Tracts	iTBS	Mapk9	mitogen-activated protein kinase 9	26420	0.184366914	0.455	0.26	2.65E-06	1.79E-10
White Matter Tracts	iTBS	Ssna1	SS nuclear autoantigen 1	68475	0.184719824	0.412	0.207	3.08E-07	2.08E-11
White Matter Tracts	iTBS	Gabbr2	gamma-aminobutyric acid (GABA) B receptor, 2	242425	0.184881883	0.301	0.125	2.17E-08	1.47E-12
White Matter Tracts	iTBS	Rps5	ribosomal protein S5	20103	0.185417505	0.996	0.993	0.004613704	3.12E-07
White Matter Tracts	iTBS	Dock3	dedicator of cyto-kinesis 3	208869	0.186059676	0.271	0.095	2.19E-09	1.48E-13
White Matter Tracts	iTBS	Gabrd	gamma-aminobutyric acid (GABA) A receptor, subunit delta	14403	0.186095988	0.204	0.052	2.65E-10	1.80E-14
White Matter Tracts	iTBS	Emc7	ER membrane protein complex subunit 7	73024	0.186703811	0.556	0.357	0.000295127	2.00E-08
White Matter Tracts	iTBS	Gabrg2	gamma-aminobutyric acid (GABA) A receptor, subunit gamma 2	14406	0.186903874	0.404	0.213	1.86E-07	1.26E-11
White Matter Tracts	iTBS	Dgkb	diacylglycerol kinase, beta	217480	0.187934953	0.256	0.095	8.32E-08	5.63E-12
White Matter Tracts	iTBS	Mmp17	matrix metallopeptidase 17	23948	0.188657657	0.283	0.113	2.23E-08	1.51E-12
White Matter Tracts	iTBS	Cadps	Ca2+-dependent secretion activator	27062	0.188668931	0.305	0.13	5.09E-08	3.44E-12
White Matter Tracts	iTBS	Rbis	ribosomal biogenesis factor	69126	0.190074673	0.664	0.527	0.01145952	7.75E-07
White Matter Tracts	iTBS	Icam5	intercellular adhesion molecule 5, telencephalin	15898	0.190396615	0.392	0.212	2.78E-06	1.88E-10
White Matter Tracts	iTBS	Psmd14	proteasome (prosome, macropain) 26S subunit, non-ATPase, 14	59029	0.190976639	0.329	0.135	1.13E-08	7.68E-13
White Matter Tracts	iTBS	Ppa1	pyrophosphatase (inorganic) 1	67895	0.191270014	0.329	0.172	0.000158302	1.07E-08
White Matter Tracts	iTBS	Mrps18a	mitochondrial ribosomal protein S18A	68565	0.19151733	0.464	0.267	3.30E-06	2.23E-10
White Matter Tracts	iTBS	Eif3g	eukaryotic translation initiation factor 3, subunit G	53356	0.191729372	0.294	0.142	0.000126723	8.58E-09
White Matter Tracts	iTBS	Mettl9	methyltransferase like 9	59052	0.191755584	0.404	0.213	1.45E-05	9.83E-10
White Matter Tracts	iTBS	Nucks1	nuclear casein kinase and cyclin-dependent kinase substrate 1	98415	0.191915506	0.525	0.338	0.000330606	2.24E-08
White Matter Tracts	iTBS	Spcs2	signal peptidase complex subunit 2 homolog (S. cerevisiae)	66624	0.192481028	0.458	0.237	1.61E-07	1.09E-11
White Matter Tracts	iTBS	Kcnj4	potassium inwardly-rectifying channel, subfamily J, member 4	16520	0.19254194	0.289	0.122	1.45E-07	9.78E-12
White Matter Tracts	iTBS	Vxn	vexin	76982	0.192669645	0.314	0.15	8.18E-06	5.53E-10
White Matter Tracts	iTBS	mt-Co3	NA	NA	0.193365685	1	1	1.53E-05	1.04E-09
White Matter Tracts	iTBS	mt-Co1	NA	NA	0.1953381	1	1	2.66E-06	1.80E-10
White Matter Tracts	iTBS	Dnm1l	dynamin 1-like	74006	0.195449941	0.413	0.225	7.39E-07	5.00E-11
White Matter Tracts	iTBS	Nbea	neurobeachin	26422	0.196376627	0.245	0.082	7.09E-09	4.80E-13
White Matter Tracts	iTBS	mt-Nd1	NA	NA	0.19656468	1	1	5.76E-05	3.90E-09
White Matter Tracts	iTBS	Rpn1	ribophorin I	103963	0.197994527	0.466	0.283	0.000215313	1.46E-08
White Matter Tracts	iTBS	Bola3	bolA-like 3 (E. coli)	78653	0.201455583	0.572	0.352	1.98E-06	1.34E-10
White Matter Tracts	iTBS	Txndc15	thioredoxin domain containing 15	69672	0.202001468	0.374	0.185	1.30E-06	8.78E-11

White Matter Tracts	iTBS	Stard10	START domain containing 10	56018	0.204303843	0.278	0.117	9.01E-07	6.10E-11
White Matter Tracts	iTBS	Lysmd2	LysM, putative peptidoglycan-binding, domain containing 2	70082	0.206707	0.325	0.128	1.06E-09	7.19E-14
White Matter Tracts	iTBS	Rell2	RELT-like 2	225392	0.207941218	0.336	0.152	1.09E-07	7.37E-12
White Matter Tracts	iTBS	Dlgap1	DLG associated protein 1	224997	0.207959641	0.451	0.263	1.44E-06	9.76E-11
White Matter Tracts	iTBS	Rpl35	ribosomal protein L35	66489	0.208400153	0.996	1	1.58E-05	1.07E-09
White Matter Tracts	iTBS	Fam49a	NA	NA	0.208689575	0.334	0.153	7.90E-08	5.35E-12
White Matter Tracts	iTBS	Dpm3	dolichyl-phosphate mannosyltransferase polypeptide 3	68563	0.212447956	0.323	0.17	0.000491385	3.33E-08
White Matter Tracts	iTBS	Rpl37	ribosomal protein L37	67281	0.212887212	1	1	0.014871922	1.01E-06
White Matter Tracts	iTBS	Ogt	O-linked N-acetylglucosamine (GlcNAc) transferase (UDP-N-acetylglucosa	108155	0.214371729	0.413	0.192	1.86E-09	1.26E-13
White Matter Tracts	iTBS	H3f3b	H3.3 histone B	15081	0.215215595	0.94	0.948	0.006800937	4.60E-07
White Matter Tracts	iTBS	Ube2l3	ubiquitin-conjugating enzyme E2L 3	22195	0.215415145	0.574	0.383	0.000229894	1.56E-08
White Matter Tracts	iTBS	Ssbp4	single stranded DNA binding protein 4	76900	0.217127796	0.415	0.22	1.18E-07	7.97E-12
White Matter Tracts	iTBS	Polr2i	polymerase (RNA) II (DNA directed) polypeptide I	69920	0.217591435	0.348	0.142	3.79E-09	2.56E-13
White Matter Tracts	iTBS	Pin4	peptidyl-prolyl cis/trans isomerase, NIMA-interacting, 4 (parvulin)	69713	0.217629176	0.426	0.215	1.20E-07	8.13E-12
White Matter Tracts	iTBS	Rps27a	ribosomal protein S27A	78294	0.221362634	1	1	0.000273954	1.85E-08
White Matter Tracts	iTBS	Ndufaf8	NADH:ubiquinone oxidoreductase complex assembly factor 8	208501	0.221457001	0.345	0.142	6.48E-09	4.39E-13
White Matter Tracts	iTBS	Rpl34	ribosomal protein L34	68436	0.222874321	1	1	0.030868616	2.09E-06
White Matter Tracts	iTBS	Snrpb	small nuclear ribonucleoprotein B	20638	0.226359858	0.451	0.238	6.36E-07	4.31E-11
White Matter Tracts	iTBS	Araf	Araf proto-oncogene, serine/threonine kinase	11836	0.227405225	0.316	0.13	2.02E-08	1.36E-12
White Matter Tracts	iTBS	Rpl39	ribosomal protein L39	67248	0.2274344	0.998	0.998	0.01107004	7.49E-07
White Matter Tracts	iTBS	Arhgef17	Rho guanine nucleotide exchange factor (GEF) 17	207212	0.2294092	0.267	0.09	3.02E-10	2.04E-14
White Matter Tracts	iTBS	Rps8	ribosomal protein S8	20116	0.231711363	1	1	4.87E-05	3.30E-09
White Matter Tracts	iTBS	Krtcap2	keratinocyte associated protein 2	66059	0.231751695	0.545	0.362	0.000478247	3.24E-08
White Matter Tracts	iTBS	Polr2j	polymerase (RNA) II (DNA directed) polypeptide J	20022	0.232463332	0.31	0.12	5.44E-09	3.68E-13
White Matter Tracts	iTBS	Tmem179	transmembrane protein 179	104885	0.232612844	0.392	0.217	6.55E-06	4.43E-10
White Matter Tracts	iTBS	Tceal9	transcription elongation factor A like 9	22381	0.23551853	0.442	0.223	5.37E-10	3.64E-14
White Matter Tracts	iTBS	Rpl9	ribosomal protein L9	20005	0.235973109	1	0.998	3.12E-05	2.11E-09
White Matter Tracts	iTBS	Pdp1	pyruvate dehydrogenase phosphatase catalytic subunit 1	381511	0.236282519	0.323	0.127	6.58E-11	4.45E-15
White Matter Tracts	iTBS	Lgmn	legumain	19141	0.236652584	0.625	0.405	3.13E-06	2.12E-10
White Matter Tracts	iTBS	Tmbim6	transmembrane BAX inhibitor motif containing 6	110213	0.236939758	0.431	0.223	4.41E-06	2.98E-10
White Matter Tracts	iTBS	Rps26	ribosomal protein S26	27370	0.237632036	0.996	0.997	0.000484328	3.28E-08
White Matter Tracts	iTBS	Commd3	COMM domain containing 3	12238	0.241499887	0.399	0.185	5.69E-09	3.85E-13
White Matter Tracts	iTBS	Psma4	proteasome subunit alpha 4	26441	0.243990046	0.37	0.153	4.09E-10	2.77E-14
White Matter Tracts	iTBS	Sec61b	Sec61 beta subunit	66212	0.248009655	0.532	0.297	2.11E-09	1.43E-13
White Matter Tracts	iTBS	Cdk16	cyclin-dependent kinase 16	18555	0.248600197	0.493	0.283	8.07E-08	5.46E-12
White Matter Tracts	iTBS	Rps12	ribosomal protein S12	20042	0.250664118	1	0.998	0.000138898	9.40E-09
White Matter Tracts	iTBS	Banf1	BAF nuclear assembly factor 1	23825	0.252885736	0.493	0.295	1.16E-05	7.82E-10
White Matter Tracts	iTBS	Cd47	CD47 antigen (Rh-related antigen, integrin-associated signal transducer)	16423	0.25602735	0.319	0.128	8.88E-09	6.01E-13
White Matter Tracts	iTBS	Cct3	chaperonin containing Tcp1, subunit 3 (gamma)	12462	0.257764942	0.439	0.213	8.20E-10	5.55E-14
White Matter Tracts	iTBS	Rpl30	ribosomal protein L30	19946	0.267108506	1	1	9.13E-05	6.18E-09
White Matter Tracts	iTBS	Hexb	hexosaminidase B	15212	0.26767016	0.44	0.222	3.43E-07	2.32E-11
White Matter Tracts	iTBS	Rpl23	ribosomal protein L23	65019	0.26928313	1	1	8.69E-08	5.88E-12
White Matter Tracts	iTBS	Cab39	calcium binding protein 39	12283	0.269462269	0.307	0.108	4.10E-11	2.78E-15
White Matter Tracts	iTBS	Crym	crystallin, mu	12971	0.270578122	0.197	0.053	4.40E-09	2.98E-13
White Matter Tracts	iTBS	Sf3b1	splicing factor 3b, subunit 1	81898	0.273697497	0.384	0.173	1.53E-09	1.03E-13
White Matter Tracts	iTBS	Rpl35a	ribosomal protein L35A	57808	0.274475287	1	1	2.00E-05	1.35E-09
White Matter Tracts	iTBS	Rps10	ribosomal protein S10	67097	0.274498159	0.998	1	6.58E-06	4.46E-10
White Matter Tracts	iTBS	Enpp2	ectonucleotide pyrophosphatase/phosphodiesterase 2	18606	0.282580977	0.666	0.468	0.012315097	8.33E-07
White Matter Tracts	iTBS	mt-Co2	NA	NA	0.296882088	1	1	8.97E-06	6.07E-10
White Matter Tracts	iTBS	Snhg8	small nucleolar RNA host gene 8	69895	0.308124984	0.5	0.237	6.81E-12	4.61E-16
White Matter Tracts	iTBS	Rps24	ribosomal protein S24	20088	0.312858966	1	1	1.35E-08	9.11E-13
White Matter Tracts	iTBS	Rps21	ribosomal protein S21	66481	0.327145778	1	1	3.90E-11	2.64E-15
White Matter Tracts	iTBS	Rps28	ribosomal protein S28	54127	0.334807146	0.987	0.995	1.79E-06	1.21E-10
White Matter Tracts	iTBS	Gstp1	glutathione S-transferase, pi 1	14870	0.538358018	0.825	0.597	1.39E-12	9.40E-17
White Matter Tracts	iTBS	Uba52	ubiquitin A-52 residue ribosomal protein fusion product 1	22186	0.57659486	0.991	0.958	1.98E-23	1.34E-27
White Matter Tracts	iTBS	Ttr	transthyretin	22139	0.609096865	0.3	0.078	3.26E-17	2.21E-21
White Matter Tracts	iTBS	Gm10076	ribosomal protein L41 pseudogene	100126819	0.847062151	0.975	0.887	2.48E-46	1.68E-50
Caudoputamen	iTBS	Dnm1	dynamin 1	13429	-0.303727691	0.849	0.944	1.04E-13	7.05E-18
Caudoputamen	iTBS	Hpcal4	hippocalcin-like 4	170638	-0.289905788	0.525	0.596	1.98E-06	1.34E-10
Caudoputamen	iTBS	Bc1	brain cytoplasmic RNA 1	100568459	-0.284200548	0.537	0.693	1.03E-08	6.97E-13
Caudoputamen	iTBS	Vsnl1	visinin-like 1	26950	-0.281697653	0.333	0.544	1.47E-17	9.96E-22
Caudoputamen	iTBS	Gm42418	NA	NA	-0.277445049	0.926	0.963	1.13E-10	7.63E-15
Caudoputamen	iTBS	Glul	glutamate-ammonia ligase (glutamine synthetase)	14645	-0.266010832	0.738	0.835	5.56E-09	3.77E-13
Caudoputamen	iTBS	Olfm1	olfactomedin 1	56177	-0.262932448	0.6	0.764	1.48E-12	1.00E-16
Caudoputamen	iTBS	Tuba1b	tubulin, alpha 1B	22143	-0.256390985	0.671	0.833	1.34E-11	9.08E-16
Caudoputamen	iTBS	Sod1	superoxide dismutase 1, soluble	20655	-0.246012289	0.749	0.812	2.35E-07	1.59E-11
Caudoputamen	iTBS	Cplx2	complexin 2	12890	-0.244154702	0.878	0.899	1.49E-05	1.01E-09
Caudoputamen	iTBS	Tubb4b	tubulin, beta 4B class IVB	227613	-0.233490235	0.538	0.625	3.16E-06	2.14E-10
Caudoputamen	iTBS	Cdk5r2	cyclin-dependent kinase 5, regulatory subunit 2 (p39)	12570	-0.230927658	0.335	0.433	8.57E-05	5.80E-09
Caudoputamen	iTBS	Kif1a	kinesin family member 1A	16560	-0.227441191	0.639	0.695	0.000421954	2.86E-08
Caudoputamen	iTBS	Atp1a3	ATPase, Na+/K+ transporting, alpha 3 polypeptide	232975	-0.219844408	0.66	0.733	1.78E-05	1.20E-09
Caudoputamen	iTBS	Ivns1abp	influenza virus NS1A binding protein	117198	-0.216426233	0.909	0.876	0.03353135	2.27E-06
Caudoputamen	iTBS	Slc17a7	solute carrier family 17 (sodium-dependent inorganic phosphate cotrans¦	72961	-0.216358944	0.217	0.361	2.65E-09	1.79E-13
Caudoputamen	iTBS	Tubb2a	tubulin, beta 2A class IIA	22151	-0.214976534	0.582	0.713	2.56E-06	1.73E-10
Caudoputamen	iTBS	Rab6b	RAB6B, member RAS oncogene family	270192	-0.210451714	0.879	0.917	1.14E-07	7.72E-12
Caudoputamen	iTBS	Tuba1a	tubulin, alpha 1A	22142	-0.209871779	0.948	0.984	4.35E-09	2.94E-13
Caudoputamen	iTBS	Slc1a2	solute carrier family 1 (glial high affinity glutamate transporter), member	20511	-0.209030992	0.975	0.991	5.08E-08	3.44E-12
Caudoputamen	iTBS	Acot7	acyl-CoA thioesterase 7	70025	-0.20437813	0.597	0.641	0.019396537	1.31E-06
Caudoputamen	iTBS	Atp1a1	ATPase, Na+/K+ transporting, alpha 1 polypeptide	11928	-0.201915106	0.886	0.954	9.56E-06	6.47E-10
Caudoputamen	iTBS	Tuba4a	tubulin, alpha 4A	22145	-0.199776266	0.878	0.915	3.98E-05	2.69E-09
Caudoputamen	iTBS	Aldoc	aldolase C, fructose-bisphosphate	11676	-0.199715765	0.981	0.997	5.30E-08	3.59E-12
Caudoputamen	iTBS	Stxbp1	syntaxin binding protein 1	20910	-0.199575621	0.683	0.782	2.49E-05	1.69E-09
Caudoputamen	iTBS	Gng3	guanine nucleotide binding protein (G protein), gamma 3	14704	-0.197950328	0.762	0.857	1.22E-05	8.29E-10
Caudoputamen	iTBS	Atp5o	ATP synthase, H+ transporting, mitochondrial F1 complex, O subunit	28080	-0.197903568	0.926	0.951	5.17E-05	3.50E-09
Caudoputamen	iTBS	Syn1	synapsin I	20964	-0.197031388	0.614	0.669	0.022272264	1.51E-06
Caudoputamen	iTBS	Kif5a	kinesin family member 5A	16572	-0.195500376	0.753	0.84	0.000176307	1.19E-08
Caudoputamen	iTBS	Stmn1	stathmin 1	16765	-0.193601561	0.628	0.772	1.30E-05	8.82E-10
Caudoputamen	iTBS	Gnas	GNAS (guanine nucleotide binding protein, alpha stimulating) complex lo	14683	-0.192941582	0.786	0.9	8.15E-07	5.52E-11
Caudoputamen	iTBS	Prkar1b	protein kinase, cAMP dependent regulatory, type I beta	19085	-0.191330678	0.616	0.728	2.23E-05	1.51E-09
Caudoputamen	iTBS	Eef1a2	eukaryotic translation elongation factor 1 alpha 2	13628	-0.191160339	0.97	0.988	0.009589435	6.49E-07
Caudoputamen	iTBS	Syp	synaptophysin	20977	-0.189262044	0.551	0.642	0.006419589	4.34E-07
Caudoputamen	iTBS	Actb	actin, beta	11461	-0.18789546	0.999	1	2.02E-06	1.36E-10
Caudoputamen	iTBS	Epn1	epsin 1	13854	-0.186174956	0.68	0.705	0.013616759	9.21E-07
Caudoputamen	iTBS	Apbb1	amyloid beta (A4) precursor protein-binding, family B, member 1	11785	-0.183486734	0.738	0.827	0.000569994	3.86E-08
Caudoputamen	iTBS	Ywhag	tyrosine 3-monooxygenase/tryptophan 5-monooxygenase activation pro	22628	-0.183181764	0.756	0.844	0.000435323	2.95E-08
Caudoputamen	iTBS	Sptbn2	spectrin beta, non-erythrocytic 2	20743	-0.181517182	0.707	0.734	0.042016382	2.84E-06
Caudoputamen	iTBS	Eef2	eukaryotic translation elongation factor 2	13629	-0.179894337	0.95	0.979	0.000115754	7.83E-09
Caudoputamen	iTBS	Ikbkb	inhibitor of kappaB kinase beta	16150	-0.179439433	0.225	0.32	0.001910955	1.29E-07
Caudoputamen	iTBS	Cpe	carboxypeptidase E	12876	-0.171715894	1	1	1.88E-09	1.27E-13
Caudoputamen	iTBS	Ndrg2	N-myc downstream regulated gene 2	29811	-0.166889928	0.891	0.932	0.001434138	9.70E-08
Caudoputamen	iTBS	Clu	clusterin	12759	-0.161061623	0.982	0.993	0.010232243	6.92E-07
Caudoputamen	iTBS	Tcf25	transcription factor 25 (basic helix-loop-helix)	66855	-0.159615308	0.848	0.894	0.012521108	8.47E-07
Caudoputamen	iTBS	Vamp2	vesicle-associated membrane protein 2	22318	-0.156580317	0.972	0.988	0.000270976	1.83E-08
Caudoputamen	iTBS	Ywhah	tyrosine 3-monooxygenase/tryptophan 5-monooxygenase activation pro	22629	-0.155143131	0.915	0.958	0.004849813	3.28E-07
Caudoputamen	iTBS	Rpl5	ribosomal protein L5	100503670	-0.154968169	0.945	0.962	0.022043917	1.49E-06
Caudoputamen	iTBS	Thy1	thymus cell antigen 1, theta	21838	-0.150630978	0.897	0.947	0.015265346	1.03E-06
Caudoputamen	iTBS	Snhg9	small nucleolar RNA host gene 9	73474	-0.149087391	0.147	0.252	4.39E-05	2.97E-09
Caudoputamen	iTBS	Hspa8	heat shock protein 8	15481	-0.137501767	1	1	4.21E-05	2.85E-09
Caudoputamen	iTBS	Rpl4	ribosomal protein L4	67891	-0.136803021	0.986	0.996	0.021725848	1.47E-06
Caudoputamen	iTBS	Rpl41	ribosomal protein L41	67945	-0.124300787	1	1	2.32E-09	1.57E-13
Caudoputamen	iTBS	Cck	cholecystokinin	12424	-0.123452561	0.308	0.416	0.028189879	1.91E-06
Caudoputamen	iTBS	Ywhaz	tyrosine 3-monooxygenase/tryptophan 5-monooxygenase activation pro	22631	-0.122034389	1	0.997	0.000760287	5.14E-08
Caudoputamen	iTBS	Aldoa	aldolase A, fructose-bisphosphate	11674	-0.109230356	0.999	1	0.014494378	9.81E-07
Caudoputamen	iTBS	Ndrg4	N-myc downstream regulated gene 4	234593	-0.108903091	0.999	0.999	0.037846313	2.56E-06
Caudoputamen	iTBS	Gapdh	glyceraldehyde-3-phosphate dehydrogenase	14433	-0.099323276	1	1	0.000161654	1.09E-08
Caudoputamen	iTBS	Zfp763	zinc finger protein 763	73451	0.077897838	0.102	0.046	0.026290066	1.78E-06
Caudoputamen	iTBS	Hmga1	high mobility group AT-hook 1	15361	0.078127536	0.114	0.053	0.01167317	7.90E-07
Caudoputamen	iTBS	Ddost	dolichyl-di-phosphooligosaccharide-protein glycotransferase	13200	0.082293064	0.509	0.373	0.045826336	3.10E-06
Caudoputamen	iTBS	Cpne2	copine II	234577	0.083188673	0.126	0.065	0.046409945	3.14E-06
Caudoputamen	iTBS	Cog6	component of oligomeric golgi complex 6	67542	0.08523283	0.192	0.115	0.03397837	2.30E-06
Caudoputamen	iTBS	Ahcy	S-adenosylhomocysteine hydrolase	269378	0.086149993	0.103	0.037	8.65E-05	5.85E-09
Caudoputamen	iTBS	Golga4	golgi autoantigen, golgin subfamily a, 4	54214	0.087169102	0.235	0.152	0.043637274	2.95E-06
Caudoputamen	iTBS	Coro1b	coronin, actin binding protein 1B	23789	0.089192604	0.1	0.035	0.000112076	7.58E-09
Caudoputamen	iTBS	P4ha1	procollagen-proline, 2-oxoglutarate 4-dioxygenase (proline 4-hydroxylase	18451	0.090141434	0.144	0.079	0.042829052	2.90E-06
Caudoputamen	iTBS	Tipin	timeless interacting protein	66131	0.090544929	0.138	0.073	0.022986409	1.56E-06
Caudoputamen	iTBS	Anapc10	anaphase promoting complex subunit 10	68999	0.091669227	0.135	0.063	0.0008655	5.86E-08
Caudoputamen	iTBS	Gpr139	G protein-coupled receptor 139	209776	0.092920896	0.12	0.052	0.000842863	5.70E-08
Caudoputamen	iTBS	Cdh9	cadherin 9	12565	0.095266466	0.167	0.095	0.027263545	1.84E-06
Caudoputamen	iTBS	Isca2	iron-sulfur cluster assembly 2	74316	0.096919967	0.334	0.232	0.041702368	2.82E-06
Caudoputamen	iTBS	Smad3	SMAD family member 3	17127	0.098166277	0.228	0.139	0.006798992	4.60E-07
Caudoputamen	iTBS	Ank	progressive ankylosis	11732	0.098238902	0.356	0.254	0.0430586	2.91E-06
Caudoputamen	iTBS	Fndc10	fibronectin type III domain containing 10	230991	0.098277046	0.177	0.1	0.009143197	6.19E-07
Caudoputamen	iTBS	Ppia	peptidylprolyl isomerase A	268373	0.098815002	1	1	0.003784163	2.56E-07
Caudoputamen	iTBS	Atp5l	ATP synthase, H+ transporting, mitochondrial F0 complex, subunit G	27425	0.100010126	0.999	1	0.039733504	2.69E-06
Caudoputamen	iTBS	Ppp5c	protein phosphatase 5, catalytic subunit	19060	0.100195099	0.307	0.195	0.002120992	1.44E-07
Caudoputamen	iTBS	Ankrd37	ankyrin repeat domain 37	654824	0.100295449	0.243	0.15	0.005886759	3.98E-07
Caudoputamen	iTBS	Galnt14	polypeptide N-acetylgalactosaminyltransferase 14	71685	0.100303405	0.162	0.085	0.001655126	1.12E-07
Caudoputamen	iTBS	Eif2b2	eukaryotic translation initiation factor 2B, subunit 2 beta	217715	0.100397824	0.226	0.142	0.019842673	1.34E-06
Caudoputamen	iTBS	Rarb	retinoic acid receptor, beta	218772	0.100676302	0.274	0.184	0.047086253	3.19E-06
Caudoputamen	iTBS	Abhd11	abhydrolase domain containing 11	68758	0.101265268	0.171	0.092	0.002224255	1.51E-07
Caudoputamen	iTBS	Gm19410	predicted gene, 19410	100502846	0.101655422	0.194	0.115	0.017292213	1.17E-06
Caudoputamen	iTBS	Akap9	A kinase (PRKA) anchor protein (yotiao) 9	100986	0.101860451	0.42	0.308	0.03987716	2.70E-06
Caudoputamen	iTBS	Rplp1	ribosomal protein, large, P1	56040	0.102400525	1	1	0.002393604	1.62E-07
Caudoputamen	iTBS	Rab10os	RAB10, member RAS oncogene family, opposite strand	74173	0.102769042	0.165	0.095	0.032747711	2.22E-06
Caudoputamen	iTBS	Tiam2	T cell lymphoma invasion and metastasis 2	24001	0.103161602	0.4	0.29	0.037208161	2.52E-06
Caudoputamen	iTBS	Plvap	plasmalemma vesicle associated protein	84094	0.10372679	0.165	0.093	0.014556594	9.85E-07
Caudoputamen	iTBS	Rpl21	ribosomal protein L21	19933	0.103985966	0.999	1	0.000693031	4.69E-08
Caudoputamen	iTBS	Unc5a	unc-5 netrin receptor A	107448	0.104156456	0.221	0.14	0.040569179	2.75E-06
Caudoputamen	iTBS	Il17rc	interleukin 17 receptor C	171095	0.104657102	0.156	0.076	0.00031268	2.12E-08
Caudoputamen	iTBS	Tgfa	transforming growth factor alpha	21802	0.104886794	0.495	0.366	0.009912524	6.71E-07
Caudoputamen	iTBS	Zbtb8os	zinc finger and BTB domain containing 8 opposite strand	67106	0.105259371	0.282	0.192	0.04892575	3.31E-06
Caudoputamen	iTBS	Psme2	proteasome (prosome, macropain) activator subunit 2 (PA28 beta)	19188	0.105583709	0.211	0.13	0.018245344	1.23E-06
Caudoputamen	iTBS	Ptrhd1	peptidyl-tRNA hydrolase domain containing 1	69709	0.106508872	0.284	0.19	0.030893527	2.09E-06
Caudoputamen	iTBS	Lrtm2	leucine-rich repeats and transmembrane domains 2	211187	0.106780954	0.368	0.263	0.025091092	1.70E-06
Caudoputamen	iTBS	Tmem80	transmembrane protein 80	71448	0.106816712	0.152	0.08	0.004751909	3.22E-07
Caudoputamen	iTBS	Ddx42	DEAD box helicase 42	72047	0.107261537	0.246	0.156	0.01257811	8.51E-07
Caudoputamen	iTBS	Dnal4	dynein, axonemal, light chain 4	54152	0.107574629	0.414	0.306	0.034529875	2.34E-06
Caudoputamen	iTBS	Plekhj1	pleckstrin homology domain containing, family J member 1	78670	0.107576027	0.335	0.227	0.006370798	4.31E-07
Caudoputamen	iTBS	Gadd45b	growth arrest and DNA-damage-inducible 45 beta	17873	0.107930747	0.193	0.113	0.007339985	4.97E-07
Caudoputamen	iTBS	Tpgs1	tubulin polyglutamylase complex subunit 1	110012	0.108673417	0.409	0.296	0.024006571	1.62E-06
Caudoputamen	iTBS	Klf9	Kruppel-like factor 9	16601	0.108695548	0.537	0.409	0.042480767	2.87E-06
Caudoputamen	iTBS	Mbd2	methyl-CpG binding domain protein 2	17191	0.108892608	0.34	0.233	0.01345581	9.11E-07
Caudoputamen	iTBS	Zfp580	zinc finger protein 580	68992	0.109174152	0.269	0.178	0.022031222	1.49E-06
Caudoputamen	iTBS	Sema7a	sema domain, immunoglobulin domain (Ig), and GPI membrane anchor, (	20361	0.109389318	0.187	0.1	0.000631621	4.27E-08
Caudoputamen	iTBS	Bckdk	branched chain ketoacid dehydrogenase kinase	12041	0.109529065	0.241	0.152	0.008726518	5.91E-07
Caudoputamen	iTBS	Slc25a33	solute carrier family 25, member 33	70556	0.109972859	0.448	0.333	0.04846185	3.28E-06
Caudoputamen	iTBS	Acbd4	acyl-Coenzyme A binding domain containing 4	67131	0.110255917	0.222	0.132	0.001893859	1.28E-07
Caudoputamen	iTBS	Son	Son DNA binding protein	20658	0.110260894	0.512	0.38	0.016601263	1.12E-06
Caudoputamen	iTBS	Cox6a1	cytochrome c oxidase subunit 6A1	12861	0.110352573	0.999	0.999	0.035053713	2.37E-06
Caudoputamen	iTBS	Rp9	retinitis pigmentosa 9 (human)	55934	0.11039729	0.233	0.144	0.005583884	3.78E-07
Caudoputamen	iTBS	Atg3	autophagy related 3	67841	0.110412103	0.243	0.154	0.009818819	6.64E-07
Caudoputamen	iTBS	Epdr1	ependymin related protein 1 (zebrafish)	105298	0.111502674	0.5	0.37	0.01652626	1.12E-06
Caudoputamen	iTBS	Eef1a1	eukaryotic translation elongation factor 1 alpha 1	13627	0.111781941	1	1	5.57E-05	3.77E-09
Caudoputamen	iTBS	Rps11	ribosomal protein S11	27207	0.111797549	0.986	0.989	0.011380849	7.70E-07
Caudoputamen	iTBS	Tmco1	transmembrane and coiled-coil domains 1	68944	0.111966261	0.317	0.21	0.005086142	3.44E-07
Caudoputamen	iTBS	Mrpl36	mitochondrial ribosomal protein L36	94066	0.112548952	0.376	0.27	0.013791998	9.33E-07
Caudoputamen	iTBS	Osbpl2	oxysterol binding protein-like 2	228983	0.112912137	0.274	0.177	0.006278747	4.25E-07
Caudoputamen	iTBS	Rps5	ribosomal protein S5	20103	0.113006038	0.969	0.957	0.008831814	5.98E-07
Caudoputamen	iTBS	Hnrnpm	heterogeneous nuclear ribonucleoprotein M	76936	0.113532446	0.526	0.379	0.002082768	1.41E-07
Caudoputamen	iTBS	Adgrb3	adhesion G protein-coupled receptor B3	210933	0.113802631	0.368	0.256	0.00867006	5.87E-07
Caudoputamen	iTBS	Mettl23	methyltransferase like 23	74319	0.113877869	0.332	0.226	0.009557195	6.47E-07
Caudoputamen	iTBS	Tmem30a	transmembrane protein 30A	69981	0.113963254	0.616	0.477	0.037234799	2.52E-06
Caudoputamen	iTBS	Ckb	creatine kinase, brain	12709	0.115592579	1	1	1.17E-07	7.95E-12
Caudoputamen	iTBS	Dctn6	dynactin 6	22428	0.115673336	0.6	0.463	0.036378754	2.46E-06
Caudoputamen	iTBS	Selenos	selenoprotein S	109815	0.116648163	0.314	0.215	0.007837009	5.30E-07
Caudoputamen	iTBS	Acy1	aminoacylase 1	109652	0.116648936	0.341	0.229	0.002970014	2.01E-07
Caudoputamen	iTBS	Skap2	src family associated phosphoprotein 2	54353	0.117718353	0.193	0.1	6.28E-05	4.25E-09
Caudoputamen	iTBS	Lingo3	leucine rich repeat and Ig domain containing 3	237403	0.118744235	0.497	0.385	0.028074716	1.90E-06
Caudoputamen	iTBS	Fam174a	family with sequence similarity 174, member A	67698	0.118910595	0.43	0.317	0.021849317	1.48E-06
Caudoputamen	iTBS	Chd9	chromodomain helicase DNA binding protein 9	109151	0.119514738	0.235	0.148	0.008978984	6.08E-07
Caudoputamen	iTBS	Gcnt2	glucosaminyl (N-acetyl) transferase 2, I-branching enzyme	14538	0.119575922	0.421	0.309	0.013918204	9.42E-07
Caudoputamen	iTBS	Tomm5	translocase of outer mitochondrial membrane 5	68512	0.1202974	0.54	0.405	0.024975071	1.69E-06
Caudoputamen	iTBS	Denr	density-regulated protein	68184	0.120550652	0.492	0.369	0.00785197	5.31E-07
Caudoputamen	iTBS	Slc6a6	solute carrier family 6 (neurotransmitter transporter, taurine), member 6	21366	0.120702391	0.347	0.242	0.013368328	9.05E-07
Caudoputamen	iTBS	Isca1	iron-sulfur cluster assembly 1	69046	0.121411584	0.444	0.334	0.037742048	2.55E-06
Caudoputamen	iTBS	Opalin	oligodendrocytic myelin paranodal and inner loop protein	226115	0.121649235	0.3	0.205	0.030735762	2.08E-06
Caudoputamen	iTBS	Cox5b	cytochrome c oxidase subunit 5B	12859	0.122580722	1	0.999	0.000282201	1.91E-08
Caudoputamen	iTBS	Sdhaf4	succinate dehydrogenase complex assembly factor 4	68002	0.122626145	0.319	0.206	0.00040243	2.72E-08
Caudoputamen	iTBS	Leprot	leptin receptor overlapping transcript	230514	0.122762855	0.199	0.115	0.002333345	1.58E-07
Caudoputamen	iTBS	Spint2	serine protease inhibitor, Kunitz type 2	20733	0.123199722	0.254	0.157	0.001771142	1.20E-07
Caudoputamen	iTBS	Lsm4	LSM4 homolog, U6 small nuclear RNA and mRNA degradation associated	50783	0.123472387	0.485	0.359	0.008412661	5.69E-07
Caudoputamen	iTBS	Jkamp	JNK1/MAPK8-associated membrane protein	104771	0.123817822	0.31	0.2	0.000904098	6.12E-08
Caudoputamen	iTBS	Fut8	fucosyltransferase 8	53618	0.124022148	0.245	0.151	0.002132619	1.44E-07
Caudoputamen	iTBS	Chga	chromogranin A	12652	0.124058929	0.379	0.265	0.012718576	8.61E-07
Caudoputamen	iTBS	Rps14	ribosomal protein S14	20044	0.12412127	0.981	0.975	0.011813394	7.99E-07
Caudoputamen	iTBS	Chchd10	coiled-coil-helix-coiled-coil-helix domain containing 10	103172	0.124584542	1	0.999	0.000185462	1.25E-08
Caudoputamen	iTBS	Mrpl18	mitochondrial ribosomal protein L18	67681	0.124748832	0.456	0.341	0.012569862	8.51E-07
Caudoputamen	iTBS	Arpp21	cyclic AMP-regulated phosphoprotein, 21	74100	0.125165285	0.988	0.97	0.010706689	7.25E-07
Caudoputamen	iTBS	Cox4i1	cytochrome c oxidase subunit 4I1	12857	0.125278231	1	1	5.94E-06	4.02E-10
Caudoputamen	iTBS	Elk1	ELK1, member of ETS oncogene family	13712	0.12666731	0.303	0.193	0.000343117	2.32E-08
Caudoputamen	iTBS	Spg21	SPG21, maspardin	27965	0.127338175	0.319	0.223	0.022159626	1.50E-06
Caudoputamen	iTBS	Gpr158	G protein-coupled receptor 158	241263	0.127686522	0.579	0.452	0.009110795	6.17E-07
Caudoputamen	iTBS	Nop10	NOP10 ribonucleoprotein	66181	0.127694849	0.779	0.664	0.040442414	2.74E-06
Caudoputamen	iTBS	Krt10	keratin 10	16661	0.128129748	0.445	0.335	0.019156045	1.30E-06
Caudoputamen	iTBS	Hagh	hydroxyacyl glutathione hydrolase	14651	0.129555228	0.518	0.395	0.005670745	3.84E-07
Caudoputamen	iTBS	Rpl29	ribosomal protein L29	19944	0.129649113	0.986	0.983	0.000316408	2.14E-08
Caudoputamen	iTBS	Trappc4	trafficking protein particle complex 4	60409	0.130703692	0.41	0.289	0.001130745	7.65E-08
Caudoputamen	iTBS	Krt12	keratin 12	268482	0.131013165	0.181	0.087	9.65E-06	6.53E-10
Caudoputamen	iTBS	Stub1	STIP1 homology and U-Box containing protein 1	56424	0.131197451	0.625	0.479	0.02576908	1.74E-06
Caudoputamen	iTBS	Tpm2	tropomyosin 2, beta	22004	0.131261686	0.221	0.122	4.89E-05	3.31E-09
Caudoputamen	iTBS	Gpx4	glutathione peroxidase 4	625249	0.132844921	0.993	0.996	0.000439738	2.98E-08
Caudoputamen	iTBS	Rps20	ribosomal protein S20	67427	0.133178848	0.998	0.997	2.33E-06	1.58E-10
Caudoputamen	iTBS	Mrps16	mitochondrial ribosomal protein S16	66242	0.133403333	0.438	0.301	7.80E-05	5.28E-09
Caudoputamen	iTBS	Cd4	CD4 antigen	12504	0.134130177	0.383	0.281	0.008603104	5.82E-07
Caudoputamen	iTBS	Gnb2	guanine nucleotide binding protein (G protein), beta 2	14693	0.134546603	0.972	0.959	0.001638347	1.11E-07
Caudoputamen	iTBS	Rps2	ribosomal protein S2	16898	0.134972777	0.993	0.997	0.00016313	1.10E-08
Caudoputamen	iTBS	Calm2	calmodulin 2	12314	0.13525223	1	1	4.34E-11	2.94E-15
Caudoputamen	iTBS	Uqcrb	ubiquinol-cytochrome c reductase binding protein	67530	0.135557184	0.963	0.95	0.000420668	2.85E-08
Caudoputamen	iTBS	Ndfip2	Nedd4 family interacting protein 2	76273	0.135707297	0.51	0.376	0.000989939	6.70E-08
Caudoputamen	iTBS	Elmod1	ELMO/CED-12 domain containing 1	270162	0.13598525	0.923	0.845	0.009992017	6.76E-07
Caudoputamen	iTBS	Guk1	guanylate kinase 1	14923	0.136233495	0.662	0.517	0.006312181	4.27E-07
Caudoputamen	iTBS	Tmbim4	transmembrane BAX inhibitor motif containing 4	68212	0.136245958	0.337	0.211	2.78E-05	1.88E-09
Caudoputamen	iTBS	Erbin	Erbb2 interacting protein	59079	0.136341747	0.254	0.152	0.000148633	1.01E-08
Caudoputamen	iTBS	Dtnbp1	dystrobrevin binding protein 1	94245	0.136515096	0.477	0.35	0.004468356	3.02E-07
Caudoputamen	iTBS	2410006H16Rik	RIKEN cDNA 2410006H16 gene	69221	0.137231477	0.55	0.407	0.000457138	3.09E-08
Caudoputamen	iTBS	Rasl11b	RAS-like, family 11, member B	68939	0.137578343	0.211	0.11	1.13E-05	7.67E-10
Caudoputamen	iTBS	Rpl11	ribosomal protein L11	67025	0.137663562	0.999	0.999	1.63E-05	1.10E-09
Caudoputamen	iTBS	Hist1h1c	NA	NA	0.137854384	0.349	0.244	0.004865876	3.29E-07
Caudoputamen	iTBS	Sdhaf1	succinate dehydrogenase complex assembly factor 1	68332	0.137997131	0.295	0.184	0.000146673	9.93E-09
Caudoputamen	iTBS	H2afz	NA	NA	0.138024265	0.965	0.944	0.002928391	1.98E-07
Caudoputamen	iTBS	H2afj	NA	NA	0.13831767	0.681	0.532	0.002303265	1.56E-07
Caudoputamen	iTBS	Ndufa7	NADH:ubiquinone oxidoreductase subunit A7	66416	0.139650783	0.906	0.86	0.012651277	8.56E-07
Caudoputamen	iTBS	Mrpl57	mitochondrial ribosomal protein L57	67840	0.140309686	0.441	0.323	0.006038727	4.09E-07
Caudoputamen	iTBS	Polr2k	polymerase (RNA) II (DNA directed) polypeptide K	17749	0.140890946	0.593	0.453	0.002529818	1.71E-07
Caudoputamen	iTBS	Rplp2	ribosomal protein, large P2	67186	0.14114778	0.99	0.984	0.000881642	5.97E-08

Caudoputamen	iTBS	Neto2	neuropilin (NRP) and tolloid (TLL)-like 2	74513	0.142864537	0.456	0.333	0.000752087	5.09E-08
Caudoputamen	iTBS	Rps29	ribosomal protein S29	20090	0.142919956	0.999	0.998	0.034581606	2.34E-06
Caudoputamen	iTBS	mt-Cytb	NA	NA	0.143204314	1	1	4.58E-17	3.10E-21
Caudoputamen	iTBS	Dpm3	dolichyl-phosphate mannosyltransferase polypeptide 3	68563	0.145135466	0.456	0.316	4.00E-05	2.70E-09
Caudoputamen	iTBS	Tmsb4x	thymosin, beta 4, X chromosome	19241	0.14532626	1	1	2.36E-11	1.60E-15
Caudoputamen	iTBS	Cacna2d3	calcium channel, voltage-dependent, alpha2/delta subunit 3	12294	0.145513371	0.596	0.457	0.000446389	3.02E-08
Caudoputamen	iTBS	Mrpl17	mitochondrial ribosomal protein L17	27397	0.14685864	0.377	0.251	4.00E-05	2.71E-09
Caudoputamen	iTBS	2310009A05Rik	NA	NA	0.147912125	0.353	0.232	5.48E-05	3.71E-09
Caudoputamen	iTBS	Sbsn	suprabasin	282619	0.148124618	0.323	0.209	7.72E-05	5.23E-09
Caudoputamen	iTBS	Cystm1	cysteine-rich transmembrane module containing 1	66060	0.148875259	0.733	0.612	0.012622757	8.54E-07
Caudoputamen	iTBS	Cox8a	cytochrome c oxidase subunit 8A	12868	0.148932369	1	1	7.90E-10	5.35E-14
Caudoputamen	iTBS	Pcp4	Purkinje cell protein 4	18546	0.149493529	1	1	2.94E-06	1.99E-10
Caudoputamen	iTBS	Fau	Finkel-Biskis-Reilly murine sarcoma virus (FBR-MuSV) ubiquitously expres	14109	0.1508702	1	1	3.77E-08	2.55E-12
Caudoputamen	iTBS	Sem1	SEM1, 26S proteasome complex subunit	20422	0.151008339	0.798	0.667	0.001803604	1.22E-07
Caudoputamen	iTBS	Alkbh7	alkB homolog 7	66400	0.151155099	0.341	0.21	2.47E-06	1.67E-10
Caudoputamen	iTBS	Rpl38	ribosomal protein L38	67671	0.151503819	1	1	2.62E-09	1.77E-13
Caudoputamen	iTBS	Tesc	tescalcin	57816	0.15184919	0.921	0.847	0.000147514	9.98E-09
Caudoputamen	iTBS	1500011B03Rik	RIKEN cDNA 1500011B03 gene	66236	0.152142612	0.693	0.543	0.000838281	5.67E-08
Caudoputamen	iTBS	Rpl23	ribosomal protein L23	65019	0.153481126	0.997	0.996	2.91E-07	1.97E-11
Caudoputamen	iTBS	Selenom	selenoprotein M	114679	0.153689128	0.877	0.822	0.001342538	9.08E-08
Caudoputamen	iTBS	Ubb	ubiquitin B	22187	0.154058705	1	1	5.82E-05	3.94E-09
Caudoputamen	iTBS	Ppp1r1a	protein phosphatase 1, regulatory inhibitor subunit 1A	58200	0.155182596	0.901	0.797	0.004002154	2.71E-07
Caudoputamen	iTBS	Bloc1s1	biogenesis of lysosomal organelles complex-1, subunit 1	14533	0.155236462	0.358	0.227	5.45E-06	3.69E-10
Caudoputamen	iTBS	Spock3	sparc/osteonectin, cwcv and kazal-like domains proteoglycan 3	72902	0.155236462	0.909	0.812	0.00029273	1.98E-08
Caudoputamen	iTBS	Mrpl53	mitochondrial ribosomal protein L53	68499	0.156315883	0.596	0.44	2.24E-05	1.52E-09
Caudoputamen	iTBS	Uqcr11	ubiquinol-cytochrome c reductase, complex III subunit XI	66594	0.157387129	1	1	9.18E-08	6.21E-12
Caudoputamen	iTBS	Fth1	ferritin heavy polypeptide 1	14319	0.157583588	1	1	1.42E-05	9.62E-10
Caudoputamen	iTBS	Them6	thioesterase superfamily member 6	223626	0.157955336	0.49	0.357	0.000171956	1.16E-08
Caudoputamen	iTBS	Rps7	ribosomal protein S7	20115	0.160084489	0.998	0.996	1.55E-08	1.05E-12
Caudoputamen	iTBS	Sep-05	NA	NA	0.160434841	0.232	0.118	2.23E-07	1.51E-11
Caudoputamen	iTBS	Rps12	ribosomal protein S12	20042	0.160542342	0.993	0.985	7.09E-05	4.80E-09
Caudoputamen	iTBS	Ndufb8	NADH:ubiquinone oxidoreductase subunit B8	67264	0.160616367	0.965	0.948	1.30E-05	8.82E-10
Caudoputamen	iTBS	Ppp2cb	protein phosphatase 2 (formerly 2A), catalytic subunit, beta isoform	19053	0.161641854	0.181	0.06	1.01E-12	6.80E-17
Caudoputamen	iTBS	Rpl19	ribosomal protein L19	19921	0.163171573	0.997	0.995	1.69E-06	1.14E-10
Caudoputamen	iTBS	Gpr155	G protein-coupled receptor 155	68526	0.165938997	0.467	0.362	0.006532214	4.42E-07
Caudoputamen	iTBS	Rpl36a	ribosomal protein L36A	19982	0.169440959	0.938	0.906	3.50E-05	2.37E-09
Caudoputamen	iTBS	Drd1	dopamine receptor D1	13488	0.17050542	0.71	0.562	1.48E-05	9.99E-10
Caudoputamen	iTBS	Rps10	ribosomal protein S10	67097	0.171482183	0.99	0.99	6.54E-07	4.43E-11
Caudoputamen	iTBS	Ano3	anoctamin 3	228432	0.174217815	0.656	0.54	0.002853381	1.93E-07
Caudoputamen	iTBS	Tmsb10	thymosin, beta 10	19240	0.175295875	1	0.999	1.66E-07	1.12E-11
Caudoputamen	iTBS	Scn4b	sodium channel, type IV, beta	399548	0.175434314	0.856	0.762	0.007682914	5.20E-07
Caudoputamen	iTBS	Rps27a	ribosomal protein S27A	78294	0.175999941	1	1	6.87E-14	4.65E-18
Caudoputamen	iTBS	Rps16	ribosomal protein S16	20055	0.176155078	0.99	0.983	2.03E-07	1.38E-11
Caudoputamen	iTBS	Cox6c	cytochrome c oxidase subunit 6C	12864	0.176316061	1	1	8.10E-21	5.48E-25
Caudoputamen	iTBS	Rasgef1b	RasGEF domain family, member 1B	320292	0.176782876	0.439	0.31	1.36E-05	9.20E-10
Caudoputamen	iTBS	St8sia3	ST8 alpha-N-acetyl-neuraminide alpha-2,8-sialyltransferase 3	20451	0.177081673	0.863	0.765	0.000957541	6.48E-08
Caudoputamen	iTBS	Rpl34	ribosomal protein L34	68436	0.18012321	1	0.994	9.18E-08	6.21E-12
Caudoputamen	iTBS	S100a10	S100 calcium binding protein A10 (calpactin)	20194	0.180961075	0.291	0.163	2.01E-07	1.36E-11
Caudoputamen	iTBS	Rpl13	ribosomal protein L13	270106	0.189188214	1	1	1.08E-15	7.32E-20
Caudoputamen	iTBS	Rpl37	ribosomal protein L37	67281	0.190933163	1	1	5.07E-16	3.43E-20
Caudoputamen	iTBS	Sap18	Sin3-associated polypeptide 18	20220	0.190968995	0.449	0.275	1.54E-10	1.04E-14
Caudoputamen	iTBS	Sf3b5	splicing factor 3b, subunit 5	66125	0.191377442	0.597	0.431	4.58E-06	3.10E-10
Caudoputamen	iTBS	mt-Nd5	NA	NA	0.194157718	0.994	0.996	7.97E-08	5.39E-12
Caudoputamen	iTBS	Ndufa1	NADH:ubiquinone oxidoreductase subunit A1	54405	0.199500467	0.918	0.843	1.11E-07	7.54E-12
Caudoputamen	iTBS	mt-Atp6	NA	NA	0.19985248	1	1	1.55E-24	1.05E-28
Caudoputamen	iTBS	Malat1	metastasis associated lung adenocarcinoma transcript 1 (non-coding RNA	72289	0.202736245	0.773	0.645	0.000890041	6.02E-08
Caudoputamen	iTBS	Rps24	ribosomal protein S24	20088	0.210576663	1	1	1.02E-18	6.92E-23
Caudoputamen	iTBS	Erh	ERH mRNA splicing and mitosis factor	13877	0.215196624	0.452	0.278	8.69E-11	5.88E-15
Caudoputamen	iTBS	Rpl30	ribosomal protein L30	19946	0.216625612	1	0.999	6.69E-15	4.53E-19
Caudoputamen	iTBS	Gpr88	G-protein coupled receptor 88	64378	0.219196673	0.992	0.981	6.65E-14	4.50E-18
Caudoputamen	iTBS	Rpl9	ribosomal protein L9	20005	0.225953243	0.999	0.998	8.70E-17	5.89E-21
Caudoputamen	iTBS	Cox7c	cytochrome c oxidase subunit 7C	12867	0.228564363	1	1	1.24E-23	8.42E-28
Caudoputamen	iTBS	mt-Nd4	NA	NA	0.228672926	1	1	9.59E-16	6.49E-20
Caudoputamen	iTBS	Rps8	ribosomal protein S8	20116	0.230594434	0.996	1	1.52E-19	1.03E-23
Caudoputamen	iTBS	Ppp3ca	protein phosphatase 3, catalytic subunit, alpha isoform	19055	0.233753474	1	0.999	1.39E-16	9.44E-21
Caudoputamen	iTBS	mt-Nd1	NA	NA	0.234517462	1	1	8.02E-19	5.43E-23
Caudoputamen	iTBS	Rps26	ribosomal protein S26	27370	0.235225817	0.992	0.978	3.77E-14	2.55E-18
Caudoputamen	iTBS	Rps28	ribosomal protein S28	54127	0.2382301	0.95	0.935	2.04E-08	1.38E-12
Caudoputamen	iTBS	Hmgn2	high mobility group nucleosomal binding domain 2	15331	0.24812134	0.624	0.443	4.79E-11	3.24E-15
Caudoputamen	iTBS	Rpl35a	ribosomal protein L35A	57808	0.252392802	1	0.996	6.67E-24	4.51E-28
Caudoputamen	iTBS	mt-Co3	NA	NA	0.260275142	1	1	9.34E-48	6.32E-52
Caudoputamen	iTBS	Ppp1r1b	protein phosphatase 1, regulatory inhibitor subunit 1B	19049	0.261919919	0.997	0.991	2.19E-21	1.48E-25
Caudoputamen	iTBS	Rps21	ribosomal protein S21	66481	0.283112319	1	1	4.45E-41	3.01E-45
Caudoputamen	iTBS	mt-Co1	NA	NA	0.289256147	1	1	8.45E-52	5.72E-56
Caudoputamen	iTBS	Cnr1	cannabinoid receptor 1 (brain)	12801	0.292881538	0.681	0.532	3.39E-09	2.30E-13
Caudoputamen	iTBS	Adora2a	adenosine A2a receptor	11540	0.300944336	0.78	0.614	1.49E-13	1.01E-17
Caudoputamen	iTBS	Rpl35	ribosomal protein L35	66489	0.36964948	0.985	0.965	1.36E-31	9.19E-36
Caudoputamen	iTBS	mt-Co2	NA	NA	0.389695174	1	1	9.42E-38	6.37E-42
Caudoputamen	iTBS	Ttr	transthyretin	22139	0.615804318	0.258	0.141	4.31E-10	2.92E-14
Caudoputamen	iTBS	Gstp1	glutathione S-transferase, pi 1	14870	0.632915936	0.612	0.27	5.38E-56	3.64E-60
Caudoputamen	iTBS	Uba52	ubiquitin A-52 residue ribosomal protein fusion product 1	22186	0.904690012	0.883	0.78	4.63E-81	3.13E-85
Caudoputamen	iTBS	Gm10076	ribosomal protein L41 pseudogene	100126819	1.504442045	0.811	0.463	8.87E-131	6.00E-135
Lateral Septal Complex	iTBS	Ttr	transthyretin	22139	-1.617776097	0.174	0.321	0.023932756	1.62E-06
Lateral Septal Complex	iTBS	Prkca	protein kinase C, alpha	18750	-0.338440643	0.264	0.426	0.017054536	1.15E-06
Lateral Septal Complex	iTBS	Phyhip	phytanoyl-CoA hydroxylase interacting protein	105653	-0.317131003	0.289	0.483	0.000450812	3.05E-08
Lateral Septal Complex	iTBS	Cdk5r2	cyclin-dependent kinase 5, regulatory subunit 2 (p39)	12570	-0.29895976	0.232	0.441	5.13E-05	3.47E-09
Lateral Septal Complex	iTBS	Luzp2	leucine zipper protein 2	233271	-0.287605523	0.402	0.561	0.016368979	1.11E-06
Lateral Septal Complex	iTBS	Kcnq2	potassium voltage-gated channel, subfamily Q, member 2	16536	-0.276817724	0.46	0.621	0.044984929	3.04E-06
Lateral Septal Complex	iTBS	Sgk1	serum/glucocorticoid regulated kinase 1	20393	-0.276265073	0.113	0.287	0.000143616	9.72E-09
Lateral Septal Complex	iTBS	Parp6	poly (ADP-ribose) polymerase family, member 6	67287	-0.256255699	0.232	0.41	0.005951657	4.03E-07
Lateral Septal Complex	iTBS	Hectd4	HECT domain E3 ubiquitin protein ligase 4	269700	-0.253133208	0.334	0.514	0.019764442	1.34E-06
Lateral Septal Complex	iTBS	Kalrn	kalirin, RhoGEF kinase	545156	-0.252111211	0.27	0.449	0.016802031	1.14E-06
Lateral Septal Complex	iTBS	Ppp1cb	protein phosphatase 1 catalytic subunit beta	19046	-0.241326741	0.482	0.658	0.033368969	2.26E-06
Lateral Septal Complex	iTBS	Tuba1a	tubulin, alpha 1A	22142	-0.22386303	0.984	0.987	0.006064108	4.10E-07
Lateral Septal Complex	iTBS	Cpe	carboxypeptidase E	12876	-0.20052739	1	1	0.000114487	7.75E-09
Lateral Septal Complex	iTBS	Kcnip3	Kv channel interacting protein 3, calsenilin	56461	-0.191869732	0.132	0.29	0.011281535	7.63E-07
Lateral Septal Complex	iTBS	Fau	Finkel-Biskis-Reilly murine sarcoma virus (FBR-MuSV) ubiquitously expres	14109	0.159567276	1	1	0.008123359	5.50E-07
Lateral Septal Complex	iTBS	Rpl9	ribosomal protein L9	20005	0.170399839	1	1	0.02034592	1.38E-06
Lateral Septal Complex	iTBS	Atp5md	ATP synthase membrane subunit DAPIT	66477	0.176142097	1	1	0.043148473	2.92E-06
Lateral Septal Complex	iTBS	Rps20	ribosomal protein S20	67427	0.17890355	1	0.997	0.003517822	2.38E-07
Lateral Septal Complex	iTBS	mt-Nd1	NA	NA	0.202311763	1	1	0.002862704	1.94E-07
Lateral Septal Complex	iTBS	Rps21	ribosomal protein S21	66481	0.216266446	1	1	1.80E-07	1.22E-11
Lateral Septal Complex	iTBS	Rpl38	ribosomal protein L38	67671	0.228828493	1	1	1.06E-05	7.14E-10
Lateral Septal Complex	iTBS	Rps29	ribosomal protein S29	20090	0.310903719	1	1	0.00011808	7.99E-09
Lateral Septal Complex	iTBS	Rps28	ribosomal protein S28	54127	0.333394575	0.977	0.958	5.20E-05	3.52E-09
Lateral Septal Complex	iTBS	Rpl35	ribosomal protein L35	66489	0.444890654	0.997	0.948	3.81E-13	2.58E-17
Lateral Septal Complex	iTBS	Uba52	ubiquitin A-52 residue ribosomal protein fusion product 1	22186	0.752594811	0.833	0.705	7.13E-16	4.82E-20
Lateral Septal Complex	iTBS	Gm10076	ribosomal protein L41 pseudogene	100126819	1.23638845	0.646	0.496	2.39E-15	1.62E-19
Striatum Ventral Region	iTBS	Bc1	brain cytoplasmic RNA 1	100568459	-0.384592556	0.676	0.83	6.83E-07	4.62E-11
Striatum Ventral Region	iTBS	Atp1a3	ATPase, Na+/K+ transporting, alpha 3 polypeptide	232975	-0.266323597	0.735	0.818	6.50E-06	4.40E-10
Striatum Ventral Region	iTBS	Actb	actin, beta	11461	-0.256477526	0.999	0.998	1.64E-07	1.11E-11
Striatum Ventral Region	iTBS	Syn1	synapsin I	20964	-0.239973357	0.615	0.712	0.022842128	1.55E-06
Striatum Ventral Region	iTBS	Meg3	maternally expressed 3	17263	-0.229628851	0.986	0.992	0.002868654	1.94E-07
Striatum Ventral Region	iTBS	Dnm1	dynamin 1	13429	-0.22820985	0.878	0.931	0.013451967	9.10E-07
Striatum Ventral Region	iTBS	Glul	glutamate-ammonia ligase (glutamine synthetase)	14645	-0.220806292	0.745	0.819	0.008322698	5.63E-07
Striatum Ventral Region	iTBS	Dynll2	dynein light chain LC8-type 2	68097	-0.216258769	0.915	0.949	0.006182429	4.18E-07
Striatum Ventral Region	iTBS	Gnao1	guanine nucleotide binding protein, alpha O	14681	-0.197577158	0.752	0.801	0.028294206	1.91E-06
Striatum Ventral Region	iTBS	Eef2	eukaryotic translation elongation factor 2	13629	-0.195034193	0.953	0.97	0.033525655	2.27E-06
Striatum Ventral Region	iTBS	Hspa8	heat shock protein 8	15481	-0.132345271	1	1	0.007522291	5.09E-07
Striatum Ventral Region	iTBS	Eef1a1	eukaryotic translation elongation factor 1 alpha 1	13627	0.095476485	1	1	0.014086426	9.53E-07
Striatum Ventral Region	iTBS	Coro1b	coronin, actin binding protein 1B	23789	0.102270482	0.121	0.044	0.005963946	4.04E-07
Striatum Ventral Region	iTBS	Amd1	S-adenosylmethionine decarboxylase 1	11702	0.113504408	0.168	0.075	0.005113521	3.46E-07
Striatum Ventral Region	iTBS	Commd10	COMM domain containing 10	69456	0.116287955	0.187	0.095	0.029554245	2.00E-06
Striatum Ventral Region	iTBS	Rpl24	ribosomal protein L24	68193	0.123032889	0.997	0.994	0.02754093	1.86E-06
Striatum Ventral Region	iTBS	Rplp1	ribosomal protein, large, P1	56040	0.124914551	1	1	0.003539279	2.39E-07
Striatum Ventral Region	iTBS	mt-Cytb	NA	NA	0.127201826	1	1	6.00E-05	4.06E-09
Striatum Ventral Region	iTBS	Atp5md	ATP synthase membrane subunit DAPIT	66477	0.135900316	1	1	0.047140952	3.19E-06
Striatum Ventral Region	iTBS	2310009A05Rik	NA	NA	0.136915374	0.32	0.201	0.026764124	1.81E-06
Striatum Ventral Region	iTBS	Rpl35a	ribosomal protein L35A	57808	0.140828462	1	1	0.001125769	7.62E-08
Striatum Ventral Region	iTBS	mt-Nd1	NA	NA	0.140855782	1	1	0.006589403	4.46E-07
Striatum Ventral Region	iTBS	Rps19bp1	ribosomal protein S19 binding protein 1	66538	0.143582824	0.363	0.238	0.041556109	2.81E-06
Striatum Ventral Region	iTBS	Rps20	ribosomal protein S20	67427	0.144449047	1	0.998	0.000390694	2.64E-08
Striatum Ventral Region	iTBS	Rpl9	ribosomal protein L9	20005	0.145143374	1	0.998	0.00075809	5.13E-08
Striatum Ventral Region	iTBS	Bloc1s1	biogenesis of lysosomal organelles complex-1, subunit 1	14533	0.145599063	0.369	0.237	0.007842228	5.31E-07
Striatum Ventral Region	iTBS	Pdap1	PDGFA associated protein 1	231887	0.146322156	0.439	0.31	0.039830082	2.70E-06
Striatum Ventral Region	iTBS	Cox6c	cytochrome c oxidase subunit 6C	12864	0.150062654	1	1	1.79E-05	1.21E-09
Striatum Ventral Region	iTBS	mt-Nd4	NA	NA	0.150259595	1	1	0.003413319	2.31E-07
Striatum Ventral Region	iTBS	Grcc10	gene rich cluster, C10 gene	14790	0.15541791	0.951	0.914	0.048171215	3.26E-06
Striatum Ventral Region	iTBS	Tmsb4x	thymosin, beta 4, X chromosome	19241	0.159733412	1	1	4.10E-06	2.77E-10
Striatum Ventral Region	iTBS	Rps23	ribosomal protein S23	66475	0.160366144	0.994	0.986	0.009850205	6.67E-07
Striatum Ventral Region	iTBS	Cebpzos	CCAAT/enhancer binding protein (C/EBP), zeta, opposite strand	68554	0.16133521	0.462	0.329	0.013544473	9.17E-07
Striatum Ventral Region	iTBS	Chrac1	chromatin accessibility complex 1	93696	0.161847675	0.326	0.192	0.000635848	4.30E-08
Striatum Ventral Region	iTBS	Rpl34	ribosomal protein L34	68436	0.163652	0.996	0.998	0.000220641	1.49E-08
Striatum Ventral Region	iTBS	Sec61b	Sec61 beta subunit	66212	0.166965415	0.532	0.416	0.048172761	3.26E-06
Striatum Ventral Region	iTBS	Snhg8	small nucleolar RNA host gene 8	69895	0.180106823	0.571	0.431	0.005217216	3.53E-07
Striatum Ventral Region	iTBS	Ppp2cb	protein phosphatase 2 (formerly 2A), catalytic subunit, beta isoform	19053	0.183863728	0.197	0.064	1.36E-08	9.20E-13
Striatum Ventral Region	iTBS	Lrpap1	low density lipoprotein receptor-related protein associated protein 1	16976	0.185620024	0.749	0.615	0.022212214	1.50E-06
Striatum Ventral Region	iTBS	Rpl38	ribosomal protein L38	67671	0.189170933	1	1	5.27E-09	3.56E-13
Striatum Ventral Region	iTBS	mt-Atp6	NA	NA	0.194787098	1	1	5.38E-12	3.64E-16
Striatum Ventral Region	iTBS	Ndufb4	NADH:ubiquinone oxidoreductase subunit B4	68194	0.195124326	0.954	0.914	0.002290551	1.55E-07
Striatum Ventral Region	iTBS	Rps27	ribosomal protein S27	57294	0.198069547	0.991	0.991	0.016344221	1.11E-06
Striatum Ventral Region	iTBS	Cox7c	cytochrome c oxidase subunit 7C	12867	0.200940927	1	0.998	5.13E-09	3.47E-13
Striatum Ventral Region	iTBS	Ubb	ubiquitin B	22187	0.201471016	1	1	3.82E-10	2.58E-14
Striatum Ventral Region	iTBS	Rps21	ribosomal protein S21	66481	0.2017355	1	1	2.38E-10	1.61E-14
Striatum Ventral Region	iTBS	Ppia	peptidylprolyl isomerase A	268373	0.203162049	1	1	1.86E-12	1.26E-16
Striatum Ventral Region	iTBS	Tmsb10	thymosin, beta 10	19240	0.206648963	1	1	1.02E-05	6.89E-10
Striatum Ventral Region	iTBS	Eno1	enolase 1, alpha non-neuron	13806	0.214430604	0.892	0.85	0.043445689	2.94E-06
Striatum Ventral Region	iTBS	Setp5	NA	NA	0.214773317	0.23	0.087	1.86E-08	1.26E-12
Striatum Ventral Region	iTBS	Rps29	ribosomal protein S29	20090	0.217502852	0.999	0.998	0.011498336	7.78E-07
Striatum Ventral Region	iTBS	mt-Co3	NA	NA	0.218541483	1	1	2.29E-16	1.55E-20
Striatum Ventral Region	iTBS	Ndufb1-ps	NA	NA	0.220992761	0.897	0.829	0.018149062	1.23E-06
Striatum Ventral Region	iTBS	mt-Co1	NA	NA	0.234626116	1	1	1.57E-16	1.07E-20
Striatum Ventral Region	iTBS	mt-Co2	NA	NA	0.254479414	1	1	9.80E-09	6.63E-13
Striatum Ventral Region	iTBS	Rps28	ribosomal protein S28	54127	0.270465407	0.96	0.964	0.00011676	7.90E-09
Striatum Ventral Region	iTBS	Rpl35	ribosomal protein L35	66489	0.305537969	0.984	0.972	2.08E-15	1.40E-19
Striatum Ventral Region	iTBS	Gstp1	glutathione S-transferase, pi 1	14870	0.478605517	0.584	0.368	7.52E-17	5.09E-21
Striatum Ventral Region	iTBS	Uba52	ubiquitin A-52 residue ribosomal protein fusion product 1	22186	0.801778525	0.879	0.832	9.45E-38	6.39E-42
Striatum Ventral Region	iTBS	Gm10076	ribosomal protein L41 pseudogene	100126819	1.338143498	0.733	0.59	7.19E-48	4.87E-52
White Matter Tracts	cTBS	Bc1	brain cytoplasmic RNA 1	100568459	-0.834899137	0.713	0.885	1.48E-22	9.99E-27
White Matter Tracts	cTBS	Nrsn2	neurensin 2	228777	-0.284072589	0.339	0.538	8.05E-08	5.45E-12
White Matter Tracts	cTBS	Tmem130	transmembrane protein 130	243339	-0.277167142	0.253	0.44	5.40E-07	3.66E-11
White Matter Tracts	cTBS	Resp18	regulated endocrine-specific protein 18	19711	-0.252238902	0.594	0.73	0.000117832	7.97E-09
White Matter Tracts	cTBS	Mgrn1	mahogunin, ring finger 1	17237	-0.241863763	0.355	0.518	0.002295737	1.55E-07
White Matter Tracts	cTBS	Ncald	neurocalcin delta	52589	-0.232309316	0.43	0.592	0.003656749	2.47E-07
White Matter Tracts	cTBS	mt-Nd2	NA	NA	-0.228247148	1	1	0.014044266	9.50E-07
White Matter Tracts	cTBS	Mapk8ip3	mitogen-activated protein kinase 8 interacting protein 3	30957	-0.223116101	0.48	0.625	0.035610238	2.41E-06
White Matter Tracts	cTBS	Fam234b	family with sequence similarity 234, member B	74525	-0.223088611	0.329	0.505	0.001068056	7.23E-08
White Matter Tracts	cTBS	Ahi1	Abelson helper integration site 1	52906	-0.22077216	0.165	0.298	0.006223014	4.21E-07
White Matter Tracts	cTBS	Atp6v1a	ATPase, H+ transporting, lysosomal V1 subunit A	11964	-0.217659317	0.289	0.453	0.000734314	4.97E-08
White Matter Tracts	cTBS	Gad2	glutamic acid decarboxylase 2	14417	-0.215179393	0.434	0.613	4.74E-05	3.21E-09
White Matter Tracts	cTBS	Rnf157	ring finger protein 157	217340	-0.212327836	0.271	0.417	0.013796978	9.34E-07
White Matter Tracts	cTBS	Nnat	neuronatin	18111	-0.209886442	0.462	0.625	0.035573989	2.41E-06
White Matter Tracts	cTBS	Ubqln2	ubiquilin 2	54609	-0.209403346	0.271	0.422	0.01200918	8.13E-07
White Matter Tracts	cTBS	Podxl2	podocalyxin-like 2	319655	-0.209344813	0.373	0.542	0.000173366	1.17E-08
White Matter Tracts	cTBS	Ank3	ankyrin 3, epithelial	11735	-0.206809269	0.359	0.523	0.009327571	6.31E-07
White Matter Tracts	cTBS	Sucla2	succinate-Coenzyme A ligase, ADP-forming, beta subunit	20916	-0.205068078	0.257	0.408	0.007131674	4.83E-07
White Matter Tracts	cTBS	Kcnq2	potassium voltage-gated channel, subfamily Q, member 2	16536	-0.200137314	0.474	0.615	0.033376997	2.26E-06
White Matter Tracts	cTBS	Spock2	sparc/osteonectin, cwcv and kazal-like domains proteoglycan 2	94214	-0.198843348	0.394	0.55	0.011626411	7.87E-07
White Matter Tracts	cTBS	Lingo1	leucine rich repeat and Ig domain containing 1	235402	-0.198233321	0.361	0.518	0.002792435	1.89E-07
White Matter Tracts	cTBS	Agpat3	1-acylglycerol-3-phosphate O-acyltransferase 3	28169	-0.197760431	0.303	0.463	0.025502357	1.73E-06
White Matter Tracts	cTBS	Laptm4a	lysosomal-associated protein transmembrane 4A	17775	-0.195376655	0.335	0.497	0.017378952	1.18E-06
White Matter Tracts	cTBS	Svop	SV2 related protein	68666	-0.193769291	0.203	0.362	0.000358251	2.42E-08
White Matter Tracts	cTBS	Nrxn3	neurexin III	18191	-0.193694903	0.303	0.453	0.007312266	4.95E-07
White Matter Tracts	cTBS	Nell2	NEL-like 2	54003	-0.193211199	0.171	0.298	0.01749695	1.18E-06
White Matter Tracts	cTBS	Ndn	necdin, MAGE family member	17984	-0.192004768	0.38	0.53	0.032937367	2.23E-06
White Matter Tracts	cTBS	Rnf208	ring finger protein 208	68846	-0.190986229	0.329	0.483	0.013495216	9.13E-07
White Matter Tracts	cTBS	Oaz2	ornithine decarboxylase antizyme 2	18247	-0.190739685	0.273	0.428	0.004861078	3.29E-07
White Matter Tracts	cTBS	Cbarp	calcium channel, voltage-dependent, beta subunit associated regulatory p	100503659	-0.189355487	0.51	0.662	0.018092201	1.22E-06
White Matter Tracts	cTBS	Setp3	NA	NA	-0.188668404	0.422	0.583	0.005538835	3.75E-07
White Matter Tracts	cTBS	Ap2a2	adaptor-related protein complex 2, alpha 2 subunit	11772	-0.187327725	0.444	0.602	0.00836411	5.66E-07
White Matter Tracts	cTBS	Lamp5	lysosomal-associated membrane protein family, member 5	76161	-0.186393368	0.339	0.495	0.00458906	3.11E-07
White Matter Tracts	cTBS	Impact	impact, RWD domain protein	16210	-0.185629156	0.546	0.698	0.011978469	8.11E-07
White Matter Tracts	cTBS	Pfdn1	prefoldin 1	67199	-0.185465292	0.315	0.477	0.00938698	6.35E-07
White Matter Tracts	cTBS	Abr	active BCR-related gene	109934	-0.184752	0.637	0.757	0.040492761	2.74E-06
White Matter Tracts	cTBS	Klc1	kinesin light chain 1	16593	-0.184045107	0.972	0.98	0.021866516	1.48E-06
White Matter Tracts	cTBS	Grik5	glutamate receptor, ionotropic, kainate 5 (gamma 2)	14809	-0.183476527	0.53	0.693	0.003171957	2.15E-07
White Matter Tracts	cTBS	Myl12b	myosin, light chain 12B, regulatory	67938	-0.180891292	0.952	0.968	0.013786298	9.33E-07
White Matter Tracts	cTBS	Tmx4	thioredoxin-related transmembrane protein 4	52837	-0.180164061	0.231	0.37	0.029434876	1.99E-06
White Matter Tracts	cTBS	Cadm3	cell adhesion molecule 3	94332	-0.179181346	0.321	0.475	0.006748344	4.57E-07
White Matter Tracts	cTBS	Arpc4	actin related protein 2/3 complex, subunit 4	68089	-0.17766192	0.261	0.417	0.005852999	3.96E-07
White Matter Tracts	cTBS	Usp14	ubiquitin specific peptidase 14	59025	-0.176679576	0.223	0.358	0.043315217	2.93E-06
White Matter Tracts	cTBS	Peg13	paternally expressed 13	353342	-0.176667368	0.5	0.657	0.009057787	6.13E-07
White Matter Tracts	cTBS	Ncs1	neuronal calcium sensor 1	14299	-0.174647799	0.345	0.507	0.002456908	1.66E-07
White Matter Tracts	cTBS	Ly6h	lymphocyte antigen 6 complex, locus H	23934	-0.174613564	0.92	0.947	0.009880292	6.69E-07
White Matter Tracts	cTBS	Gnal	guanine nucleotide binding protein, alpha stimulating, olfactory type	14680	-0.173359218	0.43	0.583	0.022761389	1.54E-06
White Matter Tracts	cTBS	Usp5	ubiquitin specific peptidase 5 (isopeptidase T)	22225	-0.172837683	0.271	0.427	0.011191193	7.57E-07
White Matter Tracts	cTBS	Scg2	secretogranin II	20254	-0.171455842	0.375	0.53	0.017674928	1.20E-06
White Matter Tracts	cTBS	Snrpn	small nuclear ribonucleoprotein N	20646	-0.169969268	0.972	0.995	0.020180695	1.37E-06
White Matter Tracts	cTBS	Atp1b1	ATPase, Na+/K+ transporting, beta 1 polypeptide	11931	-0.167378839	0.984	0.993	0.046752577	3.16E-06
White Matter Tracts	cTBS	Stxbp1	syntaxin binding protein 1	20910	-0.167100227	0.886	0.925	0.01432875	9.70E-07
White Matter Tracts	cTBS	Rasgrf1	RAS protein-specific guanine nucleotide-releasing factor 1	19417	-0.166499029	0.478	0.632	0.008057517	5.45E-07
White Matter Tracts	cTBS	Snap25	synaptosomal-associated protein 25	20614	-0.166119584	0.984	0.997	0.013510926	9.14E-07
White Matter Tracts	cTBS	Vsnl1	visinin-like 1	26950	-0.16403496	0.936	0.945	0.003732621	2.53E-07
White Matter Tracts	cTBS	Arhgef9	CDC42 guanine nucleotide exchange factor (GEF) 9	236915	-0.161932004	0.301	0.458	0.008566716	5.80E-07
White Matter Tracts	cTBS	Tceal6	transcription elongation factor A (SII)-like 6	66104	-0.161113572	0.229	0.373	0.013361967	9.04E-07
White Matter Tracts	cTBS	Pvalb	parvalbumin	19293	-0.160314451	0.319	0.468	0.017849253	1.21E-06
White Matter Tracts	cTBS	Zwint	ZW10 interactor	52696	-0.15720523	0.924	0.947	0.014738814	9.97E-07
White Matter Tracts	cTBS	Zfr	zinc finger RNA binding protein	22763	-0.155913938	0.392	0.55	0.035759334	2.42E-06
White Matter Tracts	cTBS	Chga	chromogranin A	12652	-0.155506389	0.203	0.337	0.025794313	1.75E-06
White Matter Tracts	cTBS	Ywhag	tyrosine 3-monooxygenase/tryptophan 5-monooxygenase activation pro	22628	-0.154151109	0.936	0.96	0.008799121	5.95E-07
White Matter Tracts	cTBS	Slc22a17	solute carrier family 22 (organic cation transporter), member 17	59049	-0.153594729	0.964	0.99	0.010319595	6.98E-07
White Matter Tracts	cTBS	Smim13	small integral membrane protein 13	108934	-0.153100985	0.418	0.582	0.018265882	1.24E-06
White Matter Tracts	cTBS	Tmem132a	transmembrane protein 132A	98170	-0.146683403	0.496	0.648	0.046609006	3.15E-06
White Matter Tracts	cTBS	Jph3	junctophilin 3	57340	-0.143461799	0.237	0.375	0.047528763	3.22E-06
White Matter Tracts	cTBS	Rtn1	reticulon 1	104001	-0.141832055	0.982	0.99	0.011747176	7.95E-07
White Matter Tracts	cTBS	Snhg11	small nucleolar RNA host gene 11	319317	-0.134986386	0.914	0.93	0.02253315	1.52E-06
White Matter Tracts	cTBS	Rpl21	ribosomal protein L21	19933	0.143483302	1	1	0.014644055	9.91E-07
White Matter Tracts	cTBS	Cox8a	cytochrome c oxidase subunit 8A	12868	0.152716569	1	1	0.026500607	1.79E-06
White Matter Tracts	cTBS	Rps21	ribosomal protein S21	66481	0.153368289	1	1	0.019612584	1.33E-06
White Matter Tracts	cTBS	Cox6a1	cytochrome c oxidase subunit 6A1	12861	0.189933891	1	1	0.004682218	3.17E-07
White Matter Tracts	cTBS	Rpl23	ribosomal protein L23	65019	0.200256715	1	1	0.000407168	2.76E-08
White Matter Tracts	cTBS	Rpl35	ribosomal protein L35	66489	0.224337442	0.998	1	0.010046001	6.80E-07
White Matter Tracts	cTBS	Cdc37l1	cell division cycle 37-like 1	67072	0.251715611	0.245	0.125	0.008505936	5.76E-07
White Matter Tracts	cTBS	Plekhb1	pleckstrin homology domain containing, family B (evectins) member 1	27276	0.261595664	0.982	0.978	0.019060061	1.29E-06
White Matter Tracts	cTBS	Lars2	leucyl-tRNA synthetase, mitochondrial	102436	0.264064302	0.285	0.167	0.029131291	1.97E-06

White Matter Tracts	cTBS	Ftl1	ferritin light polypeptide 1	14325	0.305588024	1	1	3.24E-07	2.19E-11
White Matter Tracts	cTBS	Kif5a	kinesin family member 5A	16572	0.31720512	0.992	0.99	1.21E-05	8.22E-10
White Matter Tracts	cTBS	Mobp	myelin-associated oligodendrocytic basic protein	17433	0.317797139	0.998	1	7.71E-06	5.22E-10
White Matter Tracts	cTBS	Mbp	myelin basic protein	17196	0.338416104	1	1	3.39E-09	2.29E-13
White Matter Tracts	cTBS	Bcas1	brain enriched myelin associated protein 1	76960	0.348352484	0.695	0.545	0.000721123	4.88E-08
White Matter Tracts	cTBS	Rapgef4	Rap guanine nucleotide exchange factor (GEF) 4	56508	0.367187139	0.843	0.795	1.53E-05	1.03E-09
White Matter Tracts	cTBS	Uba52	ubiquitin A-52 residue ribosomal protein fusion product 1	22186	0.485056077	0.968	0.958	1.50E-12	1.02E-16
White Matter Tracts	cTBS	Fth1	ferritin heavy polypeptide 1	14319	0.517891522	1	1	5.79E-25	3.92E-29
White Matter Tracts	cTBS	Gm10076	ribosomal protein L41 pseudogene	100126819	0.565014967	0.938	0.887	5.47E-20	3.70E-24
White Matter Tracts	cTBS	Gstp1	glutathione S-transferase, pi 1	14870	0.685173258	0.707	0.597	3.50E-11	2.37E-15
Caudoputamen	cTBS	Bc1	brain cytoplasmic RNA 1	100568459	-0.704317676	0.342	0.693	2.11E-61	1.43E-65
Caudoputamen	cTBS	Ttr	transthyretin	22139	-0.326153983	0.002	0.141	4.66E-33	3.15E-37
Caudoputamen	cTBS	Vsnl1	visinin-like 1	26950	-0.309329542	0.314	0.544	1.38E-22	9.34E-27
Caudoputamen	cTBS	Slc17a7	solute carrier family 17 (sodium-dependent inorganic phosphate cotrans|	72961	-0.272338813	0.196	0.361	1.47E-14	9.94E-19
Caudoputamen	cTBS	Olfm1	olfactomedin 1	56177	-0.266129212	0.585	0.764	1.65E-13	1.12E-17
Caudoputamen	cTBS	Stmn1	stathmin 1	16765	-0.252160997	0.614	0.772	7.28E-09	4.93E-13
Caudoputamen	cTBS	Gng3	guanine nucleotide binding protein (G protein), gamma 3	14704	-0.250711456	0.733	0.857	1.40E-09	9.47E-14
Caudoputamen	cTBS	Ptgds	prostaglandin D2 synthase (brain)	19215	-0.241162297	0.671	0.813	4.81E-06	3.25E-10
Caudoputamen	cTBS	Tubb2a	tubulin, beta 2A class IIA	22151	-0.237262089	0.595	0.713	2.58E-07	1.74E-11
Caudoputamen	cTBS	Nap1l5	nucleosome assembly protein 1-like 5	58243	-0.220257779	0.513	0.643	1.11E-05	7.51E-10
Caudoputamen	cTBS	Tuba1b	tubulin, alpha 1B	22143	-0.212309698	0.72	0.833	2.01E-06	1.36E-10
Caudoputamen	cTBS	Atp1a3	ATPase, Na+/K+ transporting, alpha 3 polypeptide	232975	-0.20502498	0.653	0.733	1.92E-05	1.30E-09
Caudoputamen	cTBS	Ywhah	tyrosine 3-monooxygenase/tryptophan 5-monooxygenase activation pro	22629	-0.203692008	0.918	0.958	1.32E-07	8.95E-12
Caudoputamen	cTBS	Tuba1a	tubulin, alpha 1A	22142	-0.201513444	0.978	0.984	3.39E-08	2.29E-12
Caudoputamen	cTBS	Cdk5r2	cyclin-dependent kinase 5, regulatory subunit 2 (p39)	12570	-0.201139872	0.338	0.433	0.000573152	3.88E-08
Caudoputamen	cTBS	Ikbkb	inhibitor of kappaB kinase beta	16150	-0.199450592	0.214	0.32	2.22E-05	1.50E-09
Caudoputamen	cTBS	Ly6h	lymphocyte antigen 6 complex, locus H	23934	-0.198346836	0.692	0.779	8.21E-05	5.55E-09
Caudoputamen	cTBS	Sncb	synuclein, beta	104069	-0.197357629	0.462	0.544	0.007066572	4.78E-07
Caudoputamen	cTBS	Tubb4b	tubulin, beta 4B class IVB	227613	-0.19667221	0.545	0.625	0.000546345	3.70E-08
Caudoputamen	cTBS	Cck	cholecystokinin	12424	-0.193032292	0.271	0.416	6.68E-08	4.52E-12
Caudoputamen	cTBS	Tagln3	transgelin 3	56370	-0.188334531	0.648	0.725	0.001625043	1.10E-07
Caudoputamen	cTBS	Gnas	GNAS (guanine nucleotide binding protein, alpha stimulating) complex lo	14683	-0.177807492	0.84	0.9	0.000220315	1.49E-08
Caudoputamen	cTBS	Prkar1b	protein kinase, cAMP dependent regulatory, type I beta	19085	-0.174957273	0.609	0.728	0.000102757	6.95E-09
Caudoputamen	cTBS	Atp6v1c1	ATPase, H+ transporting, lysosomal V1 subunit C1	66335	-0.166724918	0.63	0.69	0.034745517	2.35E-06
Caudoputamen	cTBS	Atp6v1g2	ATPase, H+ transporting, lysosomal V1 subunit G2	66237	-0.159546273	0.779	0.848	0.008914432	6.03E-07
Caudoputamen	cTBS	Zwint	ZW10 interactor	52696	-0.158745677	0.819	0.889	0.027501402	1.86E-06
Caudoputamen	cTBS	Snhg9	small nucleolar RNA host gene 9	73474	-0.154812189	0.155	0.252	0.000160041	1.08E-08
Caudoputamen	cTBS	Calm1	calmodulin 1	12313	-0.115803336	1	1	2.81E-05	1.90E-09
Caudoputamen	cTBS	mt-Co3	NA	NA	0.073890073	1	1	0.046084267	3.12E-06
Caudoputamen	cTBS	Gpr139	G protein-coupled receptor 139	209776	0.078920768	0.112	0.052	0.00897228	6.07E-07
Caudoputamen	cTBS	mt-Atp6	NA	NA	0.082285255	1	1	0.000337469	2.28E-08
Caudoputamen	cTBS	Ngdn	neuroguidin, EIF4E binding protein	68966	0.085301829	0.154	0.086	0.023254536	1.57E-06
Caudoputamen	cTBS	Ppp2cb	protein phosphatase 2 (formerly 2A), catalytic subunit, beta isoform	19053	0.086728982	0.118	0.06	0.045863103	3.10E-06
Caudoputamen	cTBS	Slc7a10	solute carrier family 7 (cationic amino acid transporter, y+ system), mem	53896	0.087363309	0.174	0.1	0.019610079	1.33E-06
Caudoputamen	cTBS	AI854703	expressed sequence AI854703	243373	0.089868213	0.158	0.09	0.031563864	2.14E-06
Caudoputamen	cTBS	Bmp1	bone morphogenetic protein 1	12153	0.089911578	0.158	0.086	0.008170507	5.53E-07
Caudoputamen	cTBS	Rpl13	ribosomal protein L13	270106	0.090881783	1	1	0.017044571	1.15E-06
Caudoputamen	cTBS	Ckb	creatine kinase, brain	12709	0.091838068	1	1	3.93E-05	2.66E-09
Caudoputamen	cTBS	Tmem80	transmembrane protein 80	71448	0.092756731	0.146	0.08	0.027157097	1.84E-06
Caudoputamen	cTBS	Me2	malic enzyme 2, NAD(+)-dependent, mitochondrial	107029	0.095856804	0.161	0.092	0.030245957	2.05E-06
Caudoputamen	cTBS	Rplp1	ribosomal protein, large, P1	56040	0.096395004	1	1	0.003704521	2.51E-07
Caudoputamen	cTBS	Slc30a10	solute carrier family 30, member 10	226781	0.097045834	0.197	0.122	0.041418405	2.80E-06
Caudoputamen	cTBS	Rngtt	RNA guanylyltransferase and 5'-phosphatase	24018	0.099527177	0.179	0.102	0.006782545	4.59E-07
Caudoputamen	cTBS	Leprot	leptin receptor overlapping transcript	230514	0.101956999	0.196	0.115	0.007072742	4.79E-07
Caudoputamen	cTBS	Ubb	ubiquitin B	22187	0.10383519	1	1	0.001155313	7.82E-08
Caudoputamen	cTBS	Mapk6	mitogen-activated protein kinase 6	50772	0.104936171	0.215	0.137	0.038981868	2.64E-06
Caudoputamen	cTBS	Mgat5b	mannoside acetylglucosaminyltransferase 5, isoenzyme B	268510	0.105717146	0.422	0.31	0.028703164	1.94E-06
Caudoputamen	cTBS	Esam	endothelial cell-specific adhesion molecule	69524	0.106097339	0.159	0.085	0.002548146	1.72E-07
Caudoputamen	cTBS	Eml5	echinoderm microtubule associated protein like 5	319670	0.107269628	0.301	0.205	0.017347444	1.17E-06
Caudoputamen	cTBS	Arf3	ADP-ribosylation factor 3	11842	0.107834086	0.999	0.994	0.027089307	1.83E-06
Caudoputamen	cTBS	Cox5b	cytochrome c oxidase subunit 5B	12859	0.110870975	1	0.999	0.001662978	1.13E-07
Caudoputamen	cTBS	Btrc	beta-transducin repeat containing protein	12234	0.110893671	0.266	0.174	0.009035169	6.11E-07
Caudoputamen	cTBS	Hnrnpm	heterogeneous nuclear ribonucleoprotein M	76936	0.111647034	0.512	0.379	0.013281261	8.99E-07
Caudoputamen	cTBS	Rpl37	ribosomal protein L37	67281	0.111932077	1	1	0.00036571	2.47E-08
Caudoputamen	cTBS	Letmd1	LETM1 domain containing 1	68614	0.11323381	0.27	0.172	0.002533393	1.71E-07
Caudoputamen	cTBS	Slc22a23	solute carrier family 22, member 23	73102	0.113779678	0.246	0.154	0.002504694	1.69E-07
Caudoputamen	cTBS	Gm27032	predicted gene, 27032	118568482	0.113793461	0.217	0.13	0.002385642	1.61E-07
Caudoputamen	cTBS	Sema7a	sema domain, immunoglobulin domain (Ig), and GPI membrane anchor, (	20361	0.114142869	0.179	0.1	0.002879547	1.95E-07
Caudoputamen	cTBS	Atp5j2	ATP synthase, H+ transporting, mitochondrial F0 complex, subunit F2	57423	0.115780641	1	0.999	0.003896059	2.64E-07
Caudoputamen	cTBS	Slc6a6	solute carrier family 6 (neurotransmitter transporter, taurine), member 6	21366	0.116471156	0.35	0.242	0.005002295	3.38E-07
Caudoputamen	cTBS	B3gnt2	UDP-GlcNAc:betaGal beta-1,3-N-acetylglucosaminyltransferase 2	53625	0.117776775	0.463	0.335	0.002861953	1.94E-07
Caudoputamen	cTBS	Inpp5j	inositol polyphosphate 5-phosphatase J	170835	0.118319967	0.32	0.215	0.003198792	2.16E-07
Caudoputamen	cTBS	Cox4i1	cytochrome c oxidase subunit 4I1	12857	0.118694659	1	1	4.82E-06	3.26E-10
Caudoputamen	cTBS	Actg1	actin, gamma, cytoplasmic 1	11465	0.118837336	1	1	8.04E-06	5.44E-10
Caudoputamen	cTBS	Actn1	actinin, alpha 1	109711	0.119228558	0.489	0.378	0.014583345	9.87E-07
Caudoputamen	cTBS	Rbfox1	RNA binding protein, fox-1 homolog (C. elegans) 1	268859	0.119350096	0.229	0.135	0.000393743	2.66E-08
Caudoputamen	cTBS	Fth1	ferritin heavy polypeptide 1	14319	0.120959545	1	1	0.002501526	1.69E-07
Caudoputamen	cTBS	Dach1	dachshund family transcription factor 1	13134	0.121595099	0.415	0.312	0.03131483	2.12E-06
Caudoputamen	cTBS	Ppp3ca	protein phosphatase 3, catalytic subunit, alpha isoform	19055	0.121717324	0.999	0.999	0.000819373	5.54E-08
Caudoputamen	cTBS	Atp5mpl	ATP synthase membrane subunit 6.8PL	70257	0.123498108	0.996	0.997	0.000726318	4.91E-08
Caudoputamen	cTBS	mt-Co1	NA	NA	0.124182091	1	1	1.31E-09	8.86E-14
Caudoputamen	cTBS	Hist1h1c	NA	NA	0.125851591	0.348	0.244	0.005506124	3.73E-07
Caudoputamen	cTBS	Sipa1l3	signal-induced proliferation-associated 1 like 3	74206	0.126011996	0.307	0.218	0.036496636	2.47E-06
Caudoputamen	cTBS	Ncdn	neurochondrin	26562	0.127191245	0.999	1	2.41E-05	1.63E-09
Caudoputamen	cTBS	Mn1	meningioma 1	433938	0.12965373	0.328	0.229	0.007459425	5.05E-07
Caudoputamen	cTBS	Snd1	staphylococcal nuclease and tudor domain containing 1	56463	0.129942611	0.279	0.19	0.014501776	9.81E-07
Caudoputamen	cTBS	Celf3	CUGBP, Elav-like family member 3	78784	0.131654606	0.41	0.303	0.011871816	8.03E-07
Caudoputamen	cTBS	Rps27	ribosomal protein S27	57294	0.134115747	0.982	0.977	0.006009891	4.07E-07
Caudoputamen	cTBS	Spock3	sparc/osteonectin, cwcv and kazal-like domains proteoglycan 3	72902	0.134371573	0.885	0.812	0.018842101	1.28E-06
Caudoputamen	cTBS	Ppp1r1b	protein phosphatase 1, regulatory inhibitor subunit 1B	19049	0.135207913	0.999	0.991	0.000603333	4.08E-08
Caudoputamen	cTBS	Vat1l	vesicle amine transport protein 1 like	270097	0.13587445	0.439	0.332	0.025713595	1.74E-06
Caudoputamen	cTBS	Rpl36	ribosomal protein L36	54217	0.136226239	0.997	0.999	6.53E-06	4.42E-10
Caudoputamen	cTBS	Neto2	neuropilin (NRP) and tolloid (TLL)-like 2	74513	0.13642654	0.446	0.333	0.002599465	1.76E-07
Caudoputamen	cTBS	Myo1b	myosin IB	17912	0.136964089	0.308	0.208	0.001503606	1.02E-07
Caudoputamen	cTBS	Ftl1	ferritin light polypeptide 1	14325	0.137730033	0.987	0.991	0.005039147	3.41E-07
Caudoputamen	cTBS	Tmsb10	thymosin, beta 10	19240	0.137739807	1	0.999	4.34E-08	2.94E-12
Caudoputamen	cTBS	Plxnd1	plexin D1	67784	0.13808934	0.371	0.273	0.024517659	1.66E-06
Caudoputamen	cTBS	Adcy5	adenylate cyclase 5	224129	0.139509589	0.96	0.917	0.001481751	1.00E-07
Caudoputamen	cTBS	Foxo1	forkhead box O1	56458	0.140020388	0.465	0.339	0.000424397	2.87E-08
Caudoputamen	cTBS	Prkcb	protein kinase C, beta	18751	0.140629723	0.966	0.945	0.0300527	2.03E-06
Caudoputamen	cTBS	Ppp2r2a	protein phosphatase 2, regulatory subunit B, alpha	71978	0.142169695	0.738	0.632	0.045274031	3.06E-06
Caudoputamen	cTBS	Inf2	inverted formin, FH2 and WH2 domain containing	70435	0.142378088	0.916	0.84	0.004666846	3.16E-07
Caudoputamen	cTBS	Rasd2	RASD family, member 2	75141	0.142989887	0.968	0.935	0.004040561	2.73E-07
Caudoputamen	cTBS	Stub1	STIP1 homology and U-Box containing protein 1	56424	0.143304493	0.61	0.479	0.013303789	9.00E-07
Caudoputamen	cTBS	Rpl35a	ribosomal protein L35A	57808	0.143809726	1	0.996	3.22E-07	2.18E-11
Caudoputamen	cTBS	Ppp1ca	protein phosphatase 1 catalytic subunit alpha	19045	0.14496957	0.985	0.977	0.000326126	2.21E-08
Caudoputamen	cTBS	Lrrc10b	leucine rich repeat containing 10B	278795	0.145100247	0.815	0.707	0.024447141	1.65E-06
Caudoputamen	cTBS	Fnbp1l	formin binding protein 1-like	214459	0.14576677	0.421	0.307	0.001095202	7.41E-08
Caudoputamen	cTBS	Nrgn	neurogranin	64011	0.148216192	1	1	0.00729216	4.93E-07
Caudoputamen	cTBS	Myh10	myosin, heavy polypeptide 10, non-muscle	77579	0.149483725	0.625	0.485	0.000648375	4.39E-08
Caudoputamen	cTBS	Ndufa1	NADH:ubiquinone oxidoreductase subunit A1	54405	0.151282016	0.887	0.843	0.030221999	2.05E-06
Caudoputamen	cTBS	Rps29	ribosomal protein S29	20090	0.152764044	0.999	0.998	3.04E-06	2.05E-10
Caudoputamen	cTBS	Uqcr11	ubiquinol-cytochrome c reductase, complex III subunit XI	66594	0.155508476	1	1	1.67E-08	1.13E-12
Caudoputamen	cTBS	Kcnip2	Kv channel-interacting protein 2	80906	0.156058548	0.856	0.798	0.018835484	1.27E-06
Caudoputamen	cTBS	Tesc	tescalcin	57816	0.156513445	0.921	0.847	0.000742104	5.02E-08
Caudoputamen	cTBS	Dlg4	discs large MAGUK scaffold protein 4	13385	0.15679376	0.739	0.612	0.005891252	3.99E-07
Caudoputamen	cTBS	Atp5md	ATP synthase membrane subunit DAPIT	66477	0.15708449	1	1	6.05E-11	4.09E-15
Caudoputamen	cTBS	Gas5	growth arrest specific 5	14455	0.158489788	0.969	0.944	0.000122458	8.29E-09
Caudoputamen	cTBS	Bcl11b	B cell leukemia/lymphoma 11B	58208	0.160873103	0.738	0.621	0.002306853	1.56E-07
Caudoputamen	cTBS	Rasgef1b	RasGEF domain family, member 1B	320292	0.161114756	0.426	0.31	0.000304541	2.06E-08
Caudoputamen	cTBS	Bex1	brain expressed X-linked 1	19716	0.161306201	0.846	0.763	0.011186952	7.57E-07
Caudoputamen	cTBS	Lmo2	LIM domain only 2	16909	0.162697087	0.49	0.372	0.000412324	2.79E-08
Caudoputamen	cTBS	Rpl23	ribosomal protein L23	65019	0.162824741	0.998	0.996	6.81E-08	4.61E-12
Caudoputamen	cTBS	Cox6c	cytochrome c oxidase subunit 6C	12864	0.16360121	1	1	6.00E-16	4.06E-20
Caudoputamen	cTBS	Hmgn2	high mobility group nucleosomal binding domain 2	15331	0.164094691	0.558	0.443	0.004726878	3.20E-07
Caudoputamen	cTBS	St8sia3	ST8 alpha-N-acetyl-neuraminide alpha-2,8-sialyltransferase 3	20451	0.168704084	0.863	0.765	0.001258466	8.52E-08
Caudoputamen	cTBS	Eno1	enolase 1, alpha non-neuron	13806	0.170108189	0.893	0.864	0.000372961	2.52E-08
Caudoputamen	cTBS	Cox7c	cytochrome c oxidase subunit 7C	12867	0.170578163	1	1	1.37E-13	9.28E-18
Caudoputamen	cTBS	Kctd17	potassium channel tetramerisation domain containing 17	72844	0.177165554	0.834	0.739	0.018578133	1.26E-06
Caudoputamen	cTBS	Drd1	dopamine receptor D1	13488	0.180049329	0.721	0.562	7.33E-07	4.96E-11
Caudoputamen	cTBS	Atp5k	ATP synthase, H+ transporting, mitochondrial F1F0 complex, subunit E	11958	0.184552498	0.998	0.999	2.18E-11	1.48E-15
Caudoputamen	cTBS	Cnr1	cannabinoid receptor 1 (brain)	12801	0.185031018	0.654	0.532	0.001935068	1.31E-07
Caudoputamen	cTBS	Gpr88	G-protein coupled receptor 88	64378	0.185868182	0.993	0.981	7.70E-10	5.21E-14
Caudoputamen	cTBS	Rps28	ribosomal protein S28	54127	0.199990876	0.943	0.935	5.83E-05	3.94E-09
Caudoputamen	cTBS	Rasgrp2	RAS, guanyl releasing protein 2	19395	0.207947872	0.805	0.674	1.83E-06	1.24E-10
Caudoputamen	cTBS	Polr2l	polymerase (RNA) II (DNA directed) polypeptide L	66491	0.208696574	0.507	0.362	2.31E-06	1.57E-10
Caudoputamen	cTBS	Rps21	ribosomal protein S21	66481	0.209174389	1	1	1.42E-22	9.59E-27
Caudoputamen	cTBS	Rgs9	regulator of G-protein signaling 9	19739	0.211492162	0.953	0.896	6.05E-09	4.10E-13
Caudoputamen	cTBS	Ptpn5	protein tyrosine phosphatase, non-receptor type 5	19259	0.212627431	0.986	0.966	1.23E-10	8.31E-15
Caudoputamen	cTBS	Pcp4	Purkinje cell protein 4	18546	0.213168599	1	1	1.47E-12	9.97E-17
Caudoputamen	cTBS	Rpl38	ribosomal protein L38	67671	0.214068141	1	1	1.26E-19	8.51E-24
Caudoputamen	cTBS	AC149090.1	NA	NA	0.234910805	0.727	0.563	1.62E-07	1.10E-11
Caudoputamen	cTBS	Adora2a	adenosine A2a receptor	11540	0.237865396	0.748	0.614	2.48E-07	1.68E-11
Caudoputamen	cTBS	Gstp1	glutathione S-transferase, pi 1	14870	0.355673161	0.472	0.27	4.87E-19	3.30E-23
Caudoputamen	cTBS	Rpl35	ribosomal protein L35	66489	0.404741398	0.983	0.965	1.09E-32	7.36E-37
Caudoputamen	cTBS	Uba52	ubiquitin A-52 residue ribosomal protein fusion product 1	22186	0.506746535	0.826	0.78	6.55E-29	4.43E-33
Caudoputamen	cTBS	Gm10076	ribosomal protein L41 pseudogene	100126819	1.049698447	0.772	0.463	1.59E-94	1.07E-98
Lateral Septal Complex	cTBS	Ttr	transthyretin	22139	-1.951188266	0.101	0.321	6.79E-10	4.60E-14
Lateral Septal Complex	cTBS	Bc1	brain cytoplasmic RNA 1	100568459	-1.114948558	0.585	0.909	2.59E-32	1.75E-36
Lateral Septal Complex	cTBS	Tmem158	transmembrane protein 158	72309	-0.43957192	0.735	0.838	1.26E-06	8.53E-11
Lateral Septal Complex	cTBS	Bok	BCL2-related ovarian killer	51800	-0.406603618	0.477	0.608	0.025701243	1.74E-06
Lateral Septal Complex	cTBS	Necab2	N-terminal EF-hand calcium binding protein 2	117148	-0.395191666	0.686	0.786	0.013799946	9.34E-07
Lateral Septal Complex	cTBS	Gria1	glutamate receptor, ionotropic, AMPA1 (alpha 1)	14799	-0.334501905	0.691	0.815	0.003208812	2.17E-07
Lateral Septal Complex	cTBS	Lrrc17	leucine rich repeat containing 17	74511	-0.323409984	0.119	0.334	6.96E-09	4.71E-13
Lateral Septal Complex	cTBS	Lmo4	LIM domain only 4	16911	-0.315996228	0.317	0.507	0.000422133	2.86E-08
Lateral Septal Complex	cTBS	Cnih2	cornichon family AMPA receptor auxiliary protein 2	12794	-0.289620486	0.92	0.961	0.010041113	6.79E-07
Lateral Septal Complex	cTBS	Calm1	calmodulin 1	12313	-0.258240594	1	1	0.000126483	8.56E-09
Lateral Septal Complex	cTBS	Cdk5r2	cyclin-dependent kinase 5, regulatory subunit 2 (p39)	12570	-0.257546971	0.273	0.441	0.004223403	2.86E-07
Lateral Septal Complex	cTBS	Cadm3	cell adhesion molecule 3	94332	-0.21409173	0.387	0.564	0.038008236	2.57E-06
Lateral Septal Complex	cTBS	Tuba1a	tubulin, alpha 1A	22142	-0.21235979	0.985	0.987	0.005714572	3.87E-07
Lateral Septal Complex	cTBS	Leprotl1	leptin receptor overlapping transcript-like 1	68192	-0.200108197	0.268	0.433	0.044679033	3.02E-06
Lateral Septal Complex	cTBS	Ndnf	neuron-derived neurotrophic factor	68169	-0.181717848	0.057	0.175	0.004308534	2.92E-07
Lateral Septal Complex	cTBS	mt-Cytb	NA	NA	-0.153773853	1	1	0.000795164	5.38E-08
Lateral Septal Complex	cTBS	Fau	Finkel-Biskis-Reilly murine sarcoma virus (FBR-MuSV) ubiquitously expres	14109	0.143072066	1	1	0.012421034	8.41E-07
Lateral Septal Complex	cTBS	Rplp1	ribosomal protein, large, P1	56040	0.15327933	1	1	0.000852162	5.77E-08
Lateral Septal Complex	cTBS	Rpl11	ribosomal protein L11	67025	0.153736104	0.995	1	0.036488634	2.47E-06
Lateral Septal Complex	cTBS	Rpl35a	ribosomal protein L35A	57808	0.167978277	1	1	0.032313001	2.19E-06
Lateral Septal Complex	cTBS	Atpif1	ATPase inhibitory factor 1	11983	0.172157193	1	0.997	0.041395915	2.80E-06
Lateral Septal Complex	cTBS	Uqcr11	ubiquinol-cytochrome c reductase, complex III subunit XI	66594	0.178209474	1	0.992	0.038462064	2.60E-06
Lateral Septal Complex	cTBS	Rpl36	ribosomal protein L36	54217	0.192380129	0.997	1	0.000603615	4.08E-08
Lateral Septal Complex	cTBS	Cox7c	cytochrome c oxidase subunit 7C	12867	0.195564926	1	1	0.00012135	8.21E-09
Lateral Septal Complex	cTBS	Rpl37a	ribosomal protein L37a	19981	0.20717611	1	0.997	0.000409264	2.77E-08
Lateral Septal Complex	cTBS	Igsf1	immunoglobulin superfamily, member 1	209268	0.210282896	0.245	0.11	0.013836464	9.36E-07
Lateral Septal Complex	cTBS	Rpl39	ribosomal protein L39	67248	0.216764026	0.997	0.987	0.007565678	5.12E-07
Lateral Septal Complex	cTBS	Nts	neurotensin	67405	0.22128398	0.541	0.337	0.000963977	6.52E-08
Lateral Septal Complex	cTBS	Rpl37	ribosomal protein L37	67281	0.230142733	1	1	3.02E-10	2.04E-14
Lateral Septal Complex	cTBS	Ndufb1-ps	NA	NA	0.239355893	0.954	0.898	0.008800166	5.95E-07
Lateral Septal Complex	cTBS	Atp5k	ATP synthase, H+ transporting, mitochondrial F1F0 complex, subunit E	11958	0.253587518	0.997	0.987	2.94E-06	1.99E-10
Lateral Septal Complex	cTBS	Cox17	cytochrome c oxidase assembly protein 17, copper chaperone	12856	0.256922182	0.892	0.838	0.003805351	2.58E-07
Lateral Septal Complex	cTBS	Rps21	ribosomal protein S21	66481	0.258429401	1	1	4.75E-12	3.21E-16
Lateral Septal Complex	cTBS	Rps29	ribosomal protein S29	20090	0.268069665	1	1	3.97E-07	2.69E-11
Lateral Septal Complex	cTBS	Rps27	ribosomal protein S27	57294	0.273182668	0.997	0.995	4.76E-08	3.22E-12
Lateral Septal Complex	cTBS	Polr2l	polymerase (RNA) II (DNA directed) polypeptide L	66491	0.326951536	0.624	0.491	0.000841713	5.70E-08
Lateral Septal Complex	cTBS	Rpl38	ribosomal protein L38	67671	0.327903228	1	1	1.23E-21	8.32E-26
Lateral Septal Complex	cTBS	Rps28	ribosomal protein S28	54127	0.329211043	0.995	0.958	8.13E-06	5.50E-10
Lateral Septal Complex	cTBS	Gstp1	glutathione S-transferase, pi 1	14870	0.589590609	0.714	0.449	4.34E-16	2.94E-20
Lateral Septal Complex	cTBS	Rpl35	ribosomal protein L35	66489	0.654136538	0.995	0.948	5.72E-40	3.87E-44
Lateral Septal Complex	cTBS	Uba52	ubiquitin A-52 residue ribosomal protein fusion product 1	22186	0.784122465	0.894	0.705	2.92E-28	1.98E-32
Lateral Septal Complex	cTBS	Gm10076	ribosomal protein L41 pseudogene	100126819	1.305172032	0.861	0.496	7.26E-56	4.92E-60
Striatum Ventral Region	cTBS	Bc1	brain cytoplasmic RNA 1	100568459	-0.926359105	0.48	0.83	8.05E-52	5.45E-56
Striatum Ventral Region	cTBS	Ptgds	prostaglandin D2 synthase (brain)	19215	-0.353222369	0.694	0.79	0.024112355	1.63E-06
Striatum Ventral Region	cTBS	Malat1	metastasis associated lung adenocarcinoma transcript 1 (non-coding RNA	72289	-0.307512671	0.573	0.643	0.017126764	1.16E-06
Striatum Ventral Region	cTBS	Ttr	transthyretin	22139	-0.241571137	0.004	0.126	1.19E-17	8.06E-22
Striatum Ventral Region	cTBS	Atp1a3	ATPase, Na+/K+ transporting, alpha 3 polypeptide	232975	-0.240955314	0.73	0.818	0.000127402	8.62E-09
Striatum Ventral Region	cTBS	Gm42418	NA	NA	-0.197429186	0.92	0.958	0.000910304	6.16E-08
Striatum Ventral Region	cTBS	Snhg9	small nucleolar RNA host gene 9	73474	-0.193927998	0.177	0.304	0.000111408	7.54E-09
Striatum Ventral Region	cTBS	Tatdn1	TatD DNase domain containing 1	69694	-0.169123376	0.129	0.232	0.00278797	1.89E-07
Striatum Ventral Region	cTBS	Lrrc17	leucine rich repeat containing 17	74511	-0.164650636	0.059	0.17	4.56E-07	3.09E-11
Striatum Ventral Region	cTBS	Ywhah	tyrosine 3-monooxygenase/tryptophan 5-monooxygenase activation pro	22629	-0.164605595	0.959	0.974	0.049645714	3.36E-06
Striatum Ventral Region	cTBS	2900097C17Rik	NA	NA	-0.159355239	0.966	0.977	0.025927503	1.75E-06
Striatum Ventral Region	cTBS	Calm1	calmodulin 1	12313	-0.125777514	1	1	0.010214258	6.91E-07
Striatum Ventral Region	cTBS	mt-Atp6	NA	NA	0.110562868	1	1	0.012505683	8.46E-07
Striatum Ventral Region	cTBS	Rpl35a	ribosomal protein L35A	57808	0.12619547	1	1	0.014499159	9.81E-07
Striatum Ventral Region	cTBS	Atp5mpl	ATP synthase membrane subunit 6.8PL	70257	0.139449273	0.996	0.994	0.010923237	7.39E-07
Striatum Ventral Region	cTBS	Rps15	ribosomal protein S15	20054	0.140054271	0.999	0.988	0.024058385	1.63E-06
Striatum Ventral Region	cTBS	Rps29	ribosomal protein S29	20090	0.14129544	0.999	0.998	0.026959812	1.82E-06
Striatum Ventral Region	cTBS	Uqcr11	ubiquinol-cytochrome c reductase, complex III subunit XI	66594	0.155853407	0.999	1	0.004157576	2.81E-07
Striatum Ventral Region	cTBS	Cox8a	cytochrome c oxidase subunit 8A	12868	0.161881783	1	1	2.49E-05	1.68E-09
Striatum Ventral Region	cTBS	Rps21	ribosomal protein S21	66481	0.167631544	1	1	4.35E-06	2.94E-10
Striatum Ventral Region	cTBS	mt-Co1	NA	NA	0.170373687	1	1	3.11E-06	2.11E-10
Striatum Ventral Region	cTBS	Ubb	ubiquitin B	22187	0.172388618	1	1	2.45E-09	1.66E-13
Striatum Ventral Region	cTBS	Cox6a1	cytochrome c oxidase subunit 6A1	12861	0.17622877	0.997	0.992	0.001443039	9.76E-08
Striatum Ventral Region	cTBS	Cox6c	cytochrome c oxidase subunit 6C	12864	0.179055585	1	1	1.08E-10	7.34E-15
Striatum Ventral Region	cTBS	Ndufa3	NADH:ubiquinone oxidoreductase subunit A3	66091	0.180838083	0.979	0.984	0.000323531	2.19E-08
Striatum Ventral Region	cTBS	Rps27	ribosomal protein S27	57294	0.195996845	0.995	0.991	0.000491045	3.32E-08
Striatum Ventral Region	cTBS	Atp5md	ATP synthase membrane subunit DAPIT	66477	0.209517683	1	1	6.38E-13	4.32E-17
Striatum Ventral Region	cTBS	Atp5k	ATP synthase, H+ transporting, mitochondrial F1F0 complex, subunit E	11958	0.214402215	0.999	0.997	7.26E-08	4.91E-12
Striatum Ventral Region	cTBS	Eno1	enolase 1, alpha non-neuron	13806	0.227569433	0.917	0.85	0.00111592	7.55E-08
Striatum Ventral Region	cTBS	Cox7c	cytochrome c oxidase subunit 7C	12867	0.246319387	1	0.998	3.05E-16	2.06E-20
Striatum Ventral Region	cTBS	Rpl38	ribosomal protein L38	67671	0.270207119	1	1	2.98E-24	2.02E-28
Striatum Ventral Region	cTBS	Tmsb10	thymosin, beta 10	19240	0.27410157	1	1	2.87E-17	1.94E-21
Striatum Ventral Region	cTBS	Gstp1	glutathione S-transferase, pi 1	14870	0.308468127	0.546	0.368	4.42E-09	2.99E-13
Striatum Ventral Region	cTBS	Polr2l	polymerase (RNA) II (DNA directed) polypeptide L	66491	0.386345439	0.558	0.341	4.63E-15	3.13E-19
Striatum Ventral Region	cTBS	Rpl35	ribosomal protein L35	66489	0.445589756	0.993	0.972	3.09E-33	2.09E-37
Striatum Ventral Region	cTBS	Uba52	ubiquitin A-52 residue ribosomal protein fusion product 1	22186	0.607126313	0.892	0.832	1.83E-30	1.24E-34
Striatum Ventral Region	cTBS	Gm10076	ribosomal protein L41 pseudogene	100126819	1.120235596	0.866	0.59	2.62E-78	1.78E-82

Data S3. Differentially expressed genes (DEGs) identified in the motor (M1), and somatosensory (SS) cortex following iTBS, and cTBS. DEGs were identified using a cutoff of p.adj ≤ 0.05.

Cortical Region	Cortical Layer	Stimulation	Gene Symbol	Gene Name	EntrezID	Avg log2FoldChange	% expressed in stimulated samples	% expressed in sham samples	p.adjust value (Bonferroni correction)	p.value
M1	Layer 2/3	iTBS	Bc1	brain cytoplasmic RNA 1	100568459	-0.930399046	0.468	0.78	1.53E-16	1.04E-20
M1	Layer 2/3	iTBS	Nme7	NME/NM23 family member 7	171567	-0.633147575	0.266	0.461	0.000888303	6.01E-08
M1	Layer 2/3	iTBS	Cdk5r2	cyclin-dependent kinase 5, regulatory subunit 2 (p39)	12570	-0.532907036	0.365	0.688	7.93E-13	5.37E-17
M1	Layer 2/3	iTBS	Dynll2	dynein light chain LC8-type 2	68097	-0.439269398	0.966	0.979	7.70E-06	5.21E-10
M1	Layer 2/3	iTBS	Cplx2	complexin 2	12890	-0.436041517	0.792	0.869	0.000171026	1.16E-08
M1	Layer 2/3	iTBS	Dnm1	dynamin 1	13429	-0.394078197	0.997	1	4.92E-08	3.33E-12
M1	Layer 2/3	iTBS	Actb	actin, beta	11461	-0.392267123	1	0.993	2.04E-08	1.38E-12
M1	Layer 2/3	iTBS	Syngr1	synaptogyrin 1	20972	-0.385827973	0.836	0.883	0.001770927	1.20E-07
M1	Layer 2/3	iTBS	Glul	glutamate-ammonia ligase (glutamine synthetase)	14645	-0.368222064	0.727	0.865	0.003130555	2.12E-07
M1	Layer 2/3	iTBS	Slc17a7	solute carrier family 17 (sodium-dependent inorganic phosphate cotransporter), member 7	72961	-0.351803462	1	1	0.000604668	4.09E-08
M1	Layer 2/3	iTBS	Eef1a2	eukaryotic translation elongation factor 1 alpha 2	13628	-0.35158734	0.997	0.996	0.001908941	1.29E-07
M1	Layer 2/3	iTBS	Syn1	synapsin I	20964	-0.34867417	0.973	0.972	0.032540862	2.20E-06
M1	Layer 2/3	iTBS	Epn1	epsin 1	13854	-0.344058651	0.874	0.915	0.002160859	1.46E-07
M1	Layer 2/3	iTBS	Tuba1a	tubulin, alpha 1A	22142	-0.314281119	0.997	0.993	3.22E-07	2.18E-11
M1	Layer 2/3	iTBS	Tuba1b	tubulin, alpha 1B	22143	-0.312792785	0.976	0.993	0.000152058	1.03E-08
M1	Layer 2/3	iTBS	Rpl5	ribosomal protein L5	100503670	-0.305948502	0.922	0.943	0.001926034	1.30E-07
M1	Layer 2/3	iTBS	R3hdm4	R3H domain containing 4	109284	-0.303133015	0.652	0.773	0.038279947	2.59E-06
M1	Layer 2/3	iTBS	Syngap1	synaptic Ras GTPase activating protein 1 homolog (rat)	240057	-0.286678646	0.215	0.433	0.000223026	1.51E-08
M1	Layer 2/3	iTBS	Ikbkb	inhibitor of kappaB kinase beta	16150	-0.27390344	0.28	0.493	0.001960654	1.33E-07
M1	Layer 2/3	iTBS	Ldha	lactate dehydrogenase A	16828	-0.265521606	0.908	0.943	0.014357214	9.72E-07
M1	Layer 2/3	iTBS	Atp6v0e2	ATPase, H+ transporting, lysosomal V0 subunit E2	76252	-0.26004892	0.887	0.95	0.0048681	3.29E-07
M1	Layer 2/3	iTBS	Dctn4	dynactin 4	67665	-0.237092875	0.283	0.475	0.024083992	1.63E-06
M1	Layer 2/3	iTBS	Fosl2	fos-like antigen 2	14284	-0.225587125	0.181	0.351	0.045901191	3.11E-06
M1	Layer 2/3	iTBS	Lrrc17	leucine rich repeat containing 17	74511	-0.194317382	0.109	0.259	0.047829164	3.24E-06
M1	Layer 2/3	iTBS	Calm1	calmodulin 1	12313	-0.186409037	1	1	2.13E-05	1.44E-09
M1	Layer 2/3	iTBS	Rpl41	ribosomal protein L41	67945	-0.164504145	1	1	0.002231487	1.51E-07
M1	Layer 2/3	iTBS	Gnas	GNAS (guanine nucleotide binding protein, alpha stimulating) complex locus	14683	0.156001223	1	1	0.040515152	2.74E-06
M1	Layer 2/3	iTBS	Cox8a	cytochrome c oxidase subunit 8A	12868	0.161527293	1	1	0.019583383	1.33E-06
M1	Layer 2/3	iTBS	Atp1b1	ATPase, Na+/K+ transporting, beta 1 polypeptide	11931	0.174638194	1	1	0.023429173	1.59E-06
M1	Layer 2/3	iTBS	Cox6c	cytochrome c oxidase subunit 6C	12864	0.194249754	1	1	1.73E-06	1.17E-10
M1	Layer 2/3	iTBS	Fth1	ferritin heavy polypeptide 1	14319	0.197613997	1	1	3.04E-09	2.06E-13
M1	Layer 2/3	iTBS	Rpl37	ribosomal protein L37	67281	0.20874891	1	1	0.00635227	4.30E-07
M1	Layer 2/3	iTBS	Rpl24	ribosomal protein L24	68193	0.21520329	0.993	0.975	0.013278223	8.99E-07
M1	Layer 2/3	iTBS	Rpl21	ribosomal protein L21	19933	0.220984135	1	0.996	5.42E-05	3.67E-09
M1	Layer 2/3	iTBS	Rpl11	ribosomal protein L11	67025	0.221751834	0.997	0.979	0.001588468	1.07E-07
M1	Layer 2/3	iTBS	Tmsb4x	thymosin, beta 4, X chromosome	19241	0.224602885	1	1	5.33E-08	3.60E-12
M1	Layer 2/3	iTBS	Rpl13	ribosomal protein L13	270106	0.233635205	1	1	4.70E-06	3.18E-10
M1	Layer 2/3	iTBS	Cox7c	cytochrome c oxidase subunit 7C	12867	0.234243641	1	1	0.000221573	1.50E-08
M1	Layer 2/3	iTBS	Rps7	ribosomal protein S7	20115	0.243048301	0.997	0.979	0.007034415	4.76E-07
M1	Layer 2/3	iTBS	Rps24	ribosomal protein S24	20088	0.252146286	1	0.993	2.13E-05	1.44E-09
M1	Layer 2/3	iTBS	Rps10	ribosomal protein S10	67097	0.256160549	0.99	0.94	0.004997984	3.38E-07
M1	Layer 2/3	iTBS	Rps27a	ribosomal protein S27A	78294	0.262082632	1	0.986	0.001235155	8.36E-08
M1	Layer 2/3	iTBS	Rpl30	ribosomal protein L30	19946	0.266937423	0.993	0.936	0.00383604	2.60E-07
M1	Layer 2/3	iTBS	Rpl23	ribosomal protein L23	65019	0.273637269	1	0.975	3.09E-05	2.09E-09
M1	Layer 2/3	iTBS	Rps21	ribosomal protein S21	66481	0.281924237	1	0.996	5.16E-07	3.49E-11
M1	Layer 2/3	iTBS	Rps8	ribosomal protein S8	20116	0.289422095	0.99	0.982	9.05E-06	6.13E-10
M1	Layer 2/3	iTBS	Junb	jun B proto-oncogene	16477	0.298010412	0.659	0.457	0.001060932	7.18E-08
M1	Layer 2/3	iTBS	Rpl9	ribosomal protein L9	20005	0.323306121	1	0.979	2.01E-08	1.36E-12
M1	Layer 2/3	iTBS	Nr4a1	nuclear receptor subfamily 4, group A, member 1	15370	0.325322467	0.761	0.631	0.004682657	3.17E-07
M1	Layer 2/3	iTBS	Rpl35	ribosomal protein L35	66489	0.428949855	0.969	0.851	2.05E-08	1.39E-12
M1	Layer 2/3	iTBS	Gstp1	glutathione S-transferase, pi 1	14870	0.433633298	0.515	0.248	2.29E-08	1.55E-12
M1	Layer 2/3	iTBS	Uba52	ubiquitin A-52 residue ribosomal protein fusion product 1	22186	0.996141378	0.86	0.592	3.20E-29	2.17E-33
M1	Layer 2/3	iTBS	Gm10076	ribosomal protein L41 pseudogene	100126819	1.454574365	0.819	0.301	2.76E-46	1.87E-50
M1	Layer 5	iTBS	Bc1	brain cytoplasmic RNA 1	100568459	-0.556184087	0.49	0.73	1.43E-16	9.67E-21
M1	Layer 5	iTBS	Cplx2	complexin 2	12890	-0.547573969	0.645	0.762	5.93E-12	4.01E-16
M1	Layer 5	iTBS	Dynll2	dynein light chain LC8-type 2	68097	-0.361690156	0.959	0.977	1.27E-07	8.62E-12
M1	Layer 5	iTBS	Cdk5r2	cyclin-dependent kinase 5, regulatory subunit 2 (p39)	12570	-0.332047506	0.402	0.587	3.69E-08	2.50E-12
M1	Layer 5	iTBS	Hpcal4	hippocalcin-like 4	170638	-0.326978757	0.656	0.805	5.63E-05	3.81E-09
M1	Layer 5	iTBS	Eef1a2	eukaryotic translation elongation factor 1 alpha 2	13628	-0.313475648	1	0.998	4.66E-08	3.15E-12
M1	Layer 5	iTBS	Dnm1	dynamin 1	13429	-0.309624637	0.998	1	5.74E-09	3.88E-13
M1	Layer 5	iTBS	Tuba1b	tubulin, alpha 1B	22143	-0.304908539	0.967	0.987	8.58E-12	5.80E-16
M1	Layer 5	iTBS	Glul	glutamate-ammonia ligase (glutamine synthetase)	14645	-0.293099767	0.815	0.872	5.70E-05	3.85E-09
M1	Layer 5	iTBS	Stx1b	syntaxin 1B	56216	-0.293057921	0.828	0.874	4.40E-05	2.98E-09
M1	Layer 5	iTBS	Ndrg2	N-myc downstream regulated gene 2	29811	-0.289289769	0.875	0.917	9.89E-05	6.70E-09
M1	Layer 5	iTBS	Sptbn2	spectrin beta, non-erythrocytic 2	20743	-0.28839933	0.809	0.865	0.009945331	6.73E-07
M1	Layer 5	iTBS	Actb	actin, beta	11461	-0.286079493	0.994	0.994	1.22E-06	8.25E-11
M1	Layer 5	iTBS	Phyhip	phytanoyl-CoA hydroxylase interacting protein	105653	-0.284007447	0.427	0.583	2.62E-05	1.77E-09
M1	Layer 5	iTBS	Kcnq2	potassium voltage-gated channel, subfamily Q, member 2	16536	-0.268063584	0.573	0.692	0.003602513	2.44E-07
M1	Layer 5	iTBS	Slc17a7	solute carrier family 17 (sodium-dependent inorganic phosphate cotransporter), member 7	72961	-0.267703515	0.996	0.996	0.003462609	2.34E-07
M1	Layer 5	iTBS	Rpl5	ribosomal protein L5	100503670	-0.264821637	0.923	0.962	6.73E-06	4.55E-10
M1	Layer 5	iTBS	Ivns1abp	influenza virus NS1A binding protein	117198	-0.26005929	0.456	0.589	0.014667245	9.93E-07
M1	Layer 5	iTBS	Epn1	epsin 1	13854	-0.257900631	0.846	0.893	0.009364102	6.34E-07
M1	Layer 5	iTBS	Emc10	ER membrane protein complex subunit 10	69683	-0.253231918	0.815	0.863	0.000401949	2.72E-08
M1	Layer 5	iTBS	Dusp26	dual specificity phosphatase 26 (putative)	66959	-0.252448117	0.388	0.559	5.24E-05	3.54E-09
M1	Layer 5	iTBS	Eno2	enolase 2, gamma neuronal	13807	-0.247646972	0.969	0.983	0.000173344	1.17E-08
M1	Layer 5	iTBS	Thy1	thymus cell antigen 1, theta	21838	-0.243059102	0.969	0.996	1.46E-06	9.88E-11
M1	Layer 5	iTBS	Brsk1	BR serine/threonine kinase 1	381979	-0.241335178	0.454	0.595	0.001171609	7.93E-08
M1	Layer 5	iTBS	Add2	adducin 2 (beta)	11519	-0.23948343	0.38	0.523	0.00162507	1.10E-07
M1	Layer 5	iTBS	Ikbkb	inhibitor of kappaB kinase beta	16150	-0.236972258	0.292	0.445	0.000435446	2.95E-08
M1	Layer 5	iTBS	Atp6v0a1	ATPase, H+ transporting, lysosomal V0 subunit A1	11975	-0.236710063	0.77	0.842	0.035703825	2.42E-06
M1	Layer 5	iTBS	Lars2	leucyl-tRNA synthetase, mitochondrial	102436	-0.233238855	0.257	0.426	3.95E-05	2.67E-09
M1	Layer 5	iTBS	Pde1b	phosphodiesterase 1B, Ca2+-calmodulin dependent	18574	-0.230292745	0.313	0.452	0.008075804	5.46E-07
M1	Layer 5	iTBS	Mt2	metallothionein 2	17750	-0.229777887	0.434	0.57	0.026209186	1.77E-06
M1	Layer 5	iTBS	Tuba1a	tubulin, alpha 1A	22142	-0.229620706	0.983	0.998	4.60E-06	3.12E-10
M1	Layer 5	iTBS	Rpl4	ribosomal protein L4	67891	-0.207869565	0.99	0.994	0.000111034	7.51E-09
M1	Layer 5	iTBS	Reep5	receptor accessory protein 5	13476	-0.206299967	0.859	0.906	0.044111617	2.98E-06
M1	Layer 5	iTBS	Dync1h1	dynein cytoplasmic 1 heavy chain 1	13424	-0.203017145	0.884	0.916	0.043027686	2.91E-06
M1	Layer 5	iTBS	Agap2	ArfGAP with GTPase domain, ankyrin repeat and PH domain 2	216439	-0.202242686	0.216	0.37	0.000391815	2.65E-08
M1	Layer 5	iTBS	Eef2	eukaryotic translation elongation factor 2	13629	-0.199167071	0.983	0.987	0.028233603	1.91E-06
M1	Layer 5	iTBS	Gnb1	guanine nucleotide binding protein (G protein), beta 1	14688	-0.189714784	0.958	0.981	0.001067638	7.22E-08
M1	Layer 5	iTBS	Clu	clusterin	12759	-0.187903639	0.961	0.981	0.030902846	2.09E-06
M1	Layer 5	iTBS	Ppp1r1b	protein phosphatase 1, regulatory inhibitor subunit 1B	19049	-0.164769876	0.108	0.216	0.027527854	1.86E-06
M1	Layer 5	iTBS	Aldoa	aldolase A, fructose-bisphosphate	11674	-0.144867399	1	1	0.000269084	1.82E-08
M1	Layer 5	iTBS	Cpe	carboxypeptidase E	12876	-0.140047726	1	1	0.005531318	3.74E-07
M1	Layer 5	iTBS	Hspa8	heat shock protein 8	15481	-0.115305427	1	1	0.03114526	2.11E-06
M1	Layer 5	iTBS	Rpl41	ribosomal protein L41	67945	-0.114442923	1	1	0.018975084	1.28E-06
M1	Layer 5	iTBS	Lhpp	phospholysine phosphohistidine inorganic pyrophosphate phosphatase	76429	0.125199691	0.153	0.064	0.045745145	3.10E-06
M1	Layer 5	iTBS	Calm2	calmodulin 2	12314	0.13354441	1	1	0.000733871	4.97E-08
M1	Layer 5	iTBS	Acyp2	acylphosphatase 2, muscle type	75572	0.142744163	0.27	0.152	0.041372707	2.80E-06
M1	Layer 5	iTBS	Ppia	peptidylprolyl isomerase A	268373	0.148260658	1	1	6.35E-05	4.29E-09
M1	Layer 5	iTBS	Gnas	GNAS (guanine nucleotide binding protein, alpha stimulating) complex locus	14683	0.148758834	1	1	0.00026691	1.81E-08
M1	Layer 5	iTBS	Rpl11	ribosomal protein L11	67025	0.149550676	1	0.989	0.012046139	8.15E-07
M1	Layer 5	iTBS	Fth1	ferritin heavy polypeptide 1	14319	0.153169848	1	1	6.19E-06	4.19E-10
M1	Layer 5	iTBS	Atp1b1	ATPase, Na+/K+ transporting, beta 1 polypeptide	11931	0.15400753	1	1	0.0328506	2.22E-06
M1	Layer 5	iTBS	Cox8a	cytochrome c oxidase subunit 8A	12868	0.154790004	1	1	1.27E-05	8.56E-10
M1	Layer 5	iTBS	Rpl21	ribosomal protein L21	19933	0.162458094	1	1	1.86E-06	1.26E-10
M1	Layer 5	iTBS	Tmsb4x	thymosin, beta 4, X chromosome	19241	0.163983452	1	1	5.56E-07	3.76E-11
M1	Layer 5	iTBS	Rpl37	ribosomal protein L37	67281	0.165846849	1	1	0.00056436	3.82E-08
M1	Layer 5	iTBS	Rtn1	reticulon 1	104001	0.168887932	0.998	0.994	0.003120554	2.11E-07
M1	Layer 5	iTBS	Ifit3	interferon-induced protein with tetratricopeptide repeats 3	15959	0.171970043	0.124	0.024	9.10E-06	6.16E-10
M1	Layer 5	iTBS	Rps7	ribosomal protein S7	20115	0.172569875	0.992	0.977	0.001613082	1.09E-07
M1	Layer 5	iTBS	Cox6c	cytochrome c oxidase subunit 6C	12864	0.176906952	1	1	1.03E-07	6.98E-12
M1	Layer 5	iTBS	Selenok	selenoprotein K	80795	0.179591186	0.9	0.786	0.010586322	7.16E-07
M1	Layer 5	iTBS	Rps27a	ribosomal protein S27A	78294	0.180190549	1	0.998	0.000186452	1.26E-08
M1	Layer 5	iTBS	Rpl36a	ribosomal protein L36A	19982	0.18125957	0.898	0.824	0.009950042	6.73E-07
M1	Layer 5	iTBS	Rpl13	ribosomal protein L13	270106	0.187210724	1	0.998	7.40E-07	5.01E-11
M1	Layer 5	iTBS	Dusp1	dual specificity phosphatase 1	19252	0.190441152	0.523	0.381	0.025391256	1.72E-06
M1	Layer 5	iTBS	Rps20	ribosomal protein S20	67427	0.19284623	0.996	0.998	2.94E-05	1.99E-09
M1	Layer 5	iTBS	Efhd2	EF hand domain containing 2	27984	0.197545986	0.89	0.863	0.038633185	2.61E-06
M1	Layer 5	iTBS	Ndufb9	NADH:ubiquinone oxidoreductase subunit B9	66218	0.201520228	0.996	0.977	1.08E-05	7.29E-10
M1	Layer 5	iTBS	Chd9	chromodomain helicase DNA binding protein 9	109151	0.205439686	0.394	0.238	0.000557506	3.77E-08
M1	Layer 5	iTBS	Rpl35a	ribosomal protein L35A	57808	0.207090584	0.994	0.977	0.001319519	8.93E-08
M1	Layer 5	iTBS	Rps21	ribosomal protein S21	66481	0.210037112	1	0.996	6.99E-07	4.73E-11
M1	Layer 5	iTBS	Eno1	enolase 1, alpha non-neuron	13806	0.236439726	0.965	0.904	0.000837028	5.66E-08
M1	Layer 5	iTBS	Cox7c	cytochrome c oxidase subunit 7C	12867	0.251816906	1	0.998	1.95E-11	1.32E-15
M1	Layer 5	iTBS	Tmsb10	thymosin, beta 10	19240	0.266888129	0.998	0.996	6.54E-10	4.42E-14
M1	Layer 5	iTBS	Rpl9	ribosomal protein L9	20005	0.28607227	0.998	0.983	1.75E-13	1.18E-17
M1	Layer 5	iTBS	Pvalb	parvalbumin	19293	0.29439878	0.838	0.762	0.021682193	1.47E-06
M1	Layer 5	iTBS	Rps8	ribosomal protein S8	20116	0.298601597	1	0.994	7.97E-15	5.39E-19
M1	Layer 5	iTBS	Rpl35	ribosomal protein L35	66489	0.366759307	0.981	0.901	3.65E-12	2.47E-16
M1	Layer 5	iTBS	Gstp1	glutathione S-transferase, pi 1	14870	0.389247318	0.537	0.31	2.86E-12	1.94E-16
M1	Layer 5	iTBS	Uba52	ubiquitin A-52 residue ribosomal protein fusion product 1	22186	0.779851038	0.861	0.664	5.64E-35	3.82E-39
M1	Layer 5	iTBS	Gm10076	ribosomal protein L41 pseudogene	100126819	1.28664674	0.764	0.321	6.03E-70	4.08E-74
M1	Layer 6	iTBS	Eef1a2	eukaryotic translation elongation factor 1 alpha 2	13628	-0.507722247	0.979	0.995	2.37E-05	1.60E-09
M1	Layer 6	iTBS	Ppia	peptidylprolyl isomerase A	268373	0.214189038	1	1	0.004501071	3.05E-07
M1	Layer 6	iTBS	Cox6c	cytochrome c oxidase subunit 6C	12864	0.222728066	1	1	0.001136761	7.69E-08

M1	Layer 6	iTBS	Cox5b	cytochrome c oxidase subunit 5B	12859	0.240869199	1	1	0.007105391	4.81E-07
M1	Layer 6	iTBS	Calm2	calmodulin 2	12314	0.248976302	1	1	0.015082718	1.02E-06
M1	Layer 6	iTBS	Rpl38	ribosomal protein L38	67671	0.261461166	1	1	0.002561907	1.73E-07
M1	Layer 6	iTBS	Ndufb9	NADH:ubiquinone oxidoreductase subunit B9	66218	0.280285928	1	0.984	0.039632005	2.68E-06
M1	Layer 6	iTBS	Rps8	ribosomal protein S8	20116	0.293572287	0.995	0.995	0.00213008	1.44E-07
M1	Layer 6	iTBS	Sytl2	synaptotagmin-like 2	83671	0.31277338	0.351	0.141	0.017684192	1.20E-06
M1	Layer 6	iTBS	Rpl35	ribosomal protein L35	66489	0.337141937	0.995	0.979	0.001271736	8.61E-08
M1	Layer 6	iTBS	Gstp1	glutathione S-transferase, pi 1	14870	0.644680111	0.644	0.344	1.63E-07	1.10E-11
M1	Layer 6	iTBS	Uba52	ubiquitin A-52 residue ribosomal protein fusion product 1	22186	0.774490163	0.91	0.776	5.78E-12	3.91E-16
M1	Layer 6	iTBS	Gm10076	ribosomal protein L41 pseudogene	100126819	1.224857727	0.793	0.49	4.21E-18	2.85E-22
M1	Layer 2/3	cTBS	Bc1	brain cytoplasmic RNA 1	100568459	-1.093216524	0.382	0.78	1.21E-20	8.18E-25
M1	Layer 2/3	cTBS	Ttr	transthyretin	22139	-0.737194838	0.013	0.234	1.61E-09	1.09E-13
M1	Layer 2/3	cTBS	Cdk5r2	cyclin-dependent kinase 5, regulatory subunit 2 (p39)	12570	-0.348726088	0.508	0.688	0.007100877	4.81E-07
M1	Layer 2/3	cTBS	Tatdn1	TatD DNase domain containing 1	69694	-0.309512891	0.126	0.358	1.18E-05	7.97E-10
M1	Layer 2/3	cTBS	Basp1	brain abundant, membrane attached signal protein 1	70350	-0.306187338	0.807	0.947	0.002924508	1.98E-07
M1	Layer 2/3	cTBS	Tuba1a	tubulin, alpha 1A	22142	-0.300088442	0.992	0.993	2.38E-05	1.61E-09
M1	Layer 2/3	cTBS	Tuba1b	tubulin, alpha 1B	22143	-0.29944023	0.987	0.993	0.00228783	1.55E-07
M1	Layer 2/3	cTBS	Ikbkb	inhibitor of kappaB kinase beta	16150	-0.267576532	0.277	0.493	0.009503442	6.43E-07
M1	Layer 2/3	cTBS	Fam207a	family with sequence similarity 207, member A	108707	-0.25483742	0.092	0.287	0.00039857	2.70E-08
M1	Layer 2/3	cTBS	Lrrc17	leucine rich repeat containing 17	74511	-0.243106445	0.076	0.259	0.000559054	3.78E-08
M1	Layer 2/3	cTBS	Cck	cholecystokinin	12424	-0.213469143	1	1	0.001061602	7.18E-08
M1	Layer 2/3	cTBS	Calm1	calmodulin 1	12313	-0.182311629	1	1	0.000312125	2.11E-08
M1	Layer 2/3	cTBS	Rpl38	ribosomal protein L38	67671	0.202913413	1	1	0.001661876	1.12E-07
M1	Layer 2/3	cTBS	Uqcr11	ubiquinol-cytochrome c reductase, complex III subunit XI	66594	0.26341501	1	0.996	0.000354382	2.40E-08
M1	Layer 2/3	cTBS	Atp5k	ATP synthase, H+ transporting, mitochondrial F1F0 complex, subunit E	11958	0.287120899	1	0.989	9.85E-05	6.67E-09
M1	Layer 2/3	cTBS	Eno1	enolase 1, alpha non-neuron	13806	0.327195749	0.962	0.894	0.003632396	2.46E-07
M1	Layer 2/3	cTBS	Gstp1	glutathione S-transferase, pi 1	14870	0.344800544	0.458	0.248	0.001363282	9.23E-08
M1	Layer 2/3	cTBS	Rpl35	ribosomal protein L35	66489	0.586394679	0.966	0.851	8.56E-14	5.79E-18
M1	Layer 2/3	cTBS	Uba52	ubiquitin A-52 residue ribosomal protein fusion product 1	22186	0.671484509	0.79	0.592	9.79E-12	6.63E-16
M1	Layer 2/3	cTBS	Gm10076	ribosomal protein L41 pseudogene	100126819	1.208843802	0.777	0.301	1.07E-33	7.24E-38
M1	Layer 5	cTBS	Bc1	brain cytoplasmic RNA 1	100568459	-0.719464524	0.359	0.73	1.30E-31	8.80E-36
M1	Layer 5	cTBS	Ttr	transthyretin	22139	-0.478572331	0.026	0.216	8.28E-18	5.60E-22
M1	Layer 5	cTBS	Ikbkb	inhibitor of kappaB kinase beta	16150	-0.322632072	0.226	0.445	5.89E-11	3.99E-15
M1	Layer 5	cTBS	Atp1a3	ATPase, Na+/K+ transporting, alpha 3 polypeptide	232975	-0.319363151	0.804	0.919	1.66E-07	1.12E-11
M1	Layer 5	cTBS	Cdk5r2	cyclin-dependent kinase 5, regulatory subunit 2 (p39)	12570	-0.306716563	0.403	0.587	3.65E-07	2.47E-11
M1	Layer 5	cTBS	Basp1	brain abundant, membrane attached signal protein 1	70350	-0.281820053	0.804	0.946	8.32E-08	5.63E-12
M1	Layer 5	cTBS	Tuba1b	tubulin, alpha 1B	22143	-0.248692868	0.982	0.987	7.27E-08	4.92E-12
M1	Layer 5	cTBS	Tuba1a	tubulin, alpha 1A	22142	-0.240953294	0.989	0.998	6.54E-08	4.43E-12
M1	Layer 5	cTBS	Arpc4	actin related protein 2/3 complex, subunit 4	68089	-0.239991566	0.527	0.664	0.003285191	2.22E-07
M1	Layer 5	cTBS	Trf	transferrin	22041	-0.232608624	0.379	0.522	0.02200046	1.49E-06
M1	Layer 5	cTBS	Huwe1	HECT, UBA and WWE domain containing 1	59026	-0.228818034	0.351	0.512	0.000256833	1.74E-08
M1	Layer 5	cTBS	Phyhip	phytanoyl-CoA hydroxylase interacting protein	105653	-0.226607491	0.436	0.583	0.002388576	1.62E-07
M1	Layer 5	cTBS	Tubb4a	tubulin, beta 4A class IVA	22153	-0.2195663	0.845	0.906	0.01016501	6.88E-07
M1	Layer 5	cTBS	Snhg9	small nucleolar RNA host gene 9	73474	-0.217932787	0.129	0.278	1.21E-05	8.19E-10
M1	Layer 5	cTBS	Brsk1	BR serine/threonine kinase 1	381979	-0.212463507	0.455	0.595	0.008287477	5.61E-07
M1	Layer 5	cTBS	Rnf141	ring finger protein 141	67150	-0.201042481	0.205	0.341	0.003097472	2.10E-07
M1	Layer 5	cTBS	Spcs2	signal peptidase complex subunit 2 homolog (S. cerevisiae)	66624	-0.192693091	0.329	0.473	0.011924516	8.07E-07
M1	Layer 5	cTBS	Ogdhl	oxoglutarate dehydrogenase-like	239017	-0.185221114	0.211	0.347	0.005023209	3.40E-07
M1	Layer 5	cTBS	Agap2	ArfGAP with GTPase domain, ankyrin repeat and PH domain 2	216439	-0.179837681	0.214	0.37	0.001035746	7.01E-08
M1	Layer 5	cTBS	Tm9sf4	transmembrane 9 superfamily member 4	99237	-0.179108921	0.203	0.33	0.017742338	1.20E-06
M1	Layer 5	cTBS	Satb2	special AT-rich sequence binding protein 2	212712	-0.178311074	0.222	0.36	0.014816336	1.00E-06
M1	Layer 5	cTBS	Mrpl46	mitochondrial ribosomal protein L46	67308	-0.177401165	0.255	0.411	0.001741992	1.18E-07
M1	Layer 5	cTBS	Tatdn1	TatD DNase domain containing 1	69694	-0.174666698	0.131	0.251	0.005190664	3.51E-07
M1	Layer 5	cTBS	Snrpn	small nuclear ribonucleoprotein N	20646	-0.170364311	0.993	0.998	0.014978059	1.01E-06
M1	Layer 5	cTBS	Snrpc	U1 small nuclear ribonucleoprotein C	20630	-0.166254775	0.287	0.43	0.039617137	2.68E-06
M1	Layer 5	cTBS	Dapk3	death-associated protein kinase 3	13144	-0.16588297	0.17	0.296	0.013920735	9.42E-07
M1	Layer 5	cTBS	Gm2000	ribosomal protein L35 pseudogene	100038991	-0.164618003	0.172	0.3	0.013379906	9.05E-07
M1	Layer 5	cTBS	Arxes2	adipocyte-related X-chromosome expressed sequence 2	76976	-0.159595338	0.194	0.321	0.034604954	2.34E-06
M1	Layer 5	cTBS	Abca3	ATP-binding cassette, sub-family A (ABC1), member 3	27410	-0.153237316	0.305	0.45	0.046781662	3.17E-06
M1	Layer 5	cTBS	Tmem131	transmembrane protein 131	56030	-0.149817158	0.107	0.212	0.032777275	2.22E-06
M1	Layer 5	cTBS	Srrd	SRR1 domain containing	70118	-0.136970278	0.12	0.233	0.025889537	1.75E-06
M1	Layer 5	cTBS	Atp6v0c	ATPase, H+ transporting, lysosomal V0 subunit C	11984	-0.128913468	1	1	0.020457049	1.38E-06
M1	Layer 5	cTBS	Vwa1	von Willebrand factor A domain containing 1	246228	-0.108080746	0.063	0.152	0.048344574	3.27E-06
M1	Layer 5	cTBS	Ubb	ubiquitin B	22187	0.119925642	1	1	0.017822487	1.21E-06
M1	Layer 5	cTBS	Gnas	GNAS (guanine nucleotide binding protein, alpha stimulating) complex locus	14683	0.139352927	1	1	0.034704465	2.35E-06
M1	Layer 5	cTBS	Rpl37	ribosomal protein L37	67281	0.157092073	1	1	0.003991945	2.70E-07
M1	Layer 5	cTBS	Rpl23	ribosomal protein L23	65019	0.164047075	0.998	0.994	0.005270975	3.57E-07
M1	Layer 5	cTBS	Cox6c	cytochrome c oxidase subunit 6C	12864	0.169505587	1	1	1.82E-06	1.23E-10
M1	Layer 5	cTBS	Atp5k	ATP synthase, H+ transporting, mitochondrial F1F0 complex, subunit E	11958	0.175772193	1	0.996	0.005783073	3.91E-07
M1	Layer 5	cTBS	Rpl35a	ribosomal protein L35A	57808	0.185588177	0.994	0.977	0.006611856	4.47E-07
M1	Layer 5	cTBS	Rpl26	ribosomal protein L26	19941	0.185707853	0.987	0.977	0.013785048	9.33E-07
M1	Layer 5	cTBS	Atp5mpl	ATP synthase membrane subunit 6.8PL	70257	0.186919866	0.998	0.998	0.00147888	1.00E-07
M1	Layer 5	cTBS	Rpl31	ribosomal protein L31	114641	0.193842014	0.98	0.962	0.008484951	5.74E-07
M1	Layer 5	cTBS	Gas5	growth arrest specific 5	14455	0.213537528	0.935	0.88	0.002772999	1.88E-07
M1	Layer 5	cTBS	Atp5md	ATP synthase membrane subunit DAPIT	66477	0.226176535	1	1	5.81E-10	3.93E-14
M1	Layer 5	cTBS	Rps21	ribosomal protein S21	66481	0.226694256	1	0.996	5.19E-08	3.51E-12
M1	Layer 5	cTBS	Rpl38	ribosomal protein L38	67671	0.233839864	1	1	5.84E-13	3.95E-17
M1	Layer 5	cTBS	Rps28	ribosomal protein S28	54127	0.23759416	0.9	0.842	0.008353489	5.65E-07
M1	Layer 5	cTBS	Cox7c	cytochrome c oxidase subunit 7C	12867	0.255584577	0.998	0.998	5.10E-11	3.45E-15
M1	Layer 5	cTBS	Rpl37a	ribosomal protein L37a	19981	0.255790516	0.994	0.972	5.59E-06	3.78E-10
M1	Layer 5	cTBS	Eno1	enolase 1, alpha non-neuron	13806	0.281712608	0.963	0.904	3.11E-06	2.11E-10
M1	Layer 5	cTBS	Rps27	ribosomal protein S27	57294	0.287594688	0.965	0.878	1.61E-07	1.09E-11
M1	Layer 5	cTBS	Rps29	ribosomal protein S29	20090	0.304107573	0.996	0.983	3.60E-13	2.43E-17
M1	Layer 5	cTBS	Gstp1	glutathione S-transferase, pi 1	14870	0.341951511	0.477	0.31	1.18E-06	7.99E-11
M1	Layer 5	cTBS	Uba52	ubiquitin A-52 residue ribosomal protein fusion product 1	22186	0.621487028	0.828	0.664	1.45E-24	9.79E-29
M1	Layer 5	cTBS	Rpl35	ribosomal protein L35	66489	0.629817196	0.987	0.901	4.94E-41	3.34E-45
M1	Layer 5	cTBS	Gm10076	ribosomal protein L41 pseudogene	100126819	1.19205091	0.774	0.321	2.29E-69	1.55E-73
M1	Layer 6	cTBS	Bc1	brain cytoplasmic RNA 1	100568459	-0.667902783	0.335	0.688	3.32E-09	2.24E-13
M1	Layer 6	cTBS	Ttr	transthyretin	22139	-0.410667093	0.014	0.177	9.96E-05	6.74E-09
M1	Layer 6	cTBS	Cox8a	cytochrome c oxidase subunit 8A	12868	0.235235903	1	1	0.042436011	2.87E-06
M1	Layer 6	cTBS	Fth1	ferritin heavy polypeptide 1	14319	0.242242604	1	1	0.000538103	3.64E-08
M1	Layer 6	cTBS	Cox6c	cytochrome c oxidase subunit 6C	12864	0.257019884	1	1	2.94E-05	1.99E-09
M1	Layer 6	cTBS	Atp5md	ATP synthase membrane subunit DAPIT	66477	0.267543112	1	1	0.000177199	1.20E-08
M1	Layer 6	cTBS	Rpl38	ribosomal protein L38	67671	0.286612031	1	1	2.36E-05	1.60E-09
M1	Layer 6	cTBS	Pcp4	Purkinje cell protein 4	18546	0.325715802	1	1	0.011314731	7.66E-07
M1	Layer 6	cTBS	Ftl1	ferritin light polypeptide 1	14325	0.332260911	0.991	0.964	0.013163305	8.91E-07
M1	Layer 6	cTBS	Rpl35	ribosomal protein L35	66489	0.484942586	1	0.979	4.17E-08	2.82E-12
M1	Layer 6	cTBS	Uba52	ubiquitin A-52 residue ribosomal protein fusion product 1	22186	0.562418974	0.824	0.776	0.000970955	6.57E-08
M1	Layer 6	cTBS	Gm10076	ribosomal protein L41 pseudogene	100126819	0.822078401	0.719	0.49	4.71E-10	3.19E-14
SS	Layer 2/3	iTBS	Bc1	brain cytoplasmic RNA 1	100568459	-0.680503187	0.551	0.726	3.47E-06	2.35E-10
SS	Layer 2/3	iTBS	Malat1	metastasis associated lung adenocarcinoma transcript 1 (non-coding RNA)	72289	-0.420450995	0.508	0.627	0.033937586	2.30E-06
SS	Layer 2/3	iTBS	Nme7	NME/NM23 family member 7	171567	-0.401414215	0.195	0.363	0.030227141	2.05E-06
SS	Layer 2/3	iTBS	Calm1	calmodulin 1	12313	-0.212412049	1	1	6.81E-09	4.61E-13
SS	Layer 2/3	iTBS	Dele1	DAP3 binding cell death enhancer 1	66839	0.194303666	0.215	0.076	0.01577846	1.07E-06
SS	Layer 2/3	iTBS	Rpl21	ribosomal protein L21	19933	0.208596149	1	1	0.000145878	9.87E-09
SS	Layer 2/3	iTBS	Atp1a1	ATPase, Na+/K+ transporting, alpha 1 polypeptide	11928	0.221337962	1	1	0.037275638	2.52E-06
SS	Layer 2/3	iTBS	Gtdc1	glycosyltransferase-like domain containing 1	227835	0.24961389	0.33	0.165	0.023433461	1.59E-06
SS	Layer 2/3	iTBS	Psd	pleckstrin and Sec7 domain containing	73728	0.258455079	0.967	0.842	0.027841205	1.88E-06
SS	Layer 2/3	iTBS	Rpl35	ribosomal protein L35	66489	0.278652367	0.974	0.947	0.002592361	1.75E-07
SS	Layer 2/3	iTBS	Bsg	basigin	12215	0.287372453	0.977	0.931	0.000262858	1.78E-08
SS	Layer 2/3	iTBS	Adcy1	adenylate cyclase 1	432530	0.320730341	0.825	0.726	0.001107199	7.49E-08
SS	Layer 2/3	iTBS	Gstp1	glutathione S-transferase, pi 1	14870	0.362570079	0.446	0.244	0.000247185	1.67E-08
SS	Layer 2/3	iTBS	Uba52	ubiquitin A-52 residue ribosomal protein fusion product 1	22186	0.710738094	0.848	0.64	5.15E-16	3.49E-20
SS	Layer 2/3	iTBS	Gm10076	ribosomal protein L41 pseudogene	100126819	1.068215366	0.756	0.419	1.36E-25	9.20E-30
SS	Layer 4	iTBS	Malat1	metastasis associated lung adenocarcinoma transcript 1 (non-coding RNA)	72289	-0.575145043	0.466	0.681	1.05E-08	7.12E-13
SS	Layer 4	iTBS	Bc1	brain cytoplasmic RNA 1	100568459	-0.418345608	0.355	0.563	0.001108452	7.50E-08
SS	Layer 4	iTBS	Tuba1b	tubulin, alpha 1B	22143	-0.297419376	0.938	0.987	0.000397833	2.69E-08
SS	Layer 4	iTBS	Ndufa4	Ndufa4, mitochondrial complex associated	17992	-0.266486561	1	1	0.003414701	2.31E-07
SS	Layer 4	iTBS	Snhg9	small nucleolar RNA host gene 9	73474	-0.264506064	0.098	0.271	0.000302944	2.05E-08
SS	Layer 4	iTBS	Tuba1a	tubulin, alpha 1A	22142	-0.2454036	0.99	0.983	0.003586035	2.43E-07
SS	Layer 4	iTBS	Fau	Finkel-Biskis-Reilly murine sarcoma virus (FBR-MuSV) ubiquitously expressed (fox derived)	14109	0.210565538	1	1	0.008676153	5.87E-07
SS	Layer 4	iTBS	Rps21	ribosomal protein S21	66481	0.217634338	1	1	0.001070903	7.25E-08
SS	Layer 4	iTBS	Camk2n1	calcium/calmodulin-dependent protein kinase II inhibitor 1	66259	0.265511261	1	1	1.96E-06	1.32E-10
SS	Layer 4	iTBS	Atp1b1	ATPase, Na+/K+ transporting, beta 1 polypeptide	11931	0.277359798	1	1	1.43E-05	9.66E-10
SS	Layer 4	iTBS	Gstp1	glutathione S-transferase, pi 1	14870	0.384704024	0.435	0.188	1.29E-05	8.70E-10
SS	Layer 4	iTBS	Rpl35	ribosomal protein L35	66489	0.431538062	0.987	0.943	6.57E-11	4.45E-15
SS	Layer 4	iTBS	Uba52	ubiquitin A-52 residue ribosomal protein fusion product 1	22186	0.794795851	0.909	0.721	2.43E-20	1.65E-24
SS	Layer 4	iTBS	Gm10076	ribosomal protein L41 pseudogene	100126819	1.192706911	0.806	0.393	2.58E-32	1.74E-36
SS	Layer 5	iTBS	Bc1	brain cytoplasmic RNA 1	100568459	-0.436738846	0.462	0.598	0.025236626	1.71E-06
SS	Layer 5	iTBS	Cdk5r2	cyclin-dependent kinase 5, regulatory subunit 2 (p39)	12570	-0.279423366	0.294	0.481	0.002802952	1.90E-07
SS	Layer 5	iTBS	Aldoc	aldolase C, fructose-bisphosphate	11676	-0.241461448	1	1	0.000945711	6.40E-08
SS	Layer 5	iTBS	Rpl41	ribosomal protein L41	67945	-0.178229734	1	1	0.000275532	1.86E-08
SS	Layer 5	iTBS	Ppp2cb	protein phosphatase 2 (formerly 2A), catalytic subunit, beta isoform	19053	0.167270377	0.189	0.059	0.001520583	1.03E-07
SS	Layer 5	iTBS	Ubb	ubiquitin B	22187	0.244768106	1	1	4.90E-05	3.32E-09
SS	Layer 5	iTBS	Rpl35	ribosomal protein L35	66489	0.274108668	0.991	0.96	0.000102629	6.94E-09
SS	Layer 5	iTBS	Uba52	ubiquitin A-52 residue ribosomal protein fusion product 1	22186	0.52930806	0.869	0.753	4.14E-09	2.80E-13
SS	Layer 5	iTBS	Gm10076	ribosomal protein L41 pseudogene	100126819	0.965664168	0.747	0.41	2.39E-25	1.62E-29
SS	Layer 6	iTBS	Bc1	brain cytoplasmic RNA 1	100568459	-0.515050145	0.429	0.667	1.14E-08	7.74E-13
SS	Layer 6	iTBS	Tuba1b	tubulin, alpha 1B	22143	-0.283839882	0.945	0.989	0.000186732	1.26E-08

SS	Layer 6	iTBS	Ppia	peptidylprolyl isomerase A	268373	0.160489702	1	1	0.003705946	2.51E-07
SS	Layer 6	iTBS	Rpl21	ribosomal protein L21	19933	0.176249392	1	1	0.007535071	5.10E-07
SS	Layer 6	iTBS	Stmn1	stathmin 1	16765	0.182839472	1	1	0.02124185	1.44E-06
SS	Layer 6	iTBS	Tmsb10	thymosin, beta 10	19240	0.197270893	1	1	0.028219683	1.91E-06
SS	Layer 6	iTBS	Rps24	ribosomal protein S24	20088	0.204761193	1	1	0.000780749	5.28E-08
SS	Layer 6	iTBS	Rps7	ribosomal protein S7	20115	0.205370161	1	0.997	0.0049521	3.35E-07
SS	Layer 6	iTBS	Fau	Finkel-Biskis-Reilly murine sarcoma virus (FBR-MuSV) ubiquitously expressed (fox derived)	14109	0.208450971	1	1	0.001305517	8.83E-08
SS	Layer 6	iTBS	Ccm2	cerebral cavernous malformation 2	216527	0.209186611	0.199	0.08	0.029351529	1.99E-06
SS	Layer 6	iTBS	Rpl13	ribosomal protein L13	270106	0.221383199	1	1	8.76E-06	5.93E-10
SS	Layer 6	iTBS	Rpl9	ribosomal protein L9	20005	0.228466136	0.995	0.992	0.000223451	1.51E-08
SS	Layer 6	iTBS	Gpx4	glutathione peroxidase 4	625249	0.236944967	1	1	0.000298738	2.02E-08
SS	Layer 6	iTBS	Rps27a	ribosomal protein S27A	78294	0.244873531	1	1	2.48E-05	1.68E-09
SS	Layer 6	iTBS	Rps10	ribosomal protein S10	67097	0.250814978	0.997	0.979	0.003173887	2.15E-07
SS	Layer 6	iTBS	Chd9	chromodomain helicase DNA binding protein 9	109151	0.268061223	0.325	0.144	9.88E-05	6.69E-09
SS	Layer 6	iTBS	Eno1	enolase 1, alpha non-neuron	13806	0.280086245	0.963	0.939	0.002393972	1.62E-07
SS	Layer 6	iTBS	Rps20	ribosomal protein S20	67427	0.281960799	1	0.997	7.63E-08	5.16E-12
SS	Layer 6	iTBS	Rpl35	ribosomal protein L35	66489	0.36946784	0.995	0.979	3.96E-10	2.68E-14
SS	Layer 6	iTBS	Gstp1	glutathione S-transferase, pi 1	14870	0.53023204	0.513	0.28	1.95E-08	1.32E-12
SS	Layer 6	iTBS	Uba52	ubiquitin A-52 residue ribosomal protein fusion product 1	22186	0.690898808	0.911	0.837	7.11E-17	4.81E-21
SS	Layer 6	iTBS	Gm10076	ribosomal protein L41 pseudogene	100126819	0.966468934	0.783	0.563	7.94E-22	5.37E-26
SS	Layer 2/3	cTBS	Bc1	brain cytoplasmic RNA 1	100568459	-0.934349372	0.348	0.726	2.36E-15	1.59E-19
SS	Layer 2/3	cTBS	Ikbkb	inhibitor of kappaB kinase beta	16150	-0.308652092	0.235	0.416	0.021137299	1.43E-06
SS	Layer 2/3	cTBS	Ttr	transthyretin	22139	-0.252681043	0	0.122	0.000202003	1.37E-08
SS	Layer 2/3	cTBS	Lrrc17	leucine rich repeat containing 17	74511	-0.216446063	0.045	0.185	0.009168588	6.20E-07
SS	Layer 2/3	cTBS	Camk2n1	calcium/calmodulin-dependent protein kinase II inhibitor 1	66259	0.16716948	1	1	0.006776046	4.59E-07
SS	Layer 2/3	cTBS	Psd	pleckstrin and Sec7 domain containing	73728	0.288558604	0.955	0.842	0.000261706	1.77E-08
SS	Layer 2/3	cTBS	Rpl35	ribosomal protein L35	66489	0.3468535	0.98	0.947	0.001526864	1.03E-07
SS	Layer 2/3	cTBS	Uba52	ubiquitin A-52 residue ribosomal protein fusion product 1	22186	0.49596171	0.737	0.64	0.001616433	1.09E-07
SS	Layer 2/3	cTBS	Gm10076	ribosomal protein L41 pseudogene	100126819	0.825540773	0.704	0.419	4.18E-15	2.83E-19
SS	Layer 4	cTBS	Pcp4	Purkinje cell protein 4	18546	-0.556809893	0.604	0.755	0.001595321	1.08E-07
SS	Layer 4	cTBS	Bc1	brain cytoplasmic RNA 1	100568459	-0.553197494	0.266	0.563	2.10E-08	1.42E-12
SS	Layer 4	cTBS	Ttr	transthyretin	22139	-0.294428991	0.015	0.162	7.06E-07	4.78E-11
SS	Layer 4	cTBS	Rplp1	ribosomal protein, large, P1	56040	0.19543282	1	1	0.04727985	3.20E-06
SS	Layer 4	cTBS	Rps21	ribosomal protein S21	66481	0.213574472	1	1	0.007008716	4.74E-07
SS	Layer 4	cTBS	Atp1b1	ATPase, Na+/K+ transporting, beta 1 polypeptide	11931	0.216766582	1	1	0.024394179	1.65E-06
SS	Layer 4	cTBS	Ncdn	neurochondrin	26562	0.263969948	1	0.978	0.003990921	2.70E-07
SS	Layer 4	cTBS	Camk2n2	calcium/calmodulin-dependent protein kinase II inhibitor 2	73047	0.275621869	0.908	0.843	0.015427158	1.04E-06
SS	Layer 4	cTBS	Camk2n1	calcium/calmodulin-dependent protein kinase II inhibitor 1	66259	0.29066586	1	1	5.87E-10	3.97E-14
SS	Layer 4	cTBS	Rpl35	ribosomal protein L35	66489	0.415216089	0.97	0.943	1.54E-06	1.04E-10
SS	Layer 4	cTBS	Gm10076	ribosomal protein L41 pseudogene	100126819	0.727480247	0.666	0.393	9.26E-12	6.27E-16
SS	Layer 5	cTBS	Bc1	brain cytoplasmic RNA 1	100568459	-0.553883679	0.364	0.598	1.01E-07	6.82E-12
SS	Layer 5	cTBS	Ttr	transthyretin	22139	-0.248566082	0.003	0.125	8.56E-07	5.79E-11
SS	Layer 5	cTBS	Rplp1	ribosomal protein, large, P1	56040	0.171374173	1	1	0.041136227	2.78E-06
SS	Layer 5	cTBS	Rpl38	ribosomal protein L38	67671	0.171794203	1	1	0.00326368	2.21E-07
SS	Layer 5	cTBS	Rpl35	ribosomal protein L35	66489	0.392826794	0.977	0.96	1.30E-09	8.77E-14
SS	Layer 5	cTBS	Uba52	ubiquitin A-52 residue ribosomal protein fusion product 1	22186	0.399234149	0.787	0.753	0.044643899	3.02E-06
SS	Layer 5	cTBS	Gm10076	ribosomal protein L41 pseudogene	100126819	0.762610332	0.685	0.41	2.01E-17	1.36E-21
SS	Layer 6	cTBS	Bc1	brain cytoplasmic RNA 1	100568459	-0.643015917	0.357	0.667	2.24E-14	1.51E-18
SS	Layer 6	cTBS	Rps21	ribosomal protein S21	66481	0.187773003	1	1	0.020796938	1.41E-06
SS	Layer 6	cTBS	Fth1	ferritin heavy polypeptide 1	14319	0.248986853	1	1	3.74E-06	2.53E-10
SS	Layer 6	cTBS	Rpl35	ribosomal protein L35	66489	0.287112276	0.972	0.979	0.012213146	8.26E-07
SS	Layer 6	cTBS	Eno1	enolase 1, alpha non-neuron	13806	0.297044684	0.966	0.939	0.00125099	8.47E-08
SS	Layer 6	cTBS	Gstp1	glutathione S-transferase, pi 1	14870	0.463396118	0.498	0.28	6.39E-06	4.32E-10
SS	Layer 6	cTBS	Gm10076	ribosomal protein L41 pseudogene	100126819	0.648563877	0.738	0.563	2.05E-12	1.39E-16

Data S4. Motor (M1) cortex layer-specific differentially expressed genes (DEGs) identified in aged mice 3h following iTBS, and cTBS.

M1 layer	Stimulation	Gene Symbol	Gene Name	EntrezID	Avg log2FoldChange	% expressed in stimulated samples	% expressed in sham samples	p.adjust value (Bonferroni correction)	p.value	Higher-order groups
Layer 2/3	iTBS	Gm10076	ribosomal protein L41 pseudogene	100126819	1.454574365	0.819	0.301	2.76E-46	1.87E-50	
Layer 2/3	iTBS	Uba52	ubiquitin A-52 residue ribosomal protein fusion product 1	22186	0.996141378	0.86	0.592	3.20E-29	2.17E-33	
Layer 2/3	iTBS	Gstp1	glutathione S-transferase, pi 1	14870	0.433633298	0.515	0.248	2.29E-08	1.55E-12	
Layer 2/3	iTBS	Rpl35	ribosomal protein L35	66489	0.428949855	0.969	0.851	2.05E-08	1.39E-12	
Layer 2/3	iTBS	Nr4a1	nuclear receptor subfamily 4, group A, member 1	15370	0.325322467	0.761	0.631	0.004682657	3.17E-07	1
Layer 2/3	iTBS	Rpl9	ribosomal protein L9	20005	0.323306121	1	0.979	2.01E-08	1.36E-12	
Layer 2/3	iTBS	Junb	jun B proto-oncogene	16477	0.298010412	0.659	0.457	0.001060932	7.18E-08	
Layer 2/3	iTBS	Rps8	ribosomal protein S8	20116	0.289422095	0.99	0.982	9.05E-06	6.13E-10	
Layer 2/3	iTBS	Rps21	ribosomal protein S21	66481	0.281924237	1	0.996	5.16E-07	3.49E-11	
Layer 2/3	iTBS	Rpl23	ribosomal protein L23	65019	0.273637269	1	0.975	3.09E-05	2.09E-09	
Layer 2/3	iTBS	Rpl30	ribosomal protein L30	19946	0.266937423	0.993	0.936	0.00383604	2.60E-07	
Layer 2/3	iTBS	Rps27a	ribosomal protein S27A	78294	0.262082632	1	0.986	0.001235155	8.36E-08	
Layer 2/3	iTBS	Rps10	ribosomal protein S10	67097	0.256160549	0.99	0.94	0.004997984	3.38E-07	
Layer 2/3	iTBS	Rps24	ribosomal protein S24	20088	0.252146286	1	0.993	2.13E-05	1.44E-09	
Layer 2/3	iTBS	Atp6v0e2	ATPase, H+ transporting, lysosomal V0 subunit E2	76252	-0.26004892	0.887	0.95	0.0048681	3.29E-07	
Layer 2/3	iTBS	Ldha	lactate dehydrogenase A	16828	-0.265521606	0.908	0.943	0.014357214	9.72E-07	
Layer 2/3	iTBS	Ikbkb	inhibitor of kappaB kinase beta	16150	-0.27390344	0.28	0.493	0.001960654	1.33E-07	7
Layer 2/3	iTBS	Syngap1	synaptic Ras GTPase activating protein 1 homolog (rat)	240057	-0.286678646	0.215	0.433	0.000223026	1.51E-08	1
Layer 2/3	iTBS	R3hdm4	R3H domain containing 4	109284	-0.303133015	0.652	0.773	0.038279947	2.59E-06	
Layer 2/3	iTBS	Rpl5	ribosomal protein L5	100503670	-0.305948502	0.922	0.943	0.001926034	1.30E-07	
Layer 2/3	iTBS	Tuba1b	tubulin, alpha 1B	22143	-0.312792785	0.976	0.993	0.000152058	1.03E-08	
Layer 2/3	iTBS	Tuba1a	tubulin, alpha 1A	22142	-0.314281119	0.997	0.993	3.22E-07	2.18E-11	1
Layer 2/3	iTBS	Epn1	epsin 1	13854	-0.344058651	0.874	0.915	0.002160859	1.46E-07	
Layer 2/3	iTBS	Syn1	synapsin I	20964	-0.34867417	0.973	0.972	0.032540862	2.20E-06	1
Layer 2/3	iTBS	Eef1a2	eukaryotic translation elongation factor 1 alpha 2	13628	-0.35158734	0.997	0.996	0.001908941	1.29E-07	
Layer 2/3	iTBS	Slc17a7	solute carrier family 17 (sodium-dependent inorganic phosphate cotransporter), member 7	72961	-0.351803462	1	1	0.000604668	4.09E-08	1
Layer 2/3	iTBS	Glul	glutamate-ammonia ligase (glutamine synthetase)	14645	-0.368222064	0.727	0.865	0.003130555	2.12E-07	1
Layer 2/3	iTBS	Syngr1	synaptogyrin 1	20972	-0.385827973	0.836	0.883	0.001770927	1.20E-07	1
Layer 2/3	iTBS	Actb	actin, beta	11461	-0.392267123	1	0.993	2.04E-08	1.38E-12	
Layer 2/3	iTBS	Dnm1	dynamin 1	13429	-0.394078197	0.997	1	4.92E-08	3.33E-12	1
Layer 2/3	iTBS	Cplx2	complexin 2	12890	-0.436041517	0.792	0.869	0.000171026	1.16E-08	1
Layer 2/3	iTBS	Dynll2	dynein light chain LC8-type 2	68097	-0.439269398	0.966	0.979	7.70E-06	5.21E-10	
Layer 2/3	iTBS	Cdk5r2	cyclin-dependent kinase 5, regulatory subunit 2 (p39)	12570	-0.532907036	0.365	0.688	7.93E-13	5.37E-17	
Layer 2/3	iTBS	Nme7	NME/NM23 family member 7	171567	-0.633147575	0.266	0.461	0.000888303	6.01E-08	
Layer 2/3	iTBS	Bc1	brain cytoplasmic RNA 1	100568459	-0.930399046	0.468	0.78	1.53E-16	1.04E-20	1
Layer 5	iTBS	Gm10076	ribosomal protein L41 pseudogene	100126819	1.28664674	0.764	0.321	6.03E-70	4.08E-74	
Layer 5	iTBS	Uba52	ubiquitin A-52 residue ribosomal protein fusion product 1	22186	0.779851038	0.861	0.664	5.64E-35	3.82E-39	
Layer 5	iTBS	Gstp1	glutathione S-transferase, pi 1	14870	0.389247318	0.537	0.31	2.86E-12	1.94E-16	
Layer 5	iTBS	Rpl35	ribosomal protein L35	66489	0.366759307	0.981	0.901	3.65E-12	2.47E-16	
Layer 5	iTBS	Rps8	ribosomal protein S8	20116	0.298601597	1	0.994	7.97E-15	5.39E-19	
Layer 5	iTBS	Pvalb	parvalbumin	19293	0.29439878	0.838	0.762	0.021682193	1.47E-06	1
Layer 5	iTBS	Rpl9	ribosomal protein L9	20005	0.28607227	0.998	0.983	1.75E-13	1.18E-17	
Layer 5	iTBS	Tmsb10	thymosin, beta 10	19240	0.266888129	0.998	0.996	6.54E-10	4.42E-14	
Layer 5	iTBS	Cox7c	cytochrome c oxidase subunit 7C	12867	0.251816906	1	0.998	1.95E-11	1.32E-15	
Layer 5	iTBS	Dusp26	dual specificity phosphatase 26 (putative)	66959	-0.252448117	0.388	0.559	5.24E-05	3.54E-09	
Layer 5	iTBS	Emc10	ER membrane protein complex subunit 10	69683	-0.253231918	0.815	0.863	0.000401949	2.72E-08	
Layer 5	iTBS	Epn1	epsin 1	13854	-0.257900631	0.846	0.893	0.009364102	6.34E-07	
Layer 5	iTBS	Ivns1abp	influenza virus NS1A binding protein	117198	-0.26005929	0.456	0.589	0.014667245	9.93E-07	
Layer 5	iTBS	Rpl5	ribosomal protein L5	100503670	-0.264821637	0.923	0.962	6.73E-06	4.55E-10	
Layer 5	iTBS	Slc17a7	solute carrier family 17 (sodium-dependent inorganic phosphate cotransporter), member 7	72961	-0.267703515	0.996	0.996	0.003462609	2.34E-07	1
Layer 5	iTBS	Kcnq2	potassium voltage-gated channel, subfamily Q, member 2	16536	-0.268063584	0.573	0.692	0.003602513	2.44E-07	3
Layer 5	iTBS	Phyhip	phytanoyl-CoA hydroxylase interacting protein	105653	-0.284007447	0.427	0.583	2.62E-05	1.77E-09	
Layer 5	iTBS	Actb	actin, beta	11461	-0.286079493	0.994	0.994	1.22E-06	8.25E-11	
Layer 5	iTBS	Sptbn2	spectrin beta, non-erythrocytic 2	20743	-0.28839933	0.809	0.865	0.009945331	6.73E-07	
Layer 5	iTBS	Ndrg2	N-myc downstream regulated gene 2	29811	-0.289289769	0.875	0.917	9.89E-05	6.70E-09	
Layer 5	iTBS	Stx1b	syntaxin 1B	56216	-0.293057921	0.828	0.874	4.40E-05	2.98E-09	1
Layer 5	iTBS	Glul	glutamate-ammonia ligase (glutamine synthetase)	14645	-0.293099767	0.815	0.872	5.70E-05	3.85E-09	1
Layer 5	iTBS	Tuba1b	tubulin, alpha 1B	22143	-0.304908539	0.967	0.987	8.58E-12	5.80E-16	
Layer 5	iTBS	Dnm1	dynamin 1	13429	-0.309624637	0.998	1	5.74E-09	3.88E-13	1
Layer 5	iTBS	Eef1a2	eukaryotic translation elongation factor 1 alpha 2	13628	-0.313475648	1	0.998	4.66E-08	3.15E-12	
Layer 5	iTBS	Hpcal4	hippocalcin-like 4	170638	-0.326978757	0.656	0.805	5.63E-05	3.81E-09	
Layer 5	iTBS	Cdk5r2	cyclin-dependent kinase 5, regulatory subunit 2 (p39)	12570	-0.332047506	0.402	0.587	3.69E-08	2.50E-12	
Layer 5	iTBS	Dynll2	dynein light chain LC8-type 2	68097	-0.361690156	0.959	0.977	1.27E-07	8.62E-12	
Layer 5	iTBS	Cplx2	complexin 2	12890	-0.547573969	0.645	0.762	5.93E-12	4.01E-16	1
Layer 5	iTBS	Bc1	brain cytoplasmic RNA 1	100568459	-0.556184087	0.49	0.73	1.43E-16	9.67E-21	1
Layer 6	iTBS	Gm10076	ribosomal protein L41 pseudogene	100126819	1.224857727	0.793	0.49	4.21E-18	2.85E-22	
Layer 6	iTBS	Uba52	ubiquitin A-52 residue ribosomal protein fusion product 1	22186	0.774490163	0.91	0.776	5.78E-12	3.91E-16	
Layer 6	iTBS	Gstp1	glutathione S-transferase, pi 1	14870	0.644680111	0.644	0.344	1.63E-07	1.10E-11	
Layer 6	iTBS	Rpl35	ribosomal protein L35	66489	0.337141937	0.995	0.979	0.001271736	8.61E-08	
Layer 6	iTBS	Sytl2	synaptotagmin-like 2	83671	0.31277338	0.351	0.141	0.017684192	1.20E-06	
Layer 6	iTBS	Rps8	ribosomal protein S8	20116	0.293572287	0.995	0.995	0.00213008	1.44E-07	
Layer 6	iTBS	Ndufb9	NADH:ubiquinone oxidoreductase subunit B9	66218	0.280285928	1	0.984	0.039632005	2.68E-06	
Layer 6	iTBS	Rpl38	ribosomal protein L38	67671	0.261461166	1	1	0.002561907	1.73E-07	
Layer 6	iTBS	Eef1a2	eukaryotic translation elongation factor 1 alpha 2	13628	-0.507722247	0.979	0.995	2.37E-05	1.60E-09	
Layer 2/3	cTBS	Gm10076	ribosomal protein L41 pseudogene	100126819	1.208843802	0.777	0.301	1.07E-33	7.24E-38	
Layer 2/3	cTBS	Uba52	ubiquitin A-52 residue ribosomal protein fusion product 1	22186	0.671484509	0.79	0.592	9.79E-12	6.63E-16	
Layer 2/3	cTBS	Rpl35	ribosomal protein L35	66489	0.586394679	0.966	0.851	8.56E-14	5.79E-18	
Layer 2/3	cTBS	Gstp1	glutathione S-transferase, pi 1	14870	0.344800544	0.458	0.248	0.001363282	9.23E-08	
Layer 2/3	cTBS	Eno1	enolase 1, alpha non-neuron	13806	0.327195749	0.962	0.894	0.003632396	2.46E-07	
Layer 2/3	cTBS	Atp5k	ATP synthase, H+ transporting, mitochondrial F1F0 complex, subunit E	11958	0.287120899	1	0.989	9.85E-05	6.67E-09	
Layer 2/3	cTBS	Uqcr11	ubiquinol-cytochrome c reductase, complex III subunit XI	66594	0.26341501	1	0.996	0.000354382	2.40E-08	
Layer 2/3	cTBS	Fam207a	family with sequence similarity 207, member A	108707	-0.25483742	0.092	0.287	0.00039857	2.70E-08	
Layer 2/3	cTBS	Ikbkb	inhibitor of kappaB kinase beta	16150	-0.267576532	0.277	0.493	0.009503442	6.43E-07	7
Layer 2/3	cTBS	Tuba1b	tubulin, alpha 1B	22143	-0.29944023	0.987	0.993	0.00228783	1.55E-07	
Layer 2/3	cTBS	Tuba1a	tubulin, alpha 1A	22142	-0.300088442	0.992	0.993	2.38E-05	1.61E-09	1
Layer 2/3	cTBS	Basp1	brain abundant, membrane attached signal protein 1	70350	-0.306187338	0.807	0.947	0.002924508	1.98E-07	1
Layer 2/3	cTBS	Tatdn1	TatD DNase domain containing 1	69694	-0.309512891	0.126	0.358	1.18E-05	7.97E-10	
Layer 2/3	cTBS	Cdk5r2	cyclin-dependent kinase 5, regulatory subunit 2 (p39)	12570	-0.348726088	0.508	0.688	0.007100877	4.81E-07	
Layer 2/3	cTBS	Ttr	transthyretin	22139	-0.737194838	0.013	0.234	1.61E-09	1.09E-13	1
Layer 2/3	cTBS	Bc1	brain cytoplasmic RNA 1	100568459	-1.093216524	0.382	0.78	1.21E-20	8.18E-25	1
Layer 5	cTBS	Gm10076	ribosomal protein L41 pseudogene	100126819	1.19205091	0.774	0.321	2.29E-69	1.55E-73	
Layer 5	cTBS	Rpl35	ribosomal protein L35	66489	0.629817196	0.987	0.901	4.94E-41	3.34E-45	
Layer 5	cTBS	Uba52	ubiquitin A-52 residue ribosomal protein fusion product 1	22186	0.621487028	0.828	0.664	1.45E-24	9.79E-29	
Layer 5	cTBS	Gstp1	glutathione S-transferase, pi 1	14870	0.341951511	0.477	0.31	1.18E-06	7.99E-11	
Layer 5	cTBS	Rps29	ribosomal protein S29	20090	0.304107573	0.996	0.983	3.60E-13	2.43E-17	
Layer 5	cTBS	Rps27	ribosomal protein S27	57294	0.287594688	0.965	0.878	1.61E-07	1.09E-11	
Layer 5	cTBS	Eno1	enolase 1, alpha non-neuron	13806	0.281712608	0.963	0.904	3.11E-06	2.11E-10	
Layer 5	cTBS	Rpl37a	ribosomal protein L37a	19981	0.255790516	0.994	0.972	5.59E-06	3.78E-10	
Layer 5	cTBS	Cox7c	cytochrome c oxidase subunit 7C	12867	0.255584577	0.998	0.998	5.10E-11	3.45E-15	
Layer 5	cTBS	Basp1	brain abundant, membrane attached signal protein 1	70350	-0.281820053	0.804	0.946	8.32E-08	5.63E-12	1
Layer 5	cTBS	Cdk5r2	cyclin-dependent kinase 5, regulatory subunit 2 (p39)	12570	-0.306716563	0.403	0.587	3.65E-07	2.47E-11	
Layer 5	cTBS	Atp1a3	ATPase, Na+/K+ transporting, alpha 3 polypeptide	232975	-0.319363151	0.804	0.919	1.66E-07	1.12E-11	3
Layer 5	cTBS	Ikbkb	inhibitor of kappaB kinase beta	16150	-0.322632072	0.226	0.445	5.89E-11	3.99E-15	7
Layer 5	cTBS	Ttr	transthyretin	22139	-0.478572331	0.026	0.216	8.28E-18	5.60E-22	1
Layer 5	cTBS	Bc1	brain cytoplasmic RNA 1	100568459	-0.719464524	0.359	0.73	1.30E-31	8.80E-36	1
Layer 6	cTBS	Gm10076	ribosomal protein L41 pseudogene	100126819	0.822078401	0.719	0.49	4.71E-10	3.19E-14	
Layer 6	cTBS	Uba52	ubiquitin A-52 residue ribosomal protein fusion product 1	22186	0.562418974	0.824	0.776	0.000970955	6.57E-08	
Layer 6	cTBS	Rpl35	ribosomal protein L35	66489	0.484942586	1	0.979	4.17E-08	2.82E-12	
Layer 6	cTBS	Ftl1	ferritin light polypeptide 1	14325	0.332260911	0.991	0.964	0.013163305	8.91E-07	
Layer 6	cTBS	Pcp4	Purkinje cell protein 4	18546	0.325715802	1	1	0.011314731	7.66E-07	1
Layer 6	cTBS	Rpl38	ribosomal protein L38	67671	0.286612031	1	1	2.36E-05	1.60E-09	
Layer 6	cTBS	Atp5md	ATP synthase membrane subunit DAPIT	66477	0.267543112	1	1	0.000177199	1.20E-08	
Layer 6	cTBS	Cox6c	cytochrome c oxidase subunit 6C	12864	0.257019884	1	1	2.94E-05	1.99E-09	
Layer 6	cTBS	Ttr	transthyretin	22139	-0.410667093	0.014	0.177	9.96E-05	6.74E-09	1
Layer 6	cTBS	Bc1	brain cytoplasmic RNA 1	100568459	-0.667902783	0.335	0.688	3.32E-09	2.24E-13	1

Data S5. Complete list of enriched Gene Ontology (GO) terms for the significant differentially expressed genes (DEGs) identified in the different motor (M1) cortex layers, following iTBS, and cTBS.

M1 layer	Stimulation	GO ID	GO term description	Number of genes	Gene symbols	p.value	q.value	Ontology Source	Code
Layer 2/3	iTBS	GO:0022626	cytosolic ribosome	11	Uba52/Rpl9/Rp	8.50E-19	5.19E-17	CC	8
Layer 2/3	iTBS	GO:0044391	ribosomal subunit	11	Uba52/Rpl9/Rp	4.05E-16	1.24E-14	CC	8
Layer 2/3	iTBS	GO:0005840	ribosome	11	Uba52/Rpl9/Rp	3.46E-15	7.04E-14	CC	8
Layer 2/3	iTBS	GO:0003735	structural constituent of ribosome	11	Uba52/Rpl9/Rp	4.88E-17	4.32E-15	MF	8
Layer 2/3	iTBS	GO:0043209	myelin sheath	9	Uba52/Actb/Dr	3.54E-12	5.40E-11	CC	4
Layer 2/3	iTBS	GO:0098793	presynapse	8	Actb/Dnm1/Cpl	1.00E-07	6.81E-07	CC	1
Layer 2/3	iTBS	GO:0042254	ribosome biogenesis	6	Rpl35/Rps21/Rp	1.13E-06	0.000579396	BP	8
Layer 2/3	iTBS	GO:0022613	ribonucleoprotein complex biogenesis	6	Rpl35/Rps21/Rp	7.60E-06	0.000776279	BP	8
Layer 2/3	iTBS	GO:0022627	cytosolic small ribosomal subunit	6	Uba52/Rps21/R	2.09E-11	2.55E-10	CC	8
Layer 2/3	iTBS	GO:0022625	cytosolic large ribosomal subunit	6	Uba52/Rpl9/Rp	1.30E-10	1.32E-09	CC	8
Layer 2/3	iTBS	GO:0015935	small ribosomal subunit	6	Uba52/Rps21/R	5.67E-10	4.95E-09	CC	8
Layer 2/3	iTBS	GO:0015934	large ribosomal subunit	6	Uba52/Rpl9/Rp	7.36E-09	5.62E-08	CC	8
Layer 2/3	iTBS	GO:0006364	rRNA processing	5	Rpl35/Rps21/Rp	4.87E-06	0.000776279	BP	8
Layer 2/3	iTBS	GO:0016072	rRNA metabolic process	5	Rpl35/Rps21/Rp	6.09E-06	0.000776279	BP	8
Layer 2/3	iTBS	GO:0099504	synaptic vesicle cycle	5	Actb/Dnm1/Cpl	6.63E-06	0.000776279	BP	1
Layer 2/3	iTBS	GO:0099003	vesicle-mediated transport in synapse	5	Actb/Dnm1/Cpl	1.14E-05	0.000970232	BP	1
Layer 2/3	iTBS	GO:0034470	ncRNA processing	5	Rpl35/Rps21/Rp	6.08E-05	0.003531833	BP	
Layer 2/3	iTBS	GO:0034660	ncRNA metabolic process	5	Rpl35/Rps21/Rp	0.000173986	0.006225221	BP	
Layer 2/3	iTBS	GO:0098978	glutamatergic synapse	5	Actb/Dnm1/Dy	3.94E-05	0.000240387	CC	1
Layer 2/3	iTBS	GO:0031625	ubiquitin protein ligase binding	5	Uba52/Rpl23/T	3.35E-05	0.001097492	MF	
Layer 2/3	iTBS	GO:0044389	ubiquitin-like protein ligase binding	5	Uba52/Rpl23/T	4.31E-05	0.001097492	MF	
Layer 2/3	iTBS	GO:0006836	neurotransmitter transport	4	Cplx2/Slc17a7/I	0.000185748	0.006225221	BP	2
Layer 2/3	iTBS	GO:0001505	regulation of neurotransmitter levels	4	Cplx2/Slc17a7/I	0.000226453	0.006267142	BP	2
Layer 2/3	iTBS	GO:0051348	negative regulation of transferase activity	4	Actb/Gstp1/Rpl	0.000233241	0.006267142	BP	
Layer 2/3	iTBS	GO:0008021	synaptic vesicle	4	Dnm1/Slc17a7/	0.000108553	0.000602494	CC	1
Layer 2/3	iTBS	GO:0070382	exocytic vesicle	4	Dnm1/Slc17a7/	0.000163341	0.000712314	CC	
Layer 2/3	iTBS	GO:0030133	transport vesicle	4	Dnm1/Slc17a7/	0.000556414	0.001887251	CC	
Layer 2/3	iTBS	GO:0014069	postsynaptic density	4	Actb/Syngap1/I	0.000647358	0.002080153	CC	1
Layer 2/3	iTBS	GO:0032279	asymmetric synapse	4	Actb/Syngap1/I	0.0007162	0.002186295	CC	1
Layer 2/3	iTBS	GO:0099572	postsynaptic specialization	4	Actb/Syngap1/I	0.000833386	0.002312746	CC	1
Layer 2/3	iTBS	GO:0098984	neuron to neuron synapse	4	Actb/Syngap1/I	0.000906122	0.002405267	CC	1
Layer 2/3	iTBS	GO:0150034	distal axon	4	Cdk5r2/Actb/Cp	0.001023477	0.002603583	CC	
Layer 2/3	iTBS	GO:0005874	microtubule	4	Dnm1/Tuba1a/	0.001715549	0.003879214	CC	
Layer 2/3	iTBS	GO:0003924	GTPase activity	4	Dnm1/Tuba1a/	0.000406476	0.003267366	MF	
Layer 2/3	iTBS	GO:0048168	regulation of neuronal synaptic plasticity	3	Syngap1/Syngr1	7.63E-05	0.003531833	BP	1
Layer 2/3	iTBS	GO:0048488	synaptic vesicle endocytosis	3	Actb/Dnm1/Slc	7.96E-05	0.003531833	BP	1
Layer 2/3	iTBS	GO:0140238	presynaptic endocytosis	3	Actb/Dnm1/Slc	7.96E-05	0.003531833	BP	1
Layer 2/3	iTBS	GO:0042274	ribosomal small subunit biogenesis	3	Rps21/Rps8/Rp	8.30E-05	0.003531833	BP	8
Layer 2/3	iTBS	GO:0036465	synaptic vesicle recycling	3	Actb/Dnm1/Slc	0.000122289	0.004802442	BP	1
Layer 2/3	iTBS	GO:0002181	cytoplasmic translation	3	Rpl9/Rps21/Rpl	0.0001951	0.006225221	BP	
Layer 2/3	iTBS	GO:0030672	synaptic vesicle membrane	3	Slc17a7/Syngr1	0.000130306	0.000611965	CC	1
Layer 2/3	iTBS	GO:0099501	exocytic vesicle membrane	3	Slc17a7/Syngr1	0.000130306	0.000611965	CC	
Layer 2/3	iTBS	GO:0030658	transport vesicle membrane	3	Slc17a7/Syngr1	0.000405226	0.001509937	CC	
Layer 2/3	iTBS	GO:0043679	axon terminus	3	Actb/Cplx2/Glu	0.001523325	0.003577039	CC	
Layer 2/3	iTBS	GO:0044306	neuron projection terminus	3	Actb/Cplx2/Glu	0.002088393	0.004553639	CC	
Layer 2/3	iTBS	GO:0005200	structural constituent of cytoskeleton	3	Actb/Tuba1a/T	6.19E-05	0.001097492	MF	
Layer 2/3	iTBS	GO:0019843	rRNA binding	3	Rpl9/Rpl23/Rpl	9.94E-05	0.001098673	MF	8
Layer 2/3	iTBS	GO:1904667	negative regulation of ubiquitin protein ligase activity	2	Rpl23/Rpl5	7.29E-05	0.003531833	BP	
Layer 2/3	iTBS	GO:0051444	negative regulation of ubiquitin-protein transferase activity	2	Rpl23/Rpl5	0.000225185	0.006267142	BP	
Layer 2/3	iTBS	GO:0044305	calyx of Held	2	Actb/Cplx2	0.000215569	0.000877405	CC	
Layer 2/3	iTBS	GO:0098563	intrinsic component of synaptic vesicle membrane	2	Slc17a7/Syn1	0.000420439	0.001509937	CC	1
Layer 2/3	iTBS	GO:0042788	polysomal ribosome	2	Rps21/Rpl30	0.000827554	0.002312746	CC	8
Layer 2/3	iTBS	GO:0030665	clathrin-coated vesicle membrane	2	Epn1/Syn1	0.001306772	0.003191275	CC	1
Layer 2/3	iTBS	GO:1990948	ubiquitin ligase inhibitor activity	2	Rpl23/Rpl5	6.21E-05	0.001097492	MF	
Layer 2/3	iTBS	GO:0055105	ubiquitin-protein transferase inhibitor activity	2	Rpl23/Rpl5	7.58E-05	0.001098673	MF	
Layer 2/3	iTBS	GO:0031386	protein tag	2	Uba52/Rps27a	9.09E-05	0.001098673	MF	
Layer 2/3	iTBS	GO:0050998	nitric-oxide synthase binding	2	Actb/Dnm1	0.000287237	0.002821977	MF	
Layer 2/3	iTBS	GO:0055106	ubiquitin-protein transferase regulator activity	2	Rpl23/Rpl5	0.000376663	0.003267366	MF	
Layer 5	iTBS	GO:0098793	presynapse	8	Cplx2/Dnm1/Ac	2.00E-08	1.60E-06	CC	1
Layer 5	iTBS	GO:0099504	synaptic vesicle cycle	6	Cplx2/Dnm1/Ac	9.01E-08	3.11E-05	BP	1
Layer 5	iTBS	GO:0099003	vesicle-mediated transport in synapse	6	Cplx2/Dnm1/Ac	1.74E-07	3.11E-05	BP	1
Layer 5	iTBS	GO:0043209	myelin sheath	6	Uba52/Tuba1b,	6.53E-08	2.57E-06	CC	4
Layer 5	iTBS	GO:0022626	cytosolic ribosome	5	Uba52/Rps8/Rp	9.65E-08	2.57E-06	CC	8
Layer 5	iTBS	GO:0044391	ribosomal subunit	5	Uba52/Rps8/Rp	1.46E-06	2.33E-05	CC	8
Layer 5	iTBS	GO:0005840	ribosome	5	Uba52/Rps8/Rp	3.75E-06	5.01E-05	CC	8
Layer 5	iTBS	GO:0098978	glutamatergic synapse	5	Cplx2/Dnm1/Dy	1.52E-05	0.000152066	CC	1
Layer 5	iTBS	GO:0003735	structural constituent of ribosome	5	Uba52/Rps8/Rp	6.76E-07	6.05E-05	MF	8
Layer 5	iTBS	GO:0036465	synaptic vesicle recycling	4	Dnm1/Actb/Stx	1.33E-06	0.000158747	BP	1
Layer 5	iTBS	GO:0006836	neurotransmitter transport	4	Cplx2/Stx1b/Slc	9.83E-05	0.004385529	BP	2
Layer 5	iTBS	GO:0001505	regulation of neurotransmitter levels	4	Cplx2/Stx1b/Slc	0.000120077	0.004414519	BP	2
Layer 5	iTBS	GO:0051348	negative regulation of transferase activity	4	Gstp1/Actb/Rpl	0.000123711	0.004414519	BP	
Layer 5	iTBS	GO:0016050	vesicle organization	4	Cplx2/Dnm1/St	0.000176152	0.005525982	BP	1
Layer 5	iTBS	GO:0045055	regulated exocytosis	4	Cplx2/Cdk5r2/S	0.000185829	0.005525982	BP	
Layer 5	iTBS	GO:0042254	ribosome biogenesis	4	Rps8/Rpl35/Rpl	0.00020633	0.005663629	BP	8
Layer 5	iTBS	GO:0022625	cytosolic large ribosomal subunit	4	Uba52/Rpl9/Rp	4.68E-07	9.37E-06	CC	8
Layer 5	iTBS	GO:0015934	large ribosomal subunit	4	Uba52/Rpl9/Rp	6.63E-06	7.58E-05	CC	8
Layer 5	iTBS	GO:0008021	synaptic vesicle	4	Dnm1/Stx1b/Sl	5.09E-05	0.000452517	CC	1
Layer 5	iTBS	GO:0070382	exocytic vesicle	4	Dnm1/Stx1b/Sl	7.69E-05	0.000615148	CC	
Layer 5	iTBS	GO:0030133	transport vesicle	4	Dnm1/Stx1b/Sl	0.000265712	0.001635149	CC	
Layer 5	iTBS	GO:0150034	distal axon	4	Cplx2/Cdk5r2/A	0.000493266	0.002630753	CC	
Layer 5	iTBS	GO:1903421	regulation of synaptic vesicle recycling	3	Dnm1/Stx1b/Sl	4.80E-06	0.000428491	BP	1
Layer 5	iTBS	GO:0048488	synaptic vesicle endocytosis	3	Dnm1/Actb/Slc	4.90E-05	0.002917031	BP	1
Layer 5	iTBS	GO:0140238	presynaptic endocytosis	3	Dnm1/Actb/Slc	4.90E-05	0.002917031	BP	1
Layer 5	iTBS	GO:0070373	negative regulation of ERK1 and ERK2 cascade	3	Gstp1/Dusp26/	7.82E-05	0.003987176	BP	
Layer 5	iTBS	GO:0030863	cortical cytoskeleton	3	Actb/Sptbn2/Pv	0.000247863	0.001635149	CC	
Layer 5	iTBS	GO:0042734	presynaptic membrane	3	Dnm1/Stx1b/Ep	0.000305531	0.00174589	CC	1
Layer 5	iTBS	GO:0043679	axon terminus	3	Cplx2/Actb/Glu	0.000875169	0.004375846	CC	
Layer 5	iTBS	GO:0044306	neuron projection terminus	3	Cplx2/Actb/Glu	0.001203869	0.005068921	CC	
Layer 5	iTBS	GO:0031629	synaptic vesicle fusion to presynaptic active zone membrane	2	Cplx2/Stx1b	0.000262734	0.006250311	BP	1
Layer 5	iTBS	GO:1900242	regulation of synaptic vesicle endocytosis	2	Dnm1/Slc17a7	0.000262734	0.006250311	BP	1
Layer 5	iTBS	GO:0044305	calyx of Held	2	Cplx2/Actb	0.00014742	0.001072149	CC	
Layer 5	iTBS	GO:0022627	cytosolic small ribosomal subunit	2	Uba52/Rps8	0.001022947	0.004741153	CC	8
Layer 5	iTBS	GO:0031201	SNARE complex	2	Cplx2/Stx1b	0.001066759	0.004741153	CC	1
Layer 6	iTBS	GO:0022626	cytosolic ribosome	4	Uba52/Rpl35/R	2.86E-08	3.91E-07	CC	8
Layer 6	iTBS	GO:0044391	ribosomal subunit	4	Uba52/Rpl35/R	2.57E-07	1.76E-06	CC	8
Layer 6	iTBS	GO:0005840	ribosome	4	Uba52/Rpl35/R	5.57E-07	2.54E-06	CC	8
Layer 6	iTBS	GO:0003735	structural constituent of ribosome	4	Uba52/Rpl35/R	7.70E-08	1.30E-06	MF	8
Layer 6	iTBS	GO:0022625	cytosolic large ribosomal subunit	3	Uba52/Rpl35/R	8.23E-07	2.81E-06	CC	8
Layer 6	iTBS	GO:0015934	large ribosomal subunit	3	Uba52/Rpl35/R	6.06E-06	1.66E-05	CC	8
Layer 6	iTBS	GO:0022627	cytosolic small ribosomal subunit	2	Uba52/Rps8	9.26E-05	0.000211207	CC	8
Layer 6	iTBS	GO:0015935	small ribosomal subunit	2	Uba52/Rps8	0.000269268	0.000526389	CC	8
Layer 6	iTBS	GO:0043209	myelin sheath	2	Uba52/Eef1a2	0.001917959	0.003280718	CC	4
Layer 2/3	cTBS	GO:0043209	myelin sheath	4	Uba52/Tuba1a,	5.06E-06	9.59E-05	CC	4
Layer 5	cTBS	GO:0022626	cytosolic ribosome	5	Rpl35/Uba52/R	2.55E-09	6.71E-08	CC	8
Layer 5	cTBS	GO:0044391	ribosomal subunit	5	Rpl35/Uba52/R	3.98E-08	5.24E-07	CC	8
Layer 5	cTBS	GO:0005840	ribosome	5	Rpl35/Uba52/R	1.04E-07	9.16E-07	CC	8
Layer 5	cTBS	GO:0003735	structural constituent of ribosome	5	Rpl35/Uba52/R	1.83E-08	6.35E-07	MF	8
Layer 5	cTBS	GO:0022627	cytosolic small ribosomal subunit	3	Uba52/Rps29/R	1.81E-06	1.19E-05	CC	8
Layer 5	cTBS	GO:0022625	cytosolic large ribosomal subunit	3	Rpl35/Uba52/R	4.42E-06	2.32E-05	CC	8
Layer 5	cTBS	GO:0015935	small ribosomal subunit	3	Uba52/Rps29/R	9.09E-06	3.99E-05	CC	8
Layer 5	cTBS	GO:0015934	large ribosomal subunit	3	Rpl35/Uba52/R	3.22E-05	0.000121151	CC	8
Layer 5	cTBS	GO:0043209	myelin sheath	3	Uba52/Atp1a3/	0.000173186	0.00056969	CC	4
Layer 5	cTBS	GO:1902554	serine/threonine protein kinase complex	2	Ikbkb/Cdk5r2	0.000856061	0.002503103	CC	
Layer 5	cTBS	GO:1902911	protein kinase complex	2	Ikbkb/Cdk5r2	0.001233831	0.003246923	CC	
Layer 6	cTBS	GO:0022625	cytosolic large ribosomal subunit	3	Rpl35/Rpl38/Ub	1.17E-06	9.88E-06	CC	8
Layer 6	cTBS	GO:0022626	cytosolic ribosome	3	Rpl35/Rpl38/Ub	7.04E-06	2.42E-05	CC	8
Layer 6	cTBS	GO:0015934	large ribosomal subunit	3	Rpl35/Rpl38/Ub	8.63E-06	2.42E-05	CC	8
Layer 6	cTBS	GO:0044391	ribosomal subunit	3	Rpl35/Rpl38/Ub	3.62E-05	7.62E-05	CC	8
Layer 6	cTBS	GO:0005840	ribosome	3	Rpl35/Rpl38/Ub	6.43E-05	0.000108297	CC	8
Layer 6	cTBS	GO:0003735	structural constituent of ribosome	3	Rpl35/Rpl38/Ub	2.27E-05	0.000239417	MF	8

Code key	High order function or process
1	synapse and/or synaptic plasticity
2	Neurotransmitter
3	Intrinsic plasticity and/or membrane excitability
4	Oligodendrocyte and/or myelin related
5	neurogenesis
6	neurotrophin
7	Cell death
8	Ribosome
9	ATP metabolic pathway

Data S6. Somatosensory (SS) cortex layer-specific differentially expressed genes (DEGs) identified in aged mice 3h following iTBS, and cTBS.

SS layer	Stimulation	Gene Symbol	Gene Name	EntrezID	Avg log2FoldChange	% expressed in stimulated samples	% expressed in sham samples	p.adjust value (Bonferroni correction)	p.value	Higher-order groups
Layer 2/3	iTBS	Gm10076	ribosomal protein L41 pseudogene	100126819	1.068215366	0.756	0.419	1.36E-25	9.20E-30	
Layer 2/3	iTBS	Uba52	ubiquitin A-52 residue ribosomal protein fusion product 1	22186	0.710738094	0.848	0.64	5.15E-16	3.49E-20	
Layer 2/3	iTBS	Gstp1	glutathione S-transferase, pi 1	14870	0.362570079	0.446	0.244	0.000247185	1.67E-08	
Layer 2/3	iTBS	Adcy1	adenylate cyclase 1	432530	0.320730341	0.825	0.726	0.001107199	7.49E-08	1
Layer 2/3	iTBS	Bsg	basigin	12215	0.287372453	0.977	0.931	0.000262858	1.78E-08	
Layer 2/3	iTBS	Rpl35	ribosomal protein L35	66489	0.278652367	0.974	0.947	0.002592361	1.75E-07	
Layer 2/3	iTBS	Psd	pleckstrin and Sec7 domain containing	73728	0.258455079	0.967	0.842	0.027841205	1.88E-06	1
Layer 2/3	iTBS	Nme7	NME/NM23 family member 7	171567	-0.401414215	0.195	0.363	0.030227141	2.05E-06	
Layer 2/3	iTBS	Malat1	metastasis associated lung adenocarcinoma transcript 1 (non-coding RNA)	72289	-0.420450995	0.508	0.627	0.033937586	2.30E-06	1
Layer 2/3	iTBS	Bc1	brain cytoplasmic RNA 1	100568459	-0.680503187	0.551	0.726	3.47E-06	2.35E-10	1
Layer 4	iTBS	Gm10076	ribosomal protein L41 pseudogene	100126819	1.192706911	0.806	0.393	2.58E-32	1.74E-36	
Layer 4	iTBS	Uba52	ubiquitin A-52 residue ribosomal protein fusion product 1	22186	0.794795851	0.909	0.721	2.43E-20	1.65E-24	
Layer 4	iTBS	Rpl35	ribosomal protein L35	66489	0.431538062	0.987	0.943	6.57E-11	4.45E-15	
Layer 4	iTBS	Gstp1	glutathione S-transferase, pi 1	14870	0.384704024	0.435	0.188	1.29E-05	8.70E-10	
Layer 4	iTBS	Atp1b1	ATPase, Na+/K+ transporting, beta 1 polypeptide	11931	0.277359798	1	1	1.43E-05	9.66E-10	
Layer 4	iTBS	Camk2n1	calcium/calmodulin-dependent protein kinase II inhibitor 1	66259	0.265511261	1	1	1.96E-06	1.32E-10	1
Layer 4	iTBS	Snhg9	small nucleolar RNA host gene 9	73474	-0.264506064	0.098	0.271	0.000302944	2.05E-08	7
Layer 4	iTBS	Ndufa4	Ndufa4, mitochondrial complex associated	17992	-0.266486561	1	1	0.003414701	2.31E-07	
Layer 4	iTBS	Tuba1b	tubulin, alpha 1B	22143	-0.297419376	0.938	0.987	0.000397833	2.69E-08	
Layer 4	iTBS	Bc1	brain cytoplasmic RNA 1	100568459	-0.418345608	0.355	0.563	0.001108452	7.50E-08	1
Layer 4	iTBS	Malat1	metastasis associated lung adenocarcinoma transcript 1 (non-coding RNA)	72289	-0.575145043	0.466	0.681	1.05E-08	7.12E-13	1
Layer 5	iTBS	Gm10076	ribosomal protein L41 pseudogene	100126819	0.965664168	0.747	0.41	2.39E-25	1.62E-29	
Layer 5	iTBS	Uba52	ubiquitin A-52 residue ribosomal protein fusion product 1	22186	0.52930806	0.869	0.753	4.14E-09	2.80E-13	
Layer 5	iTBS	Rpl35	ribosomal protein L35	66489	0.274108668	0.991	0.96	0.000102629	6.94E-09	
Layer 5	iTBS	Cdk5r2	cyclin-dependent kinase 5, regulatory subunit 2 (p39)	12570	-0.279423366	0.294	0.481	0.002802952	1.90E-07	
Layer 5	iTBS	Bc1	brain cytoplasmic RNA 1	100568459	-0.436738846	0.462	0.598	0.025236626	1.71E-06	1
Layer 6	iTBS	Gm10076	ribosomal protein L41 pseudogene	100126819	0.966468934	0.783	0.563	7.94E-22	5.37E-26	
Layer 6	iTBS	Uba52	ubiquitin A-52 residue ribosomal protein fusion product 1	22186	0.690898808	0.911	0.837	7.11E-17	4.81E-21	
Layer 6	iTBS	Gstp1	glutathione S-transferase, pi 1	14870	0.53023204	0.513	0.28	1.95E-08	1.32E-12	
Layer 6	iTBS	Rpl35	ribosomal protein L35	66489	0.36946784	0.995	0.979	3.96E-10	2.68E-14	
Layer 6	iTBS	Rps20	ribosomal protein S20	67427	0.281960799	1	0.997	7.63E-08	5.16E-12	
Layer 6	iTBS	Eno1	enolase 1, alpha non-neuron	13806	0.280086245	0.963	0.939	0.002393972	1.62E-07	
Layer 6	iTBS	Chd9	chromodomain helicase DNA binding protein 9	109151	0.268061223	0.325	0.144	9.88E-05	6.69E-09	
Layer 6	iTBS	Rps10	ribosomal protein S10	67097	0.250814978	0.997	0.979	0.003173887	2.15E-07	
Layer 6	iTBS	Tuba1b	tubulin, alpha 1B	22143	-0.283839882	0.945	0.989	0.000186732	1.26E-08	
Layer 6	iTBS	Bc1	brain cytoplasmic RNA 1	100568459	-0.515050145	0.429	0.667	1.14E-08	7.74E-13	1
Layer 2/3	cTBS	Gm10076	ribosomal protein L41 pseudogene	100126819	0.825540773	0.704	0.419	4.18E-15	2.83E-19	
Layer 2/3	cTBS	Uba52	ubiquitin A-52 residue ribosomal protein fusion product 1	22186	0.49596171	0.737	0.64	0.001616433	1.09E-07	
Layer 2/3	cTBS	Rpl35	ribosomal protein L35	66489	0.3468535	0.98	0.947	0.001526864	1.03E-07	
Layer 2/3	cTBS	Psd	pleckstrin and Sec7 domain containing	73728	0.288558604	0.955	0.842	0.000261706	1.77E-08	1
Layer 2/3	cTBS	Ttr	transthyretin	22139	-0.252681043	0	0.122	0.000202003	1.37E-08	1
Layer 2/3	cTBS	Ikbkb	inhibitor of kappaB kinase beta	16150	-0.308652092	0.235	0.416	0.021137299	1.43E-06	7
Layer 2/3	cTBS	Bc1	brain cytoplasmic RNA 1	100568459	-0.934349372	0.348	0.726	2.36E-15	1.59E-19	1
Layer 4	cTBS	Gm10076	ribosomal protein L41 pseudogene	100126819	0.727480247	0.666	0.393	9.26E-12	6.27E-16	
Layer 4	cTBS	Rpl35	ribosomal protein L35	66489	0.415216089	0.97	0.943	1.54E-06	1.04E-10	
Layer 4	cTBS	Camk2n1	calcium/calmodulin-dependent protein kinase II inhibitor 1	66259	0.29066586	1	1	5.87E-10	3.97E-14	1
Layer 4	cTBS	Camk2n2	calcium/calmodulin-dependent protein kinase II inhibitor 2	73047	0.275621869	0.908	0.843	0.015427158	1.04E-06	1
Layer 4	cTBS	Ncdn	neurochondrin	26562	0.263969948	1	0.978	0.003990921	2.70E-07	1
Layer 4	cTBS	Ttr	transthyretin	22139	-0.294428991	0.015	0.162	7.06E-07	4.78E-11	1
Layer 4	cTBS	Bc1	brain cytoplasmic RNA 1	100568459	-0.553197494	0.266	0.563	2.10E-08	1.42E-12	1
Layer 4	cTBS	Pcp4	Purkinje cell protein 4	18546	-0.556809893	0.604	0.755	0.001595321	1.08E-07	1
Layer 5	cTBS	Gm10076	ribosomal protein L41 pseudogene	100126819	0.762610332	0.685	0.41	2.01E-17	1.36E-21	
Layer 5	cTBS	Uba52	ubiquitin A-52 residue ribosomal protein fusion product 1	22186	0.399234149	0.787	0.753	0.044643899	3.02E-06	
Layer 5	cTBS	Rpl35	ribosomal protein L35	66489	0.392826794	0.977	0.96	1.30E-09	8.77E-14	
Layer 5	cTBS	Bc1	brain cytoplasmic RNA 1	100568459	-0.553883679	0.364	0.598	1.01E-07	6.82E-12	1
Layer 6	cTBS	Gm10076	ribosomal protein L41 pseudogene	100126819	0.648563877	0.738	0.563	2.05E-12	1.39E-16	
Layer 6	cTBS	Gstp1	glutathione S-transferase, pi 1	14870	0.463396118	0.498	0.28	6.39E-06	4.32E-10	
Layer 6	cTBS	Eno1	enolase 1, alpha non-neuron	13806	0.297044684	0.966	0.939	0.00125099	8.47E-08	
Layer 6	cTBS	Rpl35	ribosomal protein L35	66489	0.287112276	0.972	0.979	0.012213146	8.26E-07	
Layer 6	cTBS	Bc1	brain cytoplasmic RNA 1	100568459	-0.643015917	0.357	0.667	2.24E-14	1.51E-18	1

Data S7. Complete list of enriched Gene Ontology (GO) terms for the significant differentially expressed genes (DEGs), identified in the different somatosensory (SS) cortex layers, following iTBS, and cTBS.

SS layer	Stimulation	GO ID	GO term description	Number of genes	Gene symbols	p.value	q.value	Ontology Source	Code
Layer 4	iTBS	GO:0022625	cytosolic large ribosomal subunit	2	Uba52/Rpl35	4.66E-05	4.91E-05	CC	8
Layer 4	iTBS	GO:0022626	cytosolic ribosome	2	Uba52/Rpl35	0.000153198	6.16E-05	CC	8
Layer 4	iTBS	GO:0015934	large ribosomal subunit	2	Uba52/Rpl35	0.000175458	6.16E-05	CC	8
Layer 4	iTBS	GO:0044391	ribosomal subunit	2	Uba52/Rpl35	0.00045655	0.000120145	CC	8
Layer 4	iTBS	GO:0005840	ribosome	2	Uba52/Rpl35	0.000670615	0.000141182	CC	8
Layer 4	iTBS	GO:0003735	structural constituent of ribosome	2	Uba52/Rpl35	0.000334805	0.001409707	MF	8
Layer 5	iTBS	GO:0043209	myelin sheath	3	Uba52/Atp1b1/Tuba1b	6.42E-05	0.001284023	CC	4
Layer 5	iTBS	GO:0022625	cytosolic large ribosomal subunit	2	Uba52/Rpl35	0.000254233	0.002542327	CC	8
Layer 6	iTBS	GO:0022626	cytosolic ribosome	4	Uba52/Rpl35/Rps20/Rps10	4.74E-08	3.50E-07	CC	8
Layer 6	iTBS	GO:0044391	ribosomal subunit	4	Uba52/Rpl35/Rps20/Rps10	4.27E-07	1.18E-06	CC	8
Layer 6	iTBS	GO:0005840	ribosome	4	Uba52/Rpl35/Rps20/Rps10	9.23E-07	1.70E-06	CC	8
Layer 6	iTBS	GO:0003735	structural constituent of ribosome	4	Uba52/Rpl35/Rps20/Rps10	2.29E-07	3.61E-06	MF	8
Layer 6	iTBS	GO:0022627	cytosolic small ribosomal subunit	3	Uba52/Rps20/Rps10	4.81E-07	1.18E-06	CC	8
Layer 6	iTBS	GO:0015935	small ribosomal subunit	3	Uba52/Rps20/Rps10	2.42E-06	3.57E-06	CC	8
Layer 6	iTBS	GO:0043209	myelin sheath	3	Uba52/Tuba1b/Eno1	4.69E-05	5.77E-05	CC	4
Layer 6	iTBS	GO:0022625	cytosolic large ribosomal subunit	2	Uba52/Rpl35	0.000208302	0.000219265	CC	8
Layer 6	iTBS	GO:0015934	large ribosomal subunit	2	Uba52/Rpl35	0.000778704	0.000717228	CC	8
Layer 2/3	cTBS	GO:0022625	cytosolic large ribosomal subunit	2	Rpl35/Uba52	9.76e-05	0.001130322	CC	8
Layer 2/3	cTBS	GO:0022626	cytosolic ribosome	2	Rpl35/Uba52	0.000320056	0.001414275	CC	8
Layer 2/3	cTBS	GO:0015934	large ribosomal subunit	2	Rpl35/Uba52	0.000366426	0.001414275	CC	8
Layer 2/3	cTBS	GO:0044391	ribosomal subunit	2	Rpl35/Uba52	0.000950161	0.002750465	CC	8
Layer 2/3	cTBS	GO:0005840	ribosome	2	Rpl35/Uba52	0.001392962	0.003225807	CC	8
Layer 4	cTBS	GO:0006469	negative regulation of protein kinase activity	3	Camk2n1/Pcp4/Camk2n2	1.84E-05	0.000775076	BP	9
Layer 4	cTBS	GO:0033673	negative regulation of kinase activity	3	Camk2n1/Pcp4/Camk2n2	2.54E-05	0.000775076	BP	9
Layer 4	cTBS	GO:0051348	negative regulation of transferase activity	3	Camk2n1/Pcp4/Camk2n2	3.81E-05	0.000775721	BP	9
Layer 4	cTBS	GO:0001933	negative regulation of protein phosphorylation	3	Camk2n1/Pcp4/Camk2n2	9.25E-05	0.001411126	BP	9
Layer 4	cTBS	GO:0042326	negative regulation of phosphorylation	3	Camk2n1/Pcp4/Camk2n2	0.000134375	0.001640788	BP	9
Layer 4	cTBS	GO:0010563	negative regulation of phosphorus metabolic process	3	Camk2n1/Pcp4/Camk2n2	0.000199856	0.001743102	BP	9
Layer 4	cTBS	GO:0045936	negative regulation of phosphate metabolic process	3	Camk2n1/Pcp4/Camk2n2	0.000199856	0.001743102	BP	9
Layer 4	cTBS	GO:0004860	protein kinase inhibitor activity	2	Camk2n1/Camk2n2	0.000129846	0.000239546	MF	9
Layer 4	cTBS	GO:0019210	kinase inhibitor activity	2	Camk2n1/Camk2n2	0.000151713	0.000239546	MF	9
Layer 4	cTBS	GO:0019887	protein kinase regulator activity	2	Camk2n1/Camk2n2	0.00130069	0.001369148	MF	9
Layer 4	cTBS	GO:0019207	kinase regulator activity	2	Camk2n1/Camk2n2	0.001750908	0.001382296	MF	9
Layer 5	cTBS	GO:0003735	structural constituent of ribosome	2	Rpl35/Uba52	0.000201658	0.000212271	MF	8
Layer 5	cTBS	GO:0031386	protein tag	1	Uba52	0.001686903	0.000887844	MF	

Code key	High order function or process
1	synapse and/or synaptic plasticity
2	Neurotransmitter
3	Intrinsic plasticity and/or membrane excitability
4	Oligodendrocyte and/or myelin related
5	neurogenesis
6	neurotrophin
7	Cell death
8	Ribosome
9	ATP metabolic pathway

Data S8. Differentially expressed genes (DEGs) identified in different cortical layers and brain regions in aged mice 3 h following intermittent (iTBS) or continuous theta burst stimulation (cTBS). Code for higher-order groups are as follows: 1 - synapse and/or synaptic plasticity; 2 - neurotransmitter; 3 - intrinsic plasticity and/or membrane excitability; 4 - oligodendrocyte and/or myelin related; 5 - neurogenesis; 6 - neurotrophin; 7 - inflammation/apoptosis.

Brain reigon	Stimulation	Gene Symbol	Gene Name	EntrezID	Avg log2FoldChange	% expressed in stimulated samples	% expressed in sham samples	p.adjust value (Bonferroni correction)	p.value	Higher-order groups
Cortical Layer L2/3	iTBS	Nr4a1	nuclear receptor subfamily 4, group A, member 1	15370	0.334222124	0.553	0.42	0.000331119	2.2E-08	1
Cortical Layer L2/3	iTBS	Adcy1	adenylate cyclase 1	432530	0.281144358	0.858	0.779	2.09E-05	1.4E-09	1
Cortical Layer L2/3	iTBS	Glul	glutamate-ammonia ligase (glutamine synthetase)	14645	-0.277222094	0.76	0.862	0.001298862	8.8E-08	1
Cortical Layer L2/3	iTBS	Cplx2	complexin 2	12890	-0.311164116	0.856	0.853	0.010851651	7.3E-07	1
Cortical Layer L2/3	iTBS	Cdk5r2	cyclin-dependent kinase 5, regulatory subunit 2 (p39)	12570	-0.373759613	0.402	0.611	1.68E-10	1.1E-14	1
Cortical Layer L2/3	iTBS	Bc1	brain cytoplasmic RNA 1	100568459	-0.71989169	0.492	0.73	2.02E-18	1.4E-22	1
Cortical Layer 4	iTBS	Cdk5r2	cyclin-dependent kinase 5, regulatory subunit 2 (p39)	12570	-0.261825698	0.372	0.517	0.033278761	2.3E-06	1
Cortical Layer 4	iTBS	Bc1	brain cytoplasmic RNA 1	100568459	-0.387897219	0.437	0.608	0.00019765	1.3E-08	1
Cortical Layer 5	iTBS	Pvalb	parvalbumin	19293	0.291796575	0.896	0.818	2.56E-05	1.7E-09	1
Cortical Layer 5	iTBS	Syngr1	synaptogyrin 1	20972	-0.275389222	0.841	0.883	1.19E-05	8.0E-10	1
Cortical Layer 5	iTBS	Glul	glutamate-ammonia ligase (glutamine synthetase)	14645	-0.28424235	0.804	0.866	1.02E-08	6.9E-13	1
Cortical Layer 5	iTBS	Cdk5r2	cyclin-dependent kinase 5, regulatory subunit 2 (p39)	12570	-0.309355067	0.37	0.553	2.55E-13	1.7E-17	1
Cortical Layer 5	iTBS	Cplx2	complexin 2	12890	-0.353548571	0.705	0.797	1.65E-10	1.1E-14	1
Cortical Layer 5	iTBS	Bc1	brain cytoplasmic RNA 1	100568459	-0.583355218	0.437	0.665	2.18E-24	1.5E-28	1
Cortical Layer 6	iTBS	Ttr	transthyretin	22139	0.438710277	0.282	0.13	1.03E-06	6.9E-11	1
Cortical Layer 6	iTBS	Cplx2	complexin 2	12890	-0.263766222	0.851	0.899	0.002044561	1.4E-07	1
Cortical Layer 6	iTBS	Dnm1	dynamin 1	13429	-0.266230751	0.985	1	4.01E-05	2.7E-09	1
Cortical Layer 6	iTBS	Slc17a7	solute carrier family 17 (sodium-dependent inorganic phosphate cotransporter), member 7	72961	-0.268674902	0.995	0.994	0.000105398	7.1E-09	1
Cortical Layer 6	iTBS	Bc1	brain cytoplasmic RNA 1	100568459	-0.434433444	0.491	0.685	2.12E-09	1.4E-13	1
Piriform Cortex	iTBS	Ttr	transthyretin	22139	0.590664197	0.285	0.114	1.74E-08	1.2E-12	1
Piriform Cortex	iTBS	Lynx1	Ly6/neurotoxin 1	23936	-0.268718044	0.291	0.413	0.038741175	2.6E-06	1
Piriform Cortex	iTBS	Shank1	SH3 and multiple ankyrin repeat domains 1	243961	-0.269765778	0.386	0.539	0.001328322	9.0E-08	1
Piriform Cortex	iTBS	Eef2	eukaryotic translation elongation factor 2	13629	-0.324199801	0.937	0.971	1.37E-06	9.3E-11	1
Piriform Cortex	iTBS	Cplx2	complexin 2	12890	-0.361252302	0.816	0.844	0.000329245	2.2E-08	1
Piriform Cortex	iTBS	Cdk5r2	cyclin-dependent kinase 5, regulatory subunit 2 (p39)	12570	-0.385890004	0.338	0.554	1.01E-09	6.8E-14	1
Piriform Cortex	iTBS	Syngr1	synaptogyrin 1	20972	-0.409655719	0.774	0.838	8.95E-06	6.1E-10	1
Piriform Cortex	iTBS	Dnm1	dynamin 1	13429	-0.414065133	0.996	0.996	3.43E-14	2.3E-18	1
Piriform Cortex	iTBS	Syn1	synapsin I	20964	-0.422075828	0.933	0.947	4.84E-10	3.3E-14	1
Piriform Cortex	iTBS	Bc1	brain cytoplasmic RNA 1	100568459	-0.431876265	0.629	0.773	0.000814112	5.5E-08	1
Piriform Cortex	iTBS	Slc17a7	solute carrier family 17 (sodium-dependent inorganic phosphate cotransporter), member 7	72961	-0.445626054	0.996	0.998	2.56E-11	1.7E-15	1
White Matter Tracts	iTBS	Ttr	transthyretin	22139	0.609096865	0.3	0.078	3.26E-17	2.2E-21	1
White Matter Tracts	iTBS	Bc1	brain cytoplasmic RNA 1	100568459	-0.433049208	0.816	0.885	0.017471008	1.2E-06	1
Caudoputamen	iTBS	Ttr	transthyretin	22139	0.615804318	0.258	0.141	4.31E-10	2.9E-14	1
Caudoputamen	iTBS	Glul	glutamate-ammonia ligase (glutamine synthetase)	14645	-0.266010832	0.738	0.835	5.56E-09	3.8E-13	1
Caudoputamen	iTBS	Bc1	brain cytoplasmic RNA 1	100568459	-0.284200548	0.537	0.693	1.03E-08	7.0E-13	1
Caudoputamen	iTBS	Dnm1	dynamin 1	13429	-0.303727691	0.849	0.944	1.04E-13	7.1E-18	1
Lateral Septal Complex	iTBS	Kalrr	kalirin, RhoGEF kinase	545156	-0.252111211	0.27	0.449	0.016802031	1.1E-06	1
Lateral Septal Complex	iTBS	Cdk5r2	cyclin-dependent kinase 5, regulatory subunit 2 (p39)	12570	-0.29895976	0.232	0.441	5.13E-05	3.5E-09	1
Lateral Septal Complex	iTBS	Ttr	transthyretin	22139	-1.617776097	0.174	0.321	0.023932756	1.6E-06	1
Striatum Ventral Region	iTBS	Bc1	brain cytoplasmic RNA 1	100568459	-0.384592556	0.676	0.83	6.83E-07	4.9E-52	1
Cortical Layer L2/3	cTBS	Bc1	brain cytoplasmic RNA 1	100568459	-0.888235077	0.367	0.73	1.87E-28	1.3E-32	1
Cortical Layer L2/3	cTBS	Ttr	transthyretin	22139	-1.38847059	0.007	0.216	5.67E-18	3.8E-22	1
Cortical Layer 4	cTBS	Camk2n1	calcium/calmodulin-dependent protein kinase II inhibitor 1	66259	0.256554719	1	1	8.47E-12	5.7E-16	1
Cortical Layer 4	cTBS	Ttr	transthyretin	22139	-0.288348911	0.01	0.156	2.02E-12	1.4E-16	1
Cortical Layer 4	cTBS	Pcp4	Purkinje cell protein 4	18546	-0.399225391	0.681	0.789	0.007257433	4.9E-07	1
Cortical Layer 4	cTBS	Bc1	brain cytoplasmic RNA 1	100568459	-0.552946373	0.323	0.608	6.99E-13	4.7E-17	1
Cortical Layer 5	cTBS	Ttr	transthyretin	22139	-0.435559088	0.015	0.178	3.20E-26	2.2E-30	1
Cortical Layer 5	cTBS	Bc1	brain cytoplasmic RNA 1	100568459	-0.701902569	0.326	0.665	1.52E-43	1.0E-47	1
Cortical Layer 6	cTBS	Ttr	transthyretin	22139	-0.282802181	0.011	0.13	1.77E-10	1.2E-14	1
Cortical Layer 6	cTBS	Cdk5r2	cyclin-dependent kinase 5, regulatory subunit 2 (p39)	12570	-0.287295226	0.29	0.4	0.041982987	2.8E-06	1
Cortical Layer 6	cTBS	Bc1	brain cytoplasmic RNA 1	100568459	-0.645763393	0.366	0.685	1.07E-24	7.2E-29	1
Piriform Cortex	cTBS	Syn1	synapsin I	20964	-0.2807775	0.946	0.947	0.017678469	1.2E-06	1
Piriform Cortex	cTBS	Bc1	brain cytoplasmic RNA 1	100568459	-0.967071131	0.388	0.773	6.07E-32	4.1E-36	1
White Matter Tracts	cTBS	Bc1	brain cytoplasmic RNA 1	100568459	-0.834899137	0.713	0.885	1.48E-22	1.0E-26	1
Caudoputamen	cTBS	Slc17a7	solute carrier family 17 (sodium-dependent inorganic phosphate cotransporter), member 7	72961	-0.272338813	0.196	0.361	1.47E-14	9.9E-19	1
Caudoputamen	cTBS	Ttr	transthyretin	22139	-0.326153983	0.002	0.141	4.66E-33	3.2E-37	1
Caudoputamen	cTBS	Bc1	brain cytoplasmic RNA 1	100568459	-0.704317676	0.342	0.693	2.11E-61	1.4E-65	1
Lateral Septal Complex	cTBS	Cnih2	cornichon family AMPA receptor auxiliary protein 2	12794	-0.289620486	0.92	0.961	0.010041113	6.8E-07	1
Lateral Septal Complex	cTBS	Gria1	glutamate receptor, ionotropic, AMPA1 (alpha 1)	14799	-0.334501905	0.691	0.815	0.003208812	2.2E-07	1
Lateral Septal Complex	cTBS	Bc1	brain cytoplasmic RNA 1	100568459	-1.114948558	0.585	0.909	2.59E-32	1.8E-36	1
Lateral Septal Complex	cTBS	Ttr	transthyretin	22139	-1.951188266	0.101	0.321	6.79E-10	4.6E-14	1
Striatum Ventral Region	cTBS	Malat1	metastasis associated lung adenocarcinoma transcript 1 (non-coding RNA)	72289	-0.307512671	0.573	0.643	0.017126764	1.2E-06	1
Striatum Ventral Region	cTBS	Bc1	brain cytoplasmic RNA 1	100568459	-0.926359105	0.48	0.83	8.05E-52	5.4E-56	1
Lateral Septal Complex	iTBS	Kcnq2	potassium voltage-gated channel, subfamily Q, member 2	16536	-0.276817724	0.46	0.621	0.044984929	3.0E-06	3
Striatum Ventral Region	iTBS	Atp1a3	ATPase, Na+/K+ transporting, alpha 3 polypeptide	232975	-0.266323597	0.735	0.818	6.50E-06	4.9E-52	3
Piriform Cortex	cTBS	Atp1a3	ATPase, Na+/K+ transporting, alpha 3 polypeptide	232975	-0.323809229	0.753	0.863	3.61E-06	2.4E-10	3
Piriform Cortex	iTBS	Nat8l	N-acetyltransferase 8-like	269642	-0.261190961	0.583	0.684	0.036544555	2.5E-06	4
White Matter Tracts	cTBS	Bcas1	brain enriched myelin associated protein 1	76960	0.348352484	0.695	0.545	0.000721123	4.9E-08	4
White Matter Tracts	cTBS	Mbp	myelin basic protein	17196	0.338416104	1	1	3.39E-09	2.3E-13	4
White Matter Tracts	cTBS	Mobp	myelin-associated oligodendrocytic basic protein	17433	0.317797139	0.998	1	7.71E-06	5.2E-10	4
Piriform Cortex	cTBS	Ptgds	prostaglandin D2 synthase (brain)	19215	-0.279582549	0.642	0.808	0.006737367	4.6E-07	6
Striatum Ventral Region	cTBS	Ptgds	prostaglandin D2 synthase (brain)	19215	-0.353222369	0.694	0.79	0.024112355	1.6E-06	6
Cortical Layer L2/3	iTBS	Ndufa4	Ndufa4, mitochondrial complex associated	17992	-0.267810222	1	1	7.10E-07	4.8E-11	7
Cortical Layer 5	iTBS	Tuba1b	tubulin, alpha 1B	22143	-0.301187772	0.967	0.985	1.71E-18	1.2E-22	7
Piriform Cortex	iTBS	Ikbkb	inhibitor of kappaB kinase beta	16150	-0.307852202	0.245	0.432	1.03E-06	6.9E-11	7
White Matter Tracts	iTBS	Snhg8	small nucleolar RNA host gene 8	69895	0.308124984	0.5	0.237	6.81E-12	4.6E-16	7
White Matter Tracts	iTBS	Banf1	BAF nuclear assembly factor 1	23825	0.252885736	0.493	0.295	1.16E-05	7.8E-10	7
Cortical Layer L2/3	cTBS	Ikbkb	inhibitor of kappaB kinase beta	16150	-0.27364604	0.258	0.452	5.71E-06	3.9E-10	7
Piriform Cortex	cTBS	Ikbkb	inhibitor of kappaB kinase beta	16150	-0.260378449	0.275	0.432	0.001712617	1.2E-07	7
Cortical Layer L2/3	iTBS	Gm10076	ribosomal protein L41 pseudogene	100126819	1.230542179	0.776	0.348	7.79E-63	5.3E-67	
Cortical Layer L2/3	iTBS	Uba52	ubiquitin A-52 residue ribosomal protein fusion product 1	22186	0.823819101	0.841	0.62	1.14E-38	7.7E-43	
Cortical Layer L2/3	iTBS	Gstp1	glutathione S-transferase, pi 1	14870	0.398236375	0.467	0.236	7.66E-13	5.2E-17	
Cortical Layer L2/3	iTBS	Rpl35	ribosomal protein L35	66489	0.369907549	0.974	0.9	1.68E-13	1.1E-17	
Cortical Layer L2/3	iTBS	Phyhip	phytanoyl-CoA hydroxylase interacting protein	105653	-0.271420143	0.537	0.66	0.000878778	5.9E-08	
Cortical Layer L2/3	iTBS	Ivns1abp	influenza virus NS1A binding protein	117198	-0.300612084	0.451	0.558	0.017918876	1.2E-06	
Cortical Layer L2/3	iTBS	Nme7	NME/NM23 family member 7	171567	-0.539648195	0.24	0.416	5.40E-07	3.7E-11	
Cortical Layer 4	iTBS	Gm10076	ribosomal protein L41 pseudogene	100126819	1.093702705	0.821	0.406	3.67E-45	2.5E-49	
Cortical Layer 4	iTBS	Uba52	ubiquitin A-52 residue ribosomal protein fusion product 1	22186	0.70093592	0.911	0.731	2.79E-23	1.9E-27	
Cortical Layer 4	iTBS	Rpl35	ribosomal protein L35	66489	0.359278949	0.982	0.953	2.70E-11	1.8E-15	
Cortical Layer 4	iTBS	Gstp1	glutathione S-transferase, pi 1	14870	0.346288988	0.419	0.203	4.67E-07	3.2E-11	
Cortical Layer 4	iTBS	Atp1b1	ATPase, Na+/K+ transporting, beta 1 polypeptide	11931	0.274732665	1	1	1.13E-08	7.6E-13	
Cortical Layer 4	iTBS	Lars2	leucyl-tRNA synthetase, mitochondrial	102436	-0.285776713	0.256	0.394	0.024616403	1.7E-06	
Cortical Layer 4	iTBS	Nme7	NME/NM23 family member 7	171567	-0.467957477	0.276	0.436	0.000403714	2.7E-08	
Cortical Layer 4	iTBS	Malat1	metastasis associated lung adenocarcinoma transcript 1 (non-coding RNA)	72289	-0.514632877	0.494	0.65	1.24E-08	8.4E-13	
Cortical Layer 5	iTBS	Gm10076	ribosomal protein L41 pseudogene	100126819	1.229509494	0.758	0.341	4.02E-100	3E-104	
Cortical Layer 5	iTBS	Uba52	ubiquitin A-52 residue ribosomal protein fusion product 1	22186	0.738317653	0.865	0.685	1.40E-52	9.5E-57	
Cortical Layer 5	iTBS	Rpl35	ribosomal protein L35	66489	0.370962746	0.987	0.918	6.52E-24	4.4E-28	
Cortical Layer 5	iTBS	Gstp1	glutathione S-transferase, pi 1	14870	0.346645918	0.483	0.313	2.03E-12	1.4E-16	
Cortical Layer 5	iTBS	Chd9	chromodomain helicase DNA binding protein 9	109151	0.25308307	0.385	0.207	4.57E-12	3.1E-16	
Cortical Layer 5	iTBS	Dynll2	dynein light chain LC8-type 2	68097	-0.252237304	0.967	0.975	1.01E-05	6.8E-10	
Cortical Layer 5	iTBS	Hpcal4	hippocalcin-like 4	170638	-0.267315518	0.582	0.703	1.98E-06	1.3E-10	
Cortical Layer 5	iTBS	Nme7	NME/NM23 family member 7	171567	-0.40447592	0.313	0.408	0.021411937	1.4E-06	
Cortical Layer 6	iTBS	Gm10076	ribosomal protein L41 pseudogene	100126819	0.97998495	0.782	0.559	1.13E-37	7.6E-42	
Cortical Layer 6	iTBS	Uba52	ubiquitin A-52 residue ribosomal protein fusion product 1	22186	0.661968159	0.91	0.832	2.28E-27	1.5E-31	
Cortical Layer 6	iTBS	Gstp1	glutathione S-transferase, pi 1	14870	0.518666373	0.53	0.285	4.13E-15	2.8E-19	
Cortical Layer 6	iTBS	Rpl35	ribosomal protein L35	66489	0.315851569	0.992	0.985	2.36E-12	1.6E-16	
Cortical Layer 6	iTBS	Chd9	chromodomain helicase DNA binding protein 9	109151	0.250144205	0.298	0.138	1.55E-06	1.0E-10	
Cortical Layer 6	iTBS	Map2	microtubule-associated protein 2	17756	-0.255229472	0.741	0.831	0.00060652	4.1E-08	
Cortical Layer 6	iTBS	Eef1a2	eukaryotic translation elongation factor 1 alpha 2	13628	-0.271544865	0.989	0.998	1.47E-05	9.9E-10	
Cortical Layer 6	iTBS	Hpcal4	hippocalcin-like 4	170638	-0.272302736	0.768	0.842	0.007162921	4.8E-07	
Piriform Cortex	iTBS	Gm10076	ribosomal protein L41 pseudogene	100126819	1.377621314	0.751	0.423	1.48E-49	1.0E-53	
Piriform Cortex	iTBS	Uba52	ubiquitin A-52 residue ribosomal protein fusion product 1	22186	0.844989401	0.878	0.701	2.69E-35	1.8E-39	
Piriform Cortex	iTBS	Rpl35	ribosomal protein L35	66489	0.404752236	0.985	0.958	1.61E-17	1.1E-21	
Piriform Cortex	iTBS	Rps28	ribosomal protein S28	54127	0.399194726	0.908	0.857	5.16E-08	3.5E-12	
Piriform Cortex	iTBS	Gstp1	glutathione S-transferase, pi 1	14870	0.354232782	0.528	0.354	2.96E-06	2.0E-10	
Piriform Cortex	iTBS	Rps27	ribosomal protein S27	57294	0.312121998	0.948	0.905	1.68E-06	1.1E-10	
Piriform Cortex	iTBS	Rps21	ribosomal protein S21	66481	0.309049681	1	1	7.26E-17	4.9E-21	
Piriform Cortex	iTBS	Rps29	ribosomal protein S29	20090	0.306562748	0.994	0.985	4.30E-05	2.9E-09	
Piriform Cortex	iTBS	Cox7c	cytochrome c oxidase subunit 7C	12867	0.298461796	1	0.998	5.62E-14	3.8E-18	
Piriform Cortex	iTBS	Rpl35a	ribosomal protein L35A	57808	0.285960325	1	0.985	1.08E-09	7.3E-14	
Piriform Cortex	iTBS	Rps26	ribosomal protein S26	27370	0.269601214	0.977	0.954	2.84E-06	1.9E-10	
Piriform Cortex	iTBS	Rpl9	ribosomal protein L9	20005	0.259279456	1	0.987	2.92E-10	2.0E-14	
Piriform Cortex	iTBS	Rps27a	ribosomal protein S27A	78294	0.254026489	1	0.996	1.43E-10	9.7E-15	
Piriform Cortex	iTBS	Atp6v0a1	ATPase, H+ transporting, lysosomal V0 subunit A1	11975	-0.250144227	0.786	0.846	0.01914904	1.3E-06	
Piriform Cortex	iTBS	Emc10	ER membrane protein complex subunit 10	69683	-0.255475018	0.797	0.863	0.000122005	8.3E-09	
Piriform Cortex	iTBS	Atp1a3	ATPase, Na+/K+ transporting, alpha 3 polypeptide	232975	-0.256273819	0.795	0.863	0.005698402	3.9E-07	
Piriform Cortex	iTBS	Eno2	enolase 2, gamma neuronal	13807	-0.265189061	0.956	0.971	0.003479113	2.4E-07	
Piriform Cortex	iTBS	Uqcrc1	ubiquinol-cytochrome c reductase core protein 1	22273	-0.26543376	0.778	0.861	0.002256156	1.5E-07	
Piriform Cortex	iTBS	Golga7b	golgi autoantigen, golgin subfamily a, 7B	71146	-0.272646814	0.442	0.571	0.00616993	4.2E-07	
Piriform Cortex	iTBS	Faim2	Fas apoptotic inhibitory molecule 2	72393	-0.280629639	0.734	0.785	0.001188851	8.0E-08	
Piriform Cortex	iTBS	Epn1	epsin 1	13854	-0.282835387	0.826	0.874	0.00390282	2.6E-07	
Piriform Cortex	iTBS	Dync1h1	dynein cytoplasmic 1 heavy chain 1	13424	-0.306294777	0.836	0.891	0.000116051	7.9E-09	
Piriform Cortex	iTBS	Diras2	DIRAS family, GTP-binding RAS-like 2	68203	-0.325331392	0.755	0.815	0.002439183	1.7E-07	
Piriform Cortex	iTBS	Phyhip	phytanoyl-CoA hydroxylase interacting protein	105653	-0.326178554	0.501	0.629	8.52E-05	5.8E-09	
Piriform Cortex	iTBS	Sptbn2	spectrin beta, non-erythrocytic 2	20743	-0.33193069	0.805	0.844	1.30E-05	8.8E-10	
Piriform Cortex	iTBS	Tuba1b	tubulin, alpha 1B	22143	-0.359977913	0.962	0.989	3.45E-13	2.3E-17	
Piriform Cortex	iTBS	Dynll2	dynein light chain LC8-type 2	68097	-0.405864738	0.933	0.962	1.35E-12	9.1E-17	
Piriform Cortex	iTBS	Eef1a2	eukaryotic translation elongation factor 1 alpha 2	13628	-0.454289962	0.983	0.994	3.90E-15	2.6E-19	
White Matter Tracts	iTBS	Gm10076	ribosomal protein L41 pseudogene	100126819	0.847062151	0.975	0.887	2.48E-46	1.7E-50	
White Matter Tracts	iTBS	Uba52	ubiquitin A-52 residue ribosomal protein fusion product 1	22186	0.57659486	0.991	0.958	1.98E-23	1.3E-27	
White Matter Tracts	iTBS	Gstp1	glutathione S-transferase, pi 1	14870	0.538358018	0.825	0.597	1.39E-12	9.4E-17	
White Matter Tracts	iTBS	Rps28	ribosomal protein S28	54127	0.334807146	0.987	0.995	1.79E-06	1.2E-10	
White Matter Tracts	iTBS	Rps21	ribosomal protein S21	66481	0.327145778	1	1	3.90E-11	2.6E-15	
White Matter Tracts	iTBS	Rps24	ribosomal protein S24	20088	0.312858966	1	1	1.35E-08	9.1E-13	
White Matter Tracts	iTBS	Enpp2	ectonucleotide pyrophosphatase/phosphodiesterase 2	18606	0.282580977	0.666	0.468	0.012315097	8.3E-07	
White Matter Tracts	iTBS	Rps10	ribosomal protein S10	67097	0.274498159	0.998	1	6.58E-06	4.5E-10	
White Matter Tracts	iTBS	Rpl35a	ribosomal protein L35A	57808	0.274475287	1	1	2.00E-05	1.4E-09	
White Matter Tracts	iTBS	Sf3b1	splicing factor 3b, subunit 1	81898	0.273697497	0.384	0.173	1.53E-09	1.0E-13	
White Matter Tracts	iTBS	Crym	crystallin, mu	12971	0.270578122	0.197	0.053	4.40E-09	3.0E-13	
White Matter Tracts	iTBS	Cab39	calcium binding protein 39	12283	0.269462269	0.307	0.108	4.10E-11	2.8E-15	
White Matter Tracts	iTBS	Rpl23	ribosomal protein L23	65019	0.26928313	1	1	8.69E-08	5.9E-12	
White Matter Tracts	iTBS	Hexb	hexosaminidase B	15212	0.26767016	0.44	0.222	3.43E-07	2.3E-11	
White Matter Tracts	iTBS	Rpl30	ribosomal protein L30	19946	0.267108506	1	1	9.13E-05	6.2E-09	
White Matter Tracts	iTBS	Cct3	chaperonin containing Tcp1, subunit 3 (gamma)	12462	0.257764942	0.439	0.213	8.20E-10	5.5E-14	
White Matter Tracts	iTBS	Cd47	CD47 antigen (Rh-related antigen, integrin-associated signal transducer)	16423	0.25602735	0.319	0.128	8.88E-09	6.0E-13	
White Matter Tracts	iTBS	Rps12	ribosomal protein S12	20042	0.250664118	1	0.998	0.000138898	9.4E-09	
White Matter Tracts	iTBS	Snx3	sorting nexin 3	54198	-0.256664141	0.791	0.877	0.002128645	1.4E-07	
Caudoputamen	iTBS	Gm10076	ribosomal protein L41 pseudogene	100126819	1.504442045	0.811	0.463	8.87E-131	6E-135	
Caudoputamen	iTBS	Uba52	ubiquitin A-52 residue ribosomal protein fusion product 1	22186	0.904690012	0.883	0.78	4.63E-81	3.1E-85	
Caudoputamen	iTBS	Gstp1	glutathione S-transferase, pi 1	14870	0.632915936	0.612	0.27	5.38E-56	3.6E-60	
Caudoputamen	iTBS	Rpl35	ribosomal protein L35	66489	0.36964948	0.985	0.965	1.36E-31	9.2E-36	
Caudoputamen	iTBS	Adora2a	adenosine A2a receptor	11540	0.300944336	0.78	0.614	1.49E-13	1.0E-17	
Caudoputamen	iTBS	Cnr1	cannabinoid receptor 1 (brain)	12801	0.292881538	0.681	0.532	3.39E-09	2.3E-13	
Caudoputamen	iTBS	Rps21	ribosomal protein S21	66481	0.283112319	1	1	4.45E-41	3.0E-45	
Caudoputamen	iTBS	Ppp1r1b	protein phosphatase 1, regulatory inhibitor subunit 1B	19049	0.261919919	0.997	0.991	2.19E-21	1.5E-25	
Caudoputamen	iTBS	Rpl35a	ribosomal protein L35A	57808	0.252392802	1	0.996	6.67E-24	4.5E-28	
Caudoputamen	iTBS	Tuba1b	tubulin, alpha 1B	22143	-0.256390985	0.671	0.833	1.34E-11	9.1E-16	
Caudoputamen	iTBS	Olfm1	olfactomedin 1	56177	-0.262932448	0.6	0.764	1.48E-12	1.0E-16	
Caudoputamen	iTBS	Vsnl1	visinin-like 1	26950	-0.281697653	0.333	0.544	1.47E-17	1.0E-21	
Caudoputamen	iTBS	Hpcal4	hippocalcin-like 4	170638	-0.289905788	0.525	0.596	1.98E-06	1.3E-10	
Lateral Septal Complex	iTBS	Gm10076	ribosomal protein L41 pseudogene	100126819	1.23638845	0.646	0.496	2.39E-15	1.6E-19	
Lateral Septal Complex	iTBS	Uba52	ubiquitin A-52 residue ribosomal protein fusion product 1	22186	0.752594811	0.833	0.705	7.13E-16	4.8E-20	
Lateral Septal Complex	iTBS	Rpl35	ribosomal protein L35	66489	0.444890654	0.997	0.948	3.81E-13	2.6E-17	
Lateral Septal Complex	iTBS	Rps28	ribosomal protein S28	54127	0.333394575	0.977	0.958	5.20E-05	3.5E-09	
Lateral Septal Complex	iTBS	Rps29	ribosomal protein S29	20090	0.310903719	1	1	0.00011808	8.0E-09	
Lateral Septal Complex	iTBS	Hectd4	HECT domain E3 ubiquitin protein ligase 4	269700	-0.253133208	0.334	0.514	0.019764442	1.3E-06	
Lateral Septal Complex	iTBS	Parp6	poly (ADP-ribose) polymerase family, member 6	67287	-0.256255699	0.232	0.41	0.005951657	4.0E-07	
Lateral Septal Complex	iTBS	Sgk1	serum/glucocorticoid regulated kinase 1	20393	-0.276265073	0.113	0.287	0.000143616	9.7E-09	
Lateral Septal Complex	iTBS	Luzp2	leucine zipper protein 2	233271	-0.287605523	0.402	0.561	0.016368979	1.1E-06	
Lateral Septal Complex	iTBS	Phyhip	phytanoyl-CoA hydroxylase interacting protein	105653	-0.317131003	0.289	0.483	0.000450812	3.1E-08	
Lateral Septal Complex	iTBS	Prkca	protein kinase C, alpha	18750	-0.338440643	0.264	0.426	0.017054536	1.2E-06	
Striatum Ventral Region	iTBS	Gm10076	ribosomal protein L41 pseudogene	100126819	1.338143498	0.733	0.59	7.19E-48	4.9E-52	
Striatum Ventral Region	iTBS	Uba52	ubiquitin A-52 residue ribosomal protein fusion product 1	22186	0.801778525	0.879	0.832	9.45E-38	4.9E-52	
Striatum Ventral Region	iTBS	Gstp1	glutathione S-transferase, pi 1	14870	0.478605517	0.584	0.368	7.52E-17	4.9E-52	
Striatum Ventral Region	iTBS	Rpl35	ribosomal protein L35	66489	0.305537969	0.984	0.972	2.08E-15	4.9E-52	
Striatum Ventral Region	iTBS	Rps28	ribosomal protein S28	54127	0.270465407	0.96	0.964	0.00011676	4.9E-52	
Striatum Ventral Region	iTBS	Actb	actin, beta	11461	-0.256477526	0.999	0.998	1.64E-07	4.9E-52	
Cortical Layer L2/3	cTBS	Gm10076	ribosomal protein L41 pseudogene	100126819	1.072233836	0.749	0.348	2.82E-49	1.9E-53	
Cortical Layer L2/3	cTBS	Uba52	ubiquitin A-52 residue ribosomal protein fusion product 1	22186	0.577826732	0.754	0.62	1.12E-14	7.6E-19	
Cortical Layer L2/3	cTBS	Rpl35	ribosomal protein L35	66489	0.478474403	0.974	0.9	8.76E-19	5.9E-23	
Cortical Layer L2/3	cTBS	Gstp1	glutathione S-transferase, pi 1	14870	0.353586231	0.445	0.236	4.81E-09	3.3E-13	
Cortical Layer L2/3	cTBS	Psd	pleckstrin and Sec7 domain containing	73728	0.300529449	0.96	0.881	7.35E-10	5.0E-14	
Cortical Layer L2/3	cTBS	Eno1	enolase 1, alpha non-neuron	13806	0.253005294	0.915	0.892	0.005054135	3.4E-07	
Cortical Layer L2/3	cTBS	Tuba1a	tubulin, alpha 1A	22142	-0.267269854	0.986	0.994	1.25E-07	8.4E-12	
Cortical Layer L2/3	cTBS	Tatdn1	TatD DNase domain containing 1	69694	-0.269090494	0.111	0.306	6.28E-09	4.3E-13	
Cortical Layer 4	cTBS	Gm10076	ribosomal protein L41 pseudogene	100126819	0.745341144	0.705	0.406	5.11E-22	3.5E-26	
Cortical Layer 4	cTBS	Uba52	ubiquitin A-52 residue ribosomal protein fusion product 1	22186	0.434269433	0.786	0.731	2.02E-05	1.4E-09	
Cortical Layer 4	cTBS	Rpl35	ribosomal protein L35	66489	0.397464177	0.974	0.953	1.36E-11	9.2E-16	
Cortical Layer 4	cTBS	Selenop	selenoprotein P	20363	-0.294902507	0.412	0.558	0.022384033	1.5E-06	
Cortical Layer 5	cTBS	Gm10076	ribosomal protein L41 pseudogene	100126819	0.995523431	0.704	0.341	1.61E-73	1.1E-77	
Cortical Layer 5	cTBS	Uba52	ubiquitin A-52 residue ribosomal protein fusion product 1	22186	0.533661504	0.787	0.685	7.44E-22	5.0E-26	
Cortical Layer 5	cTBS	Rpl35	ribosomal protein L35	66489	0.516119763	0.981	0.918	1.51E-41	1.0E-45	
Cortical Layer 5	cTBS	Gstp1	glutathione S-transferase, pi 1	14870	0.278587356	0.43	0.313	1.77E-05	1.2E-09	
Cortical Layer 5	cTBS	Polr2l	polymerase (RNA) II (DNA directed) polypeptide L	66491	0.266333438	0.583	0.476	7.44E-05	5.0E-09	
Cortical Layer 5	cTBS	Cdk5r2	cyclin-dependent kinase 5, regulatory subunit 2 (p39)	12570	-0.284455074	0.39	0.553	1.58E-10	1.1E-14	
Cortical Layer 6	cTBS	Gm10076	ribosomal protein L41 pseudogene	100126819	0.75879212	0.767	0.559	3.37E-30	2.3E-34	
Cortical Layer 6	cTBS	Gstp1	glutathione S-transferase, pi 1	14870	0.474468605	0.512	0.285	5.93E-12	4.0E-16	
Cortical Layer 6	cTBS	Uba52	ubiquitin A-52 residue ribosomal protein fusion product 1	22186	0.46715186	0.843	0.832	9.63E-11	6.5E-15	
Cortical Layer 6	cTBS	Rpl35	ribosomal protein L35	66489	0.37508774	0.982	0.985	3.28E-14	2.2E-18	
Piriform Cortex	cTBS	Gm10076	ribosomal protein L41 pseudogene	100126819	1.095206033	0.758	0.423	1.12E-41	7.6E-46	
Piriform Cortex	cTBS	Uba52	ubiquitin A-52 residue ribosomal protein fusion product 1	22186	0.61141369	0.779	0.701	1.70E-15	1.2E-19	
Piriform Cortex	cTBS	Rpl35	ribosomal protein L35	66489	0.547358357	0.972	0.958	1.05E-20	7.1E-25	
Piriform Cortex	cTBS	Polr2l	polymerase (RNA) II (DNA directed) polypeptide L	66491	0.36691555	0.607	0.429	5.11E-07	3.5E-11	
Piriform Cortex	cTBS	Gstp1	glutathione S-transferase, pi 1	14870	0.331980422	0.534	0.354	6.30E-06	4.3E-10	
Piriform Cortex	cTBS	Rpl38	ribosomal protein L38	67671	0.32644671	1	1	9.83E-21	6.6E-25	
Piriform Cortex	cTBS	Cox7c	cytochrome c oxidase subunit 7C	12867	0.323853069	1	0.998	1.34E-17	9.1E-22	
Piriform Cortex	cTBS	Atp5md	ATP synthase membrane subunit DAPIT	66477	0.317035604	1	1	1.43E-18	9.7E-23	
Piriform Cortex	cTBS	Tmsb10	thymosin, beta 10	19240	0.306765588	0.988	1	2.26E-06	1.5E-10	
Piriform Cortex	cTBS	Rps21	ribosomal protein S21	66481	0.288886951	1	1	3.95E-15	2.7E-19	
Piriform Cortex	cTBS	Rps28	ribosomal protein S28	54127	0.270532904	0.929	0.857	4.85E-05	3.3E-09	
Piriform Cortex	cTBS	Ndufb4	NADH:ubiquinone oxidoreductase subunit B4	68194	0.257858789	0.951	0.874	5.04E-05	3.4E-09	
Piriform Cortex	cTBS	Agap2	ArfGAP with GTPase domain, ankyrin repeat and PH domain 2	216439	-0.25389471	0.228	0.385	0.000966429	6.5E-08	
Piriform Cortex	cTBS	Cdk5r2	cyclin-dependent kinase 5, regulatory subunit 2 (p39)	12570	-0.280578006	0.428	0.554	0.011840037	8.0E-07	
Piriform Cortex	cTBS	Phyhip	phytanoyl-CoA hydroxylase interacting protein	105653	-0.294262305	0.527	0.629	0.006444959	4.4E-07	
Piriform Cortex	cTBS	Dynll2	dynein light chain LC8-type 2	68097	-0.342342006	0.944	0.962	4.54E-08	3.1E-12	
White Matter Tracts	cTBS	Gstp1	glutathione S-transferase, pi 1	14870	0.685173258	0.707	0.597	3.50E-11	2.4E-15	
White Matter Tracts	cTBS	Gm10076	ribosomal protein L41 pseudogene	100126819	0.565014967	0.938	0.887	5.47E-20	3.7E-24	
White Matter Tracts	cTBS	Fth1	ferritin heavy polypeptide 1	14319	0.517891522	1	1	5.79E-25	3.9E-29	
White Matter Tracts	cTBS	Uba52	ubiquitin A-52 residue ribosomal protein fusion product 1	22186	0.485056077	0.968	0.958	1.50E-12	1.0E-16	
White Matter Tracts	cTBS	Rapgef4	Rap guanine nucleotide exchange factor (GEF) 4	56508	0.367187139	0.843	0.795	1.53E-05	1.0E-09	
White Matter Tracts	cTBS	Kif5a	kinesin family member 5A	16572	0.31720512	0.992	0.99	1.21E-05	8.2E-10	
White Matter Tracts	cTBS	Ftl1	ferritin light polypeptide 1	14325	0.305588024	1	1	3.24E-07	2.2E-11	

Data S9

M1 iTBS L2/3

DEGs Young sham vs Aged sham	iTBS DEGs unique to young adults	iTBS DEGs unique to young adults that are also ageing DEGs
Gapdh	Gstp1	
Psd	Chd9	
Mt1	Ncald	
Ndufa4	Tmsb10	
Nme7	Meg3	
Actb	Cdk2ap1	
Cox8a	Gm2000	
Malat1		
Rpl4		
Rpl5		
Lars2		
Calm1		
Rps29		
Mbp		
Rps28		
Cdk11b		
Rbm3		
Dpysl2		
Ctxn1		
Ppia		
Itm2c		
Psap		
Mt2		
Lrrc17		
Slc1a2		
Sptbn2		
Txndc15		
Ppp3r1		
Homer1		
Pfn1		
Eef1a2		
Arl8a		
Ddn		
Ewsr1		
Ppp2r1a		
Stx1a		
Cab39		
Stmn3		
Morf4l1		
Ptges3		
Pkm		
Smim19		
Rps27		
Snrnp70		
Ogfrl1		
Rpl37a		
Rps15a		
Cspg5		
Ivns1abp		
Abcf1		
Zfp365		
Arc		
Lypla2		
Tuba1b		
Set		
Nrxn3		
Dynll2		
Cdc37		
Ilf3		
Capza2		
Pcp4		
AW047730		
Rpl3		
Ikbkb		
Pja2		
Ccar2		
Ctsa		
Gramd1a		
Snhg6		
Bex2		
Pfdn5		
Gmpr		
Kif5a		
Insig1		
Atp1a1		
Dek		
Anp32a		
Rps24		
Nfkb2		
Smarcc2		
Ndn		
Rpl9-ps6		
Gabbr1		
Alkbh7		
Cirbp		
Ddx6		
Spint2		
Cox7b		
Actr3		
Maco1		
Map1s		
Eml2		
Rnf41		
St3gal5		
Sptbn1		
Tmem130		
Zwint		
Maged1		
Rps20		
Bcl7a		
Fam107a		
Rps2		
Ptprj		
Rac1		
Galnt16		
Rpn1		
Arfgap1		
Camk2b		
Ctsb		
Ppp2r5c		
Smarcb1		
Tubb5		
Usp11		
Poglut1		
Phyhip		
Pcbp2		
Bc1		
Puf60		
Spin1		
Ndrg2		
Cluh		
Ssrp1		
Cort		
Pantr1		
Usp50		
Ube3c		
Gm10076		
Prdx6		
Mical2		
Eif6		

M1 cTBS L2/3

DEGs Young sham vs Aged sham	cTBS DEGs unique to young adults	cTBS DEGs unique to young adults that are also ageing DEGs
Uba52	Lrrc17	Bcl7a
Gapdh	Rps28	Cox8a
Psd	Nrn1	Ctsb
Mt1	Tspyl4	Ctxn1
Ndufa4	Snca	Ddn
Nme7	Rps29	Eef1a2
Actb	Pacsin1	Gapdh
Cox8a	Snap25	Lrrc17
Malat1	Fam131a	Mical2
Rpl4	Ost4	Mt1
Rpl5	Cox17	Mt2
Lars2	Araf	Pfdn5
Calm1	Tomm7	Phyhip
Rps29	Ddn	Psd
Mbp	Snrpf	Rpl37a
Rps28	Ndufa1	Rpl4
Cdk11b	Cox7c	Rpl5
Rbm3	Isca1	Rpl9-ps6
Dpysl2	Dmtn	Rps15a
Ctxn1	Rps27	Rps20
Ppia	Snhg8	Rps24
Itm2c	Txn1	Rps27
Psap	Actr2	Rps28
Mt2	Snhg6	Rps29
Lrrc17	Gng13	Snhg6
Slc1a2	Kctd13	Sptbn2
Rpl35	Slc6a1	Stx1a
Sptbn2	Nptx2	Uba52
Txndc15	Atp6v1a	Usp50
Ppp3r1	Sdhd	Zfp365
Homer1	Atp5md	Zwint
Pfn1	Elmo2	
Eef1a2	Snhg9	
Arl8a	Zwint	
Ddn	Agap2	
Ewsr1	Usp50	
Ppp2r1a	Lmo4	
Stx1a	Dnajb5	
Cab39	Sema7a	
Stmn3	Arpp21	
Morf4l1	Hpcal4	
Ptges3	Naa20	
Pkm	Sem1	
Smim19	Nr4a1	
Rps27	Schip1	
Snrnp70	Wsb2	
Ogfrl1	Megf9	
Rpl37a	Atp1a3	
Rps15a	Smim26	
Cspg5	Sipa1l1	
Ivns1abp	Zfp365	
Abcf1	Slc25a22	
Zfp365	Camk2a	
Arc	Stxbp1	
Lypla2	App	
Tuba1b	Ttc7b	
Set	Shank1	
Nrxn3	Plppr4	
Dynll2	Atn1	
Cdc37	Rpl37a	
Ilf3	Ppp6r1	
Capza2	Cox8a	
Pcp4	Vxn	
AW047730	Fam131b	

Rpl3	Stum
Ikbkb	Pam
Pja2	Slc4a10
Ccar2	Fbxl16
Ctsa	Rab3c
Gramd1a	Gas7
Snhg6	Lmtk2
Bex2	Phyhip
Pfdn5	Cops7a
Gmpr	Ppm1h
Kif5a	Gucy1b1
Insig1	Kcnh3
Atp1a1	Nell2
Dek	Rnf5
Anp32a	Dusp7
Rps24	Vgf
Nfkb2	Lpcat4
Smarcc2	Huwe1
Ndn	Jph4
Rpl9-ps6	Mgat3
Gabbr1	Sptbn2
Alkbh7	Sprn
Cirbp	Rusc2
Ddx6	F3
Spint2	Cacna2d1
Cox7b	Rpl9-ps6
Actr3	Spag9
Maco1	Cinp
Map1s	Dnm1
Eml2	Psd
Rnf41	Ncald
St3gal5	Syngap1
Sptbn1	Senp3
Tmem130	Mat2b
Zwint	Inpp4a
Maged1	Dnajc21
Rps20	Tsnax
Bcl7a	Comt
Fam107a	Rgs7bp
Rps2	Cmpk1
Ptprj	Cs
Rac1	Atp6v1h
Galnt16	Gnai1
Rpn1	Ccnb1
Arfgap1	Phpt1
Camk2b	Gm1673
Ctsb	Dkk3
Ppp2r5c	Chst2
Smarcb1	Pip5k1c
Tubb5	Chd9
Usp11	Kif3c
Poglut1	Dnajb4
Phyhip	Bloc1s1
Pcbp2	Csnk2a2
Bc1	Junb
Puf60	1810037I17Rik
Spin1	Dab2ip
Ndrg2	Eif4a2
Cluh	Dnajc6
Ssrp1	Slc8a2
Cort	Psma5
Pantr1	Hectd4
Usp50	Mical2
Ube3c	Cadm4
Gm10076	Klc2
Prdx6	Eef1a2
Mical2	Fgfr1op2

Eif6	Car2
	Phyhipl
	Unc5a
	Ank2
	Rap2a
	Usp19
	Arpc4
	Rheb
	Abhd8
	Tsc22d4
	Dact3
	Hivep2
	Numb
	Mt1
	Itpka
	Ndufa3
	Ctxn1
	Atxn2l
	Zmiz1
	Bhlhe40
	Brms1l
	Rtn4r
	Glul
	Nrxn2
	Cux2
	Ndrg4
	Cck
	Cfap20
	Erh
	Gpr26
	Sv2b
	Acyp2
	Nfix
	Tmem196
	Spred1
	Camk1g
	Frrs1l
	Bccip
	Sft2d1
	Ppp1r9b
	Stx1a
	B230334C09Rik
	Ncan
	Bzw1
	Cacng3
	Atxn2
	Rai1
	Rps27rt
	Lzts3
	Pofut2
	Higd2a
	Ubqln2
	Prkaca
	Pou3f3
	Insyn1
	Cnksr2
	Wwc1
	Enc1
	Med31
	Dusp6
	Nutf2-ps1
	Ppme1
	Apba1
	Spcs3
	Tacc1
	Spry2
	Rimbp2

Clcn2
Lnpep
Gja1
U2af2
Cd47
Tmeff1
Ano3
Add2
Bcl2l2
Jdp2
Arhgap32
Phactr1
Hprt
Slc30a3
Rapgef2
Btbd10
Psip1
Tiprl
B3gat1
Unc50
Cacng7
Herc3
Rgcc
Ndfip2
Cd302
Myadm
Yme1l1
Arhgap39
Klf13
Rnd1
Arhgef12
Prrc2a
Ank
Ppp2cb
Sptan1
Lsm8
Rab28
Kcna2
Mif
Ogdhl
Spop
Rraga
Prex1
Golph3
Mt2
Frmpd4
Ptms
Trappc2
Pde4dip
Yaf2
Ndufb3
Stard8
Arhgap1
Coa6
Rgs6
Mt3
Cntnap1
Skil
Nbl1
Nrgn
Mrps28
Sncb
Ryr2
Mapre2
Hopx
Med25
Romo1

Bcl7a
Prpf8
Fus
Pmpca
Irak1bp1
Cyth1
Nptxr
Trrap
Srsf5
Thra
Mgat5b
Scn2a
Gnao1
Rpl39
Lrrc57
Ccn3
Chrm3
Usp7
Etv5
Syt17
Dgkz
Fam81a
Ccnh
Ahcyl2
Car10
Usp9x
Acap2
Ppp2r2c
Rapgefl1
Myrip
Grk6
Trp53i11
Samd8
Pin1
Tmed7
L1cam
Rasgef1c
Pcdh10
Lingo1
Luzp2
Dpf1
Nova2
Lamtor3
Crip2
Arpp19
Ssbp4
Cds2
Exoc1
Abhd2
Usp12
Kitl
Atxn1
Msi2
Irs2
Rragd
Acat1
Mthfsl
Rcn2
Mpv17
Rnasek
Rpl10-ps3
Rbbp6
Cnnm1
Lrfn5
Syn1
Insig2
Prkar1a

Cpeb2
Klhl9
Mfsd14a
Polr3k
Hmgb1
Fam126b
Zfr
Kmt2d
Ap1ar
Yipf4
Kdm7a
Cnep1r1
Eif4g1
Slc17a7
Gfra4
Scn1a
Wdr89
Kcnh1
Arnt2
Rnf114
Sgsm2
Homer2
Kcnv1
Npas2
Bpnt1
Ccdc32
Nae1
Olfm2
5730455P16Rik
Gtpbp2
Pde4b
Rabggtb
Stx4a
Ramp2
Tmem178b
Gabra3
Katnal1
Atp6v1b2
Cggbp1
Clip1
Map6d1
Usp25
Fjx1
Cdc42se2
Fmn2
Mtln
Cstb
Mtmr9
Atp5mpl
Rtn4rl1
Pde6d
Gas6
Spred2
Smim4
Kctd1
Pld3
Stim2
Cadm1
Mpp3
Socs7
Lsm3
Llgl1
Orc6
Thrb
Copb1
Prkci
Rock2

Pgls
Klhl29
Ifngr2
Abhd3
Cul2
Ulk1
Gm20300
Ptprf
Idi1
Pmpcb
Stx1b
Ctnnd1
Gramd4
Dvl1
Lst1
Meis2
Ttyh3
Fam13c
Rgs20
Shank3
Pank4
Ehd1
Ak4
Dagla
Shd
Hes5
Acvr1b
Slc4a4
Pcsk1
Rnf141
Erg28
Trim8
Tmem184c
Ube2q1
Ift57
Ccdc6
Mrpl27
Srrm1
Sdf2
Lin52
Arhgef10l
Smim15
Rabl6
Cebpb
Cxcl12
C130071C03Rik
Sfxn1
Atp6v1g2
Khsrp
Dhx9
Rgs14
Rnf146
Myef2
Nme3
Zdhhc14
Tmed2
Iars2
Nmt2
Dcakd
Satb2
Mtx2
Raph1
Sh3bp5l
Rnf123
Tnnc1
Fam241b
Tcea1

Setd5
Coq5
Zfp445
Nol4
Neurl1b
Rpl7l1
Hspa14
Alas1
Spats2l
Hnrnpf
Clasp2
Lix1
Rap1a
Pitpnm3
Tbc1d10a
Polr2h
Cebpg
Cd200
Gspt1
Tbl1xr1
Shprh
Jmjd1c
Hba-a2
Gapdh
Rps5
Ndufa13
Eef1a1
Rpl31
Rps17
Rpsa
Rpl13
Gabarap
Rpl35a
Rpl30
Hsp90aa1
Pfdn5
Ctsb
Rpl9
Rps11
Fkbp2
Rps4x
Chgb
Rps12
Rpl26
Ppp3ca
Gabarapl2
Gpr88
Rpl6
Naca
Rpl11
Rps15a
Fau
Rps3a1
Rpl7
Mrfap1
Nenf
Rpl13a
Rpl14
Pink1
Rpl24
Mobp
Rps7
Rpl4
Rpl21
Nefm
Rpl10
Rps20

Rps8
Cryab
Rpl5
Rps27a
Rps24
Hbb-bs

M1 iTBS L5

DEGs Young sham vs Aged sham	iTBS DEGs unique to young adults	iTBS DEGs unique to young adults that are also ageing DEGs
Uba52	Uba52	Bc1
Rpl4	Gstp1	Chd9
Gapdh	Mobp	Gm10076
Rps29	Chd9	Mbp
Mt1	Mt2	Mobp
Cox8a	Dbi	Mt1
Malat1	Eno1	Mt2
Snhg6	Slc1a3	Rps27
Rps28	Mbp	Uba52
Lrrc17	Mt1	
Psd	Fbxl16	
Mt2	Dbndd2	
Slc1a2	Rps27	
Rps24	Pitpnm1	
Rpl5	Sncb	
Cox7b	Gm2000	
Nme7	Bc1	
Usp50		
Dpysl2		
Rph3a		
Fam107a		
Ndufa4		
Actb		
Rpl9-ps6		
Lmtk2		
Sema7a		
Fau		
Zwint		
Ctxn1		
Mfge8		
Arc		
Itm2c		
Mbp		
Homer1		
Rpl24		
Rpl21		
Lars2		
Cspg5		
Eef1a1		
Pcp4		
Rps27a		
Sst		
Zfp365		
Mobp		
Mical2		
Ndufa3		
Stmn2		
Kif5a		
Eef1a2		
Rpl37a		
Ddn		
Ivns1abp		
Rps2		
Rps27		
Gm10076		
Gnb2		

Plcxd2
Bc1
Egr3
Dclk1
Kifc2
Gpr26
Rpl35
Pip5k1c
Pfn1
Ndufv1
Eno1b
Myadm
Mif
Tomm7
Atp6v1g2
Ypel3
Rpl8
Cplx1
Tspyl4
Ndrg2
Scn1a
Pak1
Rbm3
Gtf3c3
Rims1
Cyb5r4
Rps7
Ina
Rnasek
Sptbn1
Rpl9
Rps8
Cinp
Adcy1
Dusp7
Hspa5
Ost4
Hecw1
Atxn2l
Sptbn2
Fxyd7
Rundc3a
Camk1g
Utrn
Ndufa7
Kif5c
Nptx2
Rpsa
Ptma
Rapgef4
Rpl3
Tmem160
Pfdn5
Capzb
Atp1a1
Oaz1
Nrxn3
Napb
Kcnj10

Stac2
Sptan1
Galnt9
Asap2
Rab31
Kif21a
Ewsr1
Stx1a
Ids
Stxbp1
Rpl14
Pfdn2
Gde1
Hpcal4
Dctn5
Camk2a
Rps4x
Vgf
Eif4a1
Dynll2
Arl8a
Hs3st2
Dok6
Chd9
Hivep2
Saxo2
Acsl5
Aff3
Kif21b
Hsph1
Agap2
Ntsr2
Rps3a1
Gfod1
Csf1r
BC005537
Stradb
Asap1
Ubap2l
Ly6e
Tfrc
Tlcd1
Slc32a1
Atxn2
Tpp1
Hnrnpa2b1
Washc5
Jund
Sgsm1
Fkbp1b
Nucks1
Nme2
Rpl6
Car10
Plk2
Aldh1a1
Man1c1
Ipo7
Kcnv1

Bex1

M1 cTBS L5

DEGs Young sham vs Aged sham	cTBS DEGs unique to young adults	cTBS DEGs unique to young adults that are also ageing DEGs
Uba52	Lrrc17	Agap2
Rpl4	Rps28	Atxn2
Gapdh	Tspyl4	Atxn2l
Rps29	Snap25	Bex1
Mt1	Pacsin1	Camk2a
Cox8a	Rps29	Chd9
Malat1	Actr2	Cinp
Snhg6	Snca	Cox8a
Rps28	Nrn1	Cplx1
Lrrc17	Glul	Ddn
Psd	Tomm7	Dusp7
Mt2	Camk2a	Eef1a1
Slc1a2	Atp6v1a	Eno1b
Rps24	Slc25a22	Ewsr1
Rpl5	Dmtn	Fau
Cox7b	Ndufa1	Gapdh
Nme7	App	Gpr26
Usp50	Agap2	Hnrnpa2b1
Dpysl2	Zwint	Hpcal4
Rph3a	Tatdn1	Ids
Fam107a	Ddn	Jund
Ndufa4	Cox17	Kif5a
Actb	Ost4	Kif5c
Rpl9-ps6	Araf	Kifc2
Lmtk2	Huwe1	Lmtk2
Sema7a	Snhg9	Lrrc17
Fau	Rap1gds1	Mical2
Zwint	Snrpf	Mt1
Ctxn1	Cplx2	Mt2
Mfge8	Ndrg4	Myadm
Arc	Snhg6	Ndufa3
Itm2c	Phpt1	Ndufa7
Mbp	Sdhd	Nptx2
Homer1	Cops7a	Oaz1
Rpl24	Stxbp1	Ost4
Rpl21	Ank	Pak1
Lars2	Slc6a1	Pfdn5
Cspg5	Txn1	Pip5k1c
Eef1a1	Chd9	Psd
Pcp4	Usp50	Rapgef4
Rps27a	Mt2	Rnasek
Sst	Isca1	Rph3a
Zfp365	Cntnap1	Rpl14
Mobp	Eif4a2	Rpl21
Mical2	Hpcal4	Rpl24
Ndufa3	Frrs1l	Rpl3
Stmn2	Rapgef4	Rpl4
Kif5a	Rab3c	Rpl5
Eef1a2	Arpp21	Rpl6
Rpl37a	Snhg8	Rpl8
Ddn	Kctd13	Rpl9
Ivns1abp	Ppp1r9b	Rpl9-ps6
Rps2	Fbxl16	Rps2
Rps27	Zfp365	Rps24
Gm10076	Phactr1	Rps27a
Gnb2	Rgs7bp	Rps28

Plcxd2
Bc1
Egr3
Dclk1
Kifc2
Gpr26
Rpl35
Pip5k1c
Pfn1
Ndufv1
Eno1b
Myadm
Mif
Tomm7
Atp6v1g2
Ypel3
Rpl8
Cplx1
Tspyl4
Ndrg2
Scn1a
Pak1
Rbm3
Gtf3c3
Rims1
Cyb5r4
Rps7
Ina
Rnasek
Sptbn1
Rpl9
Rps8
Cinp
Adcy1
Dusp7
Hspa5
Ost4
Hecw1
Atxn2l
Sptbn2
Fxyd7
Rundc3a
Camk1g
Utrn
Ndufa7
Kif5c
Nptx2
Rpsa
Ptma
Rapgef4
Rpl3
Tmem160
Pfdn5
Capzb
Atp1a1
Oaz1
Nrxn3
Napb
Kcnj10
Schip1
Ank2
Smim26
Rpl9-ps6
Rnf5
Lmo4
Dnajc6
Fus
Naa20
Sst
Atn1
Wsb2
Pip5k1c
Rph3a
Sem1
Cs
Sema7a
Nptx2
Gja1
Prrc2b
Slc1a3
Unc5a
Kif3c
Fam131a
Atp5g3
Sptbn1
Ppp1r16b
Spag9
Psip1
Prrc2a
Ids
Atp5md
Lpcat4
Mical2
Slc4a10
Klc2
R3hdm4
Ewsr1
Sptbn2
Fam131b
Rusc2
Eps15
Ndufa3
1810037I17Rik
Brms1l
Spop
Gucy1b1
Phyhipl
Shank1
Nrxn1
Psd3
Bloc1s1
Sipa1l1
Atp6v1h
Luzp2
Kif5c
Map2
Phyhip
Syn1
Rps29
Rps3a1
Rps4x
Rps7
Rps8
Rpsa
Saxo2
Scn1a
Sema7a
Slc1a2
Snhg6
Sptan1
Sptbn1
Sptbn2
Sst
Stxbp1
Tomm7
Tspyl4
Ubap2l
Usp50
Vgf
Zfp365
Zwint

Stac2	Ahcyl2
Sptan1	Mgat3
Galnt9	Herc3
Asap2	Rcn2
Rab31	Ndfip2
Kif21a	Chst2
Ewsr1	Paip2
Stx1a	Car2
Ids	Sptan1
Stxbp1	Kif5a
Rpl14	Gnao1
Pfdn2	Srsf5
Gde1	Atxn2l
Hpcal4	Inpp4a
Dctn5	Msi2
Camk2a	Atxn2
Rps4x	Lmtk2
Vgf	Dusp7
Eif4a1	Ubqln2
Dynll2	Ttc7b
Arl8a	Arhgef12
Hs3st2	Comt
Dok6	Psma5
Chd9	Ccnb1
Hivep2	Lynx1
Saxo2	Gng13
Acsl5	Scn1a
Aff3	Fgfr1op2
Kif21b	Syt1
Hsph1	Mapk1
Agap2	Apba1
Ntsr2	Rabgap1l
Rps3a1	Arrb1
Gfod1	Spock2
Csf1r	Add2
BC005537	Rheb
Stradb	Gnai1
Asap1	Tacc1
Ubap2l	Tsc22d3
Ly6e	Sparcl1
Tfrc	Eno1b
Tlcd1	Ptprz1
Slc32a1	Panx2
Atxn2	Syngr1
Tpp1	Mapre2
Hnrnpa2b1	Ptn
Washc5	Hectd4
Jund	Bpnt1
Sgsm1	Romo1
Fkbp1b	Bhlhe40
Nucks1	Rabggtb
Nme2	Kcna2
Rpl6	Gpr26
Car10	Slc4a4
Plk2	Slc8a2
Aldh1a1	Kifc2
Man1c1	Cstb
Ipo7	Scg2
Kcnv1	Pdpk1

Bex1	Apc
	Stx1b
	Erc2
	Lamp1
	Psd
	Tcf4
	Klf13
	Cd47
	Nell2
	Ctnnb1
	Ssb
	Ppm1h
	Cmpk1
	Tmeff1
	Syngap1
	Dgkz
	Ncald
	Insyn1
	Dusp6
	Gria2
	Sprn
	Klf9
	Megf9
	Idi1
	Cox8a
	Senp3
	Acyp2
	Churc1
	Myadm
	Wdr89
	S1pr1
	Dnm1
	Arhgap39
	Selenof
	Ppme1
	Cds2
	Numb
	Stum
	Ttyh1
	Iars2
	Kcnh1
	Prkar1a
	Pde4dip
	Tmed7
	Tef
	Dnajb4
	Ap1ar
	Sft2d1
	Reep1
	Lrp11
	Nedd4
	Dnajb5
	Brsk1
	Elavl3
	Clasp2
	Actn4
	Ogdhl
	Fam126b
	Slc12a5

Rnf157
Vxn
Cinp
Arpc4
Cadm4
Acat1
Ssr3
Dab2ip
Dbndd2
Camk2n1
Pik3r1
Mfsd14a
Hopx
Plppr4
Scn4b
Oxr1
Tiprl
Hnrnpa2b1
Gpm6b
Rnf114
Pcdh1
F3
Pitpnm3
Socs7
Ccser2
Kcnh3
B230312C02Rik
Cpeb2
Bcl2l2
Snx5
Arhgef4
Usp31
Sdf2
Rab28
Atf2
Ndufa7
Dhx9
Rpl10-ps3
Dnal1
Vgf
Myrip
Cfap20
Cplx1
Hibadh
Aftph
Add3
Arhgef10l
Trrap
Rps27rt
Rpl39
Ppp2cb
Slc1a2
Rnasek
Fmn2
Kif1a
Slc36a1
Tmem128
Pmpcb
Gas6

Unc50
Cyth1
Fam120a
Epb41l1
Ldb1
Cd302
Gtpbp2
Ube2d1
Mrps28
Samd8
Mrpl10
Ppp1r12b
Alas1
Pdcd6ip
Ccng2
Ubap2l
Rnf19b
Mt1
Abhd2
Hace1
Dock4
Vstm2b
Fbrsl1
Ppp2r2c
Ttbk2
Nae1
Npc1
Ifngr2
Adgrl2
Spry2
Pank4
Stox2
Spns2
Saxo2
Aagab
A830018L16Rik
Pak1
Arpp19
Nadk
Atp5d
Mpc2
Rpl3
Actg1
Eef1a1
Ftl1
Rpl23a
Atp5o
Chchd2
Rps13
Rpl31
Rps5
Oaz1
Rpl23
Ubb
Ndufa2
Ndufb11
Rpl19
Rpl35a
Pebp1

Jund
Tpt1
Rps16
Dynll1
Rps3
Hba-a1
Rpl27a
Atp9a
Rps14
Gabarapl2
Rps15a
Ndufb10
Rpl32
Rps15
Rps3a1
Selenom
Rpsa
Fkbp2
Smdt1
Rps4x
Bex1
Hras
Rpl17
Rps10
Rps11
Ndufb9
Gapdh
Eno1
Rpl7
Rpl6
Ndufb8
Ndufa13
Rpl26
Rpl18
Rpl8
Rpl30
Rpl5
Rpl11
Rps12
Rpl18a
Rpl14
Nenf
Rpl24
Pfdn5
Rpl13a
Rpl13
Rps20
Rpl9
Rpl4
Myl4
Fau
Rps2
Rps7
Rpl21
Rps8
Rps27a
Rps24
Rpl10
Hbb-bs

M1 iTBS L6

DEGs Young sham vs Aged sham	iTBS DEGs unique to young adults	iTBS DEGs unique to young adults that are also ageing DEGs
Mobp	Penk	Gapdh
Uba52	Dbi	Rps27
Mbp	Ptn	
Pcp4	Gpr88	
Gapdh	Rps27	
Rps29	Stmn3	
Lrrc17	Gapdh	
Fau	Akt1s1	
Rpl9-ps6	Fbxl19	
Rps24	Gprin1	
Ost4	Hapln4	
Kif5a	Ttc9b	
Ighm		
Rpl4		
Epas1		
Ccn2		
Cox8a		
Dpysl2		
Slc1a2		
Rps28		
Eef1a1		
Rpl5		
Ro60		
Tmem167		
Pacsin1		
Hivep2		
Ctxn1		
Rph3a		
Rps27a		
Rpl21		
Rps2		
Far1		
Mtmr9		
Gadd45a		
Zdhhc14		
Tatdn1		
Trp53inp2		
Asap1		
Mical2		
Flcn		
Unc80		
Kctd1		
Usp50		
Pitpnc1		
Cldn11		
Setd7		
Snhg12		
Arhgap39		
Snx6		
Arc		
Stac2		
Ndfip2		
Kcnk1		
Man1a2		
Micu1		
Tsc22d3		

Camkk1
Myo6
Ccp110
Nrbp2
Tnk2
Nme7
Gnai1
Tspan2
Large1
Hacd1
Psd
Gapvd1
Rapgef1
Rps27
Synrg
Cisd2
Efna3
Mal
Dvl3
Vps37a
Rps7
Sf3b4
Ptp4a3
Prss23
Tnks2
Tbc1d20
Sesn1
Adcy1
Evi2a
Itgb1
Ppp2r5b
Agap2

M1 cTBS L6

DEGs Young sham vs Aged sham	cTBS DEGs unique to young adults	cTBS DEGs unique to young adults that are also ageing DEGs
Mobp	Lrrc17	Agap2
Uba52	Basp1	Ccn2
Mbp	Rps28	Eef1a1
Pcp4	Pacsin1	Fau
Gapdh	Tspyl4	Gapdh
Rps29	Ccn2	Kif5a
Lrrc17	Rps29	Lrrc17
Fau	Ost4	Mtmr9
Rpl9-ps6	Snca	Ndfip2
Rps24	Araf	Ost4
Ost4	Snap25	Pacsin1
Kif5a	Ndufa1	Rpl21
Ighm	Glul	Rpl4
Rpl4	Agap2	Rpl5
Epas1	Atp1a3	Rpl9-ps6
Ccn2	Dmtn	Rps2
Cox8a	Tatdn1	Rps24
Dpysl2	Rapgef4	Rps27
Slc1a2	Tomm7	Rps27a
Rps28	Snhg9	Rps28
Eef1a1	Cox17	Rps29
Rpl5	Phactr1	Rps7
Ro60	Gas7	Tatdn1
Tmem167	App	Usp50
Pacsin1	Isca1	
Hivep2	Slc6a1	
Ctxn1	Car2	
Rph3a	Rps27	
Rps27a	Snrpf	
Rpl21	Zwint	
Rps2	Ndfip2	
Far1	Ank2	
Mtmr9	Rpl9-ps6	
Gadd45a	Smim26	
Zdhhc14	Kctd13	
Tatdn1	Srsf5	
Trp53inp2	Slc25a22	
Asap1	Ewsr1	
Mical2	Ddn	
Flcn	Hpcal4	
Unc80	Ndrg4	
Kctd1	Sem1	
Usp50	Phpt1	
Pitpnc1	Fam131b	
Cldn11	Cox7c	
Setd7	Huwe1	
Snhg12	Ank	
Arhgap39	Arpp21	
Snx6	Dnajc6	
Arc	Sdhd	
Stac2	Fam131a	
Ndfip2	Klf13	
Kcnk1	Rap1gds1	
Man1a2	Fam168a	
Micu1	Snhg8	
Tsc22d3	Eif4a2	

Camkk1
Myo6
Ccp110
Nrbp2
Tnk2
Nme7
Gnai1
Tspan2
Large1
Hacd1
Psd
Gapvd1
Rapgef1
Rps27
Synrg
Cisd2
Efna3
Mal
Dvl3
Vps37a
Rps7
Sf3b4
Ptp4a3
Prss23
Tnks2
Tbc1d20
Sesn1
Adcy1
Evi2a
Itgb1
Ppp2r5b
Agap2

Naa20
Jph4
Stxbp1
Mt2
Atn1
Cadm4
Mtmr9
Ppp1r9b
Rnf5
Camk2a
Tef
Prkar1a
Kif5a
Rpl39
Hnrnpa2b1
Rusc2
Add3
Rgs7bp
Vxn
Lpcat4
Gja1
Sptbn1
Rab3c
Fam219a
Atp5g3
Syngr1
Sptan1
Gpr88
Nsf
Usp50
Comt
Rps27rt
Rab28
Atxn2
Spry2
Arhgap32
Senp3
Rpl37a
Sdk2
Cyth1
Kcnh1
Cpox
Idi1
Mrps28
Cpe
Ubb
Rpl23
Dynll1
Cox4i1
Rpl15
Uqcrq
Rpl27a
Rps16
Rps15
Rpl29
Eef1a1
Rps15a
Cst3
Rps12

Uqcrh
Pfdn5
Rpl35a
Rpsa
Rpl30
Rps11
Rps3a1
Tpt1
Fis1
Rpl8
Rps5
Rps13
Rpl18
Rpl18a
Rpl4
Rpl17
Ttc9b
Hba-a1
Rps2
Rpl7
Rps3
Ndufa13
Rpl26
Rpl5
Rpl9
Gapdh
Ndufb8
Nme2
Rps4x
Rpl24
Rpl13a
Cryab
Fau
Rpl14
Myl4
Rpl11
Ndufb9
Rpl21
Rps20
Rpl13
Rps8
Hbb-bs
Rpl6
Rps7
Rps27a
Rps24
Rpl10

SS iTBS L2/3

DEGs Young sham vs Aged sham	iTBS DEGs unique to young adults	iTBS DEGs unique to young adults that are also ageing DEGs
Gapdh	Gm10076	
Uba52	Rpl35	
Rpl4	Pcp4	
Malat1	Gstp1	
Nme7	Tmsb10	
Psd	Rps27	
Calm1	Camk2n1	
Cox8a	Aldoc	
Ndufa4	Ddit4l	
Actb	Gm2000	
Dpysl2	Ppme1	
Ost4	Kcnj10	
Lrrc17	Glul	
Slc1a2		
Snhg6		
Bc1		
Lars2		
Pgam1		
Rps2		
Penk		
Eef1a1		
Ddn		
Rpl9-ps6		
Rpl14		
Mt1		
Pfn1		
Rps28		
Rps24		
Hsp90ab1		
Comt		
Ndufa1		
Txn1		
Ppp2r1a		
Arl8a		
Rps27a		
Eef1a2		
Rpl5		
Rpl6		
Fkbp8		
Usp50		
Tspyl4		
Gnb2		
Rps8		
Rps29		
Ddx5		
Tubb3		
Ewsr1		
Mical2		
Dlg4		

Cab39
Gpr26
Stub1
Rpl3
BC005537
Arc
Rps15a
Maged1
Ppp3r1
Nap1l5
Cinp
Rgs20
Lmtk2
A830009L08Rik
Ptma
Atox1
Cox7b
Ctxn1
Junb
Pfdn5
Ptk2b

SS cTBS L2/3

DEGs Young sham vs Aged sham	cTBS DEGs unique to young adults	cTBS DEGs unique to young adults that are also ageing DEGs
Gapdh	Lrrc17	Cinp
Uba52	Basp1	Comt
Rpl4	Rps28	Cox8a
Malat1	Tspyl4	Ddn
Nme7	Snap25	Eef1a1
Psd	Snca	Ewsr1
Calm1	Dmtn	Fkbp8
Cox8a	Rps29	Gapdh
Ndufa4	Cox17	Gpr26
Actb	Ndufa1	Lmtk2
Dpysl2	Nrn1	Lrrc17
Ost4	Tomm7	Ndufa1
Lrrc17	Isca1	Ost4
Slc1a2	Tatdn1	Pfdn5
Snhg6	Snrpf	Ppp2r1a
Bc1	Pacsin1	Rpl14
Lars2	Actr2	Rpl4
Pgam1	Arpp21	Rpl6
Rps2	Araf	Rpl9-ps6
Penk	Atp5md	Rps2
Eef1a1	Rps27	Rps24
Ddn	Sdhd	Rps27a
Rpl9-ps6	Ost4	Rps28
Rpl14	Atp6v1a	Rps29
Mt1	Sem1	Rps8
Pfn1	Cdk5r2	Snhg6
Rps28	Atp1a3	Tspyl4
Rps24	Cox7c	Txn1
Hsp90ab1	Snhg9	Usp50
Comt	Snhg6	
Ndufa1	Eif4a2	
Txn1	Slc6a1	
Ppp2r1a	Spag9	
Arl8a	App	
Rps27a	Slc25a22	
Eef1a2	Snhg8	
Rpl5	Schip1	
Rpl6	Ddn	
Fkbp8	Rgs7bp	
Usp50	Comt	
Tspyl4	Txn1	
Gnb2	Ndrg4	
Rps8	Smim26	
Rps29	Rnf5	
Ddx5	Zwint	
Tubb3	Agap2	
Ewsr1	Naa20	
Mical2	Fam131b	
Dlg4	Stxbp1	

Cab39
Gpr26
Stub1
Rpl3
BC005537
Arc
Rps15a
Maged1
Ppp3r1
Nap1l5
Cinp
Rgs20
Lmtk2
A830009L08Rik
Ptma
Atox1
Cox7b
Ctxn1
Junb
Pfdn5
Ptk2b

Bloc1s1
Wsb2
Gucy1b1
Sema7a
Cops7a
Selenof
Acyp2
Cdk17
Rheb
Huwe1
Srsf5
Cmpk1
Lpcat4
1810037I17Rik
Pam
Usp50
Ndufa3
Mrpl33
Rpl39
Lmtk2
Fus
Lmo4
B2m
Arhgap39
Hmgb1
Psip1
Atn1
Psd3
Kcna2
Glul
Atp6v1h
Ndufb3
Hnrnpa2b1
Rab28
Unc13a
Snhg3
Prrc2a
Ank2
Fgfr1op2
Camk2a
Ewsr1
Hprt
Kctd13
Ubqln2
Slc4a10
Qk
Ndfip2
Zfp365
Prkar1a
Phyhipl
Brms1l
Oxr1

Cntnap1
Mgat3
Paip2
Ppp1r16b
Tmem258
Numb
Cd302
Serinc3
Atp5mpl
Shank1
Dnajc6
Megf9
Atp8a1
Btbd10
R3hdm4
Phpt1
Azin1
Unc5a
Gng13
Mapre2
Fbxl16
Herc3
Hlf
Cnksr2
Rpl37a
Capza2
Rab1a
Pde2a
Gas7
Golph3
Gria2
Rcn2
Mt2
Fam131a
Tmed7
Spry2
Cinp
Gja1
Chd9
Ank
Ptprz1
Vps29
Sec14l1
Anks1b
Ppm1h
Atp6ap2
Sft2d1
Gabra4
Ids
Dnajb5
Auts2
Bccip

Slc12a5
Kalrn
Gas5
Abhd8
Vxn
Btf3l4
Senp3
Slc4a4
Arhgap32
Wdr89
Rpl9-ps6
Stk25
Irf2bpl
Fez1
Rsrp1
Arhgef12
Mpv17
Cacng7
Lzts3
Rab3c
Scn1a
Rps27rt
Ogdhl
Arpp19
Hopx
Nedd4
Tma7
Ppp2cb
Dusp7
Plxna2
Sptbn2
Ccn3
Abhd3
Ssr3
Rap2a
Atxn2
Cox8a
Kitl
Tmx3
Car2
Tmem184b
Ncoa2
Gpr26
Deaf1
Saxo2
Dnajb4
Pmpcb
Atp5k
Ssbp1
Rai1
Nme3
Coa6

Cnnm1
Vstm2b
Atp8b2
Syngap1
Dmtf1
B230334C09Rik
Mrps28
Gtpbp2
Kif22
Vps50
Cyth1
Rps16
Hspa8
Rpl27a
Edf1
Nrsn1
Cst3
Tubb5
Mrfap1
Rps15
Fkbp8
Ndufb10
Clstn1
Nme2
Pkm
Saraf
Rps5
Ppp3ca
Rps11
Ctsd
Flywch1
Rpl30
Chgb
Ndufb8
Syp
Smdt1
Ppp2r1a
Rps12
Eef1a1
Rpl28
Pink1
Ubb
Rpl9
Rpl8
Rps10
Ndufa13
Eif3k
Psap
Rpl6
Rpl29
Nenf
Rps3a1

Rpl26
Fkbp2
Rps3
Rpsa
Rpl11
Rpl18a
Ap2b1
Pebp1
Pfdn5
Rps20
Rps7
Hbb-bs
Rps2
Rpl13a
Rpl13
Rpl24
Rpl14
Fau
Ndufb9
Rpl4
Eno1
Gapdh
Rpl21
Nefm
Rpl10
Rps27a
Rps8
Rps24

SS iTBS L4

DEGs Young sham vs Aged sham	iTBS DEGs unique to young adults	iTBS DEGs unique to young adults that are also ageing DEGs
Gapdh	Rps27	Rps27
Uba52	Pcp4	Rps29
Rpl4	Tusc3	
Camk2n1	Rps29	
Rps29	Rpl10	
Malat1	Tmsb10	
Nme7	Tmsb4x	
Rps28	Atp5md	
Lrrc17	Ppia	
Lmtk2		
Dpysl2		
Rpl35		
Ost4		
Sema7a		
Gpr26		
Bex2		
Lsm8		
Ndufa4		
Cacna1g		
Rgs4		
Tspyl4		
Plcxd2		
Psd		
Snhg6		
Rpl9-ps6		
Mical2		
Maged1		
Kcnab3		
Sel1l3		
Itm2c		
Rps27rt		
Dab2ip		
Rraga		
Banp		
Schip1		
Bbs4		
Pkm		
Actg1		
Mcee		
Txn1		
Cd302		
Scn1a		
Mtpn		
Syndig1		
Medag		
Rps27		
Car7		
Gstp1		
Mtch1		

Calb1
Nrn1
Akt3
Grm2
Reep2
Ptpn5
Vcp
Cinp
Sdhd
Plec
Mbp
Ly6e
Gm10076
Krt10
Slc17a6
Ptpn4
Stx1a
Fam241b
Vegfa
Actr2
Rps2
Gaa
Stmn3
Lrrc57
Lrp11
Fam107a
Pantr1
Caprin1
Rabggta
Prkaca
Dynll2
Cnot2
Kcnj10
Dact2
Rnf181
Zfr
Pdia3
Dcdc2a
Serinc3
0610010K14Rik
Unc50
Tubb5
Stradb
Kansl1
Tln1
Bag5
Rims1
Inpp5a
Nisch
Usp50
Isca1
Slc25a23

SS cTBS L4

DEGs Young sham vs Aged sham	cTBS DEGs unique to young adults	cTBS DEGs unique to young adults that are also ageing DEGs
Gapdh	Uba52	Actr2
Uba52	Lrrc17	Bbs4
Rpl4	Tspyl4	Cacna1g
Camk2n1	Rps28	Cd302
Rps29	Basp1	Dab2ip
Malat1	Nrn1	Gapdh
Nme7	Rps29	Gpr26
Rps28	Isca1	Isca1
Lrrc17	Araf	Kansl1
Lmtk2	Snap25	Lmtk2
Dpysl2	Snca	Lrrc17
Rpl35	Actr2	Lrrc57
Ost4	Rps27	Lsm8
Sema7a	Tomm7	Nrn1
Gpr26	Cox17	Ost4
Bex2	Slc6a1	Pkm
Lsm8	Sdhd	Rpl4
Ndufa4	Ost4	Rpl9-ps6
Cacna1g	Ndufa1	Rps2
Rgs4	Sem1	Rps27
Tspyl4	Spag9	Rps27rt
Plcxd2	Snrpf	Rps28
Psd	Cops7a	Rps29
Snhg6	Pacsin1	Rraga
Rpl9-ps6	Arpp21	Schip1
Mical2	Snhg9	Scn1a
Maged1	1810037I17Rik	Sdhd
Kcnab3	Lmo4	Sema7a
Sel1l3	Dmtn	Snhg6
Itm2c	Kcna2	Stmn3
Rps27rt	Phpt1	Tspyl4
Dab2ip	Snhg6	Txn1
Rraga	Snhg8	Uba52
Banp	Ank	Unc50
Schip1	Selenof	Usp50
Bbs4	Atp1a3	Vegfa
Pkm	Lmtk2	Zfr
Actg1	Eif4a2	
Mcee	App	
Txn1	Paip2	
Cd302	Ppp1r16b	
Scn1a	Sema7a	
Mtpn	Rcn2	
Syndig1	Mgat3	
Medag	Ddn	
Rps27	Tatdn1	
Car7	Cox7c	
Gstp1	Atp6v1a	
Mtch1	Srsf5	

Calb1	Naa20
Nrn1	Gja1
Akt3	Car2
Grm2	Atp5md
Reep2	Bloc1s1
Ptpn5	Schip1
Vcp	Smim26
Cinp	Rbis
Sdhd	Klf9
Plec	Rnf5
Mbp	Huwe1
Ly6e	Txn1
Gm10076	Mat2b
Krt10	Ndrg4
Slc17a6	Ewsr1
Ptpn4	Cd302
Stx1a	Ank2
Fam241b	Kif3c
Vegfa	Cacna2d1
Actr2	Oxr1
Rps2	Atp6v1h
Gaa	Inpp4a
Stmn3	Zwint
Lrrc57	Slc25a22
Lrp11	Rnf7
Fam107a	Rgs7bp
Pantr1	Cdk5r2
Caprin1	Smim10l1
Rabggta	Nrxn1
Prkaca	Scg2
Dynll2	Ndfip2
Cnot2	Ndufa3
Kcnj10	Scn1a
Dact2	Arpc2
Rnf181	Gria2
Zfr	Slc4a10
Pdia3	Vps29
Dcdc2a	Zfr
Serinc3	Comt
0610010K14Rik	Ids
Unc50	Rap1gds1
Tubb5	Unc5a
Stradb	Fus
Kansl1	Nedd4
Tln1	Rusc2
Bag5	Dbndd2
Rims1	Gpr26
Inpp5a	Fmc1
Nisch	Camk2a
Usp50	Ttc7b
Isca1	Cntnap1
Slc25a23	Apba1

Sdcbp
Glul
Rpl39
Herc3
F3
Tmem258
Rheb
Rbfox3
Zfp365
Capza2
Eif5
Dpm3
Csnk1a1
Cs
Trappc6b
Tafa2
Rpl9-ps6
Phactr1
Acyp2
Hectd4
Hypk
Shank1
Csnk2a2
Atf2
Snn
Tsc22d4
Lsm8
Lrrc57
Ndufb3
Uba3
Cacna1g
Iscu
Ppp1r2
Slc12a5
Prrc2b
Hopx
Mapre2
Pet100
Dusp6
Rraga
Mapk1
Wsb2
Unc50
Fbxo34
Dab2ip
Usp50
Lpcat4
Foxk2
Senp3
Sgip1
Cystm1
Fbxl16

Cdk17
Gnai1
Cacng7
Rapgef4
Vegfa
Vstm2b
Trappc2
Atn1
Atxn2
Impact
Gas7
Fam120a
Bbs4
Brms1l
Car11
Kansl1
Chd9
Mir124a-1hg
Cds2
Hpcal4
Slc4a4
Rps27rt
Rpl37a
Arpp19
Idi1
Samd8
Btf3l4
Tceal1
Myrip
Mrpl3
Acat1
Med30
Churc1
Atxn2l
Hibadh
Klf6
Cox8a
Syt1
Gtpbp2
Ccnh
C130071C03Rik
Myadm
Clcn2
Atp5k
Acap2
Shd
Agk
Dusp7
Cspp1
Kif22
Fam98b
Cpeb2

Adam9
Taf1d
Cep104
Tac1
Eef1a1
Selenow
Rpl32
Psap
Rpl26
Stmn3
Rpl9
Cox4i1
Rpl30
Rps3
Tpt1
Cst3
Syp
Rps3a1
Rpl31
Hspa8
Tuba4a
Rpl17
Rpl18
Rps15
Rps10
Bsg
Aplp1
Atp5d
Rpl10
Rpl6
Pebp1
Rps7
Rpl28
Eef1b2
Rps11
Rps2
Rpl8
Ubb
Rpl11
Rpl13a
Ndufa13
Fkbp2
Pfdn5
Pkm
Rpl24
Rpsa
Nenf
Rpl18a
Chgb
Rpl4
Ndufb9
Rpl13

Rpl21
Eno1
Fau
Rps20
Rps8
Gapdh
Rpl14
Rps24
Rps27a

SS iTBS L5

DEGs Young sham vs Aged sham	iTBS DEGs unique to young adults	iTBS DEGs unique to young adults that are also ageing DEGs
Gapdh	Penk	Penk
Uba52	Eno1	
Nme7	Rps27	
Rps20	Tusc3	
Rpl4	Gpr88	
Plcxd2	Tac1	
Ndufa4	Gas5	
Lrrc17	Tmsb4x	
Rps24	Gap43	
Snhg6	H3f3a	
Lmtk2	Rpl17	
Fmnl1	Nr1d1	
Fth1	Tecr	
Zwint	Tatdn1	
Malat1	Nop53	
Ost4	Gm2000	
Fau	Bc1	
Rpl21		
Gpr26		
Itm2c		
Rps27a		
Rps2		
Pak1		
Reep1		
Ly6e		
Aldoc		
Panx2		
Dab2ip		
Rps28		
Satb1		
Rpl9		
Rtn1		
Mical2		
Rtn4r		
Ifngr2		
Usp50		
Vapa		
Eef1a1		
St3gal3		
Nrep		
Ptma		
Rps8		
Fxyd7		
Fam107a		
Sst		
Kcnab3		
Sema7a		
Shisa4		
Mef2c		

Oaz1
Prkaca
Atp1a3
Vsnl1
Atp6v1g2
Stx1a
Pfdn5
Homer1
Dock4
Napb
Arhgap32
Nlgn2
Dbi
Plxnd1
Atp2a2
Pacsin1
Scn1a
Rps4x
Syt1
Pip5k1c
Neurod6
Coro2b
Cinp
Schip1
Adcy1
Ccdc136
Btbd3
Slc6a7
Prdx1
Mir124a-1hg
Maz
Zfp938
Txn1
Tspyl4
Rpl5
Rpl14
Rps7
Sncb
Gnai1
Actb
Syn1
Ttll11
Lrp11
Chgb
Sdhd
Ppp6r1
Rpl18a
Nrip3
Sptbn2
Dkkl1
Atf4
Atp6v1a

Dok6
Rab6b
Cox7b
Nudt4
Shank1
Neurod2
Nlk
Sel1l3
Myo5a
Syndig1
Aldh1l1
Rps10
Stxbp1
Arc
Prrt1
Tmem145
Agap2
Rps29
Adora1
Kcnj10
Galnt9
Atg13
Atxn7l3
Dnajb2
Map6
Camk2n1
Nrn1
Igsf9b
Rph3a
Tln1
Dclk1
Cx3cl1
Slc30a3
R3hdm1
Eif3c
Psmg4
Cnih1
Arap2
Whrn
Hsd11b1
Ndfip1
Slc25a5
Opcml
Ppm1b
Atp8a1
Jdp2
Rpl22
Ablim1
Gtdc1
Eif4a2
Nrbp2
Bmpr2

Eif5a2
Ulk2
Dnm1
Penk
Esd
Gfod1
Mb21d2
Sv2b
Ptprd
Gpr158
Ephb6
Ppp1r13b
Porcn
Ptprk
Gria4
Myrip
Usp15
Car7
Pafah1b1
Kifc2
Nmt2
Dpysl4
Mpp3
Rgs4
Cpne9
Morf4l1
Tafa2
Atxn2
Luzp1
Syngr1
Ids
Comt
Mrpl55
Myl4
Rgs17
Sfxn3
Mpi
Tmx3
Senp2
Rubcn
Fgf13
Cacnb1
Chmp4b
Ddn
Camk2n2
Psd
Adnp
Ypel4
Nptxr
Shfl
Arf2
Rabl6

Arhgap20
Rae1
Lingo1
Ncald
Uba3
Tmbim6
Mfge8
Reep2
Hlf
Shank2
Lrpprc
Gm20300
Clip2
Ddost
Plekhj1
Il34
Rab3gap1
Pamr1
Hgs
Stac2
Hspa5
Arhgap44
Hacd3
Brsk2
Eipr1
Ttl
Extl2
Casc3
Rpl24
Gfra4
Slc35a4
Unc50
Arpc4
Tmem151a
Ube2ql1
Mcf2l
Asph
Prrc2a
Nsf
Snrpd2
Ncan
Naa60
Fkbp1b
Atxn1
Dnajb14
Twf2
Ankrd24
Cnbp
Chd9
Dpysl2
Ptprn
Pdhx

Brinp1
D430041D05Rik
Zdhhc2
Arxes2
Vps9d1
Ttc4
Sez6l
Rims4
Gbf1
Hecw1
Mapk8
Kif5c
Zranb2
Higd2a

SS cTBS L5

DEGs Young sham vs Aged sham	cTBS DEGs unique to young adults	cTBS DEGs unique to young adults that are also ageing DEGs
Gapdh	Lrrc17	Agap2
Uba52	Basp1	Arhgap32
Nme7	Tspyl4	Arpc4
Rps20	Rps28	Atp1a3
Rpl4	Snca	Atp6v1a
Plcxd2	Snap25	Atxn1
Ndufa4	Pacsin1	Atxn2
Lrrc17	Araf	Chd9
Rps24	Nrn1	Cinp
Snhg6	Ndufa1	Comt
Lmtk2	Ost4	Dab2ip
Fmnl1	Rps29	Ddn
Fth1	Dmtn	Dnm1
Zwint	Atp6v1a	Dok6
Malat1	Tomm7	Eif4a2
Ost4	Rps27	Fau
Fau	Isca1	Gapdh
Rpl21	Sdhd	Gnai1
Gpr26	Cox17	Gpr26
Itm2c	App	Higd2a
Rps27a	Slc6a1	Ifngr2
Rps2	Slc25a22	Kifc2
Pak1	Actr2	Lmtk2
Reep1	Snrpf	Lrrc17
Ly6e	Zwint	Myo5a
Aldoc	Cops7a	Nrn1
Panx2	Smim26	Ost4
Dab2ip	Hprt	Pacsin1
Rps28	Snhg6	Pfdn5
Satb1	Naa20	Pip5k1c
Rpl9	Atp1a3	Prrc2a
Rtn1	Wsb2	Reep1
Mical2	Snhg9	Rpl14
Rtn4r	Atp5md	Rpl18a
Ifngr2	Stxbp1	Rpl21
Usp50	Cox7c	Rpl24
Vapa	Sem1	Rpl4
Eef1a1	Arpp21	Rpl9
St3gal3	Ddn	Rps10
Nrep	Spag9	Rps2
Ptma	Dnajc6	Rps20
Rps8	Glul	Rps24
Fxyd7	Rap1gds1	Rps27a
Fam107a	Rapgef4	Rps28
Sst	Eif4a2	Rps29
Kcnab3	Cdk5r2	Rps4x
Sema7a	Rgs7bp	Rps7
Shisa4	Rnf5	Rps8
Mef2c	Schip1	Schip1

Oaz1
Prkaca
Atp1a3
Vsnl1
Atp6v1g2
Stx1a
Pfdn5
Homer1
Dock4
Napb
Arhgap32
Nlgn2
Dbi
Plxnd1
Atp2a2
Pacsin1
Scn1a
Rps4x
Syt1
Pip5k1c
Neurod6
Coro2b
Cinp
Schip1
Adcy1
Ccdc136
Btbd3
Slc6a7
Prdx1
Mir124a-1hg
Maz
Zfp938
Txn1
Tspyl4
Rpl5
Rpl14
Rps7
Sncb
Gnai1
Actb
Syn1
Ttll11
Lrp11
Chgb
Sdhd
Ppp6r1
Rpl18a
Nrip3
Sptbn2
Dkkl1
Atf4
Atp6v1a
Kcna2
Tatdn1
Ndrg4
Car2
1810037I17Rik
Frrs1l
Lmtk2
Phpt1
Hpcal4
Txn1
Srsf5
Snhg8
Fus
Klc2
Ank2
Rab3c
Bloc1s1
Brms1l
Cntnap1
Agap2
Ndfip2
Rheb
Add2
Huwe1
Tef
Slc4a10
Cadm4
Camk2a
Fam131a
Mgat3
Rcn2
Prrc2a
Kctd13
Psd3
Lmo4
Syngr1
Herc3
Kif3c
Zfp365
Smim10l1
Ppp1r2
Usp50
Tmbim6
Ndufa3
Scn1a
Chd9
Nedd4
Phyhipl
Acyp2
Erc2
Sptbn2
Selenof
Scn1a
Sdhd
Shank1
Snhg6
Sptbn2
Stxbp1
Syngr1
Tmbim6
Tspyl4
Txn1
Usp50
Zwint

Dok6	Fam131b
Rab6b	Chmp5
Cox7b	Tsc22d4
Nudt4	Atp6v1h
Shank1	Atp5mpl
Neurod2	Oxr1
Nlk	Zfr
Sel1l3	Hectd4
Myo5a	Atn1
Syndig1	Atp1b2
Aldh1l1	Paip2
Rps10	Csnk1e
Stxbp1	R3hdm4
Arc	Lpcat4
Prrt1	Gng13
Tmem145	Rpl39
Agap2	Kif5a
Rps29	Ppp1r16b
Adora1	Shank1
Kcnj10	Atxn2l
Galnt9	Rnf157
Atg13	Smim14
Atxn7l3	Gnai1
Dnajb2	Tmem258
Map6	Ewsr1
Camk2n1	Ogdhl
Nrn1	Rpl37a
Igsf9b	Hypk
Rph3a	Ifngr2
Tln1	Sec61g
Dclk1	Scn2a
Cx3cl1	F3
Slc30a3	Psip1
R3hdm1	Luzp2
Eif3c	Cyth1
Psmg4	Ppp2r2c
Cnih1	Cinp
Arap2	Fbxl16
Whrn	Prkar1a
Hsd11b1	Unc5a
Ndfip1	Apba1
Slc25a5	Ubqln2
Opcml	Trappc2
Ppm1b	B2m
Atp8a1	Arpc4
Jdp2	Sft2d1
Rpl22	Phactr1
Ablim1	Gja1
Gtdc1	Emc7
Eif4a2	Jph4
Nrbp2	Pet100
Bmpr2	Rps27rt

Eif5a2	Tppp
Ulk2	Megf9
Dnm1	Arrb1
Penk	Psma5
Esd	Slc12a5
Gfod1	Gas7
Mb21d2	Rian
Sv2b	Gm14305
Ptprd	Bcl2l2
Gpr158	Dnajb5
Ephb6	Tmed7
Ppp1r13b	Atxn2
Porcn	Azin1
Ptprk	Reep1
Gria4	Scn4b
Myrip	Sucla2
Usp15	Rsrp1
Car7	Erh
Pafah1b1	Idi1
Kifc2	Kifc2
Nmt2	Capza2
Dpysl4	Ank
Mpp3	Mapk1
Rgs4	Gpr26
Cpne9	Scg2
Morf4l1	Mrps28
Tafa2	Atxn1
Atxn2	Sod2
Luzp1	Peg3
Syngr1	Necap1
Ids	Higd2a
Comt	Gria2
Mrpl55	Insyn1
Myl4	Kcnh1
Rgs17	Arhgap32
Sfxn3	Senp3
Mpi	Comt
Tmx3	Cd302
Senp2	Rpl9-ps6
Rubcn	Tmem59
Fgf13	Rora
Cacnb1	Sparcl1
Chmp4b	Plppr4
Ddn	Ptprz1
Camk2n2	Sgip1
Psd	Rusc2
Adnp	Gas5
Ypel4	Tiprl
Nptxr	Lrrc4c
Shfl	Pip5k1c
Arf2	Dab2ip
Rabl6	Dnajb4

Arhgap20	Vps29
Rae1	Cystm1
Lingo1	Arpc2
Ncald	Rap2a
Uba3	Abhd8
Tmbim6	Tac1
Mfge8	Slc4a4
Reep2	Dnm1
Hlf	Necab2
Shank2	Aftph
Lrpprc	Gnao1
Gm20300	Fez1
Clip2	Map2k1
Ddost	Fmc1
Plekhj1	Dynlt3
Il34	Trrap
Rab3gap1	Frmpd4
Pamr1	Qk
Hgs	Hnrnpk
Stac2	Nme3
Hspa5	Vma21
Arhgap44	Dok6
Hacd3	Ppip5k1
Brsk2	Fjx1
Eipr1	Btf3l4
Ttl	Snn
Extl2	Anks1b
Casc3	Myo5a
Rpl24	Wdr89
Gfra4	Rps16
Slc35a4	Rplp1
Unc50	Rps19
Arpc4	Rps3a1
Tmem151a	Pebp1
Ube2ql1	Rpl36al
Mcf2l	Rpl27a
Asph	Pkm
Prrc2a	Ctsd
Nsf	Ndufb11
Snrpd2	Rrp1
Ncan	Rpl6
Naa60	Ppp2r1a
Fkbp1b	Rpl7
Atxn1	Rpsa
Dnajb14	Rps15
Twf2	Tubb3
Ankrd24	Calr
Cnbp	Rpl23
Chd9	Eef1b2
Dpysl2	Ndufb8
Ptprn	Tpt1
Pdhx	Smdt1

Brinp1	Rpl30
D430041D05Rik	Atp5d
Zdhhc2	Rpl28
Arxes2	Rps11
Vps9d1	Rps12
Ttc4	Syp
Sez6l	Ndufa13
Rims4	Fkbp2
Gbf1	Rpl11
Hecw1	Rpl4
Mapk8	Rpl9
Kif5c	Tmem160
Zranb2	Eno1
Higd2a	Rps4x
	Ttr
	Fkbp8
	Rpl8
	Edf1
	Rpl26
	Gapdh
	Rpl13
	Rps7
	Rps2
	Ndufb9
	Ndufb10
	Rpl21
	Rpl18a
	Rpl14
	Rps20
	Rps10
	Pfdn5
	Rpl24
	Rps8
	Fau
	Rpl13a
	Rpl10
	Pvalb
	Rps27a
	Rps24
	Hbb-bs

SS iTBS L6

DEGs Young sham vs Aged sham	iTBS DEGs unique to young adults	iTBS DEGs unique to young adults that are also ageing DEGs
Gapdh	Rps29	Gapdh
Uba52	Rps27	Gas5
Pcp4	Gas5	Gstp1
Mbp	Rps28	Pcp4
Lrrc17	Pcp4	Penk
Cox8a	Rpl37a	Rpl35
Mobp	Tmsb4x	Rps27
Snhg6	Rpl37a	Rps28
Rpl4	Ftl1	Rps29
Rps28	Penk	Sem1
Rps29	Tma7	Tmsb4x
Tmsb4x	Dbi	
Psd	Tac1	
Ost4	Rpl22l1	
Pkm	Atp5md	
Klf9	Ndufb3	
Rpl21	Rpl39	
Tatdn1	Gpr88	
Slc1a2	Sem1	
Kif5a	Rps15a	
Bcas1	Mrpl42	
Eef1a1	Tmsb10	
Cox7b	Tuba1a	
Usp50	Igfbp6	
Clic4	Rpusd1	
Lpcat4	Lynx1	
Dpysl2	Myl4	
Dusp6	Gapdh	
Ndfip2	Cplx2	
Ndufa1	Apba1	
Chd9	Tubb4a	
Rps24	Cdk5r2	
Arc	Klc2	
Isca1		
Mical2		
Twf2		
Ighm		
Polr3e		
Txn1		
Cldn11		
Fau		
Pfdn5		
Atraid		
Mal		
Gadd45a		
Hivep2		
Rps27		
Comt		
Actg1		

Rpl14
Schip1
Cspg5
Agap2
Smim26
Ptp4a3
Ctxn1
Gm10076
Shisa4
Gstp1
Tubb5
Tspyl4
Mrpl27
Rps2
Malat1
Hs3st2
Dlg2
Ccm2
Sema7a
Rpl37a
Sgsm1
Bloc1s1
Ccdc12
6330403K07Rik
Eef1akmt1
Stmn3
Nbea
Ina
Atxn7l3b
Exoc6
Ddn
Tsc22d3
Arhgap33
Eif3c
Eef1a2
Unc50
Chn2
Zfp365
Slc4a10
Acap3
Actb
Lrp11
Uba3
Pacsin1
Epas1
Nae1
S1pr1
Rasl10a
Coa6
Srcin1
Snhg9
Jagn1

Syt6
Fgf13
Tmbim4
Ly6e
Hpcal4
Golga7
Grb14
Fxyd7
Homer1
Arhgef7
Ncald
Immp1l
Ube2j1
Vxn
Bex2
Lsm8
Fut9
Fmnl1
Tmem151b
Tomm7
Kif5c
Trp53inp2
Nsg2
Ntsr2
Acyp2
Slirp
Snrpf
Kcnj10
Rims2
B3galt2
Tmem167
Rpl5
Gnai1
Sdr39u1
Prrc2a
Mog
Slc8a1
Emc3
Grsf1
Cplx3
Fyn
Ddah1
Phldb1
Gas5
Cfap36
Mrpl35
Kcnip4
Fkbp8
Rmnd5a
Epn2
Nxf1
Ttc9b

Wdr13
Cdc123
Cd200
Cdc37l1
Usp31
Rab15
Actl6b
Tnfrsf21
Abhd16a
Eif4a2
Tbc1d9b
Polr2f
Crmp1
Polr2i
Prkaca
Cops7a
Reep1
Sdhd
Plxna2
Sipa1l1
Rnmt
Sdhaf2
Lamtor3
Hipk3
Ak1
Tspan2
Chrm3
Tmeff2
Trak2
Rap1gap2
Rnasek
Maged1
Mtx1
Kcnh7
Itm2c
Vps50
Zfand2a
Nr1h2
Rraga
Cox16
Rpl35
Dnajb4
Ube2w
Necab3
Snhg8
Abhd17a
Golph3
Igsf21
Tmem135
Psip1
Zfp445
Ppp2r5a

Ugp2
Slc32a1
Cox20
Zfas1
Thra
Ermn
Sem1
Elmo2
Ntrk3
Slc2a1
Mfsd6
Wnk1
Pcna
Btbd1
Aopep
Zdhhc17
Tmem208
Abracl
Basp1
Nisch
Snhg12
Brwd1
Setd7
Rps8
9130401M01Rik
Ccnh
Plpbp
Naa20
Nsa2
Pnpla8
Pdlim2
Ubl7
Gfod1
Plppr4
Kcnc4
Tfg
Usp32
Limk1
R3hdm4
Rer1
Rbm3
Pomgnt1
Slc12a2
Trbc2
Taf13
Serpinb6a
Fez2
Pitpnc1
Adora1
Ndufv1
Vcp
Large1

Rala
Epb41l1
Clec2l
Pou3f3
Pdpk1
Cisd3
Tmem126a
Penk
Gjb6
Necab1
Frrs1l
Maz
Igsf9b
Paqr4
AI593442
Commd6
Otud5
Spop
Fa2h
Abce1
Arhgef12
Elof1
Trpc4ap
Dok6
Bcap31
Mpv17
Tmem50b
Snrpc
Clns1a
Tubb3
Slmap
Wdr47
Rprd1a
Shank2
Map4k3
A830018L16Rik
Vstm2l
Pltp
Ptprz1
Ube3a
Pdcd10
Tsg101
Ubl4a
Ly6c1
Snapc5
St8sia5
Tln1
Cckbr
Becn1
Panx2
Kcnc1
Acsl6

Src
Fxyd1
Neurod6
Mxi1
Arhgap23
Carm1
Nme7
Zmynd8
Rab5if
Anp32b
Rps27l
Mgat4b
Asrgl1
Etv5
Arxes2
Rab28
Rida
Mir124a-1hg
Clstn2
Trim28
Srsf10
Dop1b
Dctn4
Gpd1l
Tnk2
Rcn2
Pik3r3
Slc27a4
Med10
Rab3gap1
Dagla
Manf
Zyx
Enpp2
BC004004
Amz2
Gaa
Uba2
Tacc1
B3gat1
Slc25a23
Eid2
Ppib
Scarb2
Supt6
Gpd1
Dtnb
Arl6ip1
Mxd4
Negr1
Pcmtd2
Emc2

Slc25a18
Higd2a
Hdhd2
Chka
Sprn
Cryab
Prpf8
Slc8a2
Atp2c1
Speg
Arfgef1
Cstb
Klf13
Atg4b
Acyp1
Ddx1

SS cTBS L6

DEGs Young sham vs Aged sham	cTBS DEGs unique to young adults	cTBS DEGs unique to young adults that are also ageing DEGs
Gapdh	Uba52	Acyp2
Uba52	Lrrc17	Agap2
Pcp4	Rps28	Basp1
Mbp	Basp1	Bloc1s1
Lrrc17	Tspyl4	Chd9
Cox8a	Rps29	Coa6
Mobp	Cox17	Comt
Snhg6	Ndufa1	Cops7a
Rpl4	Tomm7	Cox8a
Rps28	Snap25	Cryab
Rps29	Araf	Ddn
Tmsb4x	Rps27	Dnajb4
Psd	Pacsin1	Eef1a1
Ost4	Snca	Eif4a2
Pkm	Snrpf	Fau
Klf9	Isca1	Fkbp8
Rpl21	Slc6a1	Gapdh
Tatdn1	Arpp21	Gas5
Slc1a2	Sem1	Hpcal4
Kif5a	Ost4	Isca1
Bcas1	Car2	Itm2c
Eef1a1	Rapgef4	Kif5a
Cox7b	App	Kif5c
Usp50	Snhg9	Klf9
Clic4	Tatdn1	Lpcat4
Lpcat4	Ndrg4	Lrp11
Dpysl2	Atp6v1a	Lrrc17
Dusp6	Nrn1	Lsm8
Ndfip2	Glul	Mbp
Ndufa1	Txn1	Naa20
Chd9	Smim26	Nae1
Rps24	Atp1a3	Ndfip2
Arc	Dmtn	Ndufa1
Isca1	Actr2	Ost4
Mical2	Eif4a2	Pacsin1
Twf2	Ddn	Pfdn5
Ighm	Srsf5	Pkm
Polr3e	Selenof	Plppr4
Txn1	Cops7a	Prrc2a
Cldn11	Cox7c	R3hdm4
Fau	Rgs7bp	Rab28
Pfdn5	Bloc1s1	Rcn2
Atraid	Naa20	Rpl14
Mal	Atp5md	Rpl21
Gadd45a	Ndfip2	Rpl37a
Hivep2	Slc25a22	Rpl4
Rps27	Wsb2	Rpl5
Comt	Snhg6	Rps2
Actg1	Camk2a	Rps24

Rpl14
Schip1
Cspg5
Agap2
Smim26
Ptp4a3
Ctxn1
Gm10076
Shisa4
Gstp1
Tubb5
Tspyl4
Mrpl27
Rps2
Malat1
Hs3st2
Dlg2
Ccm2
Sema7a
Rpl37a
Sgsm1
Bloc1s1
Ccdc12
6330403K07Rik
Eef1akmt1
Stmn3
Nbea
Ina
Atxn7l3b
Exoc6
Ddn
Tsc22d3
Arhgap33
Eif3c
Eef1a2
Unc50
Chn2
Zfp365
Slc4a10
Acap3
Actb
Lrp11
Uba3
Pacsin1
Epas1
Nae1
S1pr1
Rasl10a
Coa6
Srcin1
Snhg9
Jagn1

Phpt1
Zfp365
Rap1gds1
Rpl39
Rnf5
Sdhd
Agap2
Schip1
Zwint
Ppp1r2
Klf9
Cdk5r2
Slc4a10
Spag9
Ccn2
Kif5a
Fus
Snhg8
Lpcat4
Kctd13
Ndufa3
Rcn2
Nedd4
Fbxl16
Hprt
Gpm6b
Comt
Unc5a
Tsc22d3
Paip2
Ank2
Hmgb1
Vxn
Sipa1l1
Usp50
1810037I17Rik
Gria2
Chd9
Slc12a5
Ids
Sparcl1
Sptbn1
Ewsr1
Apc
R3hdm4
Acyp2
Ppp1r16b
Fam131b
Slc1a3
Gnao1
Plppr4
Gpr88

Rps27
Rps28
Rps29
Rps8
S1pr1
Schip1
Sdhd
Sem1
Sema7a
Sipa1l1
Slc4a10
Smim26
Snhg6
Snhg8
Snhg9
Snrpf
Tatdn1
Tomm7
Tsc22d3
Tspyl4
Ttc9b
Tubb5
Txn1
Uba52
Ugp2
Usp50
Vxn
Zfp365
Zfp445

Syt6
Fgf13
Tmbim4
Ly6e
Hpcal4
Golga7
Grb14
Fxyd7
Homer1
Arhgef7
Ncald
Immp1l
Ube2j1
Vxn
Bex2
Lsm8
Fut9
Fmnl1
Tmem151b
Tomm7
Kif5c
Trp53inp2
Nsg2
Ntsr2
Acyp2
Slirp
Snrpf
Kcnj10
Rims2
B3galt2
Tmem167
Rpl5
Gnai1
Sdr39u1
Prrc2a
Mog
Slc8a1
Emc3
Grsf1
Cplx3
Fyn
Ddah1
Phldb1
Gas5
Cfap36
Mrpl35
Kcnip4
Fkbp8
Rmnd5a
Epn2
Nxf1
Ttc9b
Scn4b
Stxbp1
Lrp11
Mapk1
Rpl37a
Ndufb3
Gad1
Cmpk1
Fez1
Gas5
Inpp4a
Romo1
Huwe1
Phyhipl
Rheb
Ppp2r2c
Tmem258
Cs
Cadm4
Arpc2
Rab3c
Gng13
Hnrnpa2b1
Atp1b2
Ttyh1
Ubqln2
Atp5mpl
Kcna2
Psma5
Nap1l2
Ctnnb1
Ppp1r1b
Selenop
Vps29
Dnajc6
Rusc2
Rps27rt
Sema7a
Rgs4
Sec61g
Ank
Rsrp1
Hpcal4
Tac1
Prkar1a
Kif5c
Atxn2
Lsm8
Abhd8
Impact
Idi1
Sod2

Wdr13	Rpl9-ps6
Cdc123	Tmem256
Cd200	Lmtk2
Cdc37l1	Mapre2
Usp31	Anks1b
Rab15	Ccnb1
Actl6b	Cox8a
Tnfrsf21	Herc3
Abhd16a	Bcl2l2
Eif4a2	Pet100
Tbc1d9b	Smim10l1
Polr2f	Mrpl33
Crmp1	Rasd2
Polr2i	Dnajb5
Prkaca	Oxr1
Cops7a	Gng7
Reep1	Ppp1r9b
Sdhd	Hectd4
Plxna2	Sgip1
Sipa1l1	Prrc2a
Rnmt	Coa6
Sdhaf2	Phactr1
Lamtor3	Ugp2
Hipk3	Zfp445
Ak1	Sst
Tspan2	Laptm4a
Chrm3	Enpp5
Tmeff2	Atn1
Trak2	F3
Rap1gap2	Camk2n1
Rnasek	Capza2
Maged1	Adcy5
Mtx1	Aco2
Kcnh7	Ppp2cb
Itm2c	Nptx2
Vps50	Slc4a4
Zfand2a	Ube2d1
Nr1h2	Rpl22l1
Rraga	Sft2d1
Cox16	Nae1
Rpl35	Morf4l1
Dnajb4	Cystm1
Ube2w	Rab28
Necab3	S1pr1
Snhg8	Dnajb4
Abhd17a	Med30
Golph3	Map2k1
Igsf21	Bpnt1
Tmem135	Mthfsl
Psip1	Senp3
Zfp445	Rpl10-ps3
Ppp2r5a	Mbp

Ugp2	Wdr89
Slc32a1	Mrps28
Cox20	Necab2
Zfas1	Nme3
Thra	Atxn2l
Ermn	Dync1h1
Sem1	Pcsk1n
Elmo2	Eif3k
Ntrk3	Rps14
Slc2a1	Ndufb8
Mfsd6	Atp5d
Wnk1	Ndufs6
Pcna	Tubb5
Btbd1	Cd81
Aopep	Ubb
Zdhhc17	Nrgn
Tmem208	Yars
Abracl	Rps16
Basp1	Rangap1
Nisch	Cst3
Snhg12	Eef1a1
Brwd1	Ndufb10
Setd7	Fkbp2
Rps8	Tpt1
9130401M01Rik	Pomp
Ccnh	Uchl1
Plpbp	Smdt1
Naa20	Ppp2r1a
Nsa2	Cryab
Pnpla8	Ndufb11
Pdlim2	Rpl18
Ubl7	Rpl30
Gfod1	Hspa8
Plppr4	Rplp1
Kcnc4	Tle5
Tfg	Rpl29
Usp32	Ctsd
Limk1	Rpl5
R3hdm4	Rpl17
Rer1	Rpl32
Rbm3	Clstn1
Pomgnt1	Rps3a1
Slc12a2	Gnaz
Trbc2	Atp9a
Taf13	Psap
Serpinb6a	Rps4x
Fez2	Rps12
Pitpnc1	Myl4
Adora1	Rpl7
Ndufv1	Chgb
Vcp	Rpl26
Large1	Cox4i1

Rala
Epb41l1
Clec2l
Pou3f3
Pdpk1
Cisd3
Tmem126a
Penk
Gjb6
Necab1
Frrs1l
Maz
Igsf9b
Paqr4
AI593442
Commd6
Otud5
Spop
Fa2h
Abce1
Arhgef12
Elof1
Trpc4ap
Dok6
Bcap31
Mpv17
Tmem50b
Snrpc
Clns1a
Tubb3
Slmap
Wdr47
Rprd1a
Shank2
Map4k3
A830018L16Rik
Vstm2l
Pltp
Ptprz1
Ube3a
Pdcd10
Tsg101
Ubl4a
Ly6c1
Snapc5
St8sia5
Tln1
Cckbr
Becn1
Panx2
Kcnc1
Acsl6

Rps11
Ppp1r1a
Nenf
Tmem160
Rpl6
Map1lc3a
Pebp1
Rpl28
Rpl11
Ndufa13
Rps5
Pkm
Itm2c
Fkbp8
Cck
Rpsa
Rps3
Rpl24
Ndufb9
Rps10
Rps7
Rps2
Rpl8
Rpl9
Ttc9b
Rps15
Rps20
Rpl13
Rps27a
Pfdn5
Hbb-bs
Rpl4
Rpl13a
Rpl18a
Rpl21
Ttr
Fau
Gapdh
Rpl14
Rps8
Rpl10
Rps24

Src
Fxyd1
Neurod6
Mxi1
Arhgap23
Carm1
Nme7
Zmynd8
Rab5if
Anp32b
Rps27l
Mgat4b
Asrgl1
Etv5
Arxes2
Rab28
Rida
Mir124a-1hg
Clstn2
Trim28
Srsf10
Dop1b
Dctn4
Gpd1l
Tnk2
Rcn2
Pik3r3
Slc27a4
Med10
Rab3gap1
Dagla
Manf
Zyx
Enpp2
BC004004
Amz2
Gaa
Uba2
Tacc1
B3gat1
Slc25a23
Eid2
Ppib
Scarb2
Supt6
Gpd1
Dtnb
Arl6ip1
Mxd4
Negr1
Pcmtd2
Emc2

Slc25a18
Higd2a
Hdhd2
Chka
Sprn
Cryab
Prpf8
Slc8a2
Atp2c1
Speg
Arfgef1
Cstb
Klf13
Atg4b
Acyp1
Ddx1

CP iTBS

DEGs Young sham vs Aged sham	iTBS DEGs unique to young adults	iTBS DEGs unique to young adults that are also ageing DEGs
Gm10076	Rps27	Lrrc17
Uba52	Rps29	Rps27
Rpl35	Tmsb4x	Rps29
Gstp1	Pcp4	Tatdn1
Rps27	Tusc3	
Rps29	Rpl10	
Tmsb4x	Gas5	
Pcp4	Hmgn2	
Tusc3	Ftl1	
Rpl10	Tatdn1	
Gas5	Gm2000	
Hmgn2	Lrrc17	
Ftl1		
Tatdn1		
Gm2000		
Lrrc17		
Bc1		

CP cTBS

DEGs Young sham vs Aged sham	cTBS DEGs unique to young adults	cTBS DEGs unique to young adults that are also ageing DEGs
Gapdh	Lrrc17	AI593442
Uba52	Pde10a	Acat1
Rpl4	Rps28	Acyp2
Rps29	Scn4b	Add3
Lrrc17	Rps29	Agap2
Mbp	Ndufa1	Anapc16
Rps28	Rps27	Ank
Lzts3	Arpp21	Anks1b
Atp1a1	Tatdn1	Ano3
Slc17a7	Araf	Ap1ar
Cox8a	Ost4	Apbb1
Rpl21	Tspyl4	Araf
Snhg6	Snca	Arhgef12
Mobp	Actr2	Arpp21
Stmn3	Snrpf	Atp1b2
Fau	Snhg9	B2m
Rps24	Cox17	Basp1
Rps27	Ddn	Bloc1s1
Rpl5	Isca1	Bzw1
Ywhah	Sem1	Cacna2d3
Calm1	Snap25	Cbr3
Ndufa4	Ppp1r16b	Cck
Gng7	Rgs7bp	Ccng2
Eef1a2	Tomm7	Cd302
Malat1	Agap2	Cdk5r2
Ppp1r1b	Snhg8	Cdr1os
Olfm1	Plppr4	Cfap36
Ivns1abp	Ndrg4	Chst2
Bcas1	Bloc1s1	Churc1
Bex2	Cox7c	Cinp
Vsnl1	Sdhd	Clstn1
Pkm	Snhg6	Cnp
Dpysl2	Atp5md	Comt
Tuba1b	Slc6a1	Cox16
Ost4	Dmtn	Cox8a
Usp50	Basp1	Crym
Rpl9-ps6	Glul	Cystm1
Ddn	Rnf5	Ddn
Tubb5	Ndfip2	Dhx15
Rasd2	Eif4a2	Dnajb4
Itm2c	Spag9	Dock4
Tmsb10	App	Dync1i2
Lmtk2	Klf9	Eno1
Psap	Cdk5r2	Erh
Scn4b	Pacsin1	Ewsr1
Slc1a2	Lzts3	Fam120a
Ctxn1	Fbxl16	Fam174a
Gng3	Smim26	Fau
Ly6c1	Car2	Fgfr1op2

Mal	Comt	Fis1
Zdhhc14	Naa20	Fkbp8
Comt	Rpl39	Fth1
Rps20	Lpcat4	Gapdh
Rgs20	Meis2	Gja1
Cck	Ppp1r2	Gm33651
Psd	Ido1	Gng7
Ndufa1	Atp1a3	Gpm6b
Tatdn1	Slc4a4	Hdhd2
Rarb	Huwe1	Hnrnpa2b1
Rpl35	Gucy1b1	Id4
Rps4x	Phactr1	Ido1
Unc13c	Wsb2	Ids
Gstp1	Ewsr1	Insyn1
Rps27rt	Rab3c	Isca1
Bloc1s1	Cd302	Jph4
Rpl3	Phpt1	Kazn
Mog	Txn1	Klf13
Pfdn5	Psip1	Klf9
Cldn11	Fus	Klhl7
Rpl37a	Zwint	L1cam
Pgam1	Cops7a	Lmtk2
P4ha1	Srsf5	Lpcat4
Trak2	1810037I17Rik	Lrp11
Sema7a	Cntnap1	Lrrc17
Pde10a	Caln1	Lrrk2
Rgs4	Rgs14	Lsm8
Actb	Spry2	Luzp2
Ppp2r1a	Ap1ar	Lzts3
Crym	Usp50	Mapre2
Prkar1b	Kctd13	Meis2
Gpm6b	Ank2	Morf4l1
Trp53inp2	Rapgef4	Mrpl27
Tubb3	Rps27rt	Mrpl33
Epas1	Atp6v1a	Mrps28
Cinp	Sipa1l1	Mt2
Acyp2	Tsc22d4	Naa20
Grb14	Rpl9-ps6	Ndfip2
Gm10076	Rgs20	Ndufa1
Dnm1	Cs	Nenf
Hspa5	Rarb	Neto1
Ido1	Slc25a22	Ost4
Gnas	Id4	Pacsin1
Gpr88	Ndufa3	Pcdh17
Adcy5	Zfp365	Pdcd10
Slc2a1	Gabra4	Pde10a
Vcp	Mapre2	Pde4b
Rps2	Chst2	Pde4dip
Adora2a	Prrc2a	Pet100
Pdlim2	Jph4	Pfdn5
Arl6ip1	Selenof	Phactr1
Plppr4	Dnajb4	Pkm

Tspyl4	Acyp2	Plp1
Rpl24	Cacna2d3	Plppr4
Mt1	Arf4	Ppp1r16b
Tspan2	Cd47	Ppp2r1a
Cap1	Spop	Pptc7
Cd302	Tmem258	Prpf8
Eno1	Cdk17	Prrc2a
Ablim2	Gng7	Psap
Rraga	Rpl37a	Psd3
Ndufa7	Churc1	Psma5
Txn1	Rab28	Ptprz1
Akap9	Rcn2	Rab28
Ly6a	Ubqln2	Rai1
6330403K07Rik	Insyn1	Rapgef2
Rps7	Ano3	Rapgef4
Gamt	Ptprz1	Rarb
Sirt2	Scg2	Reep1
Dynll2	Camk2a	Rgs14
Tmeff2	Tmed7	Rgs20
Rabac1	Sdcbp	Rian
Polb	Hprt	Rnf5
Cdr1os	Acat1	Rpl14
Fkbp8	Ids	Rpl21
Agap2	Psd3	Rpl24
Acot7	Shank3	Rpl37a
Apbb1	Rheb	Rpl39
Tubb4b	B230334C09Rik	Rpl4
Mag	Mt2	Rpl5
Nptxr	Atp5mpl	Rpl6
Slc22a23	Ppp1r9b	Rpl9-ps6
Slc41a1	Sparcl1	Rps2
Mir124a-1hg	Lmtk2	Rps20
Lingo3	Mapk1	Rps24
Arhgap33	Gja1	Rps27
Rgs14	Paip2	Rps27rt
Golga7	Dock4	Rps28
Dock4	Spock3	Rps29
Rpl39	Atxn2l	Rps3a1
Pet100	Cfap20	Rps4x
Hnrnpa2b1	Gria2	Rps7
Kcnab1	Sowaha	Rraga
Ppp3r1	Strn	Rrp1
Tlnrd1	Slc4a10	Scn4b
Wnk1	Phyhip	Sdhd
Syp	Anks1b	Sec14l1
Prnp	Sft2d1	Sem1
Actn1	Smim14	Sgip1
Dlg2	Lmo4	Sipa1l1
Fam120a	Pet100	Slc4a4
Josd2	Romo1	Smim26
Lpcat4	Fam174a	Snca
Stmn1	Erh	Snhg6

Coro2b
Nme7
Mrpl33
Snhg9
Timm22
Nisch
Car12
Gng5
Tiam2
Slc32a1
Ano3
Clstn1
Arc
Pdzd2
Ugp2
Acat1
Sbk1
Dnajb11
Pde4dip
Cacna2d3
Mbnl2
Npdc1
Rasgef1b
Reep1
Atp6v1b2
Snca
Psenen
Klf9
Rpl6
Synpr
Sdhd
Smim27
Neto2
Slain1
Rpl14
Fa2h
Chst2
Ctsb
Nap1l5
Prkar2b
Gnb1
Slco1c1
Clic4
Sncb
L1cam
Ntsr2
Chordc1
Kcnq3
Ppm1b
Syn1
Fxyd2
Ostc
Hnrnpa2b1
Sgip1
Neto1
Rapgef2
Wdr89
Psma5
Tmeff1
Gas7
Stxbp1
Arpc2
Kazn
Gm33651
Mrpl27
Tgfa
Mrps28
Atp6v1h
Tacc1
Atp5g3
Brms1l
Fgfr1op2
Senp3
Ccng2
Pde4dip
Gad1
F3
Rragd
Crym
Chmp5
Frrs1l
Usp9x
R3hdm4
Klhl9
Cdr1os
Prkar1a
Trappc6b
Sec14l1
Atp1b2
Tmbim6
L1cam
Bzw1
Mrpl33
Dgkb
AI593442
Nedd4
Cmpk1
Gpm6b
Klf13
Add2
Morf4l1
Lmo3
Rai1
Arhgef12
Snhg8
Snhg9
Sowaha
Spop
Spry2
Ssr3
Stmn3
Strn
Tatdn1
Tgfa
Tmbim6
Tmeff1
Trf
Trpc1
Tspyl4
Txn1
U2af2
Unc50
Unc5a
Usp50
Usp9x
Vgf
Zfr

Atg3	Mat2b
Csf1r	Zcchc14
Thy1	Rian
Hmgcs1	Ndufb3
Meis2	Mgat3
Plekhj1	Rpl22l1
Tle4	Luzp2
Ly6h	Rap1gds1
Ppp1r16b	Gnao1
Btbd1	Map2k1
Insyn1	Dnajc6
Dpy19l3	Fam168a
Churc1	Plp1
Rps3a1	Cstb
Nenf	Lrrk2
Htr1b	Trrap
Gpr6	Atn1
Ryr3	Golph3
Unc80	Hpcal4
Sdhaf4	Hdhd2
Ntrk3	Inpp4a
Smpd3	Lars2
Mmd2	Prpf8
Sfxn1	Add3
Mt2	Meg3
Kctd12	Klhl2
Shisa4	Rraga
Asrgl1	Fam131b
Kcnj10	Azin1
Erbin	Atp8b2
Dnm2	Ppp2r2c
Cmtm5	Pptc7
Etv1	Erc2
Eif4a1	Cbx3
Gja1	Unc5a
Tmbim4	Cox8a
Ppdpf	Ssr3
Ttll11	Cystm1
Crocc	Wdr17
Ap1ar	Hnrnpa0
Mrpl10	Phyhipl
Scn3b	Nrxn1
Pon2	Unc50
Leprot	Yipf4
Dock10	Cinp
Hivep2	Prdx3
Eef2	Lrp11
Pts	Nr4a1
Gpr158	Nus1
Gpr155	Cacnb4
Syndig1	Kcna2
Htra1	Dcaf17

Robo2	Dync1i2
Elovl5	Klhl7
Snhg1	Zfr
Ift57	Arpp19
Srpk1	Reep1
Ttc9b	Prrc2b
Ezr	Ldb1
Sptbn1	Capza2
Syngr1	Tmed2
Ddit3	Slc1a3
B3gnt2	Ctnnb1
Gnptg	B230312C02Rik
Snrpa	Hmgb1
Tmem33	Abat
Calb1	B2m
Tmem229a	Dhx15
Cnp	Dnajb5
Taf13	Lsm8
Dguok	Tiprl
Rnf5	Hlf
Tbc1d9b	Vgf
Abce1	Cfap36
B230219D22Rik	Ank
Itm2a	Abhd3
Gsn	Rpl10-ps3
Pigp	Hypk
Lsm8	Rabggtb
Msra	Trpc1
Tmco1	Pcdh17
Btrc	Anapc16
Gabarap	Fam120a
Capzb	U2af2
Cacna1h	Ssb
Pllp	Pde4b
Ppp1r14a	Ube2d1
Scn1b	Cbr3
Epn1	Cox16
C1qtnf12	Pdcd10
Psat1	Pam
Homer1	Rps15a
Mrpl17	Ctsd
Lyrm2	Ubb
Isca1	Cd81
Eps15	Rps14
Naca	Trf
Enpp2	Rpl18
Atp6v1e1	Clstn1
Sowaha	Ndufb8
Abcd3	Rpl23
Smim11	Fkbp8
Rapgef2	Nefm
Dmkn	Rpl30

Ncoa2	Actg1
Far1	Psap
St8sia3	Rpl6
Snrpe	Stmn3
Eml5	Fis1
Ppid	Hras
Stard3	Rpl32
Dusp6	Ftl1
Gnb2	Apbb1
Ccdc88c	Rps11
Plp1	Rpl9
Drd2	Hba-a1
Sgip1	Ndufb10
Mapre2	Ndufb9
Ss18l2	Rpl11
Cbr3	Rrp1
Inpp5j	Fth1
Unc50	Rps5
Ndfip2	Ppp2r1a
Gltp	Rpl26
Cdk5r2	Ptgds
Tubb4a	Pkm
Myl12b	Rpl7
Myo6	Rps12
Hexb	Atp5d
Dag1	Smdt1
Fnip1	Pvalb
Zswim6	Rpl5
Epb41l2	Rps15
Igfbp7	Atp9a
Cspg5	Rpsa
Lrp11	Rps3a1
Vps29	Pebp1
Dync1h1	Fkbp2
Atf2	Rps3
Trappc4	Rpl28
Lmo7	Rpl8
Atp6v1c1	Cnp
Ube2g1	Ndufa13
Zfp445	Cck
Tpd52l1	Gapdh
Atraid	Rpl24
Phgdh	Rps10
Rbm3	Rpl18a
Max	Nenf
Dmac1	Rps4x
Cnst	Cryab
Anks1b	Rps2
Zfr	Rpl4
Gadd45g	Rps7
Sem1	Rpl14
Abhd17a	Rpl13

Pdia3
Scrg1
Inpp5a
Gcc2
Ndufv1
Capn2
Scamp3
Ipo7
Luzp2
Bhlhb9
Mark1
Usp33
Phactr1
Lcmt1
Sptb
Plpbp
Phldb1
Med4
Trf
Aldh6a1
Mphosph8
Syt6
Dhx15
Rfk
Wipf3
Mbd2
Kctd3
Prpf8
Csnk2a2
Rrp1
Fxyd1
Ubc
Cnot6
Jak1
Rab28
Gphn
Snapc5
Arpp21
Rnf166
Zfp385a
Isca2
Fez1
Mrps36
Ankrd10
Ramp1
Fmnl1
Hectd1
Mrps28
Pcdh17
Npas2
Prkch
Eml1
Rpl13a
Eno1
Fau
Rps27a
Pfdn5
Rps20
Rps8
Rpl10
Rpl21
Apod
Rps24
Hbb-bs
Ttr

Tubb2a
Rbis
Epb41l4aos
Kpnb1
Phlpp1
Maged1
Fnbp1l
Ina
Scg5
Hadhb
Rph3a
Jund
Nebl
Araf
Hs3st4
Dmxl2
Pfn1
Clk3
Park7
Gba2
Tppp3
Fhl1
Gng11
Olig1
Mn1
9330159F19Rik
Ctnna1
Zcchc24
Nexn
Lrrk2
Sec14l1
Tcf25
Rap1a
Eif4a3
Vbp1
Slc9a1
Ptprz1
Pmepa1
Pycr2
2010204K13Rik
Ccdc106
Phyh
Slc39a3
Grik2
Pnisr
Grm4
Tmod1
Wbp2
Gas2l1
Xbp1
Pitpnc1
3110039M20Rik

Acap2
Plppr5
Car11
Ssr3
Clvs1
Utrn
Setd7
Macf1
Snhg8
Agrn
Cenpx
Cacng4
Coa6
Api5
D5Ertd579e
Pdp1
Ssbp1
Selenop
Schip1
Higd2a
Lap3
Opalin
Efcab14
Gprc5b
Mapk1ip1
Ogt
Fads1
Sez6l2
Slirp
Chmp2b
Rnf13
Grn
Luc7l
Mrpl57
Lage3
Clip1
Brwd1
Marcks
Snrpc
Atp6v1g2
Tbc1d8
Neat1
Tln1
Ythdf1
Pcdh10
B2m
Ak3
Snapin
Pptc7
Mbtps1
Myh10
Srrt

Kif17
Dusp11
Vps52
Smarca5
Spata2l
Vps35
Ppp4r2
Sec23a
Ermn
Arhgap21
Tuba4a
Kdm4b
Stmn2
Ppp2r5a
2510009E07Rik
Lrrtm1
Tagln3
Xpo1
Apc
Prrc2a
Atp6v0e
Scd1
Atg12
Aggf1
Rapgef4
Tmem167
Isyna1
Ctss
Gramd1b
Psmg4
Ankrd13a
Abhd4
Rbp4
Nipa1
Rgs8
Usp3
Fxyd7
Pdk3
Arhgef2
Letmd1
Xpo7
Tmem47
Gtf3c2
Map7
Mcf2l
Ccnh
Got1
Tmem183a
AI593442
Mpv17l
Vgf
Ric8b

Cldn5
Sh3rf2
Myo1b
Serinc5
Snhg20
Ifrd1
Hax1
Id4
Cdk2ap2
Yipf3
Mpped2
Prkacb
Spry2
Unc5a
Tsc22d3
Rabggta
Tyrobp
Smim26
Zfas1
Pbx3
Gng12
Tmem170b
Arhgdib
Fam216a
Dtna
Ryr1
Kdm3a
Ciao2b
Cd9
Sesn1
Foxk2
Add3
Nsd2
Ralgapa1
Cobl
Fcho1
Acy1
Pttg1ip
Dab2ip
Pacsin1
Tra2a
Dusp3
Fyn
Paqr8
Tnk2
Rian
Dip2c
Mxd4
Chka
Gdap1
Dld
Rbp1

Mrps11
Il33
Dpf2
Foxo1
Ccdc85c
Six3
Mrpl40
Uqcrc1
Polr2f
Degs1
Mzt1
Cfap36
Aldh5a1
Polr2i
Vat1l
Tmem208
S1pr1
Ankrd13c
Nme2
Slc35a4
Cope
Ldah
Surf1
Ephx1
Phf24
Apba1
Pou3f3
Plpp6
Caprin1
Vps9d1
Gaa
Med27
Tafa5
Neto1
Nat8f1
Fnta
Golga4
Cdk19
Sh3kbp1
Azi2
Ube2e3
Rcn1
Acvr1c
Mcee
Smim20
Dnajb4
Fgf13
Ube2m
Mrpl44
Smad3
Rtf2
Ralgds

Srsf6
Ccnl2
Epb41l3
Ppp6r1
Nomo1
P4hb
Fut8
Csnk1d
Fam120b
Ddx50
U2af2
Hsd17b12
Grk6
Cstf2t
Pdxp
Dpy30
Limch1
Tram1l1
Srr
Agtpbp1
Polr2j
Stip1
Eif2s1
Faim2
Nrep
Sh3gl3
Immp1l
Hmgcl
Ccm2
Rubcn
Gnb5
Taf1b
Dpm3
Chrm1
Kansl2
Mrpl13
Fnbp1
Fbxl3
Mettl23
Fabp3
Chgb
Ddx1
Emc3
Ahcyl2
Srsf7
Rabl6
Arl8a
Gpsm1
Gm10419
Arhgap23
Ppp4r4
Zfp871

Fis1
Slitrk5
Rnf181
Lsm7
Sccpdh
Bcr
Lrrc10b
Kazn
Dhrs7
Sec11a
Armc1
Polr2k
Ubqln1
Actn2
Relch
Ktn1
Dnaja4
Manf
Fam174a
Adgrl2
Sumo2
Rgs2
Saraf
Plxna2
Sh3gl2
Tbc1d16
Trappc13
Dclk2
Fcer1g
Smg1
Hspd1
Zmym2
Wscd1
Tardbp
Pcmtd2
Dixdc1
Slc35f3
Mctp1
Stk32a
Sbds
Ube2i
Sf3b5
Asb13
Pmm1
C2cd2l
Rhot1
Cdk16
Asah1
Galnt13
Mink1
Cdo1
Junb

Abcc5
Rab10
Acyp1
Ap1s2
Rprd1a
Nme1
Nxf1
Bud23
Rmdn3
Sppl3
Chn2
Snx2
Ccdc47
Ttc9
Sv2a
Fcor
Mtus1
Nin
Trim9
Osbpl2
Atp8a1
Polr2g
Cox20
Dnajc2
Rab40c
Pltp
Dmtf1
Mapkap1
Maz
Mdh2
Anapc16
Cyb5a
Papss1
Tomm34
Phf20l1
9130401M01Rik
Adcy1
Slc35c2
Vma21
Stub1
Akap8
Pdcd10
Kbtbd2
Fth1
Dctpp1
Crat
Aph1a
Prkaca
1110038F14Rik
Maneal
Snrnp27
Coro7

Tomm5
Lrrc4b
Shisa9
Gnpat
Ndufaf5
Txndc16
Commd6
Tmem134
Ptpn4
Uba5
Rpp21
Scarb2
Lpl
Wbp1
Rps6ka5
Sri
Rbfa
Cacng3
Ino80e
Jak2
Hnrnpc
Dpm2
Sipa1l3
Ppp2r2d
Lamtor3
Slc35d3
Sel1l
Pdzd8
Timm10
Scn3a
Tmem201
Spop
Ripor2
Sdr39u1
Akap5
Myl12a
Aopep
Vta1
Cystm1
Ndrg1
Yme1l1
Mtmr9
Stk19
Sod1
Slc1a1
Srgap1
Naa20
Dmwd
Phf5a
Dop1b
Skil
Timm9

Cstf3
Bcl11a
Shb
Cd4
Abl2
Emc6
Large1
Stau1
Akt3
Son
Gmpr
Prr7
Wwc1
Secisbp2l
Iah1
Pantr1
Dctn5
Ap3b1
Dut
Cdc34
Ubr2
Cacybp
Casc3
Rala
Cd99l2
Bap1
Hikeshi
Ilk
Arih1
Pde4b
Rhobtb2
Dctn4
Mrpl32
Usp9x
Osbpl1a
Gpcpd1
Ewsr1
Orc4
Esd
Mrpl16
Hspa4l
Senp2
Jtb
Ankrd37
Chtop
Dagla
Morf4l1
Chd9
Rgs5
Cnn3
Gcsh
Jkamp

Ankrd40
Chmp4b
Klhl7
Cox16
Tmem185a
Rmnd5b
Plekhb1
Gga3
Lgi1
Sfpq
Slc49a4
Cstf2
Pxdn
Plcxd1
Fzd3
Mrpl3
Pip5k1a
Ube2a
Tpgs2
Prelid3b
Maea
Apbb3
Grsf1
Hnrnph3
Uba3
Stam
Bud31
Dda1
Arel1
Mien1
Slk
Enah
Gabrb3
Pcna
Timp3
Nedd4l
Pdia6
Fbxw11
Bptf
C1qb
Dtymk
Carm1
Lrtm2
Cacnb1
Ube2f
Otulinl
Kcnk1
Rnf6
Smpdl3a
Cdh13
Dcaf7
Snd1

Sipa1l1
Scg3
Plekhn1
Fabp5
Itgb1bp1
Serp1
Clta
Serinc3
Tor1aip2
Ank3
Ankmy2
Rad23a
Cacna1c
Atp1b2
Kmt2e
Asic4
Gpatch8
Appl2
Rc3h2
Tbc1d20
Ankrd46
Ncbp1
Irf2bpl
Ubald2
Clk1
Il18
Ncor2
Trp53bp1
Hsd17b10
St6galnac5
Tex264
Fgfr1op2
Atxn10
Iffo1
Hbs1l
Fubp1
Chst15
Ppib
Emc2
Spred1
Dnal4
Gcnt2
B3galt5
Vps53
Sh3bgrl
Jph4
Slc44a1
Sae1
Ap3m1
Gsg1l
Acaa2
Psmd13

Snap47
Etv5
Slc25a44
Rai1
Gpd1
Zdhhc18
Ado
Mbnl1
Slc25a46
Dst
Ranbp3
1110059G10Rik
Ctdsp2
Ipo11
Tm2d2
Tprkb
Ccng2
Ppa2
Pip4p1
Abhd16a
Usp32
Srm
Krt10
Syn2
Tax1bp1
Abhd17c
Gm3764
Ppp1r12a
Ccdc32
Snx1
Timm29
Rell2
Ubp1
Zfp180
Necab1
Cnot7
Rnf114
Tmub2
Mat2a
Cnih1
Fam8a1
Rab5if
Micu1
Rbm7
Epn2
Gm33651
C1qc
Hmbs
Taf11
Rdh14
Slc25a25
Plec

Arhgap5
Srsf1
Pnrc1
Stau2
Slc30a9
Mpp1
Mocs2
Rnf4
Sybu
Atg4b
Ncoa1
Dnajb9
Rnf34
Ran
Sorcs2
Chchd3
Slc35b4
Ddit4l
Dhx36
Ppp5c
Krtcap2
Malsu1
Arfrp1
Rbm18
Nktr
Lrpprc
Naxd
Nsf
Cdc42ep3
Wdr26
Cisd2
S100a13
Ap3s1
Hnrnpll
Dhx32
Dync1i2
Luc7l2
Sst
Nipsnap1
Ank
Ppp6c
Ubr3
Ndufaf3
Mrpl41
Nt5c3
Pfkm
B4galnt1
Becn1
Trim2
Prickle2
Sun2
Cdadc1

Thyn1
Leprotl1
Afdn
Cnr1
Kmt5a
Ptgr2
Prpf4b
Hadha
Cdc42bpa
Psd3
Lypd1
Tmem131
Dnajb2
Msmo1
Gm2a
Pik3c3
Lrrtm2
Nsd1
Rbm33
Endod1
Ccdc28b
Mageh1
Zeb1
Alkbh7
Foxp2
Lman2
M6pr
Cbx4
Msrb1
Ulk2
Pten
Cnih4
Zdhhc2
Med21
Supt6
Rin1
Zfp637
Scrt1
Fibp
Ubr5
Adk
Thsd7a
Adcyap1r1
Mrps12
Elp5
Opa1
Mtdh
Kif3c
Cnot2
Ubac1
Ndst1
Impdh1

Rgs10
Ctnna2
Trpc4ap
Syt16
Fkbp4
Ythdf3
Pigs
Xiap
Tmeff1
Yipf5
Polr2l
Plxnd1
Pgs1
Usf2
Pomgnt1
Zranb2
Leng8
Sugt1
Sh3glb1
Camk4
Sirt7
Tsr3
Gpam
Mpp3
Eef1g
Psmc6
Stt3b
Mrpl27
Gskip
Hpf1
Gna12
Desi1
Plppr1
Ube2h
Ubl4a
Arsb
Zhx1
Uso1
Gtpbp6
Pclo
Trap1
Efna3
Cacnb2
Ncbp2
Ralbp1
Bri3
Exoc5
Atxn1
Tia1
Ica1
Irak1bp1
Akt1

Tcf4
Ubr4
Cdc123
Mlec
Ubxn2b
Otud6b
U2af1l4
Ndel1
Bckdha
Ggt7
Kbtbd11
Npy
Tomm40
Med9
Abracl
Zfp638
Gosr2
Tmbim6
Eif1a
Gpr27
Ilkap
Trim11
Erg28
B4galt3
Pi4k2a
Tug1
Babam2
Tmem68
Elp1
Slmap
Dipk1b
Sacm1l
Klf13
Tmem14a
Usp7
Atp6v0a2
Picalm
Med25
Tmem126a
Pum1
Rnf20
Lrrc8b
Snhg12
Ube3c
Eif3e
Eef1akmt1
Sap18
Strn
Fgf12
Adgrb3
Ddx42
Mal2

Basp1
Acvrl1
Gpbp1
Tgfa
Lyrm4
Rnf7
Ckmt1
Jagn1
Dctn2
Rab26
Man1a2
Tmx3
Wasl
Arl6
Igfbp4
Evi5l
Tceal8
Mapk9
Scrt2
Eif4g1
Seh1l
Fbxo11
Fundc1
Gap43
Snap91
Shroom2
Cttnbp2
Ylpm1
BC004004
Cfl2
Oaz2
Elof1
Vps11
Tmem181a
Top1
Manbal
Cacna1e
Kcns2
Acbd5
Kctd1
Syne1
Anapc5
Clns1a
Magi2
Chst11
Ddah1
Abhd14a
Tmed4
Nfx1
Mrpl50
Nicn1
Dgke

Magoh
Rab11fip3
Mtor
Tmem184b
Etf1
Txnl4a
Cox19
B9d2
Akt1s1
Aup1
Ehbp1l1
B4galt2
Zfp580
Emg1
Adam15
Trpc1
Glcci1
Mdp1
Mrps7
Nat8l
Sult4a1
Aldh7a1
Sdc2
Blcap
Nt5c2
Srsf10
Slc25a1
Anapc2
Slc4a4
Hexa
Nudt4
Got2
Mrpl49
Scmh1
Pdzd11
Cipc
Lsm1
Gtf2a2
Bzw1
Ids
Tmem263
Snw1
Sec61a1
Zbtb8os
Mia3
Sgcb
Med10
Bphl
Snx27
Adra2c
Dhx30
Ptpa

Morn4
Pde8b
Vezt
Rnpep
Zmynd8
Coa3
Vtn
Rgs7
Plekhm2
Ckap5
Ebna1bp2
Kif5c
Traf7
Naa50
Gclm
Trappc11
Plekha3
Ddit4
Myo9a
Adcy3
Rad21
Snx5
Ankrd17
Slc9a5
Kat7
Ufd1
Pla2g12a
Caml
Chst1
Mark2
Pnn
Pacsin2
Mrpl46
Psma5
Psen1
Amer2
Trim3
Ube2j1
Arhgef12
Coq10b
Copa
Ube3a
Tango2
Slc38a3
Ncoa7
2300009A05Rik
Tmem59l
Osgep
Ptpn11
Pkp4
Peli1
Ipo13

Flot1
Ajap1
Ttc14
Tmem205
Plxnb1
Agap1
Cop1
Abcb9
Hdhd2
Limk2
Fam91a1
Cyfip1
Med14
Rbbp4
Prkca
Srgap2
Sf3b3
Mib2
Lman2l
Rit1
Dalrd3
Celf3
Exosc7
Nmt2
Trip12
Ski
Stx7
Ufm1
Pdk2
Arid1a
Mfap1b
Commd1
Hsd17b4
Tpst2
B3galt1
Mxi1
Rab3ip
Cdk5r1
Nrbp1
Ppip5k1
Yaf2
Ppil4
Mfsd13a
Cuedc2
Ecpas
Lsm14a
Rsrc2
Nfasc
Znhit3
Trappc12
Eif3c
Prkcsh

Sf3b4
Wdr45b
Trmt1
Fam107a
Nipa2
Tppp
Upf1
0610010K14Rik
Arf2
Nr2c2
Syt3
Ruvbl1
Pls3
Metap2
Pigx
Aldh1l1
Calcoco1
Kcna4
Morn2
Mob4
Fam117b
Erh
Zdhhc3
Pcbp3
Ache
Ube2q1
Mrpl36
Rbm28
Mrpl4
Ganab
Map3k10
Dapk3
Nipsnap2
Agpat5
Slc6a8
Mydgf
Tmem70
Parp1
R3hdm1
Gbf1
Ifi27
Naa10
Coq9
Oxa1l
Gfra4
Gdpd5
Ap1g1
Mrpl14
Aff3
Cyth2
Fech
Rpl7l1

Med19
Ociad2
Hdgf
Sdhaf1
Hmgcr
Mrtfa
Fbll1
Otud5
Arfgef1
Foxo3
Ralgapb
B230217C12Rik
Ogfr

Lat Septal Complex iTBS

DEGs Young sham vs Aged sham	iTBS DEGs unique to young adults	iTBS DEGs unique to young adults that are also ageing DEGs
Cpe	Gstp1	Bc1
Uba52	Ppp1r1b	Cartpt
Mt1	Camk2n1	Gpm6b
Rpl4	Cck	Lrrc17
Slc1a2	Eno1	Pcp4
Rps24	Pde1b	Penk
Mt2	Pcp4	Rpl37
Bc1	Penk	
Ndufa4	Gpm6b	
Rpl5	Itpr1	
Thrsp	Fbxl16	
Rpl37	Canx	
Rpl14	Rgs4	
Gapdh	Arpp21	
Cox8a	Ndn	
Fau	Syt5	
Rps27rt	Rpl37	
Rpl21	Lrrc17	
Malat1	Tatdn1	
Ppia	Gm2000	
Ddn	Nts	
Fam107a	Cartpt	
Cnih2	Bc1	
Nme7		
Rps7		
Rps10		
Pcp4		
Rps29		
Rpl9-ps6		
Rps20		
Camk2a		
Ezr		
Dbi		
Bok		
Rps27a		
Dbn1		
Zcchc24		
Fads1		
Rpl9		
Agap2		
Epas1		
Calm2		
Mlc1		
Whrn		
Myo6		
Pfdn5		
Synm		
Pfn1		
Usp50		

Slc1a3
Rps28
Rps8
Paqr8
Zic1
Stmn1
Neurl1a
Lrrc17
Rpl35
Itm2c
Igfbpl1
Gng4
Chst2
Kcnj10
Atp1a1
Penk
Rpsa
Kctd12
Rpl35a
Lats2
Doc2g
Ctnnb1
Scn1b
Cacna1g
Cacng5
Pde2a
Necab2
Klhl5
Rpl3
Dlg2
Lars2
Gap43
Rbm3
Hspa14
Rps4x
Tprkb
Rpl24
Tspyl4
Rps2
Timp3
Nbl1
Rps11
Ppm1e
Ndrg2
Aldh5a1
Sptbn1
Aldh7a1
Cartpt
Gfap
Shank1
Slc8a1
Ldah

Snhg6
Arhgef12
Dpysl2
Ewsr1
Fhl1
Dag1
Prkcd
Rps3
Stac2
Slc39a12
Lrrfip1
Ndufa7
Slco1c1
Prdx1
Virma
Pitpnm3
Trim8
Atp1a2
Atp2a2
Kansl2
Ivns1abp
Psph
Tcf4
Mvb12b
Timm22
Ifngr2
Kif5c
Hs3st2
Tnfrsf21
Maneal
Rims2
Nfia
Cdk2ap2
Tubb5
Chd3
Map4k3
Slc6a1
Il6st
Pygb
Nenf
Mink1
Pea15a
Vim
Setd7
Atxn1
Tbc1d9b
Plekha1
Eef1a2
Stmn4
Ube2ql1
Ost4
Lsm2

Chordc1
Chd9
Tmem47
Clu
Prmt5
Klc1
Jund
Nfib
Sulf2
Twf2
Zic4
Clic4
Gpr137
Trpm3
Lix1
Map9
Mir100hg
Sh3bgrl
Elfn2
Rph3a
Jak1
Epb41l2
Zfr
Rgs10
Plcg1
Enah
Nfkbia
Ppp1r9b
Ly6a
Arhgap1
Txndc16
Tssc4
Zfp521
Parva
Gpm6b
Acss1
Gpam
Sacm1l
Hdac6
Gldc
Map4
Rab31
Gpd2
Sox1ot
Ythdf1
Cnot6l
Zfr2
Ids
Brinp2
Nwd1
Map7
Epn2

Btbd1
Zdhhc14
Abhd17b
Araf
Apc
Api5
Mmd2
Gnb2
Ahcyl2
Fbxo41
Cep120
Ap1s2
Tpd52l1
Rapgefl1
Scrt1
Foxk2
Shd
Exoc1
Trappc5
Eif4a1
Epha4
Pdzd2
Adk
Pacsin1
Mat2a
Fam120a
Ywhah
Sp9
Dok6
Iqsec3
Megf9
Atg4b
Prxl2b
Sgip1
Gadd45g
Crat
Zeb2
Sptan1
Kcng1
Luc7l2
Cdr1os
Ormdl3
Ak3
Abcb7
Rc3h2
Fam210b
Nif3l1
Prdm16
D430019H16Rik
Acyp2
Srgap3
Adarb1

Homer2
Nubp2
Plppr4
Glul
Ddx17
Mark4
Pmpca
Vma21
Klhl24
Eps15
Syt1
Bud23
Serp1
Nek6
Prelp
Clmn
Immt
Slc6a11
Tfdp1
Arhgef2
Osbpl9
Ttyh1
Zfp423
Nf2
Dlx6os1
Dvl1
Calb1
Mrtfa
Cnn3
Ptpn4
Camsap1
Idh2

Lat Septal Complex cTBS

DEGs Young sham vs Aged sham	cTBS DEGs unique to young adults	cTBS DEGs unique to young adults that are also ageing DEGs
Cpe	Tatdn1	Acyp2
Uba52	Araf	Adk
Mt1	Tspyl4	Agap2
Rpl4	Actr2	Ahcyl2
Slc1a2	Agap2	Aldh5a1
Rps24	Slc6a1	Apc
Mt2	Prkcd	Araf
Bc1	Basp1	Arhgef12
Ndufa4	Snap25	Atxn1
Rpl5	Rasd1	Cacna1g
Thrsp	Glul	Camk2a
Rpl37	Snhg9	Cdr1os
Rpl14	Ddn	Chd3
Gapdh	Tomm7	Chd9
Cox8a	Isca1	Chst2
Fau	Ndufa1	Clic4
Rps27rt	Camk2a	Cpe
Rpl21	Atp1a3	Ctnnb1
Malat1	Snca	D430019H16Rik
Ppia	Hopx	Ddn
Ddn	Car2	Dlg2
Fam107a	Ost4	Elfn2
Cnih2	Snrpf	Eps15
Nme7	Pacsin1	Ewsr1
Rps7	Ppp1r9b	Fads1
Rps10	App	Fam120a
Pcp4	Phpt1	Fau
Rps29	Ptprz1	Gapdh
Rpl9-ps6	Ank2	Glul
Rps20	Bloc1s1	Gpm6b
Camk2a	Snhg8	Ids
Ezr	Sem1	Kctd12
Dbi	Mbp	Map4
Bok	Ndrg4	Megf9
Rps27a	Naa20	Mt2
Dbn1	Eif4a2	Nenf
Zcchc24	Sdhd	Ost4
Fads1	Plppr4	Pacsin1
Rpl9	Slc4a4	Paqr8
Agap2	Atp6v1a	Pfdn5
Epas1	Gja1	Plppr4
Calm2	Zwint	Pmpca
Mlc1	Wsb2	Ppp1r9b
Whrn	Atn1	Prkcd
Myo6	Rnf5	Rpl14
Pfdn5	Snhg6	Rpl21
Synm	Slc25a22	Rpl24
Pfn1	Cdk5r2	Rpl3
Usp50	Spag9	Rpl35a

Slc1a3
Rps28
Rps8
Paqr8
Zic1
Stmn1
Neurl1a
Lrrc17
Rpl35
Itm2c
Igfbpl1
Gng4
Chst2
Kcnj10
Atp1a1
Penk
Rpsa
Kctd12
Rpl35a
Lats2
Doc2g
Ctnnb1
Scn1b
Cacna1g
Cacng5
Pde2a
Necab2
Klhl5
Rpl3
Dlg2
Lars2
Gap43
Rbm3
Hspa14
Rps4x
Tprkb
Rpl24
Tspyl4
Rps2
Timp3
Nbl1
Rps11
Ppm1e
Ndrg2
Aldh5a1
Sptbn1
Aldh7a1
Cartpt
Gfap
Shank1
Slc8a1
Ldah

Luzp2
Rps27rt
Arpp21
Chst2
Rpl9-ps6
Prrc2a
Slc1a2
F3
Ppp1r1b
Cox7c
Map2
Rab3c
Mt2
Tmed7
Kctd13
Gpm6b
Acyp2
Plp1
Tsc22d4
Sipa1l1
Ctnnb1
Rcn2
Prpf8
Usp50
Atp5md
Mgat3
Gnai1
Phactr1
1810037I17Rik
Slc4a10
Psip1
Ubqln2
Huwe1
Ids
Fam131b
Fbxl16
Cpe
Megf9
Ahcyl2
Fus
Slc1a3
Cs
Id4
Dmtn
Jph4
Sparcl1
Man1a
Cops7a
Prrc2b
Ppp1r16b
Hpcal4
Phyhip

Rpl4
Rpl5
Rpl9
Rpl9-ps6
Rps10
Rps11
Rps2
Rps20
Rps24
Rps27a
Rps27rt
Rps3
Rps4x
Rps7
Rps8
Rpsa
Setd7
Sgip1
Shank1
Slc1a2
Slc1a3
Slc39a12
Slc6a1
Snhg6
Sptan1
Srgap3
Syt1
Tbc1d9b
Tmem47
Trim8
Tspyl4
Ttyh1
Usp50
Zcchc24

Snhg6	Txn1
Arhgef12	Rpl39
Dpysl2	Rusc2
Ewsr1	Camk2n1
Fhl1	Atp6v1h
Dag1	Kctd12
Prkcd	Peg10
Rps3	Ap1ar
Stac2	Scd1
Slc39a12	Lmtk3
Lrrfip1	Stxbp1
Ndufa7	Klf13
Slco1c1	Tmbim6
Prdx1	Ndfip2
Virma	Dnajb5
Pitpnm3	Ndufa3
Trim8	Rap1gds1
Atp1a2	B2m
Atp2a2	Brms1l
Kansl2	Dock4
Ivns1abp	Ncan
Psph	Cfap20
Tcf4	B230312C02Rik
Mvb12b	Gad1
Timm22	Tbc1d9b
Ifngr2	Gnao1
Kif5c	Rab28
Hs3st2	Spop
Tnfrsf21	Kif3c
Maneal	Crk
Rims2	Rheb
Nfia	Peg3
Cdk2ap2	Selenof
Tubb5	Eps15
Chd3	Cadm4
Map4k3	Cd9
Slc6a1	Shank1
Il6st	Khsrp
Pygb	Arpc4
Nenf	Paip2
Mink1	Plekhb1
Pea15a	Ank
Vim	Prkar1a
Setd7	Rerg
Atxn1	Pde10a
Tbc1d9b	Add3
Plekha1	Senp3
Eef1a2	Stum
Stmn4	Abhd3
Ube2ql1	Hectd4
Ost4	Cds2
Lsm2	Atp5g3

Chordc1	Cdk17
Chd9	Fam168a
Tmem47	Qk
Clu	Apc
Prmt5	Wdr89
Klc1	Hprt
Jund	Pou3f3
Nfib	Cd47
Sulf2	Elfn2
Twf2	Sptan1
Zic4	Clic4
Clic4	Bzw1
Gpr137	Fam219a
Trpm3	Necap1
Lix1	Fads1
Map9	Usp19
Mir100hg	Fam102a
Sh3bgrl	Smim4
Elfn2	Kif5a
Rph3a	Cbx3
Jak1	Dlx1
Epb41l2	Smim26
Zfr	Sft2d1
Rgs10	Tuba1a
Plcg1	Syngap1
Enah	Ccdc85c
Nfkbia	Fam171b
Ppp1r9b	Arhgef12
Ly6a	Tmem47
Arhgap1	Chd9
Txndc16	Csrnp3
Tssc4	Hnrnpa2b1
Zfp521	Rapgef4
Parva	Ewsr1
Gpm6b	Ttyh3
Acss1	Gsk3b
Gpam	Setd7
Sacm1l	Tm9sf2
Hdac6	Arhgap39
Gldc	Atp1b2
Map4	Slc39a12
Rab31	Rpl10-ps3
Gpd2	Gria2
Sox1ot	Atxn2
Ythdf1	Psma5
Cnot6l	Yipf4
Zfr2	Cntnap1
Ids	Hnrnpa3
Brinp2	Nol4l
Nwd1	Atxn2l
Map7	Dnajc6
Epn2	Dnajb4

Btbd1	Chmp5
Zdhhc14	Acsl3
Abhd17b	Ttc7b
Araf	Usp9x
Apc	Anks1b
Api5	Gng7
Mmd2	Mrps28
Gnb2	Ripor2
Ahcyl2	Trrap
Fbxo41	Ssr3
Cep120	Kif1b
Ap1s2	Map4
Tpd52l1	Cyth1
Rapgefl1	Tsc22d3
Scrt1	Klf9
Foxk2	Zcchc24
Shd	Ccni
Exoc1	U2af2
Trappc5	Mtmr9
Eif4a1	Abat
Epha4	Aldoc
Pdzd2	Sgip1
Adk	Lamp1
Pacsin1	Aldh5a1
Mat2a	Trim8
Fam120a	Dact3
Ywhah	Mapre2
Sp9	Larp1
Dok6	Tmem258
Iqsec3	Syt1
Megf9	Chd3
Atg4b	Dlg2
Prxl2b	Rraga
Sgip1	Gucy1b1
Gadd45g	Dhx15
Crat	Srsf5
Zeb2	Cdr1os
Sptan1	Comt
Kcng1	Ogdhl
Luc7l2	Acat1
Cdr1os	Morf4l1
Ormdl3	Fbrsl1
Ak3	Bmpr2
Abcb7	Msi2
Rc3h2	Hectd2
Fam210b	Ttyh1
Nif3l1	Nova2
Prdm16	Hacd2
D430019H16Rik	Arel1
Acyp2	Nus1
Srgap3	Tnk2
Adarb1	Serinc3

Homer2
Nubp2
Plppr4
Glul
Ddx17
Mark4
Pmpca
Vma21
Klhl24
Eps15
Syt1
Bud23
Serp1
Nek6
Prelp
Clmn
Immt
Slc6a11
Tfdp1
Arhgef2
Osbpl9
Ttyh1
Zfp423
Nf2
Dlx6os1
Dvl1
Calb1
Mrtfa
Cnn3
Ptpn4
Camsap1
Idh2

Max
Mapk1
Pde4b
Frmpd3
Sbds
Pdpk1
B230334C09Rik
Umad1
Fgfr1op2
Fam120a
Shank3
Atp5mpl
9330159F19Rik
Rragd
Mrpl10
Abhd8
Lzts1
Rasd2
Pde4dip
Gabra4
Bhlhe40
Adk
Prkcb
Cacna1g
Srgap3
Lin52
Slc8a2
Cdc42se2
Rpl37a
Mpv17l
Pmpca
Dlgap1
Srrm1
Hnrnpu
Nutf2-ps1
Rnf141
Lpcat4
D430019H16Rik
Smim10l1
Xiap
Zcchc14
Gm20594
Unc80
Lsm8
Unc50
Paqr8
Spry2
Eno1b
Atxn1
Hibadh
Egln1
Rgs7bp

Rap1a
Dazap2
Miga1
Thra
Tiprl
Pmm1
Rps17
Ndufb8
Rpl3
Rpl27a
Psmc5
Rpl23a
Rpl7
Gpx4
Rps14
Ndufb11
Rpl29
Fdx2
Uqcrh
Timm8b
Hint1
Rpl28
Rpl23
Naca
Rpl36al
Rpsa
Ndn
Rplp1
Rps19
Eif3k
Nme2
Ubb
Edf1
Ftl1
Rpl5
Rpl35a
Rpl8
Ndufb9
Rps15
Rpl30
Eno1
Dynll1
Rps3a1
Rpl19
Rpl17
Rpl4
Rpl11
Pebp1
Rps4x
Rps16
Rpl32
Rps5

Rps2
Rpl18a
Gapdh
Rpl26
Rpl6
Rpl13
Rps15a
Rpl9
Rps3
Rpl18
Rps12
Pfdn5
Rpl24
Nenf
Rps11
Rps10
Rps20
Rpl13a
Ndufa13
Rps7
Rps27a
Fau
Rpl21
Rpl14
Hbb-bs
Rps8
Rpl10
Rps24

Striatum iTBS

DEGs Young sham vs Aged sham	iTBS DEGs unique to young adults	iTBS DEGs unique to young adults that are also ageing DEGs
Bc1	Eno1	Lrrc17
Cox8a	Pcp4	Mbp
Actb	Hmgn2	Pcp4
Rps24	Mbp	
Malat1	Nrgn	
Rpl4	Agap2	
Sst	Snhg9	
Fau	Lrrc17	
Dpysl2		
Pcp4		
Mt1		
Rpl3		
Rpl5		
Bloc1s1		
Rasgrf2		
Snca		
Rpl37		
Rpl13		
Eef1a1		
Ly6c1		
Usp50		
Nts		
Gamt		
Otof		
Rpl14		
Rps10		
Ntsr2		
Hspa5		
Snhg6		
Ddn		
Lingo3		
Cpe		
Itm2c		
Rps8		
Ptgds		
Uba52		
Cacna1h		
Calm1		
Tsc22d1		
Them6		
Atp1b1		
Polr2l		
Ndrg4		
Cox7b		
Rpl13a		
Rps2		
Psd		
Rpl9		
Polb		

Kcnip2
Strip2
Snhg11
Rps7
Josd2
Ankrd13b
Ndufa4
Nefm
Unc13c
Tac1
Pfdn5
Polr2j
Mbp
Snhg20
Necab1
Cck
Pacsin2
Arhgap33
Maneal
Mal
Higd2a
Coro7
Ccm2
Zdhhc14
Trak2
Dalrd3
Rps3a1
Jsrp1
Arpp21
Lrpap1
Camk1g
Snhg1
Srsf11
Mrpl41
Pitpnm2
Eef1a2
Gucy1a1
Sdhaf2
Chordc1
Rac3
Acvr2a
Rpl9-ps6
Rnf20
Srp72
Smpd3
Rps14
Yipf1
Morf4l1
Acsbg1
Rps27a
Mapk1ip1
Ppid

Oga
Sirt2
Mat2a
Pfn1
Btbd3
Agrn
Elp2
Ralgds
Sirt3
Scn4b
Limd2
Mrpl21
Slc2a1
Emc2
Lyst
Ly6a
Rps4x
Epas1
Taf13
Tmem230
Ost4
Kansl2
Amz2
Filip1
Arel1
Slc35d3
Rgl2
Acyp2
Tmub2
Rpl30
Nedd4l
Tubb5
Tbc1d9b
Pttg1ip
Prxl2a
Brinp1
Nlgn1
Trmt1
Cbx4
Inf2
Rasd2
Pvalb
Ccdc28b
Timm9
Grm4
Ubap2l
Adcy5
Stmn1
Trmt2a
Snhg3
Myo5b
Mydgf

Phldb1
Ahcyl2
Arhgef2
Tmem126a
Sptbn4
Kif21b
Cdo1
Adam15
Ctxn1
Ndufa7
Hip1r
Mrps12
Panx2
Zmat5
Ptpro
Cinp
Rps6ka3
Ypel4
Mirg
Txndc16
Mir124a-1hg
Rps20
Adora2a
Cspg5
Fam210b
Ywhah
Rps5
Ephx1
Cyp51
Mcee
Pds5b
Ppp5c
Mog
Cdk4
Chn2
Tmem208
Carm1
Degs1
Fxyd2
Creg1
Fyco1
Large1
Cox19
Slc17a7
Ddit3
Hspe1
Pop7
Kcnk1
Luc7l
Dlx6os1
Tmem167
Fhl1

Rasgrp2
Mcf2l
Adarb1
Senp2
Aldh7a1
R3hdm4
Rpl24
Slco1c1
Dpf2
Tmod1
Ythdf1
Pdia6
Eml5
Ldah
Scg2
Zmym3
Polr3e
Tspan2
Kcnd3
Mobp
Dmac1
Sirt7
Plekhj1
Coq2
Cldn5
Ilkap
Nt5c3b
Mdk
Gadd45g
Chst2
Wfs1
Mmp17
Il18
Ube2f
Rell2
Dnajb11
Gap43
Lamp5
Rbm10
Trib2
Rpl27a
Zfp580
Rnf166
Ppp1r2
Mcts1
Lrrc10b
Ina
Dtx3
Rpp21
Egfl7
Otud4
Rhobtb2

Impdh1
Npy
Cyp46a1
Myo6
Shroom2
Anapc16
Rpl35a
Rpl8
Psmg4
Mrpl22
Rps19
Pgs1
Atp6v0a2
Pofut2
Dop1b
Cope
Ajap1
Coro2b
Ddx50
Ak3
Plcg1
Fgfr1op2
Pip4p1
Lage3
Cdk2ap2
Ndufa1
Cpsf1
Rprm
Med25
Gga3
Ppp6r3
3110039M20Rik
Elmo2
Rgs20
Ryr1
Eif4a3
Inha
Cdk16
Eloc
Olig1
Ptcd3
Mef2c
Snrpd3
Dgkd
Abhd14a
Fcor
Lrrc17
B9d2
Tram1l1
Adra2c
Rpl32
Gpr83

Tbc1d20
Med15
Ubp1
Pex6
Ahsa1
Mt2
Ttc4
Mrps18a
Pon2
Rps27rt
Ubr2
Rpsa
Ets2
Dclk2
Srm
Mrpl57
Rbis
Wipf3
Shisa4
Enah
Ccdc106
Rgs8
St3gal3
Capn2
Parp6
Cacna1c
Hs6st1
Ipo8
Adk
Akap9
Rpl36a
Mrtfa
Cops5
Reep2
Ssbp4
Plpbp
Rilpl1
Isyna1
Rida
Fndc5
Cfap410
Ino80e
Gm3764
Stk16
Clvs1
Aldh5a1
Rnf40
Ksr1
Dlg4
Traip
Xpo7
Tmem33

Tprkb
Vps4a
Smpdl3a
Tiam1
Mpi
Tmem126b
Cldn11
Dvl1
Clec2l
Ttc19
Klhl23
Igfbp7
Dpm2
Acy1
Mbnl2
Srrt
Lgi3
Psenen
Zcchc7
Psmg2
Nudt19
Nt5dc3
Pdia3
Abcc5
Eif2s3y
Dab1
Dnal4
Krt10
Rhoq
Stip1
Gpsm1
Adcy3
Cxxc1
Tmem185a
Mier2
Lrpprc
Map4k5
Smad3
Ankrd45
Surf4
Shisa7
Pdap1
Pcp4l1
Max
Bcas1
Fam120a
Tyro3
Ormdl3
Dnm2
Tars2
Dot1l
Orc4

Flna
Disp2
Sel1l
Zfr2
Rexo2
Gramd1b
Diras1
Foxo3
Prmt5
Cygb
Smox
Arih1
Tln1
Rnft2
Myh10
Tceal6
Smim27
Neat1
Ppp1r1b
Map1a
Speg
Prr7
Comt
Tmem70
Pcmtd1
Aup1
Ppp2r5a
Vrk1
Nicn1
Chid1
Tmem175
Chka
Chmp1a
Hivep2
Mpp3
Gng7
1500004A13Rik
Kcnab1
C1qb
Lratd1
Rapgefl1
Tmem234
Fbxw2
Rpl19
Cnih1
1110038F14Rik
Ppp1r13b
Il6st
Pde6d
Acap2
Ankrd46
Nubp2

Tenm1
Mrpl33
Fam193b
Gdpd1
Klhl13
Prpf8
Pip5k1c
Ramp2
Atad3a
Creld1
Rps19bp1
Magi2
Zfp511
Dagla
Mcm3ap
Med21
U2af2
Gfra4
Asic1
Mark1
Pigs
Cnot2
Usp32
Gnb2
Cmtm5
Sfxn3
Fnbp1l
Smg5
Cstf2
Ptcd2
Stub1
Lxn
Shank2
Slc25a23
Nae1
Isca2
Neu1
Limk2
Cxcl14
Nup50
Zfp871
Cbx7
Gramd1a
Syt3
Dlst
Nat8f1
Elovl5
Dnajc2
Dcaf7
Map7
Fam8a1
Vps13b

Kif17
Plpp1
Plppr3
Ints10
Rdh14
Slirp
Yipf3
Ppp6r1
Man1a2
Wasl
Rtf2
Gpr88
Jkamp
Rasgef1c
Selenos
Ndufaf5
Tango2
Stim2
Dlk2
Smim20
Baz1b
Gjb6
Czib
Ing4
Abhd17c
Comtd1
Chchd7
Opa1
Snrpa1
Anp32b
Rtn2
Trim46
Snx15
Hsph1
Mtx1
Begain
Sdr39u1
Ankrd10
Fam20c
Nr1h2
Dpm1
Krt12
Tsen15
Vcl
Abcb9
Cat
Cyb5r4
Timp3
Mocs2
Neto1
Immp1l
Car11

Sh3gl3
Psme2
Tmem223
Kansl3
Mlycd
Trak1
Ppp1cc
Mrpl19
Snapc5
Cdr1os
Rab4b
Stx8
Mrps36
Rubcn
Tomm5
Phf12
Hpcal1
Arhgap21
Cdc123
AU040320
Fads2
Ppp2r5b
Inpp1
Utrn
Polr2i
Htra1
Miga2
Inpp5j
Plxnb1
Ptma
Tsr3
Bcat1
Lrrc4b
Gtf3c2
Borcs6
Ydjc
Snx19
Timm10
Dhrs1
Naxd
Smim12
Wscd1
Scrt2
Sugt1
Ech1
Gba2
Cops2
Htr1b
Gm33651
Rap1a
Ssbp1
Tmem184b

Creld2
Ndufaf3
Kctd12
Ylpm1
Grin2a
Spsb3
Acap3
Rpl6
Pdzd2
Ppp1r37
Pik3c3
Actr1b
Ccndbp1
Syncrip
Ankrd13a
Smim26
Rtkn
Rraga
Nrbp2
Ablim1
Kifc2
Ccdc88c
Grik2
Ndufs1
Sec31a
Osbpl9
Trp53bp1
Gmpr2
Scrg1
Gnptg
Pbx2
Ss18l2
Erp29
Mrps11
Mir124-2hg
Srrd
Tmem240
Emc6
Pla2g7
Usp20
Mpv17l
Epb41l2
Nrbp1
Dctn3
Lgi2
Rrbp1
Letmd1
Fam110b
Mxd4
Dcun1d4
Med23
Tomm7

Dmtf1
Preb
Rala
Phf5a
Txnl1
Snx2
Ezr
Sucla2
Armc1
Tsc22d3
Uqcc3
Pdp1
Otud5
Zfp598
Hnrnpa2b1
Brwd1
Cuta
Phyhipl
Magoh
Lamtor3
Gapvd1
Cstf3
Dph3
Tomm34
Map4k2
Ranbp9
Rem2
Arf2
Leprot
Uchl3
Tpgs2
Pccb
Tmem258
Eif2b5
Pcdh9
Spire1
Tmem91
Smim18
Rab36
Hnrnpm
Dock10
Fn3k
Slc12a6
Eif4g3
Epb41l4aos
Pet100
Tra2a
Ncor2
Lmo7
Drd2
Zfp664
Lrrc45

Endog
Noc2l
Lars2
Rmdn3
Wdr83os
Pdzd11
Fcho1
Pno1
Kirrel3
Flot2
Zfp445
Zfp207
Csf1r
Cnot7
Btg3
Apoo
Med8
Exosc10
Asb13
Secisbp2l
Gm27032
Cnp
Pclo
Amph
Sec61b
Fut8
Cenpx
Ubac1
Lrrc42
Gt(ROSA)26Sor
Itgb1bp1
Jagn1
Eef1akmt1
Fam3c
Rin1
Stxbp6
Tssc4
Arhgef18
Slc6a11
Tspan9
Lrrtm3
Ttll7
Sacm1l
Emc8
Slain1
Rtl8a
Bap1
Tiam2
Mrpl46
Ryr3
Spata2l
Ndufaf2

Trim41
Kpna6
Slc50a1
Mrps14
Rprml
Cop1
Gtpbp6
Mctp1
Tbce
Spns1
Scn3b
Rpl10a
Trappc5
Tmem68
Gsk3a
Slc2a3
Entpd6
Gpr162
Smap2
Ccdc167
Cept1
Fbxo7
2310061I04Rik
Lysmd2
Rbm28
Bloc1s6
Mdp1
Rnf25
Ccser2
Tufm
B9d1
Tm2d2
Impdh2
Rcan1
Lrrfip1
Lyrm2
Suclg1
Vps52
Rbfa
Rimbp2
Osbpl8
Josd1
Ift22
Crabp1
Snn
Paip2b
Scamp3
Surf2
Hnrnph3
Atrx
Ubr4
Coa6

Abcd3
Ccdc97
Mb21d2
Zfp532
Sh3bgrl
Fam91a1
Rnmt
Prkab2
Srsf1
Cend1
Pnisr
Fem1b
L1cam
Serp1
Rab5c
Mark2
Ccng1
Tpp1
Kpnb1
Slc38a3
Lrrk2
Cog4
Sdhaf4
C1qtnf12
Emc4
Erbin
Nme7
Cntnap2
Armcx1
Cnot8
Ephx4
Nacc2
Stx16
Unc5a
Wac
Adam11
Csnk2a2
Ppil2
Ttll1
Dhrs7b
Cacna1b
Wtap
Leng8
Zfp385a
Dtnbp1
Mrps25
Timm29
Gga1
Ccs
Tyrobp
Zranb2
Iffo1

Kat7
Dcun1d5
Pop4
Gnb1
Chchd5
Col6a1
Rgs9
Gm10419
Homer1
Aldh1l1
Ptp4a3
Atat1
Gdpd5
Mapk6
Gmpr
Rapgef6
Herc2
Nrsn1
Cdk19
Ap3m1
Asrgl1
Mlec
Rab5if
Mrps6
Zcchc17
Ppp1r14a
Dhrs7
Rer1
Izumo4
Aip
C1qtnf4
Chrac1
Dnaja4
Usp33
Gsg1l
Dld
Gsto1
Mfap3l
Rnf181
Tmed3
Gbf1
Plppr4
Borcs8
Tmem14a
Jtb
Znhit3
Rasgef1b
Mthfsl
Wdr45
Sgcb
Hsd17b12
Kcnip4

Ramp1
Yeats4
Phyh
Dynll2
Slc25a12
Mrpl36
Ciao2b
Brd9
Fuom
2410002F23Rik
Tnks2
Wdr45b
Bax
Fam163b
Tmem38a
Cisd2
Nfyc
Tmem214
Mrpl15
Iah1
Acat1
Dnajc18
2010204K13Rik
Pnn
Mdm2
Hspbp1
Mrps17
Dtna
Trim3
Dnajb2
Dmac2
Pnpo
Isca1
Snd1
Gpr158
Chmp2b
Sp3os
Dpy30
Selenop
Nfasc
Ttbk1
Cox20
Snw1
Cacnb1
Vrk3
Foxk2
Churc1
Pim2
Pllp
Dpm3
Slc27a4
Robo2

Nr2c2
Cadm2
Ulk2
Parp1
Gng11
Vps29
Mrpl40
Med10
Copz1
Tafa5
Itch
Lamtor5
Akap5
Zpr1
Calcoco1
Bpgm
Alcam
Tmem201
Lman2
Txlna
Wdr13
Unc80
Kmt2e
Snx32
Apex1
Manf
Ube2i
Pstk
Fam107a
Ppm1b
Mtus1
Tmem127
Cnot6
BC004004
Sprn
Vps9d1
Trappc1
Tlk2
Tmbim4
Ube2h
Ccdc107
Pum1
Atp6v0e
Pin4
Paqr9
Men1
Pnrc1
Arfgef1
Dzip3
Rab24
Pkia
Clasrp

Mms19
Rgs17
Dhdds
Cyth2
Slc1a1
Hdgfl2
Dpysl4
Abca3
Txn1
Pdhb
Bcr
Atg3
Paqr4
Akap8l
Ndfip2
Rrn3
Ncbp2
Cacna1e
Mrps18b
Hectd1
Gtf2h5
Higd1a
Tpst2
Map9
C1qc
Ehd3
Capzb
Ptrhd1
Ccnh
Zmynd8
Sap30bp
Matk
Cep19
Cog1
Src
Cox14
Ppm1f
Itm2a
Api5
Zhx1
Myl12a
Myo9b
Ppip5k1
Tia1
Acadvl
Slc35e1
Ola1
Cebpzos
Med14
Emg1
Acp2
Lrtm2

1110059G10Rik
Ypel5
Agpat3
Fem1a
Prpf31
Srrm3
Sptan1
Mrpl16
Inpp5a
Ckap5
Agpat5
Cfap36
Ist1
Plxna2
Ugp2
Coa5
Gnb4
Washc2
Crbn
Hpcal4
Ppa2
Shfl
Ctss
Clip1
Kxd1
Abhd17b
Metrn
Kctd3
Hrh3
Impa1
Dgkh
Gng5
Ewsr1
Rfng
Smim19
Naa30
Ric3
Colgalt1
Pak6
Zfyve27
Hspa12a
Cops4
Car12
Psmd14
Hdac6
Ttc1
Ppp1r12a
Rbfox3
Aph1a
Ly6e
Tubg2
Rsrc2

Tax1bp1
Nprl2
Tmem183a
Osgep
Trap1
Cog7
Chd8
Etv5
Uba5
Tmem219
Ablim2
Mmd2
Dhx36
Bend6
Pbrm1
Mob4
Cst6
Smpd4
Adh5
Tesc
Rpl10
Cfap298
H13
Crat
Lst1
Nap1l5
Mrpl44
Mink1
Mrps9
Cacfd1
Wnk1
Tbc1d14
Ano8
Bptf
Mrpl43
Btbd1
Ndrg2
Lemd2
Cyld
Arpc5l
Asic4
Slc25a19
Pip5k1a
Stk39
Tmem60
Fam162a
Ddhd1
Nop56
Mrpl14
Mpp2
Med9
Vldlr

Rabac1
Exoc4
Polg
Mrpl18
Rnf34
Dag1
Rims2
Ndufaf7
Spock3
Prickle2
Ccnl2
Prelid3b
Mgrn1
Klhl22
Pnma2
Nfu1
Camk2d
Rbck1
Acd
Mrps30
Epb41l3
Atp13a2

Striatum cTBS

DEGs Young sham vs Aged sham	cTBS DEGs unique to young adults	cTBS DEGs unique to young adults that are also ageing DEGs
Bc1	Lrrc17	Acyp2
Cox8a	Rps28	Ahcyl2
Actb	Ndufa1	Arpp21
Rps24	Pde10a	Bloc1s1
Malat1	Tomm7	Chst2
Rpl4	Ost4	Churc1
Sst	Snhg9	Comt
Fau	Tspyl4	Cox8a
Dpysl2	Araf	Cpe
Pcp4	Basp1	Cyp46a1
Mt1	Snap25	Ddn
Rpl3	Snrpf	Dlg4
Rpl5	Cox7c	Dpysl2
Bloc1s1	Rps27	Elmo2
Rasgrf2	Bloc1s1	Ewsr1
Snca	Cox17	Fam120a
Rpl37	Atp5md	Fau
Rpl13	Sem1	Fgfr1op2
Eef1a1	Isca1	Hspa5
Ly6c1	Dmtn	Isca1
Usp50	Snca	Itm2c
Nts	Ddn	Lrrc17
Gamt	Smim26	Mbp
Otof	Scn4b	Mt1
Rpl14	Ndrg4	Mt2
Rps10	Snhg6	Ndfip2
Ntsr2	Rps29	Ndrg4
Hspa5	Phpt1	Ndufa1
Snhg6	Slc6a1	Ost4
Ddn	Actr2	Pdia3
Lingo3	Tatdn1	Pet100
Cpe	Arpp21	Pfdn5
Itm2c	Naa20	Phyhipl
Rps8	Ndufa3	Plppr4
Ptgds	Wsb2	Ppp1r2
Uba52	Atp5mpl	Psd
Cacna1h	Car2	R3hdm4
Calm1	Sdhd	Rap1a
Tsc22d1	Ewsr1	Rasd2
Them6	Snhg8	Rasgrp2
Atp1b1	Pacsin1	Rbis
Polr2l	Atp1a3	Rpl10
Ndrg4	Plppr4	Rpl13
Cox7b	Sst	Rpl13a
Rpl13a	Fus	Rpl14
Rps2	Srsf5	Rpl24
Psd	App	Rpl27a
Rpl9	Cdk5r2	Rpl32
Polb	Cops7a	Rpl4

Kcnip2
Strip2
Snhg11
Rps7
Josd2
Ankrd13b
Ndufa4
Nefm
Unc13c
Tac1
Pfdn5
Polr2j
Mbp
Snhg20
Necab1
Cck
Pacsin2
Arhgap33
Maneal
Mal
Higd2a
Coro7
Ccm2
Zdhhc14
Trak2
Dalrd3
Rps3a1
Jsrp1
Arpp21
Lrpap1
Camk1g
Snhg1
Srsf11
Mrpl41
Pitpnm2
Eef1a2
Gucy1a1
Sdhaf2
Chordc1
Rac3
Acvr2a
Rpl9-ps6
Rnf20
Srp72
Smpd3
Rps14
Yipf1
Morf4l1
Acsbg1
Rps27a
Mapk1ip1
Ppid

Tmem258
Rpl39
Huwe1
Txn1
Spag9
Acyp2
1810037I17Rik
Cntnap1
Rnf5
Sparcl1
Mbp
Rps27rt
Rap1gds1
Chmp5
Mt2
Slc25a22
Kctd13
Ppp1r16b
R3hdm4
Rab3c
Mt1
Ppp1r9b
Agap2
Gucy1b1
Gng13
Rgs7bp
Selenof
Kif5a
Cox8a
Rasd2
Meg3
Rpl9-ps6
Jph4
Atp1b2
Sec61g
Atp6v1a
Cs
Snrpg
Camk2a
Rheb
Lzts3
Ndufb3
Tsc22d4
Comt
Cadm4
Atxn2l
Pet100
Zwint
Arpc4
Rcn2
Sipa1l1
Atp5k

Rpl5
Rpl8
Rpl9-ps6
Rps10
Rps14
Rps19
Rps2
Rps20
Rps24
Rps27a
Rps27rt
Rps3a1
Rps4x
Rps5
Rps7
Rps8
Rpsa
Rraga
Scn4b
Smim26
Snca
Snhg11
Snhg3
Snhg6
Sst
Tmem258
Tomm7
Txn1
Usp50

Oga	Usp50
Sirt2	Stxbp1
Mat2a	Cpe
Pfn1	Snhg11
Btbd3	Cox6c
Agrn	Churc1
Elp2	Senp3
Ralgds	Glul
Sirt3	Abat
Scn4b	Chst2
Limd2	Meis2
Mrpl21	Paip2
Slc2a1	Hypk
Emc2	Ndfip2
Lyst	Rapgef4
Ly6a	Mapre2
Rps4x	Fbxl16
Epas1	Ppp1r2
Taf13	Dlg4
Tmem230	Cfap20
Ost4	Prrc2a
Kansl2	Eif4a2
Amz2	Snhg3
Filip1	Mtmr9
Arel1	Sdcbp
Slc35d3	Cacna2d3
Rgl2	Fam120a
Acyp2	Rgs14
Tmub2	Rab28
Rpl30	Ttc7b
Nedd4l	Rasgrp2
Tubb5	Ikbkb
Tbc1d9b	Ahcyl2
Pttg1ip	Add2
Prxl2a	Hectd4
Brinp1	B2m
Nlgn1	Dbndd2
Trmt1	Hprt
Cbx4	Gpm6b
Inf2	Mrps28
Rasd2	Erh
Pvalb	Atl1
Ccdc28b	Elmo2
Timm9	Phyhip
Grm4	Trrap
Ubap2l	Rbis
Adcy5	Arhgap39
Stmn1	Phyhipl
Trmt2a	Mrpl27
Snhg3	Pdia3
Myo5b	Dnajb4
Mydgf	Hspa5

Phldb1	Cd302
Ahcyl2	Atn1
Arhgef2	Fgfr1op2
Tmem126a	Psd
Sptbn4	Acsl3
Kif21b	Rraga
Cdo1	Wdr89
Adam15	Cyp46a1
Ctxn1	Polr2k
Ndufa7	Atp5g3
Hip1r	Miga1
Mrps12	Rap1a
Panx2	Gas5
Zmat5	Srrm2
Ptpro	Slc4a4
Cinp	Ank
Rps6ka3	Cstb
Ypel4	Rpl18
Mirg	Rps3
Txndc16	Rps4x
Mir124a-1hg	Rps3a1
Rps20	Eef1b2
Adora2a	Syt11
Cspg5	Rpl21
Fam210b	Dpysl2
Ywhah	Rpsa
Rps5	Rpl23a
Ephx1	Rpl24
Cyp51	Rpl27a
Mcee	Rps7
Pds5b	Rpl11
Ppp5c	Rps15
Mog	Rps27a
Cdk4	Ttr
Chn2	Rpl8
Tmem208	Nenf
Carm1	Rps19
Degs1	Rplp1
Fxyd2	Rpl32
Creg1	Itm2c
Fyco1	Fau
Large1	Rpl5
Cox19	Rpl18a
Slc17a7	Rps20
Ddit3	Eno1
Hspe1	Rps5
Pop7	Rpl17
Kcnk1	Rps2
Luc7l	Rps12
Dlx6os1	Pfdn5
Tmem167	Rps10
Fhl1	Rps11

Rasgrp2
Mcf2l
Adarb1
Senp2
Aldh7a1
R3hdm4
Rpl24
Slco1c1
Dpf2
Tmod1
Ythdf1
Pdia6
Eml5
Ldah
Scg2
Zmym3
Polr3e
Tspan2
Kcnd3
Mobp
Dmac1
Sirt7
Plekhj1
Coq2
Cldn5
Ilkap
Nt5c3b
Mdk
Gadd45g
Chst2
Wfs1
Mmp17
Il18
Ube2f
Rell2
Dnajb11
Gap43
Lamp5
Rbm10
Trib2
Rpl27a
Zfp580
Rnf166
Ppp1r2
Mcts1
Lrrc10b
Ina
Dtx3
Rpp21
Egfl7
Otud4
Rhobtb2

Rps14
Rpl13
Rpl4
Atp9a
Rpl26
Rps8
Rpl13a
Rps24
Rpl14
Hbb-bs
Rpl10

Impdh1
Npy
Cyp46a1
Myo6
Shroom2
Anapc16
Rpl35a
Rpl8
Psmg4
Mrpl22
Rps19
Pgs1
Atp6v0a2
Pofut2
Dop1b
Cope
Ajap1
Coro2b
Ddx50
Ak3
Plcg1
Fgfr1op2
Pip4p1
Lage3
Cdk2ap2
Ndufa1
Cpsf1
Rprm
Med25
Gga3
Ppp6r3
3110039M20Rik
Elmo2
Rgs20
Ryr1
Eif4a3
Inha
Cdk16
Eloc
Olig1
Ptcd3
Mef2c
Snrpd3
Dgkd
Abhd14a
Fcor
Lrrc17
B9d2
Tram1l1
Adra2c
Rpl32
Gpr83

Tbc1d20
Med15
Ubp1
Pex6
Ahsa1
Mt2
Ttc4
Mrps18a
Pon2
Rps27rt
Ubr2
Rpsa
Ets2
Dclk2
Srm
Mrpl57
Rbis
Wipf3
Shisa4
Enah
Ccdc106
Rgs8
St3gal3
Capn2
Parp6
Cacna1c
Hs6st1
Ipo8
Adk
Akap9
Rpl36a
Mrtfa
Cops5
Reep2
Ssbp4
Plpbp
Rilpl1
Isyna1
Rida
Fndc5
Cfap410
Ino80e
Gm3764
Stk16
Clvs1
Aldh5a1
Rnf40
Ksr1
Dlg4
Traip
Xpo7
Tmem33

Tprkb
Vps4a
Smpdl3a
Tiam1
Mpi
Tmem126b
Cldn11
Dvl1
Clec2l
Ttc19
Klhl23
Igfbp7
Dpm2
Acy1
Mbnl2
Srrt
Lgi3
Psenen
Zcchc7
Psmg2
Nudt19
Nt5dc3
Pdia3
Abcc5
Eif2s3y
Dab1
Dnal4
Krt10
Rhoq
Stip1
Gpsm1
Adcy3
Cxxc1
Tmem185a
Mier2
Lrpprc
Map4k5
Smad3
Ankrd45
Surf4
Shisa7
Pdap1
Pcp4l1
Max
Bcas1
Fam120a
Tyro3
Ormdl3
Dnm2
Tars2
Dot1l
Orc4

Flna
Disp2
Sel1l
Zfr2
Rexo2
Gramd1b
Diras1
Foxo3
Prmt5
Cygb
Smox
Arih1
Tln1
Rnft2
Myh10
Tceal6
Smim27
Neat1
Ppp1r1b
Map1a
Speg
Prr7
Comt
Tmem70
Pcmtd1
Aup1
Ppp2r5a
Vrk1
Nicn1
Chid1
Tmem175
Chka
Chmp1a
Hivep2
Mpp3
Gng7
1500004A13Rik
Kcnab1
C1qb
Lratd1
Rapgefl1
Tmem234
Fbxw2
Rpl19
Cnih1
1110038F14Rik
Ppp1r13b
Il6st
Pde6d
Acap2
Ankrd46
Nubp2

Tenm1
Mrpl33
Fam193b
Gdpd1
Klhl13
Prpf8
Pip5k1c
Ramp2
Atad3a
Creld1
Rps19bp1
Magi2
Zfp511
Dagla
Mcm3ap
Med21
U2af2
Gfra4
Asic1
Mark1
Pigs
Cnot2
Usp32
Gnb2
Cmtm5
Sfxn3
Fnbp1l
Smg5
Cstf2
Ptcd2
Stub1
Lxn
Shank2
Slc25a23
Nae1
Isca2
Neu1
Limk2
Cxcl14
Nup50
Zfp871
Cbx7
Gramd1a
Syt3
Dlst
Nat8f1
Elovl5
Dnajc2
Dcaf7
Map7
Fam8a1
Vps13b

Kif17
Plpp1
Plppr3
Ints10
Rdh14
Slirp
Yipf3
Ppp6r1
Man1a2
Wasl
Rtf2
Gpr88
Jkamp
Rasgef1c
Selenos
Ndufaf5
Tango2
Stim2
Dlk2
Smim20
Baz1b
Gjb6
Czib
Ing4
Abhd17c
Comtd1
Chchd7
Opa1
Snrpa1
Anp32b
Rtn2
Trim46
Snx15
Hsph1
Mtx1
Begain
Sdr39u1
Ankrd10
Fam20c
Nr1h2
Dpm1
Krt12
Tsen15
Vcl
Abcb9
Cat
Cyb5r4
Timp3
Mocs2
Neto1
Immp1l
Car11

Sh3gl3
Psme2
Tmem223
Kansl3
Mlycd
Trak1
Ppp1cc
Mrpl19
Snapc5
Cdr1os
Rab4b
Stx8
Mrps36
Rubcn
Tomm5
Phf12
Hpcal1
Arhgap21
Cdc123
AU040320
Fads2
Ppp2r5b
Inpp1
Utrn
Polr2i
Htra1
Miga2
Inpp5j
Plxnb1
Ptma
Tsr3
Bcat1
Lrrc4b
Gtf3c2
Borcs6
Ydjc
Snx19
Timm10
Dhrs1
Naxd
Smim12
Wscd1
Scrt2
Sugt1
Ech1
Gba2
Cops2
Htr1b
Gm33651
Rap1a
Ssbp1
Tmem184b

Creld2
Ndufaf3
Kctd12
Ylpm1
Grin2a
Spsb3
Acap3
Rpl6
Pdzd2
Ppp1r37
Pik3c3
Actr1b
Ccndbp1
Syncrip
Ankrd13a
Smim26
Rtkn
Rraga
Nrbp2
Ablim1
Kifc2
Ccdc88c
Grik2
Ndufs1
Sec31a
Osbpl9
Trp53bp1
Gmpr2
Scrg1
Gnptg
Pbx2
Ss18l2
Erp29
Mrps11
Mir124-2hg
Srrd
Tmem240
Emc6
Pla2g7
Usp20
Mpv17l
Epb41l2
Nrbp1
Dctn3
Lgi2
Rrbp1
Letmd1
Fam110b
Mxd4
Dcun1d4
Med23
Tomm7

Dmtf1
Preb
Rala
Phf5a
Txnl1
Snx2
Ezr
Sucla2
Armc1
Tsc22d3
Uqcc3
Pdp1
Otud5
Zfp598
Hnrnpa2b1
Brwd1
Cuta
Phyhipl
Magoh
Lamtor3
Gapvd1
Cstf3
Dph3
Tomm34
Map4k2
Ranbp9
Rem2
Arf2
Leprot
Uchl3
Tpgs2
Pccb
Tmem258
Eif2b5
Pcdh9
Spire1
Tmem91
Smim18
Rab36
Hnrnpm
Dock10
Fn3k
Slc12a6
Eif4g3
Epb41l4aos
Pet100
Tra2a
Ncor2
Lmo7
Drd2
Zfp664
Lrrc45

Endog
Noc2l
Lars2
Rmdn3
Wdr83os
Pdzd11
Fcho1
Pno1
Kirrel3
Flot2
Zfp445
Zfp207
Csf1r
Cnot7
Btg3
Apoo
Med8
Exosc10
Asb13
Secisbp2l
Gm27032
Cnp
Pclo
Amph
Sec61b
Fut8
Cenpx
Ubac1
Lrrc42
Gt(ROSA)26Sor
Itgb1bp1
Jagn1
Eef1akmt1
Fam3c
Rin1
Stxbp6
Tssc4
Arhgef18
Slc6a11
Tspan9
Lrrtm3
Ttll7
Sacm1l
Emc8
Slain1
Rtl8a
Bap1
Tiam2
Mrpl46
Ryr3
Spata2l
Ndufaf2

Trim41
Kpna6
Slc50a1
Mrps14
Rprml
Cop1
Gtpbp6
Mctp1
Tbce
Spns1
Scn3b
Rpl10a
Trappc5
Tmem68
Gsk3a
Slc2a3
Entpd6
Gpr162
Smap2
Ccdc167
Cept1
Fbxo7
2310061I04Rik
Lysmd2
Rbm28
Bloc1s6
Mdp1
Rnf25
Ccser2
Tufm
B9d1
Tm2d2
Impdh2
Rcan1
Lrrfip1
Lyrm2
Suclg1
Vps52
Rbfa
Rimbp2
Osbpl8
Josd1
Ift22
Crabp1
Snn
Paip2b
Scamp3
Surf2
Hnrnph3
Atrx
Ubr4
Coa6

Abcd3
Ccdc97
Mb21d2
Zfp532
Sh3bgrl
Fam91a1
Rnmt
Prkab2
Srsf1
Cend1
Pnisr
Fem1b
L1cam
Serp1
Rab5c
Mark2
Ccng1
Tpp1
Kpnb1
Slc38a3
Lrrk2
Cog4
Sdhaf4
C1qtnf12
Emc4
Erbin
Nme7
Cntnap2
Armcx1
Cnot8
Ephx4
Nacc2
Stx16
Unc5a
Wac
Adam11
Csnk2a2
Ppil2
Ttll1
Dhrs7b
Cacna1b
Wtap
Leng8
Zfp385a
Dtnbp1
Mrps25
Timm29
Gga1
Ccs
Tyrobp
Zranb2
Iffo1

Kat7
Dcun1d5
Pop4
Gnb1
Chchd5
Col6a1
Rgs9
Gm10419
Homer1
Aldh1l1
Ptp4a3
Atat1
Gdpd5
Mapk6
Gmpr
Rapgef6
Herc2
Nrsn1
Cdk19
Ap3m1
Asrgl1
Mlec
Rab5if
Mrps6
Zcchc17
Ppp1r14a
Dhrs7
Rer1
Izumo4
Aip
C1qtnf4
Chrac1
Dnaja4
Usp33
Gsg1l
Dld
Gsto1
Mfap3l
Rnf181
Tmed3
Gbf1
Plppr4
Borcs8
Tmem14a
Jtb
Znhit3
Rasgef1b
Mthfsl
Wdr45
Sgcb
Hsd17b12
Kcnip4

Ramp1
Yeats4
Phyh
Dynll2
Slc25a12
Mrpl36
Ciao2b
Brd9
Fuom
2410002F23Rik
Tnks2
Wdr45b
Bax
Fam163b
Tmem38a
Cisd2
Nfyc
Tmem214
Mrpl15
Iah1
Acat1
Dnajc18
2010204K13Rik
Pnn
Mdm2
Hspbp1
Mrps17
Dtna
Trim3
Dnajb2
Dmac2
Pnpo
Isca1
Snd1
Gpr158
Chmp2b
Sp3os
Dpy30
Selenop
Nfasc
Ttbk1
Cox20
Snw1
Cacnb1
Vrk3
Foxk2
Churc1
Pim2
Pllp
Dpm3
Slc27a4
Robo2

Nr2c2
Cadm2
Ulk2
Parp1
Gng11
Vps29
Mrpl40
Med10
Copz1
Tafa5
Itch
Lamtor5
Akap5
Zpr1
Calcoco1
Bpgm
Alcam
Tmem201
Lman2
Txlna
Wdr13
Unc80
Kmt2e
Snx32
Apex1
Manf
Ube2i
Pstk
Fam107a
Ppm1b
Mtus1
Tmem127
Cnot6
BC004004
Sprn
Vps9d1
Trappc1
Tlk2
Tmbim4
Ube2h
Ccdc107
Pum1
Atp6v0e
Pin4
Paqr9
Men1
Pnrc1
Arfgef1
Dzip3
Rab24
Pkia
Clasrp

Mms19
Rgs17
Dhdds
Cyth2
Slc1a1
Hdgfl2
Dpysl4
Abca3
Txn1
Pdhb
Bcr
Atg3
Paqr4
Akap8l
Ndfip2
Rrn3
Ncbp2
Cacna1e
Mrps18b
Hectd1
Gtf2h5
Higd1a
Tpst2
Map9
C1qc
Ehd3
Capzb
Ptrhd1
Ccnh
Zmynd8
Sap30bp
Matk
Cep19
Cog1
Src
Cox14
Ppm1f
Itm2a
Api5
Zhx1
Myl12a
Myo9b
Ppip5k1
Tia1
Acadvl
Slc35e1
Ola1
Cebpzos
Med14
Emg1
Acp2
Lrtm2

1110059G10Rik
Ypel5
Agpat3
Fem1a
Prpf31
Srrm3
Sptan1
Mrpl16
Inpp5a
Ckap5
Agpat5
Cfap36
Ist1
Plxna2
Ugp2
Coa5
Gnb4
Washc2
Crbn
Hpcal4
Ppa2
Shfl
Ctss
Clip1
Kxd1
Abhd17b
Metrn
Kctd3
Hrh3
Impa1
Dgkh
Gng5
Ewsr1
Rfng
Smim19
Naa30
Ric3
Colgalt1
Pak6
Zfyve27
Hspa12a
Cops4
Car12
Psmd14
Hdac6
Ttc1
Ppp1r12a
Rbfox3
Aph1a
Ly6e
Tubg2
Rsrc2

Tax1bp1
Nprl2
Tmem183a
Osgep
Trap1
Cog7
Chd8
Etv5
Uba5
Tmem219
Ablim2
Mmd2
Dhx36
Bend6
Pbrm1
Mob4
Cst6
Smpd4
Adh5
Tesc
Rpl10
Cfap298
H13
Crat
Lst1
Nap1l5
Mrpl44
Mink1
Mrps9
Cacfd1
Wnk1
Tbc1d14
Ano8
Bptf
Mrpl43
Btbd1
Ndrg2
Lemd2
Cyld
Arpc5l
Asic4
Slc25a19
Pip5k1a
Stk39
Tmem60
Fam162a
Ddhd1
Nop56
Mrpl14
Mpp2
Med9
Vldlr

Rabac1
Exoc4
Polg
Mrpl18
Rnf34
Dag1
Rims2
Ndufaf7
Spock3
Prickle2
Ccnl2
Prelid3b
Mgrn1
Klhl22
Pnma2
Nfu1
Camk2d
Rbck1
Acd
Mrps30
Epb41l3
Atp13a2

WMT iTBS

DEGs Young sham vs Aged sham	iTBS DEGs unique to young adults	iTBS DEGs unique to young adults that are also ageing DEGs
Mobp	Penk	AI593442
Mbp	Rgs9	Apod
Uba52	Pde1b	Calb1
Cabp1	Calb1	Cd82
Plekhb1	Gnal	Cldn11
Bcas1	Pcp4	Csrp1
Chn1	Ppp1r1b	Desi1
Hpca	AI593442	Fbxl16
Scn1b	Ptpn5	Gnal
Bsn	Zwint	Gng11
Slc17a7	Fbxl16	Kcnab1
Tesc	Tac1	Lrrc17
Lingo1	Prrc2b	Mag
Ppp3r1	Kcnab1	Metrn
Nrgn	Rap1gap	Nkx6-2
Nptxr	Nedd4	Opalin
Mef2c	Gng11	Ppp1r14a
Rpl9-ps6	Aplp1	Prrc2b
Phf24	Cdc42ep1	Ptgds
Eef1a2	Opalin	Ptpn5
Sv2b	Abca2	Rgs9
Gria3	Cldn11	Rhog
Cacnb3	Rhog	Tac1
Lamp5	Metrn	Tatdn1
Pvalb	Csrp1	Trf
Cadm3	Cd82	
Mal	Apod	
Mt1	Desi1	
Dnm1	Gjb1	
Rprml	Nkx6-2	
Camkk2	Ppp1r14a	
Plk2	Lgi3	
Pi4ka	Cnot3	
Trp53inp2	Trf	
Atp1a1	Mag	
Fabp3	Gm2000	
Itpka	Tubb4a	
Tceal5	Tatdn1	
Nat8l	Ptgds	
Prr18	Lrrc17	
Tmeff2		
Tmsb4x		
Tceal6		
Bloc1s1		
Kcnab2		
Ptk2b		
Pdlim2		
Pdp1		
Matk		

Lmo4
Clic4
Camk2b
Ppp3ca
Sri
Cnp
Rps29
Stx1a
Lin7b
Svop
Brinp1
Mmp17
Grin1
Kalrn
Snhg6
Cplx1
Rnf112
Pgm2l1
Calm1
Tspan2
Thy1
Spock1
Nell2
Gpr162
Cx3cl1
Pea15a
Fam131a
Cdk5r1
Ptprz1
Egr1
Golga7
Polr3e
Rell2
Cap2
Fxyd1
Snap25
Atp2b2
Pkm
Syn1
Kcnip2
Trak2
Habp4
Arf3
Snph
Myh10
Celf2
Marcksl1
Mrtfb
Slc6a17
Evi2a
Rbfox3
Itpr1

Tppp3
Syp
Tmem132a
Scrg1
Camk2a
Cyfip2
R3hdm1
Ier5
Prkcg
Cplx2
Cck
St3gal5
Rasgef1a
Gnb5
Calb1
Carhsp1
Snhg9
Zbtb18
Nrxn3
Fa2h
Gls
Sccpdh
Lynx1
Gabrg2
Car11
Ncs1
Myo6
Gamt
Cacng3
Bcat1
Phgdh
Baiap2
Rab31
Plcb1
Ak3
Pianp
Jph3
Reep2
AI593442
Ugt8a
Tns3
Adcy1
Tac1
Serpini1
Nsf
Tmem59l
Mog
Josd2
Myt1l
Grina
Madd
Timm22

Gapdh
Pcsk2
Snx10
Napb
Mapk10
Tpi1
Sncb
Litaf
Ywhah
Dgkz
Dbi
Grik5
Tbc1d9b
Tatdn1
Tmeff1
Cpe
Icam5
Gng12
Snca
BC004004
Ddah1
Cmtm5
Rgs9
Rasgrp1
Syngr3
Nfe2l1
Rps28
Dcaf17
Setd7
Ptprn
Igfbp5
Peg13
St8sia3
Grb14
Cdk2ap1
Cldn11
Tusc3
Gabrb3
Ckmt1
Gnal
Paqr8
Tspan13
Slc2a1
Syt4
Qdpr
Dmtn
Sh3gl3
Ugp2
Ppp1r9a
Dbn1
Cat
Atp6v0e

Phyhip
Cdc37l1
Rnf157
Aak1
Mgst3
Lamp1
Aktip
Tsnax
Arl6ip5
Dock10
Gabbr2
Kcnj4
Rps27
Arhgef9
Ptpn11
Vbp1
Rab15
Ncor2
Gnai1
Tmem47
Ewsr1
Enc1
Ptpn5
Unc50
Eno2
Nkiras1
Id3
Pip5k1c
Tra2a
Fhl1
Myo5a
Aqp4
Cd9
Neurl1a
Reps2
Rasl10b
Stmn3
Anp32b
Efhd2
Bex2
Ppp2r5a
Rusc1
Cadps
Cfap36
2310022B05Rik
Scg2
Rab3a
Ivns1abp
Dlgap1
Syt13
Rpl4
Mt2

Tnks2
Oip5os1
Rasd2
Homer1
Strn4
Ywhaz
Dock3
Tmco1
Nefm
Tubb5
Tmem179
Evl
Fyn
Kcnq2
Rab6b
Dbp
Chga
Tuba4a
Plekhj1
Zfas1
Clstn3
Ednrb
Syn2
Add2
Dlgap4
Gsn
Kndc1
Pja2
Got2
Celf5
Cryab
Serinc5
Ddit3
Daam2
Rhog
Cadm2
Shtn1
Dgkb
Vamp2
Churc1
Rps12
Lrrn1
Mapk9
Sar1b
Adgrb2
Prkcb
Map4k4
Unc13a
Adgrl1
Kcnk1
Atp6v1b2
Zfand5

Pip4k2a
Otub1
Scd1
Asphd2
Sptbn2
Egr3
Fgf1
Idh3a
Fads2
Enah
Cnn3
Atxn7l3
B3gat3
Usp14
Arrdc3
Stk25
Scg5
Aamdc
Camta2
Scn2a
Cntn2
Sorl1
Anapc2
Nptn
Rdx
Nsg2
Wnk1
Stxbp1
Dkk3
Sc5d
C1qtnf4
Gal3st1
Ap1b1
Cyb5b
Fam234b
Zfp664
Ptprd
Kctd17
Got1
Dclk1
Prkce
Tcf4
Actr1b
Rnf130
Meg3
Atp6v0c
Picalm
Clstn1
Gpr88
Rad21
Elovl5
Adgrb1

Rian
Chst1
Nat14
Ralgds
Rps27l
Astn1
B230219D22Rik
Wasf1
Ogdh
Epb41l2
Pltp
Syt5
Fxyd7
Abhd4
Txndc9
Necap1
Ankrd13a
Plekhh1
Ndrg1
Ado
Snhg11
Plp1
Aspa
Med21
Smim13
Cdc42se2
Arhgap23
Malat1
Chn2
Ddr1
Taf13
Eif6
Mllt11
Nrbp2
Ctnna1
Tprkb
Rnf227
Olig2
Pdxdc1
Ap2a2
Strn
Nrcam
S1pr5
Vgf
Mmd
Cd81
Pou3f3
Sirt2
Rph3a
Nefl
Olig1
Cd164

Plekha1
Cdk17
Gstm7
Myl12b
Scarb2
Nhp2
Srpk2
Srsf11
Ppp2r3a
Efhd1
Gjc2
Snn
Ptk2
Pdhb
Mras
Jam3
Hepacam
Hadha
Atp6v1e1
Nacc2
Gltp
Sh2d5
Micall1
Snap91
Arpc1b
Khdrbs3
Pop5
Slc48a1
Mrpl17
Arhgdig
Fdx2
Snapin
Gabra1
Pam16
Eno1
Ufm1
Rnps1
Rtkn
Haghl
Timm23
Arpp21
Tmem134
Igfbp6
Smap2
Mrpl4
Zcchc24
Mrps26
Eci1
Adcyap1r1
Cers2
Strbp
Arhgap5

Tmem229a
Phf5a
Asns
Erbin
Ddx24
Pop4
Phlda3
Lrp1
Trim9
Pim3
Pik3r1
Pfkl
1700047M11Rik
Cxcl14
Gnptg
Slc44a1
Rpl37a
Ccdc124
Ina
Gjc3
Pdcd10
2900052N01Rik
Tusc2
Mat2b
Stard7
Dync1i1
Wscd1
Lrrc58
Kifc2
Rpl35
Sdcbp
Atp1b1
Usp5
Aldh6a1
Sptbn4
Anxa5
Adgrg1
Schip1
Ybx3
Fez1
Ifi27
Tmbim4
Ssbp4
Selenop
Hras
Trim37
Fbxl16
Rogdi
Syt7
Csnk1d
Ten1
Adk

Nfu1
Lpar1
Prkar1b
Hnrnpdl
Lamp2
Slc25a11
Camkv
Rnf181
Ppa1
Sult4a1
Zer1
Gas5
B4gat1
Hcn2
Gpbp1
Atp6v1g2
Mtus1
Snx5
Tmem208
Arhgef4
Sh3gl2
Calm2
Phldb1
Sptssa
Emc3
Wipf3
Hypk
Ccl27a
Spock2
Ei24
Prxl2b
Egln1
Commd4
Ttc9b
Znhit1
Paqr4
Apc
Cdipt
Arhgap33
Gria1
Oaz2
Sox2ot
Mapk8ip2
Pttg1ip
Cdk5r2
Pld3
Ssr3
Rbm3
Svip
Mrpl12
Pacsin1
Trim32

Map4
S1pr1
Ube2ql1
Rgs4
Prrt1
Clcn3
Map7
Ctxn1
Smim11
Nrsn2
Sugt1
Gda
Pde6d
Kcnab1
Rap1b
Sec13
Kdelr1
Usp54
Ndrg4
Rbbp4
Slain1
Dync1i2
Grhpr
Gm2a
Kif1c
Atp2b1
Ngef
Gstp1
Atg3
Echs1
Secisbp2l
Ppp1r2
Necab2
Ctdsp2
Ppt1
Cox5a
Plcl1
Gsk3a
Dnm1l
Tmem88b
Ppp2r2a
Dnajb2
Arhgef17
Commd6
Zfp207
Prmt2
Dctn4
Sulf2
Cavin3
Ociad2
Ncoa4
Car14

Rnf208
Mboat7
Chchd6
Gna12
Chgb
Cbx6
Lrrc17
Tfg
Dixdc1
Vkorc1
Scn2b
Tomm70a
Dnajc15
Kif5a
Tmem33
Ddx1
Tspan5
Ermn
Kcnj10
Il18
Nipa1
Sbf1
Sbds
Polr3h
Plin3
Gng11
Klhdc3
Bcap31
Hnrnph2
Pogk
Epas1
Iqsec1
Klf13
Camta1
Btbd1
Add3
M6pr
Gng3
Sumo3
Sema4d
Nrxn2
Slc12a2
Atp6v1a
Clk1
Gcsh
Sucla2
Psmc1
Stx1b
Pak2
Herc1
Atp6v1d
Ppp1r14b

Eif3e
Snhg12
Ncdn
Mtss2
Grn
Efnb3
Oxsr1
Spock3
Snrnp27
Ppp2r1a
Eml1
Kif3c
Syf2
Tmx4
Dlg4
Clip2
Disp2
Ypel5
Rufy3
Rit2
Bnip3
Sox10
Ppp1r11
Fnta
Scrn1
Map2k1
Papss1
Hectd4
Sh3glb1
Psph
Vstm2l
Adh5
Prpf19
Dip2a
Sfpq
Ly6e
Nptx1
2210016L21Rik
Serpinb6a
Slc25a12
Prrc2b
5031439G07Rik
Polr2l
Rnf5
Tmem98
Hk1
Pgam1
Zmiz2
Rbbp6
Tmem160
Scamp1
Slc24a2

Pcna
Eif1ax
Cyp46a1
Gja1
Dtx3
Slc25a23
Mbnl1
Agpat4
Rida
Pkig
Clmn
Mycbp2
Dcaf8
Cpd
Rasgrf1
Mink1
Camk2g
Tmcc3
Gabrd
Spin1
Fgfr2
Gclm
Dlgap3
Syngr1
Ptgds
Nsfl1c
Fnbp1
Hnrnpr
Retreg2
Lxn
Rap1a
Tmed4
Spryd3
Tmcc2
Pcif1
Arrb1
Slc35b1
Arl1
Magee1
Ubald1
Sh3glb2
Trappc2l
Paip2b
Akr7a5
Nckap1
Maf1
Ppme1
Hnrnph3
Amer2
Tmem158
Fam168b
Epdr1

Abhd17b
Fbxo9
Podxl2
Trpc4ap
Glrx5
Arpc2
Kctd3
Trim3
Etv1
Hexa
Dnm2
Vamp3
Baalc
Cldnd1
Tpd52
Jup
Dnajc5
Actr10
Ppm1a
Ctnnd2
Commd1
Dtna
Acot13
Napg
Mcts1
Spag9
Ddit4
Stard3nl
Rexo2
Rnf141
Mtmr6
Gab1
Metap2
Mtfr1l
Snap47
S100a6
Mrpl53
Ly6a
Alcam
Anln
Taok1
Polr2c
Psd3
Stk11
Dlg2
Kmt5a
Celsr2
Tmod1
Psma1
Pfkp
Ppp2r5c
Adipor2

Cxxc5
Gps1
Zeb2
Gprasp1
Epn2
Ndufa4
Cyb5r3
Slu7
Cdk19
Cdk2ap2
Ufc1
Eif3a
Tulp4
Ap1s1
Cenpb
Abi2
Asrgl1
Znrf1
Hnrnph1
Bag6
Cs
Ptprs
Fkbp8
Tspan15
Smox
Txnl1
Ccp110
Ensa
Pdk2
Ift20
Nsa2
Gprc5b
Slc32a1
Dner
Dnlz
Tmbim1
Srsf1
Hopx
Ppp1r14a
Abhd8
Txnl4a
Rnh1
Enoph1
Ttc3
Lzts2
Tpm1
Csnk2b
Hdhd2
Gad1
Cops6
Mapre3
Tnfaip6

Anp32e
Psat1
Tsg101
Tpd52l2
Epn1
Rad23b
Mrpl28
Gfap
Mrps14
Rgs7bp
Gap43
Itpk1
Gpr62
Il33
Mrpl24
Nkx6-2
Parp6
Pin1
Mrfap1
Itm2c
Cbarp
Pdcd5
Tcf25
Vdac1
Clasp2
Pllp
Ndufb5
Mapk8ip3
Eif4a3
Ppp1r16b
Uqcc2
Rplp0
Lrpap1
Adcy5
Lrrc8b
Gm10076
Mrpl42
Fbxl3
Adi1
Slc6a9
Rangap1
Frmd8
Sh3bgrl
Agap1
Hsd17b4
Trf
Smarcd1
Ola1
Abr
Dynlt3
Reep3
Mcee

Ntrk2
Arpc3
Ufd1
Ttyh2
Hnrnpf
Nsg1
Tubb3
Enpp2
Cuedc2
Capzb
Gprasp2
Tceal9
Snrpc
Eif3b
Derl1
Uqcrc1
Celf4
Dync1h1
Olfm1
Mfge8
Higd1a
Ndufa5
Gng2
Hp1bp3
Enpp5
Limch1
Pcmt1
Slc1a2
Opa1
Rnf187
Gng7
Adipor1
Gpr37
Atrn
Cmas
Trim59
Opalin
Metrn
Oxct1
Psmd8
Mapre1
Scnm1
Lap3
Arl3
BC005537
Nbea
Snx1
Itgav
Cdc42ep2
Adar
Rheb
Prmt1

Prkcsh
Epb41l1
Nkain4
Ktn1
Pdcd6
Smarca2
Bckdha
Fbxo44
Selenoh
Clcn4
Tmed9
Gnl1
Pja1
Kctd2
Mrpl54
Mag
Ube2e1
Glrx3
Kif21a
Gsk3b
Aig1
Diras2
Limd2
Snapc5
Sgta
Cfl2
Sirpa
Tra2b
Mrps34
Dnaja2
Nmral1
Pigq
Ap2m1
Nfix
Psmd2
Nucks1
Cltc
Flywch1
Pip4p1
Fcer1g
Phlpp1
Ddt
Copg1
Cd82
Psma4
Lpgat1
Plxdc2
Kazn
Tmem50b
Peg3
Ldha
Nrxn1

Slc25a5
Tex264
Tcaf1
Slc38a2
Atp6v0b
Smarcd3
Rad23a
Pdha1
Ech1
Vxn
Usp22
Ociad1
Pfn1
Gabbr1
Mrpl41
Cdc37
Rpn1
Nudt9
Chchd3
Ndufb8
Synj1
Emc8
Rhou
Dph3
Ankrd40
Hapln2
Coa3
Sema6a
Gabarapl1
Mrpl11
Pkp4
Chd3
Msrb1
P4hb
Trnp1
Cycs
St13
Cdk16
Plaat3
Ermp1
Tm7sf3
Dlg1
Tprn
Mrpl23
Efcab14
Ccdc12
Bmyc
Npc1
Cbr1
Wipi2
Mrps36
Ube2d1

Plxnb1
1500004A13Rik
Agpat3
Foxn3
Rab1b
Tsn
Apod
Pcyt2
C1qc
Myrf
Srp19
Psmd14
Trappc6b
Kidins220
Mydgf
Ehmt2
Rgs10
1190005I06Rik
Glul
Dctn6
Ssu72
Tsc22d4
Pfdn6
Nudt19
Ccdc47
Pdxp
Banf1
Sf1
Trim44
Emc1
Gpi1
Hdac5
Sap18
Agap3
Dzank1
Mrps24
Bmerb1
Rcbtb1
Polr1d
Ap3d1
Desi1
Nop53
Dlg3
Emc4
Mgrn1
1110004F10Rik
Cct8
Abhd17a
Ndufv1
Smarca4
Atp6v1c1
Mprip

Thrsp
Ndufb6
Mtpn
Ube2v1
Rnf220
Hspa9
Tubb4b
Grk2
Mtch2
Csrp1
Tafa5
Chchd1
Actr3
Ube2n
Ost4
Mrpl30
Jph4
Tmem63a
Smpd1
Pnck
Ablim2
Slc39a10
Mrps18c
Mast3
Ppid
Sesn3
Rnf13
Dpy19l1
Tomm22
Pebp1
Idh3g
Pttg1
Pon2
Lancl1
Rapgef4
Myl12a
Gnb1
Arsg
Ank3
Slc1a3
Ube2s
Nenf
Cdkn2d
Vps4a
Gng13
Arhgef10
Ank
Tmx2
Tmed7
Arf5
Azin1
C2cd2l

Csnk1g2
Pum2
Rbbp7
Tkt
Tspyl1
Ubqln2
Nono
Bod1
Hnrnpa0
Grb2
Atf4
Pfdn1
Arl8a
Dhrs1
Fbxl5
Mrps18a
Vps29
Bpgm
Acsbg1
Cyth1
Mapk1
S100b
Psmc5
Crmp1
Smim10l1
Kcna6
Stard10
Kifap3
Mrps12
Ptms
Mkrn1
Rbm39
Rpn2
Pdxk
Csnk1a1
Bcas2
Nbr1
Endod1
Ogt
Sort1
Prkaca
Ube2v2
Cdk5
Hagh
Mrpl48
Eif4a1
Acox1
Ubr4
Cntnap1
Nfasc
Ppp1r1a
Mrpl51

Prkacb
Zfp771
Vegfb
Polr2g
Ddx17
Srsf6
Gtf2i
Paics
Elovl1
Vps41

WMT cTBS

DEGs Young sham vs Aged sham	cTBS DEGs unique to young adults	cTBS DEGs unique to young adults that are also ageing DEGs
Mobp	Lrrc17	AI593442
Mbp	Tatdn1	Adgrl1
Uba52	Tspyl4	Apod
Cabp1	Ost4	Arpc2
Plekhb1	Snrpf	Atp6v1a
Bcas1	Phpt1	Bloc1s1
Chn1	Snhg9	Cadps
Hpca	Araf	Calb1
Scn1b	Bloc1s1	Cd81
Bsn	Rps28	Cdk5r2
Slc17a7	Snap25	Celf5
Tesc	Dcaf17	Chd3
Lingo1	Ndufa1	Clasp2
Ppp3r1	Car2	Cnp
Nrgn	Rps27	Cpe
Nptxr	Rps29	Cryab
Mef2c	Rpl9-ps6	Dcaf17
Rpl9-ps6	Zwint	Disp2
Phf24	Sparc	Dlg2
Eef1a2	Ppp1r16b	Dmtn
Sv2b	Scg2	Dynlt3
Gria3	Gja1	Dzank1
Cacnb3	Hectd4	Eno1
Lamp5	Srsf5	Epdr1
Pvalb	Ptprz1	Evl
Cadm3	Actr2	Ewsr1
Mal	Snhg6	Fbxl16
Mt1	Atp1a3	Gad1
Dnm1	Kctd13	Gapdh
Rprml	Slc6a1	Gja1
Camkk2	Ubqln2	Glul
Plk2	Isca1	Grk2
Pi4ka	Rnf5	Gsk3b
Trp53inp2	Pacsin1	Gsn
Atp1a1	Tomm7	Hectd4
Fabp3	Impact	Hnrnpa0
Itpka	Smim26	Homer1
Tceal5	Eif4a2	Ina
Nat8l	Ndrg4	Jph4
Prr18	Tsc22d4	Khdrbs3
Tmeff2	Spag9	Lmo4
Tmsb4x	Atp6v1a	Lrrc17
Tceal6	Txn1	Lynx1
Bloc1s1	Glul	Mag
Kcnab2	Wdr6	Magee1
Ptk2b	Cox17	Malat1
Pdlim2	Jph4	Mapk9
Pdp1	Hmgb1	Meg3
Matk	Basp1	Mog

Lmo4
Clic4
Camk2b
Ppp3ca
Sri
Cnp
Rps29
Stx1a
Lin7b
Svop
Brinp1
Mmp17
Grin1
Kalrn
Snhg6
Cplx1
Rnf112
Pgm2l1
Calm1
Tspan2
Thy1
Spock1
Nell2
Gpr162
Cx3cl1
Pea15a
Fam131a
Cdk5r1
Ptprz1
Egr1
Golga7
Polr3e
Rell2
Cap2
Fxyd1
Snap25
Atp2b2
Pkm
Syn1
Kcnip2
Trak2
Habp4
Arf3
Snph
Myh10
Celf2
Marcksl1
Mrtfb
Slc6a17
Evi2a
Rbfox3
Itpr1
Chmp5
Sdhd
Snhg8
Sdcbp
Tmeff1
Atp5md
Zcchc12
Nedd4
Clasp2
Scn4b
Huwe1
Agap2
Fus
Selenof
Cdk5r2
Ddn
Cox7c
Dmtn
Camk2n1
Stxbp1
Slc4a4
Ank2
Psma5
Nrxn1
Tuba1b
Ndufa3
Sem1
Hprt
Ina
Gas7
Gsk3b
Polr2g
Peg3
Atp5g3
Id2
Snca
Usp50
Smarca2
Hnrnpa0
Pip5k1c
Sparcl1
Epdr1
Usp9x
Hpcal4
Hnrnpa2b1
Meg3
Pde10a
Paip2
App
Cadm4
Erh
Map2
Mycbp2
Myt1l
Ndrg4
Ndufb8
Necap1
Nrxn1
Opalin
Ost4
Pacsin1
Pdxk
Peg3
Phyhip
Pip5k1c
Polr2g
Ppp1r16b
Prkaca
Ptgds
Ptprz1
Rheb
Rian
Rit2
Rnf5
Rpl4
Rpl9-ps6
Rps12
Rps27
Rps28
Rps29
Scg2
Sdcbp
Slc44a1
Smarca2
Smim10l1
Snap25
Snca
Snhg11
Snhg6
Snhg9
Spag9
Spryd3
Stxbp1
Sucla2
Tatdn1
Tcaf1
Tmeff1
Tmx4
Tomm70a
Trf
Tsc22d4
Tsnax
Ubqln2

Tppp3	Phyhip
Syp	Id4
Tmem132a	Rps27rt
Scrg1	Cpox
Camk2a	Kif5c
Cyfip2	Oxr1
R3hdm1	Rraga
Ier5	Phyhipl
Prkcg	Magee1
Cplx2	Bzw1
Cck	Prpf8
St3gal5	Slc25a22
Rasgef1a	Cystm1
Gnb5	Tmx4
Calb1	Rheb
Carhsp1	Dzank1
Snhg9	Rian
Zbtb18	Calb1
Nrxn3	Cops7a
Fa2h	Snhg11
Gls	Mcam
Sccpdh	Grk2
Lynx1	Ndfip2
Gabrg2	Sec61g
Car11	Psip1
Ncs1	Prkaca
Myo6	Necap1
Gamt	B230312C02Rik
Cacng3	Emc7
Bcat1	Fbxl16
Phgdh	Cacybp
Baiap2	Rit2
Rab31	Disp2
Plcb1	Unc80
Ak3	Arpc2
Pianp	Idi1
Jph3	F3
Reep2	Mgat3
AI593442	Acyp2
Ugt8a	Bpnt1
Tns3	Gad1
Adcy1	Evl
Tac1	Med30
Serpini1	Rtl8a
Nsf	Mapre2
Tmem59l	Rab28
Mog	Tsnax
Josd2	Prkar1a
Myt1l	Naa20
Grina	Bcl2l2
Madd	Gm20594
Timm22	Adgrl1

Gapdh	Prrc2a
Pcsk2	Gtf2a1
Snx10	Sucla2
Napb	Dynlt3
Mapk10	Smim10l1
Tpi1	Frrs1l
Sncb	Atp6v1h
Litaf	Mapk9
Ywhah	Rab3c
Dgkz	Pdxk
Dbi	Homer1
Grik5	AI593442
Tbc1d9b	Lynx1
Tatdn1	Morf4l1
Tmeff1	Khdrbs3
Cpe	Cadps
Icam5	Tomm70a
Gng12	Chd3
Snca	Fmc1
BC004004	Lmo4
Ddah1	Tcaf1
Cmtm5	Abhd3
Rgs9	Mycbp2
Rasgrp1	Myt1l
Syngr3	Pkia
Nfe2l1	Ip6k1
Rps28	Dlg2
Dcaf17	Arpc4
Setd7	Cpe
Ptprn	Inpp4a
Igfbp5	Wdr89
Peg13	Celf5
St8sia3	Spryd3
Grb14	Ewsr1
Cdk2ap1	Snrpn
Cldn11	Atn1
Tusc3	Rps19
Gabrb3	Eif1
Ckmt1	Ndufb9
Gnal	Ndufb8
Paqr8	Smdt1
Tspan13	Rpl23a
Slc2a1	Rpl27a
Syt4	Cd81
Qdpr	Rpl5
Dmtn	Rpl7
Sh3gl3	Rps6
Ugp2	Rpl17
Ppp1r9a	Aplp1
Dbn1	Cnp
Cat	Rps15a
Atp6v0e	Rplp1

Phyhip	Mog
Cdc37l1	Eno1
Rnf157	Rps2
Aak1	Slc44a1
Mgst3	Gapdh
Lamp1	Rpl4
Aktip	Pacs2
Tsnax	Rpl9
Arl6ip5	Rps3a1
Dock10	Fkbp2
Gabbr2	Tubb4a
Kcnj4	Rpl8
Rps27	Rps15
Arhgef9	Ptma
Ptpn11	Rpl32
Vbp1	Opalin
Rab15	Pfdn5
Ncor2	Rpl35a
Gnai1	Eef1b2
Tmem47	Rps11
Ewsr1	Ptgds
Enc1	Rpl18
Ptpn5	Tpt1
Unc50	Rps14
Eno2	Rpl6
Nkiras1	Rpl26
Id3	Rpl23
Pip5k1c	Rps16
Tra2a	Rpl28
Fhl1	Rps4x
Myo5a	Trf
Aqp4	Rps8
Cd9	Gjb1
Neurl1a	Rpl24
Reps2	Rpl13
Rasl10b	Rpl11
Stmn3	Rpl18a
Anp32b	Rpl30
Efhd2	Cryab
Bex2	Rps12
Ppp2r5a	Rps5
Rusc1	Rps27a
Cadps	Rps10
Cfap36	Rps7
2310022B05Rik	Rps20
Scg2	Rpl14
Rab3a	Mag
Ivns1abp	Rps3
Dlgap1	Gsn
Syt13	Rpl13a
Rpl4	Fau
Mt2	Rpl21

Tnks2
Oip5os1
Rasd2
Homer1
Strn4
Ywhaz
Dock3
Tmco1
Nefm
Tubb5
Tmem179
Evl
Fyn
Kcnq2
Rab6b
Dbp
Chga
Tuba4a
Plekhj1
Zfas1
Clstn3
Ednrb
Syn2
Add2
Dlgap4
Gsn
Kndc1
Pja2
Got2
Celf5
Cryab
Serinc5
Ddit3
Daam2
Rhog
Cadm2
Shtn1
Dgkb
Vamp2
Churc1
Rps12
Lrrn1
Mapk9
Sar1b
Adgrb2
Prkcb
Map4k4
Unc13a
Adgrl1
Kcnk1
Atp6v1b2
Zfand5
Rps24
Apod
Rpl10
Malat1
Hba-a2
Hba-a1
Hbb-bs
Ttr

Pip4k2a
Otub1
Scd1
Asphd2
Sptbn2
Egr3
Fgf1
Idh3a
Fads2
Enah
Cnn3
Atxn7l3
B3gat3
Usp14
Arrdc3
Stk25
Scg5
Aamdc
Camta2
Scn2a
Cntn2
Sorl1
Anapc2
Nptn
Rdx
Nsg2
Wnk1
Stxbp1
Dkk3
Sc5d
C1qtnf4
Gal3st1
Ap1b1
Cyb5b
Fam234b
Zfp664
Ptprd
Kctd17
Got1
Dclk1
Prkce
Tcf4
Actr1b
Rnf130
Meg3
Atp6v0c
Picalm
Clstn1
Gpr88
Rad21
Elovl5
Adgrb1

Rian
Chst1
Nat14
Ralgds
Rps27l
Astn1
B230219D22Rik
Wasf1
Ogdh
Epb41l2
Pltp
Syt5
Fxyd7
Abhd4
Txndc9
Necap1
Ankrd13a
Plekhh1
Ndrg1
Ado
Snhg11
Plp1
Aspa
Med21
Smim13
Cdc42se2
Arhgap23
Malat1
Chn2
Ddr1
Taf13
Eif6
Mllt11
Nrbp2
Ctnna1
Tprkb
Rnf227
Olig2
Pdxdc1
Ap2a2
Strn
Nrcam
S1pr5
Vgf
Mmd
Cd81
Pou3f3
Sirt2
Rph3a
Nefl
Olig1
Cd164

Plekha1
Cdk17
Gstm7
Myl12b
Scarb2
Nhp2
Srpk2
Srsf11
Ppp2r3a
Efhd1
Gjc2
Snn
Ptk2
Pdhb
Mras
Jam3
Hepacam
Hadha
Atp6v1e1
Nacc2
Gltp
Sh2d5
Micall1
Snap91
Arpc1b
Khdrbs3
Pop5
Slc48a1
Mrpl17
Arhgdig
Fdx2
Snapin
Gabra1
Pam16
Eno1
Ufm1
Rnps1
Rtkn
Haghl
Timm23
Arpp21
Tmem134
Igfbp6
Smap2
Mrpl4
Zcchc24
Mrps26
Eci1
Adcyap1r1
Cers2
Strbp
Arhgap5

Tmem229a
Phf5a
Asns
Erbin
Ddx24
Pop4
Phlda3
Lrp1
Trim9
Pim3
Pik3r1
Pfkl
1700047M11Rik
Cxcl14
Gnptg
Slc44a1
Rpl37a
Ccdc124
Ina
Gjc3
Pdcd10
2900052N01Rik
Tusc2
Mat2b
Stard7
Dync1i1
Wscd1
Lrrc58
Kifc2
Rpl35
Sdcbp
Atp1b1
Usp5
Aldh6a1
Sptbn4
Anxa5
Adgrg1
Schip1
Ybx3
Fez1
Ifi27
Tmbim4
Ssbp4
Selenop
Hras
Trim37
Fbxl16
Rogdi
Syt7
Csnk1d
Ten1
Adk

Nfu1
Lpar1
Prkar1b
Hnrnpdl
Lamp2
Slc25a11
Camkv
Rnf181
Ppa1
Sult4a1
Zer1
Gas5
B4gat1
Hcn2
Gpbp1
Atp6v1g2
Mtus1
Snx5
Tmem208
Arhgef4
Sh3gl2
Calm2
Phldb1
Sptssa
Emc3
Wipf3
Hypk
Ccl27a
Spock2
Ei24
Prxl2b
Egln1
Commd4
Ttc9b
Znhit1
Paqr4
Apc
Cdipt
Arhgap33
Gria1
Oaz2
Sox2ot
Mapk8ip2
Pttg1ip
Cdk5r2
Pld3
Ssr3
Rbm3
Svip
Mrpl12
Pacsin1
Trim32

Map4
S1pr1
Ube2ql1
Rgs4
Prrt1
Clcn3
Map7
Ctxn1
Smim11
Nrsn2
Sugt1
Gda
Pde6d
Kcnab1
Rap1b
Sec13
Kdelr1
Usp54
Ndrg4
Rbbp4
Slain1
Dync1i2
Grhpr
Gm2a
Kif1c
Atp2b1
Ngef
Gstp1
Atg3
Echs1
Secisbp2l
Ppp1r2
Necab2
Ctdsp2
Ppt1
Cox5a
Plcl1
Gsk3a
Dnm1l
Tmem88b
Ppp2r2a
Dnajb2
Arhgef17
Commd6
Zfp207
Prmt2
Dctn4
Sulf2
Cavin3
Ociad2
Ncoa4
Car14

Rnf208
Mboat7
Chchd6
Gna12
Chgb
Cbx6
Lrrc17
Tfg
Dixdc1
Vkorc1
Scn2b
Tomm70a
Dnajc15
Kif5a
Tmem33
Ddx1
Tspan5
Ermn
Kcnj10
Il18
Nipa1
Sbf1
Sbds
Polr3h
Plin3
Gng11
Klhdc3
Bcap31
Hnrnph2
Pogk
Epas1
Iqsec1
Klf13
Camta1
Btbd1
Add3
M6pr
Gng3
Sumo3
Sema4d
Nrxn2
Slc12a2
Atp6v1a
Clk1
Gcsh
Sucla2
Psmc1
Stx1b
Pak2
Herc1
Atp6v1d
Ppp1r14b

Eif3e
Snhg12
Ncdn
Mtss2
Grn
Efnb3
Oxsr1
Spock3
Snrnp27
Ppp2r1a
Eml1
Kif3c
Syf2
Tmx4
Dlg4
Clip2
Disp2
Ypel5
Rufy3
Rit2
Bnip3
Sox10
Ppp1r11
Fnta
Scrn1
Map2k1
Papss1
Hectd4
Sh3glb1
Psph
Vstm2l
Adh5
Prpf19
Dip2a
Sfpq
Ly6e
Nptx1
2210016L21Rik
Serpinb6a
Slc25a12
Prrc2b
5031439G07Rik
Polr2l
Rnf5
Tmem98
Hk1
Pgam1
Zmiz2
Rbbp6
Tmem160
Scamp1
Slc24a2

Pcna
Eif1ax
Cyp46a1
Gja1
Dtx3
Slc25a23
Mbnl1
Agpat4
Rida
Pkig
Clmn
Mycbp2
Dcaf8
Cpd
Rasgrf1
Mink1
Camk2g
Tmcc3
Gabrd
Spin1
Fgfr2
Gclm
Dlgap3
Syngr1
Ptgds
Nsfl1c
Fnbp1
Hnrnpr
Retreg2
Lxn
Rap1a
Tmed4
Spryd3
Tmcc2
Pcif1
Arrb1
Slc35b1
Arl1
Magee1
Ubald1
Sh3glb2
Trappc2l
Paip2b
Akr7a5
Nckap1
Maf1
Ppme1
Hnrnph3
Amer2
Tmem158
Fam168b
Epdr1

Abhd17b
Fbxo9
Podxl2
Trpc4ap
Glrx5
Arpc2
Kctd3
Trim3
Etv1
Hexa
Dnm2
Vamp3
Baalc
Cldnd1
Tpd52
Jup
Dnajc5
Actr10
Ppm1a
Ctnnd2
Commd1
Dtna
Acot13
Napg
Mcts1
Spag9
Ddit4
Stard3nl
Rexo2
Rnf141
Mtmr6
Gab1
Metap2
Mtfr1l
Snap47
S100a6
Mrpl53
Ly6a
Alcam
Anln
Taok1
Polr2c
Psd3
Stk11
Dlg2
Kmt5a
Celsr2
Tmod1
Psma1
Pfkp
Ppp2r5c
Adipor2

Cxxc5
Gps1
Zeb2
Gprasp1
Epn2
Ndufa4
Cyb5r3
Slu7
Cdk19
Cdk2ap2
Ufc1
Eif3a
Tulp4
Ap1s1
Cenpb
Abi2
Asrgl1
Znrf1
Hnrnph1
Bag6
Cs
Ptprs
Fkbp8
Tspan15
Smox
Txnl1
Ccp110
Ensa
Pdk2
Ift20
Nsa2
Gprc5b
Slc32a1
Dner
Dnlz
Tmbim1
Srsf1
Hopx
Ppp1r14a
Abhd8
Txnl4a
Rnh1
Enoph1
Ttc3
Lzts2
Tpm1
Csnk2b
Hdhd2
Gad1
Cops6
Mapre3
Tnfaip6

Anp32e
Psat1
Tsg101
Tpd52l2
Epn1
Rad23b
Mrpl28
Gfap
Mrps14
Rgs7bp
Gap43
Itpk1
Gpr62
Il33
Mrpl24
Nkx6-2
Parp6
Pin1
Mrfap1
Itm2c
Cbarp
Pdcd5
Tcf25
Vdac1
Clasp2
Pllp
Ndufb5
Mapk8ip3
Eif4a3
Ppp1r16b
Uqcc2
Rplp0
Lrpap1
Adcy5
Lrrc8b
Gm10076
Mrpl42
Fbxl3
Adi1
Slc6a9
Rangap1
Frmd8
Sh3bgrl
Agap1
Hsd17b4
Trf
Smarcd1
Ola1
Abr
Dynlt3
Reep3
Mcee

Ntrk2
Arpc3
Ufd1
Ttyh2
Hnrnpf
Nsg1
Tubb3
Enpp2
Cuedc2
Capzb
Gprasp2
Tceal9
Snrpc
Eif3b
Derl1
Uqcrc1
Celf4
Dync1h1
Olfm1
Mfge8
Higd1a
Ndufa5
Gng2
Hp1bp3
Enpp5
Limch1
Pcmt1
Slc1a2
Opa1
Rnf187
Gng7
Adipor1
Gpr37
Atrn
Cmas
Trim59
Opalin
Metrn
Oxct1
Psmd8
Mapre1
Scnm1
Lap3
Arl3
BC005537
Nbea
Snx1
Itgav
Cdc42ep2
Adar
Rheb
Prmt1

Prkcsh
Epb41l1
Nkain4
Ktn1
Pdcd6
Smarca2
Bckdha
Fbxo44
Selenoh
Clcn4
Tmed9
Gnl1
Pja1
Kctd2
Mrpl54
Mag
Ube2e1
Glrx3
Kif21a
Gsk3b
Aig1
Diras2
Limd2
Snapc5
Sgta
Cfl2
Sirpa
Tra2b
Mrps34
Dnaja2
Nmral1
Pigq
Ap2m1
Nfix
Psmd2
Nucks1
Cltc
Flywch1
Pip4p1
Fcer1g
Phlpp1
Ddt
Copg1
Cd82
Psma4
Lpgat1
Plxdc2
Kazn
Tmem50b
Peg3
Ldha
Nrxn1

Slc25a5
Tex264
Tcaf1
Slc38a2
Atp6v0b
Smarcd3
Rad23a
Pdha1
Ech1
Vxn
Usp22
Ociad1
Pfn1
Gabbr1
Mrpl41
Cdc37
Rpn1
Nudt9
Chchd3
Ndufb8
Synj1
Emc8
Rhou
Dph3
Ankrd40
Hapln2
Coa3
Sema6a
Gabarapl1
Mrpl11
Pkp4
Chd3
Msrb1
P4hb
Trnp1
Cycs
St13
Cdk16
Plaat3
Ermp1
Tm7sf3
Dlg1
Tprn
Mrpl23
Efcab14
Ccdc12
Bmyc
Npc1
Cbr1
Wipi2
Mrps36
Ube2d1

Plxnb1
1500004A13Rik
Agpat3
Foxn3
Rab1b
Tsn
Apod
Pcyt2
C1qc
Myrf
Srp19
Psmd14
Trappc6b
Kidins220
Mydgf
Ehmt2
Rgs10
1190005I06Rik
Glul
Dctn6
Ssu72
Tsc22d4
Pfdn6
Nudt19
Ccdc47
Pdxp
Banf1
Sf1
Trim44
Emc1
Gpi1
Hdac5
Sap18
Agap3
Dzank1
Mrps24
Bmerb1
Rcbtb1
Polr1d
Ap3d1
Desi1
Nop53
Dlg3
Emc4
Mgrn1
1110004F10Rik
Cct8
Abhd17a
Ndufv1
Smarca4
Atp6v1c1
Mprip

Thrsp
Ndufb6
Mtpn
Ube2v1
Rnf220
Hspa9
Tubb4b
Grk2
Mtch2
Csrp1
Tafa5
Chchd1
Actr3
Ube2n
Ost4
Mrpl30
Jph4
Tmem63a
Smpd1
Pnck
Ablim2
Slc39a10
Mrps18c
Mast3
Ppid
Sesn3
Rnf13
Dpy19l1
Tomm22
Pebp1
Idh3g
Pttg1
Pon2
Lancl1
Rapgef4
Myl12a
Gnb1
Arsg
Ank3
Slc1a3
Ube2s
Nenf
Cdkn2d
Vps4a
Gng13
Arhgef10
Ank
Tmx2
Tmed7
Arf5
Azin1
C2cd2l

Csnk1g2
Pum2
Rbbp7
Tkt
Tspyl1
Ubqln2
Nono
Bod1
Hnrnpa0
Grb2
Atf4
Pfdn1
Arl8a
Dhrs1
Fbxl5
Mrps18a
Vps29
Bpgm
Acsbg1
Cyth1
Mapk1
S100b
Psmc5
Crmp1
Smim10l1
Kcna6
Stard10
Kifap3
Mrps12
Ptms
Mkrn1
Rbm39
Rpn2
Pdxk
Csnk1a1
Bcas2
Nbr1
Endod1
Ogt
Sort1
Prkaca
Ube2v2
Cdk5
Hagh
Mrpl48
Eif4a1
Acox1
Ubr4
Cntnap1
Nfasc
Ppp1r1a
Mrpl51

Prkacb
Zfp771
Vegfb
Polr2g
Ddx17
Srsf6
Gtf2i
Paics
Elovl1
Vps41
