# Supplementary figures and images for: Normal Ageing Impacts the Extent and Diversity of Neural Plasticity Induced in the Mouse Brain With Repetitive Transcranial Magnetic Stimulation
Source: Aging Cell. 2025 Sep 1;24(10):e70206. doi: 10.1111/acel.70206 (PMC12507392; doi:10.1111/acel.70206)

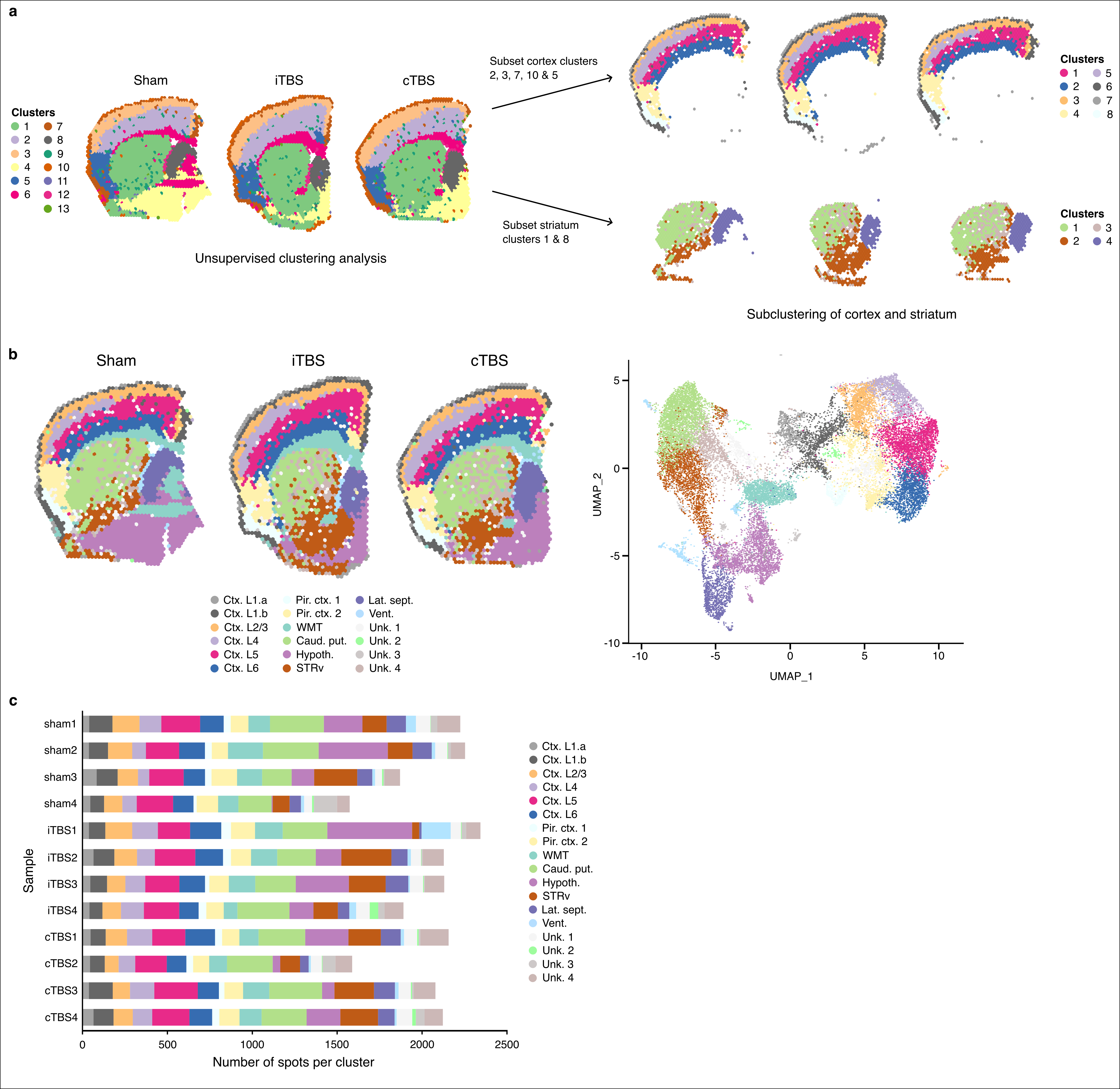

Supplement: Supplementary file 2 — Figure S1: Bulk RNA‐sequencing of the whole sensorimotor cortex of aged mice indicated little‐to‐no significant effect of stimulation on gene expression. [file ACEL-24-e70206-s002.png]

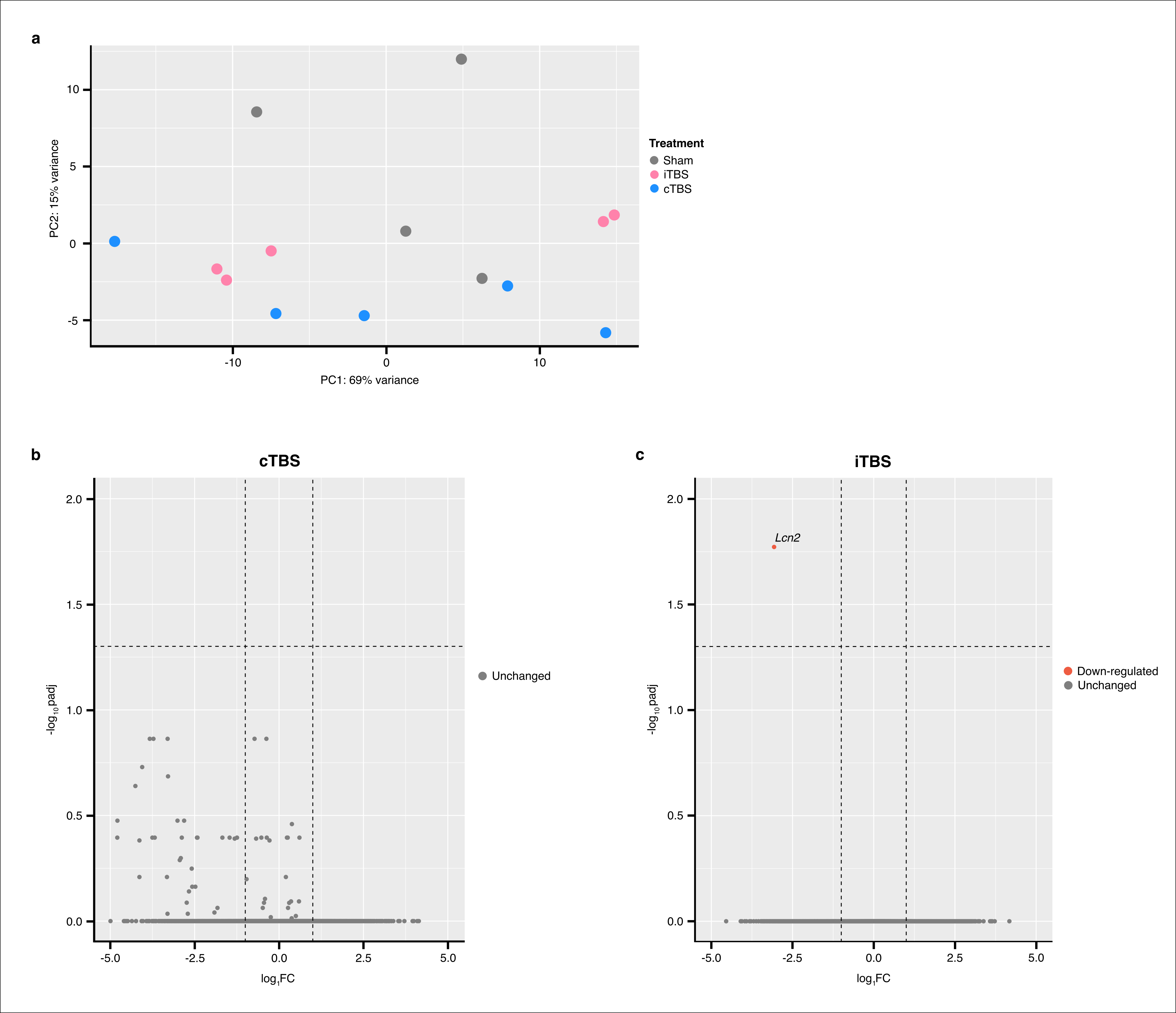

Supplement: Supplementary file 3 — Figure S2: Clustering and quality checks of spatial transcriptomics samples. [file ACEL-24-e70206-s001.png]
